# Melting Is Well-Known, but Is It Also Well-Understood?

**DOI:** 10.1021/acs.chemrev.3c00489

**Published:** 2023-11-14

**Authors:** Gijsbertus de With

**Affiliations:** Laboratory of Physical Chemistry, Eindhoven University of Technology, Het Kranenveld 14, P.O. Box 513, 5600 MB Eindhoven, The Netherlands

## Abstract

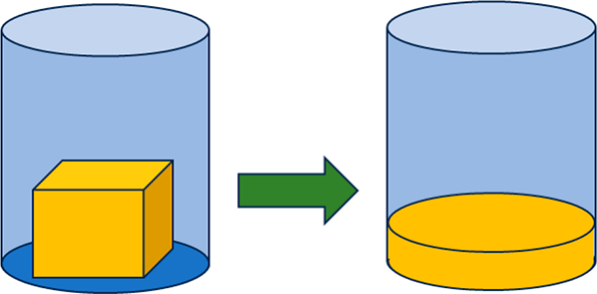

Contrary to continuous phase transitions, where renormalization group theory provides a general framework, for discontinuous phase transitions such a framework seems to be absent. Although the thermodynamics of the latter type of transitions is well-known and requires input from two phases, for melting a variety of one-phase theories and models based on solids has been proposed, as a generally accepted theory for liquids is (yet) missing. Each theory or model deals with a specific mechanism using typically one of the various defects (vacancies, interstitials, dislocations, interstitialcies) present in solids. Furthermore, recognizing that surfaces are often present, one distinguishes between mechanical or bulk melting and thermodynamic or surface-mediated melting. After providing the necessary preliminaries, we discuss both types of melting in relation to the various defects. Thereafter we deal with the effect of pressure on the melting process, followed by a discussion along the line of type of materials. Subsequently, some other aspects and approaches are dealt with. An attempt to put melting in perspective concludes this review.

## 1 Introduction

Phase transitions are important in many fields of science and technology. One typically distinguishes between first-order or discontinuous and second-order or continuous transitions. An important example of continuous phase transitions is the transition over the critical point. To describe such transitions, a generally accepted framework, renormalization group theory, is available. It seems that for discontinuous transitions such a general framework is absent. An important example of the latter type of transition is melting. The thermodynamics of melting is relatively simple and well-described by the Clapeyron–Clausius equation. It has been stated by Grimvall and Sjödin^1^ that for most practical purposes it is best to use the melting temperature *T*_mel_ as a correlation parameter for several properties, including the Debye temperature θ_D_. As will become clear from the following sections, various mechanisms have been advocated to determine *T*_mel_ which are hardly related, as each emphasizes the role of one of the defects existing in solids. In brief, although melting is ubiquitous, understanding of the “why”, “how”, and “what”, i.e., mechanistic understanding, has not been established completely, as has been emphasized through time, see, e.g., Mansoori,^2^ Vorob’ev,^3^ and Pedersen et al.^4^

Even brief browsing makes clear that the literature on melting is massive and contains many, sometimes diverging, views. Moreover, although generality often is claimed, some of the approaches are clearly dependent on the type of material. In the following sections we first discuss some preliminaries. Second, we deal with mechanical (or bulk) melting and thermodynamic (or surface-mediated) melting in relation to the various defects in solids, such as vacancies, interstitials, dislocations, and interstitialcies. Third, we discuss the effect of pressure on the melting point. These parts form the core of this review. Thereafter we change gears by discussing melting along the line of individual materials of which the individual sections typically do not contain general theory. This is followed by a discussion on three other aspects, namely history dependence, odd–even effect, and ultrafast heating, and some other one-phase and two-phase approaches. We conclude with an overall perspective on melting. Although the effect of impurities is important, e.g., for metals^5^ and geomaterials,^6^ the focus is largely on (general) mechanisms for pure three-dimensional crystalline solids, thereby avoiding details on experimental conditions, but in the sections on the various materials we highlight specifics including pressure effects for the individual materials. We generally provide details so that the reader does not need to retrieve a reference right away to check for herself or himself, but as this is fairly impossible for all references, generally one or two representative approaches are discussed in some detail, while others are more limitedly discussed or enumerated.

For a broad early overview we refer to the book by Ubbelohde,^7^ which in spite of its age, is still worth reading. Further, in modeling the Debye model and its consequences are often used, which are well in described by Grimvall,^8^ while an introduction can be found Slater’s book^9^ and Poirier^10^ provided some illuminating remarks. Also simulations are frequently referred to, the principles and details of which can be found in the treatises by Frenkel and Smit^11^ and by Berendsen.^12^

Before continuing it may be useful to indicate some previous and related reviews. The thermodynamics of melting was reviewed by Stishov,^13^ while Poirier^10,14^ discussed inorganic materials without taking interfaces into account and a review by Nabarro^15^ dealt with dislocation mechanisms only. Bilgram^16^ focused on dynamics at the solid–liquid transition, Löwen^17^ dealt with superheating, dynamics, and a comparison with colloids, while Haymet^18^ reviewed melting from the DFT point of view. Kofman et al.^19^ emphasized results on metal particles by using high sensitivity reflectance and electron microscopy measurements, while Stishov^20^ focused on the role of entropy. Finally, Mei and Lu^21^ primarily paid attention to surface effects and superheating of nanosized particles and thin films and Ram^22^ discussed equilibrium theory of molecular fluids in relation to freezing. Apart from the papers by Mei and Lu^21^ and Ram,^22^ they are all several decades old.

## 2 Some General Considerations

**Figure 1:**
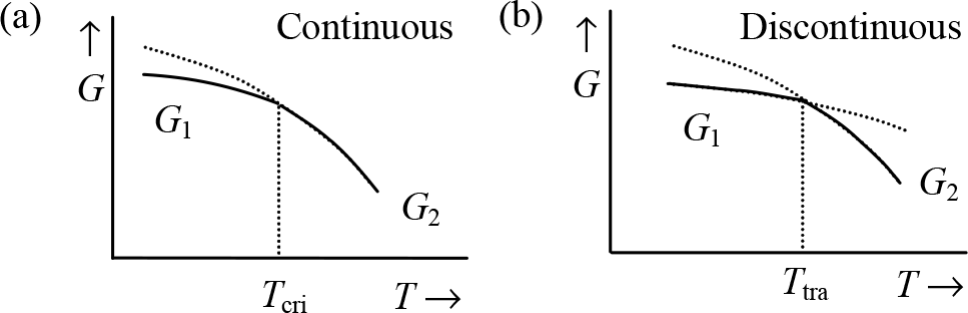
Schematic of the behavior of the Gibbs energy *G* for two phases around (a) a continuous phase transition and (b) a discontinuous phase transition. In both cases the stable states below the transition temperature have a Gibbs energy *G*_1_, while above the transition temperature the Gibbs energy is *G*_2_. The continuous transition occurs at the critical temperature *T*_cri_ with a continuous change in *G*; that is, ∂Δ*G*/∂*T* = 0, where Δ*G* = *G*_2_ – *G*_1_. The dotted line indicates the metastable continuation of the high-temperature *G* below *T*_cri_. The discontinuous transition occurs at a certain transition temperature *T*_tra_ with a discontinuous change in *G* (∂Δ*G*/∂*T* ≠ 0).

By changing the conditions—such as pressure *P* or temperature *T*—for many materials, a transition from one phase to another can be induced and, under certain conditions, two phases of the same material coexist. Since each of the two phases has its own Gibbs energy expression,^23^ under these conditions the chemical potentials of these phases are equal. The Gibbs energy *G* itself is always continuous over the transition, but the partial derivatives ∂*G*/∂*T* and ∂*G*/∂*P* may be discontinuous (Figure 1). In that case, the phase transition is denoted as *discontinuous* (or *first order*), while for the situation where the first derivative is continuous, but the higher derivatives are either zero or infinite, one speaks of a *continuous* (or *second order*) phase transition. In the past, the transitions were often labeled as first and second order according to the discontinuity of their first- or second-order derivatives of the Gibbs energy. However, the second-order “class” appeared to be more complex than anticipated, and therefore these transitions are nowadays often labeled as continuous, due to the fact that in all cases a continuous transition from a one-phase state to a two-phase state occurs with a continuous change in order parameter (Δρ for fluids) over the transition. Although the label “first order” stuck, for consistency, we refer to this transition as discontinuous, the more so since the density behavior for fluids, apart from one-component plasmas, is discontinuous over the transition.

**Figure 2:**
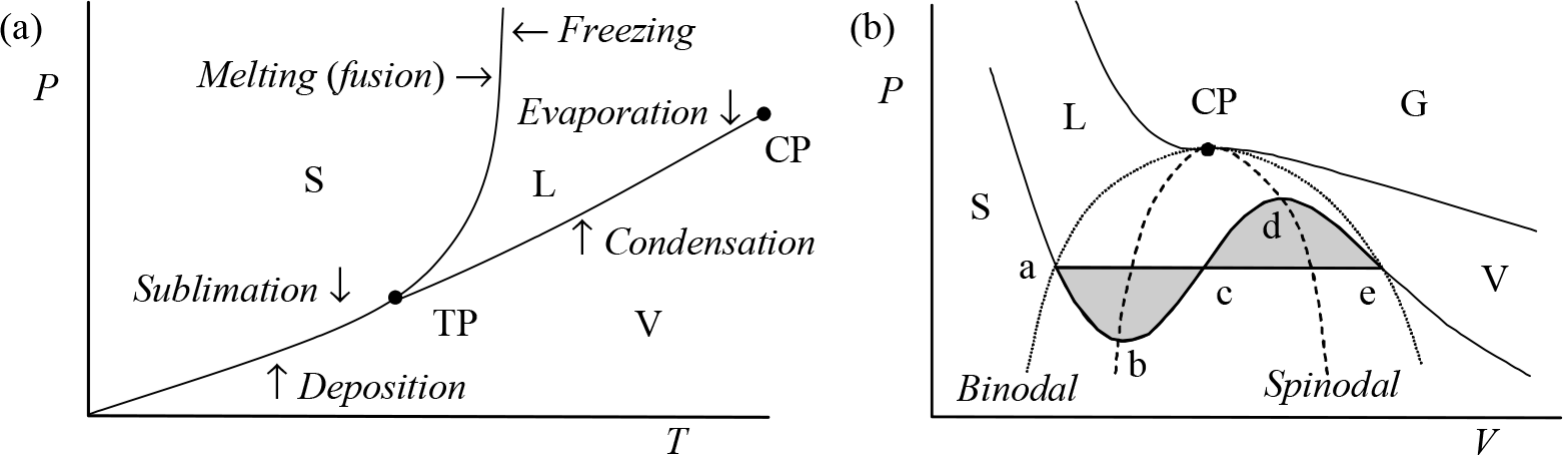
(a) Schematic of the phase equilibrium between the solid (S), liquid (L), and vapor (V) phases in the *P*–*T* plane, showing the triple point (TP) and critical point (CP). These are natural reference points since the melting temperature *T*_mel_ and the boiling temperature *T*_n_ depend on the environment, in particular the pressure *P*. For water, for example, *P*_cri_ = 218.3 atm, *T*_cri_ = 374.15 °C, ρ_cri_ = 320 kg/m^3^, and *T*_tri_ = 0.01 °C. While the transition *across* a coexistence line relates to a discontinuous phase transition, the transition over the critical point *along* the coexistence line relates to a continuous phase transition. (b) Schematic of the phase equilibrium in the *P*–*V* plane. The horizontal line indicates the equal area Maxwell construction. Above the CP only gases (G) can exist. The binodal line indicates the demarcation of global stability, while the spinodal line indicates the limits of local stability.

The angle of intersection of the *G*_1_ and *G*_2_ curves for phases 1 and 2, respectively, determines the entropy and volume change associated with the phase transitions and, hence, the type of phase transition. Experimentally, it appears that, by moving along the liquid–vapor (L–V) coexistence line over the critical point (CP), the differences in properties, in particular the density, between the liquid and gas phases vanish in a continuous way and the transition is continuous (Figure 2). Moving across the L–V curve from one phase to the other leads to a discontinuous transition.

Following the coexistence (vapor pressure) line between liquid and vapor in the *P*–*T* diagram, starting at the triple point *T*_tri_ and passing the normal boiling point *T*_n_, we end at the critical point with temperature *T*_cri_. In this process the density of the liquid ρ_L_ decreases, while the density of the vapor ρ_V_ increases. At *T*_cri_, ρ_V_ = ρ_L_. Moreover, for *T* < *T*_cri_ a meniscus—that is, a sharp transition region between liquid and vapor—is present except for temperatures close to *T*_cri_ (say, within 1 degree), where this meniscus widens and suddenly disappears at *T*_cri_. The first use of the term “critical point” was by Andrews in 1869.^24^

## 3 Discontinuous Transitions

**Figure 3:**
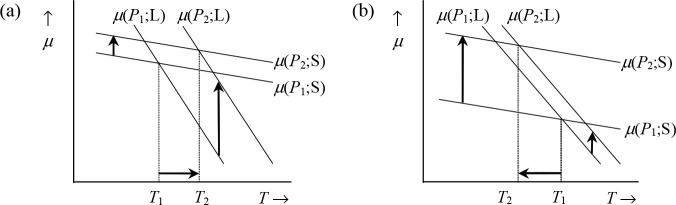
Effect of pressure on melting point. (a) *V*_m_(S) < *V*_m_(L) leading to melting point rising (*T*_2_ > *T*_1_); (b) *V*_m_(S) > *V*_m_(L) leading to melting point lowering (*T*_2_ < *T*_1_).

For equilibrium between phases the values of their chemical potential μ should be equal; otherwise transfer of matter occurs until d*G* = (μ_2_ – μ_1_) d*n* = 0, where *n* is the number of moles, is fulfilled. If we consider that each phase has its own Gibbs energy function *G*, the crossover temperatures between solid and liquid and liquid and vapor determine the melting and boiling temperatures, respectively. The effect of pressure *P* is illustrated in Figure 3, showing that, upon increasing *P*, the melting point rises if the molar volume of the solid *V*_m_(S) is less than the molar volume of the liquid *V*_m_(L), while the melting point decreases if *V*_m_(S) > *V*_m_(L).

The coexistence curves for two phases, say, L and V, in the *P*–*T* plane (Figure 2a) can be obtained from the *Clapeyron–Clausius equation* (as first given by Clapeyron in his paper in 1834, reprinted in 1843, the latter which made Carnot’s work known^25^). This equation, resulting from Δ*G* = Δ*H* – *T*Δ*S* = 0 with enthalpy *H* and entropy *S*, or equivalently μ_L_ – μ_V_ = 0, in combination with Maxwell’s relation d*P*/d*T* = d*S*/d*V*, is given by
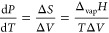
1

When liquid and vapor are both present in equilibrium, we have Δ*G* = *G*_V_ – *G*_L_ = 0. Hence, we have (Figure 2b), since *G* = *F* + *PV* with *F* the Helmholtz energy, the relation *F*_a_ – *F*_e_ = −*P*(*V*_a_ – *V*_e_). But the work required to go from vapor to liquid is also *F*_a_ – *F*_e_ = −∫_e_^a^*P* d*V*. We conclude that the (gray) area (Figure 2b) described by the curve abc must equal the (gray) area described by the curve cde. Hence, phase equilibrium is determined by the horizontal line for which these two areas are equal. Note, though, that in practice doing reversible work along the curve bcd is impossible. This is *Maxwell’s equal area rule*. *Metastable states* can occur for the ranges ab (*supersaturation*) and de (*superheating*), while the range bcd represents *unstable states*. The Clapeyron–Clausius equation evidently can be applied to solid–solid and solid–liquid transitions as well. Note that frequently the Clapeyron–Clausius equation is simplified by neglecting the liquid volume and approximating the equation of state (EoS) for the vapor phase by the perfect gas law. However, as has been noted,^26^ this may lead to significant errors.

From the above it will be clear that for a proper treatment of melting both phases should be considered. Nevertheless, many attempts deal only with the solid phase. Hoover and Ross^27^ have advanced general arguments of why such an approach might still work and to which we come in section 4. Some in principle straightforward attempts using the Gibbs energy for both phases have been put forward, usually employing relatively drastic approximations and the Maxwell construction to keep the models tractable. So far, the only model that yields flat pressure versus density isotherms in the coexistence region as well as distinct binodal and spinodal curves without invoking separately the Maxwell construction is hierarchical reference theory (HRT), to which we come in section 10.2, although also for HRT still an expression for the Helmholtz energy of the solid phase has to be provided.

## 4 Melting

Upon melting a solid, the most eye-catching change is that the solid becomes a fluid, implying a tremendous change in viscosity and loss of shear modulus.^29^ Other macroscopic properties change far less dramatically, although some microscopic properties change to a large extent as well. We first discuss a few phenomenological changes. We note upfront that a significant effort has been paid to metals and less to other materials, so that more examples from metals are used as might be expected.

To illustrate the molar volume increase upon melting, Table 1 provides some typical data. Typically, Δ*V*/*V*_S_ ≅ 10% for molecular liquids, while for metals and ionic compounds (one-component plasmas excluded) Δ*V*/*V*_S_ ≅ 4 and 20%, respectively. A simple argument^30^ indicates the reason why such an increase generally occurs. Consider an arbitrary plane in the liquid. For such a plane the arrangement of molecules must be such as to allow them to pass to another plane while still being in contact with (a number of) neighbors. If we take, for example, a close-packed configuration for this plane in which the molecule is 6-fold coordinated, the molecular area is *o*_L_ = (1/2)3^1/2^σ^2^, where σ is the diameter of the molecule. It is then not unreasonable to suppose that the volume available to the molecule in the liquid is *v*_L_ = *o*_L_^3/2^ = 2^–3/2^3^3/4^σ^3^. If we compare *v*_L_ with the volume available in the BCC lattice, *v*_BCC_ = 2^2^3^–3/2^σ^3^, we obtain *v*_L_/*v*_BCC_ = 1.05, in good correspondence with the value for, e.g., CCl_4_ (Table 1). Similarly, for the FCC structure *v*_FCC_ = 2^–1/2^σ^3^ and we obtain *v*_L_/*v*_FCC_ = 1.14, in good correspondence with the value for the inert gases (indeed crystallizing in the FCC or HCP structure). This estimate indicates clearly the general trend, but as many other factors play a role, it should not be taken too seriously.

**Table 1:** Volumes (cm^3^ mol^–1^) and Enthalpies (kcal mol^–1^) at the Melting Point *T*_mel_ (K)^a^

liquid	*T*_mel_	*V*_S_	*V*_L_	Δ*V*/*V*_S_	Δ_mel_*H*
Ne	24.6	14.03	16.18	15.3	0.080
Ar	83.8	24.61	28.14	14.4	0.281
Kr	116.0	29.65	34.13	15.2	0.391
Xe	161.4	37.09	42.69	15.1	0.549
H_2_O	273.1	19.82	18.18	–8.3	1.436
CH_4_	90.7	30.94	33.63	8.6	0.226
CD_4_	89.8	29.2	31.7	8.6	–
CCl_4_	250.4	87.9	91.87	4.5	0.577
C_2_H_4_	104.0	39.06	43.63	11.7	0.801
C_6_H_6_	278.5	77.28	88.28	11.4	2.348
C_10_H_8_	353.2	112.2	130.9	11.7	4.550
NaF	1265	16.4	21.5	31.1	7.81
NaCl	1073	29.6	37.7	27.4	7.22
KF	1133	23.4	30.4	29.8	6.28
KCl	1043	40.5	48.8	20.5	6.41
KBr	611	45.0	56.0	24.4	2.84
Bi	544	21.6	20.8	–4.8	2.51
Cd	594	13.5	14.1	4.7	1.46
Hg	234	14.1	14.7	3.6	0.58
Sn	505	16.5	17.0	2.8	1.72
Pb	601	18.5	19.4	4.8	1.22

a Data at ambient pressure from refs (9 and 28).

The ratio of the enthalpies of fusion Δ_mel_*H* and vaporization Δ_vap_*H* for molecular liquids typically has a value of 0.1–0.2, while for metals this ratio is 0.03–0.04. Molecular compounds, however, are in the solid state well-packed but in the liquid state they may rotate, which requires some extra space, and this leads to an increased average distance between the molecules. The structure of such a liquid is more open, much less ordered than the corresponding solid, and the work necessary to pull the molecules apart results in a higher value for Δ_mel_*H*.

Possibly the simplest fluid–solid transition occurs in hard sphere systems, and we follow the description as given by Lekkerkerker and Tuinier.^31^ On the one hand, we know that the fluid state EoS can be described accurately by the Carnahan–Starling EoS:^32^
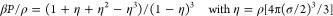
2The chemical potential is given by
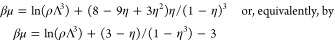
3where the thermal wavelength Λ = (*h*^2^/2π*mkT*)^1/2^ with mass *m*, Boltzmann constant *k* and Planck constant *h*.

On the other hand, we know that the solid state EoS can be reasonably well described by the Lennard-Jones–Devonshire (LJD) model.^33^ For this model the Helmholtz energy *F* = −*kT* ln *Z*, using as free volume *v*_f_ = 8(*v*^1/3^ – v_*_^1/3^)^3^ with v_*_ = 4π(σ/2)^3^/3, is given by
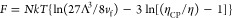
4so that the pressure becomes
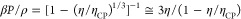
5where η_CP_ = ρπσ^3^/6 = π/3√2 ≅ 0.741 for a close-packed crystal. The chemical potential is then
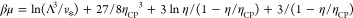
6

The coexistence criteria *P*_F_(η_F_) = *P*_S_(η_S_) and μ_F_(η_F_) = μ_S_(η_S_) yield η_F_ = 0.491 (0.494) and η_S_ = 0.541 (0.545) at a pressure of βPv_*_ = 6.01 (6.12) with in parentheses the Monte Carlo (MC) results of Hoover and Ree.^34^ A very good agreement is thus observed. For a comparable discussion, see the somewhat old, but still highly relevant, discussion on disordered materials by Ziman.^35^ Note, however, that for liquids, generally, *v*_f_ = 4π(*v*^1/3^ – v_*_^1/3^)^3^/3 is used, rendering a difference in *v*_f_ of about 50%. The thermodynamic stability is governed by maximum entropy, and the equilibrium configuration of a hard sphere system is the one that maximizes the entropy. At low density the disordered state (fluid) corresponds to maximum entropy, while at higher density crystalline arrangements are optimum. It is satisfying to observe that this transition is not only observed “in silico”, but that, with the proper precaution to realize a hard sphere system, the transition is also observed experimentally, as reported by Pusey and van Meegen;^36^ see also ref (31).

**Figure 4:**
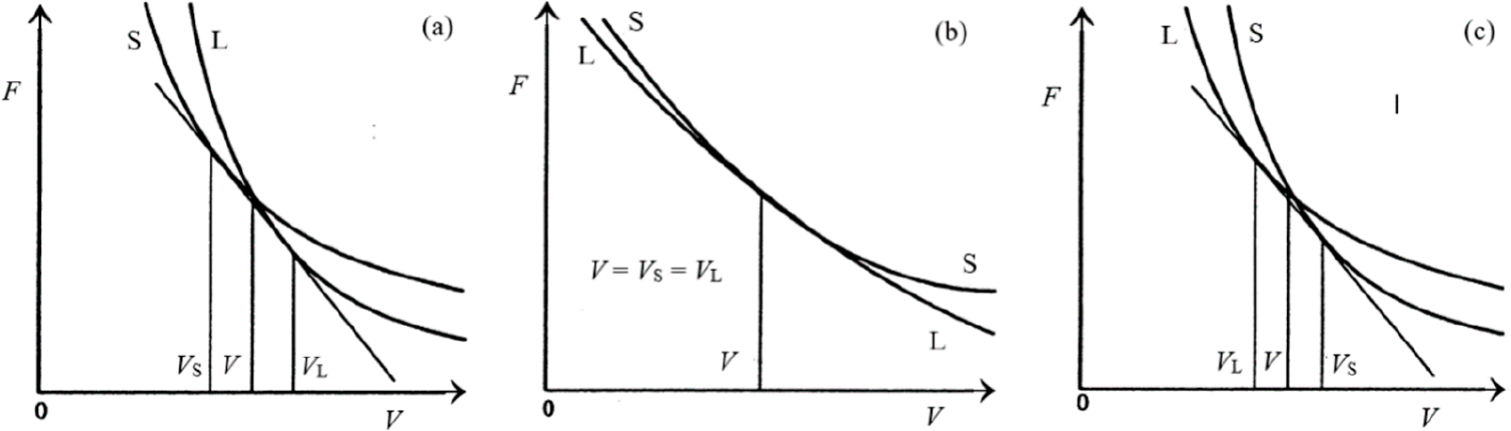
Behavior of the Helmholtz energy *F*(*V*) for solid (S) and liquid (L) around the melting temperature *T*_mel_. *V* is the volume for the crossing point, while the tangents determine the equilibrium pressures, and volumes *V*_S_ and *V*_L_. Indicated are the situations where (a) *V*_L_ > *V*_S_, (b) *V* = *V*_S_ = *V*_L_, and (c) *V*_S_ > *V*_L_. Redrawn after ref (3).

Such a two-phase approach has been given before by Vorob’ev^3^ using for the Helmholtz energy *F* for the solid state (S) the Debye approximation for the thermal contribution plus a potential energy contribution and for the liquid state (L) the Debye approximation plus a potential energy plus an entropy term related to melting, with the parameters for both phases somewhat different. The author starts a clear discussion on the crossing of Helmholtz energy *F*(*V*) for both phases by noting that both curves show a downward curvature and cross at some point. The crossing point is always in the middle of the range of two points, *V*_1_ and *V*_2_, on these curves where the tangents are identical, i.e., *P* = *P*(*V*_1_) = *P*(*V*_2_) (Figure 4a, the points *V*_1_, *V*, and *V*_2_ corresponding to the points a, c, and e in Figure 2b). At the crossing point it holds that *P*_L_(*V*) > *P*_S_(*V*) and for their derivatives *P*_L_′(*V*) > *P*_S_′(*V*) > 0. This describes “normal” melting where *V*_L_ > *V*_S_. For some parameter values the situation as depicted in Figure 4b arises and, when that is the case, *P* = *P*_S_(*V*) = *P*_L_(*V*) and *V* = *V*_S_ = *V*_L_ but with the derivatives *P*′ and *P*″ nonzero. If the crossing path changes from that of Figure 4a via that of Figure 4b to that of Figure 4c, *V*_L_ < *V*_S_ for some region of the parameter values and melting is “anomalous”. Because for the relative density differences it holds that (*V* – *V*_S_)/*V* ≪ 1 and (*V*_L_ – *V*)/*V* ≪ 1, the functions *F*_S_(*V*_S_), *F*_L_(*V*_L_), *P*_S_(*V*_S_), and *P*_L_(*V*_L_) can be represented as a power series around *V*. Keeping only first-order terms, the result is (*V*_L_ – *V*_S_)/*V* = (ζ – 1)/ζ (1 + *VP*_S_″/*P*_S_′) with ζ = *P*_L_′/*P*_S_′. The dominant contributions to *P*′ and *P*″ are due to the repulsive part of the potential energy and, if described by a power function, (*V*_L_ – *V*_S_)/*V* → *const*. if ζ → *const*. For normal melting ζ is slightly larger than unity for the initial part of the melting curve and thereafter drops, tending to a constant larger than unity. This implies that ζ varies but little along the melting curve. For anomalous melting ζ is initially also slightly larger than unity and thereafter drops, passing zero, and tending to a constant smaller than unity.

All these considerations are general, but Vorob’ev took two other steps. The first step was to estimate ζ from the model indicated, which led to ξ = (*kθ*_0_/ℏ)^2^*m*(*mV*_0_/2)^2/3^(*V*_0_/*V*)^2γ–(2/3)^(*m*/*kT*) or, approximately, *m*(*mV*)^2/3^θ_D_^2^/*γkT* = *const*., where *m* is the mass, γ is the Grüneisen parameter, and θ_D_ is the Debye temperature. Because γ is near constant, the latter relation represents the Lindemann rule (section 5.1). The second step was calculating the melting curves for Ne, Ar, Kr, and Xe as well as Na and Cs, which was done using ζ′ and *L*′ as parameters because, he argued, several assumptions and simplifications were used in the derivation. While for the noble gases, showing normal melting, the fitted value ζ = 1.18 was conform that of full calculations, the fitted value parameter *L*′ = 19 deviated considerably from the calculated value of 27.3. For Na, also showing normal melting, and Cs, showing anomalous melting, the results are satisfactory in view of the approximate nature of the theory. The main point is probably that an analytical two-phase calculation is feasible, but because, in general, an accepted liquid state model is absent and many data are required and, in this case, the model is limited to first-order expansions, the numerical results are rather approximate.

Having introduced “anomalous melting”, where the melting line shows a temperature maximum with *P* at constant *T*, it may be useful to point out that for this type of melting the solid coexists with a denser liquid, as is well-known for ice and liquid water. Usually, anomalous melting is associated with other anomalous features, such as polymorphism in the liquid and solid phases, as well as a number of thermodynamic, dynamic, and structural anomalies that include the density anomaly (the decrease in density upon cooling), the diffusion anomaly (the increase of diffusivity upon pressurizing), and the structural anomaly (the decrease of structural order for increasing pressure).^37,38^

According to Dash,^39,40^ the question of how a solid melts can be addressed using three approaches. The first states simply that melting is a discontinuous transition, in which solid and liquid can coexist at a certain pressure for each temperature, and that the process has no intermediate states. The second approach assumes that melting is a nearly continuous transition, as suggested by the small increase in volume, small enthalpy of fusion, and small change in specific heat. According to this approach, if studied with sufficient resolution, it should be possible to discern intermediate stages. The third approach acknowledges that continuous melting is typical for almost all crystals but accepts that interfaces play an important role. Limiting ourselves to the second and third approaches, the second is often addressed as mechanical or bulk melting while the third is often denoted as thermodynamic or surface-mediated melting.

Clearly, in principle any treatment dealing with melting should consider both the solid phase and the liquid phase. Hoover and Ross^27^ considered that for purely inverse power law (pair) potentials ϕ(*r*) = ε(σ/*r*)^*n*^, all properties can be scaled with the characteristic length (*V*/*N*)^1/3^ and the relative importance of any configuration in scaled space is always the same at fixed ρ(ε/*kT*)^3/*n*^. This applies to both solids and liquids. In reality attractive interactions are present, and this scaling is not obeyed. However, over a limited temperature–density interval and considering that repulsive interactions dominate, this scaling might still be approximately valid, thereby explaining why one-phase approaches might still yield reasonable results.

## 5 Mechanical or Bulk Melting

Because melting is an almost universal phenomenon, many one-phase attempts have been made to rationalize and predict melting temperatures. They were initially based on the most important aspects to consider, that is, the vibrational and lattice instabilities, often using only one of the two. Other terms contributing are (i) an electronic term, mostly for metals; (ii) an orientational term, mostly for polar molecules; (iii) a configuration term due to the distortion in shape of the molecules, mostly for nonspherical flexible molecules; and (iv) an association term due to the local microstructure of the melt, mostly for polyatomic anions. The sometimes observed melting over a certain temperature interval has been advanced to support the continuous melting hypothesis. However, impurities and polycrystallinity are often present, and both broaden the melting transition. Nevertheless, one should realize that *disorder* is present in solids, either intrinsically (point defects) or as a result of processing or thermal excitations (point defects, dislocations, orientational disorder). The characteristic energy associated with each of these defects governs their increase in number with temperature, and this increase may accelerate the approach to the melting point, although the structure remains crystalline until the transition itself. In fact, most types of these defects have been proposed as being responsible for melting. From this list of aspects, it is probably clear that a thorough discussion of all these aspects requires a book by itself. Hence, we limit ourselves to more general considerations. Since the melting curve is thermodynamically defined as the locus of the *PVT* points where the Gibbs energies (*G*) of the solid (S) and the liquid (L) are equal, and since the differences in the *G*_L_ and *G*_S_ are typically small, rather accurate and consistent approximations are required. This has limited so far the applicability of first-principle approaches to only relatively simple solids. Attempts to model more complex solids thus require other approaches and have resulted in a substantial number of mechanisms proposed.

### 5.1 Vibrational Instability

Probably the most well-known vibrational instability approach is based on a paper by Lindemann,^41^ although Lindemann himself nowhere suggested that his approach could be used to estimate the melting point, *T*_mel_. Actually, he attempted to estimate the Einstein frequency of solids ω_E_—Einstein published his theory just three years earlier—by assuming that at melting, due to their thermal motion, the molecules would just make contact.^42^ Writing for the hard sphere atom diameter σ = *d*(1 – δ), where *d* is the atom–atom distance and δ is the gap between the atoms expressed as a fraction of *d*, each atom has to be displaced on average by *σδ*/2 for contact with another atom. Equating the kinetic energy at *T*_mel_, estimated as *U*_kin_ = ∫_0_^*σδ*/2^*ax* d*x* = *aσ*^2^δ^2^/8 = (1/2)*mω*_E_^2^*u*^2^ (*a* = force constant, ω_E_ = (*a*/*m*)^1/2^), with the high-temperature limit of the Einstein (harmonic oscillator) function ε(ω;*T*_mel_) = ℏω_E_{[exp(βℏω_E_) – 1]^−1^ + 1/2} ≅ *kT*_mel_, he obtained θ_E_ = ℏω_E_/*k* = (ℏ/*k*)(8*T*_mel_/*mδ*^2^σ^2^)^1/2^. Here ℏ = *h*/2π, where *h* is Planck’s constant, *k* is Boltzmann’s constant, β = 1/*kT*, *m* is the mass of the molecule, and θ_E_ is the Einstein temperature. For CaF_2_ Lindemann estimated δ ≅ 0.05 from the dielectric permittivity by using the Clausius–Mossotti theory. Using ν_E_ = ω_E_/2π = *c*(*T*_mel_/*MV*_m_^2/3^)^1/2^, with *c* ≅ 2.06 × 10^12^ an empirical proportionality constant in cgs units, *M* the molar mass and *V*_m_ the molar volume, he further obtained a not unreasonable agreement with the experimental “Reststrahlen” frequencies, that is, the optic lattice frequencies at wave vector ***q*** = 0, for several metals and salts. Later the argument was reversed by others to estimate from *T*_mel_ from θ_E_ (or the Debye temperature θ_D_ for that matter). Actually, in 1890 Sutherland^43^ explicitly introduced the idea that *T*_mel_ is related to the displacements of the atoms, but it seems that Lindemann was not aware of this paper.

“The concept of direct contact of neighboring atoms at fusion is factitious”, as Gilvarry^44,45^ called it, and he was, it seems, the first to assume that the root-mean square displacement ⟨*u*^2^⟩^1/2^ of an atom from its equilibrium position reaches a critical fraction ξ of the nearest-neighbor distance *r*_0_ = (*V*_m_/*N*_A_)^1/3^ ≡ Ω^1/3^ of atoms at fusion, that is, ⟨*u*^2^⟩ = ξ^2^*r*_0_^2^, with ξ the (*Lindemann*−)*Gilvarry ratio*. The mean square displacement of atoms packed in a primitive cubic lattice reads ⟨*u*^2^⟩ = (3*mN*)^−1^∫_0_^ω_max_^ε(ω;*T*)ω^–2^*g*(ω)dω, and using the Debye approximation for the vibrational DoS *g*(ω) = 9*Nω*^2^/ω_D_^3^ and the high-temperature limit of ε(ω;*T*) ≅ *kT*, one obtains ⟨*u*^2^⟩ = 3*kT*/*mω*_D_^2^ = 3ℏ^2^*T*/*mkθ*_D_^2^, where ω_D_ and θ_D_ are the Debye frequency and temperature, respectively. Hence, *T*_mel_ = ξ^2^(*k*/3ℏ^2^)*mθ*_D_^2^*r*_0_^2^, which in the literature is often called the *Lindemann relation* (or *rule* or, even, *law*). The values for ξ as obtained for 10 metals are indeed approximately constant and are given by ξ_FCC_ ≅ 0.11, ξ_BCC_ ≅ 0.13, and ξ_HCP_ ≅ 0.09. Cho^46^ refined these values by considering 54 metals, leading to ξ_FCC_ ≅ 0.096, ξ_BCC_ ≅ 0.121, and ξ_HCP_ ≅ 0.069.

Before we continue it is appropriate to note that a direct comparison for various treatments for ξ is complicated by the use of ⟨*u*^2^⟩ or ⟨***uu***^T^⟩ and Ω = *v*_0_ = *r*_0_^3^ or v_*_ = *v*_0_/γ with γ a lattice structure dependent constant. The difference between ⟨*u*^2^⟩ in a particular direction or one component of ***u*** (as usually obtained from diffraction data) differs from the spherically averaged ⟨***uu***^T^⟩, while the difference between Ω and v_*_ speaks for itself.

Recently Vopson et al.^47^ suggested that a ξ-value per group of the periodic systems is more appropriate for metals (groups 16–18 were not included). They noticed that, upon the general increase of *T*_mel_ with the atomic mass *m*, peaks were visible, which upon further analysis appeared to be correlated with the group number, except for groups 3, 13, and 14. All 12 remaining groups contained three elements, except groups 1 and 2, containing five elements, and group 7, containing two elements. For the remaining groups the ξ-values were estimated by the least-squares fit of *T*_mel_/θ_D_^2^*a* versus *m* with *a* the nearest-neighbor distance. The overall correlation can be described by *T*_mel,calc_ = *cT*_mel,exp_ with slope *c* = 0.972 and correlation coefficient *R*^2^ = 0.991. The resulting ξ-values are shown in Table 2. Estimating the uncertainty in *a* as 5% and that of θ_D_ as 10%, the overall uncertainty becomes 22% and, indeed, for five elements the deviation of *T*_mel,calc_ from *T*_mel,exp_ is larger than 20%.

**Table 2:** Lindemann Ratios at *T*_mel_ for Various Groups of the Periodic Table^47^

group	1	2	4	5	6	7
ξ-value	0.139	0.113	0.119	0.136	0.109	0.12
group	8	9	10	11	12	15
ξ-value	0.084	0.07	0.111	0.108	0.08	0.095

Long before that Gupta^48^ suggested that ξ is not only structure dependent but also interaction dependent. This resulted from his average ξ ≅ 0.118 for the set Ne (ξ = 0.145), Ar (ξ = 0.115), Kr (ξ = 0.113), and Ne (ξ = 0.099) and the average ξ ≅ 0.071 for the set Al, Cu, Au, Pb, and Ni, all individual values for the metals as calculated by Shapiro^49^ close to the average value. He calculated these values using quasi-harmonic lattice dynamics with a Buckingham potential including interactions up to the 12th neighbors and a zero-point contribution by the Debye model. The deviations for Ne and Xe are attributed to the large anharmonic contribution for the zero-point energy for Ne and a small contribution for Xe, respectively 30 and 3.2% of the cohesive energy.^50^ In view of the “Lindemann” assumptions, such deviations are not a surprise at all.

Much closer values of ξ for the set Ar, Ne, Ar, Kr, and Xe were obtained by Mohazzabi and Behroozi^51^ using the Einstein model in combination with an LJ potential with parameters derived from the Debye temperature θ_D_ and the depth of the potential from various sources. The values calculated as well as the results of several other early calculations are shown in Table 3. We also note that for a one-component plasma in three dimensions (with a Yukawa interaction) ξ is about 20% larger.

**Table 3:** Lindemann Ratios at *T*_mel_ for Ne, Ar, Kr, and Xe^a^

material	ref (51)	ref (52)	ref (53)	ref (54)	ref (55)	ref (56)	ref (57)
Ne	0.148	0.099	0.127	0.109	–	0.14	0.121
Ar	0.122	0.107	0.113	0.101	–	0.14	0.113
Kr	0.110	0.108	0.115	0.10	0.11	0.14	0.114
Xe	0.106	0.108	0.114	0.099	–	0.14	0.109

a Reference (51), Einstein model; ref (52), LJ potential for one-dimensional chain; ref (53), entropy data; ref (54), lattice dynamics; ref (55), MD simulations; ref (56), MC simulations; ref (57), Wallis formula.

The reformulation of the Lindemann rule by Gilvarry employs only harmonic terms in the potential energy (while anharmonic terms and, more importantly, bond breaking are involved). It also neglects the possible effect of lattice defects near the melting point and, in the usual treatment, restricts the discussion to monatomic solids. Moreover, melting is linked to individual atomic properties (while it is a cooperative process) and the solid alone (while it should be linked to both liquid and solid).

In an attempt to put Lindemann’s rule on a firm statistical-mechanical basis, Ross^58^ and Kuramoto^59^ argued that, when the melting transition is viewed at an atomistic level, we would always see the same picture when properly scaled, as clarified by Hoover and Ross.^27^ Postulating that for all points along the melting curve the solid always occupies the same fraction of configuration space, that is, scaling the configurational partition function *Q* by using the reduced coordinates ***x*** = ***r***/*V*^1/3^ with *V* the volume of the system and ***r*** the coordinates of the *N* molecules so that *Q* = *V*^*N*^Q*, we will have Q*(*T*_mel_,*V*_mel_) = *const*. As a general theory of liquids was (is) lacking, the LJD model was used, in which *Q* = *v*_f_^*N*^ exp[−*βΦ*(**0**)] with free volume *v*_f_ = ∫exp(−βΔΦ)d***r***, ΔΦ = Φ(***r***) – Φ(**0**), and β = 1/*kT*, leading to *v*_f_*(*T*_mel_,*V*_mel_) = *const*. Using for Φ(***r***) the LJ potential, good agreement with thermodynamic data is shown for Ar. Reducing ΔΦ to a harmonic oscillator potential, ΔΦ = 1/2*ar*^2^ = 1/2*aV*^2/3^*x*^2^ = 1/2*mω*^2^*V*^2/3^*x*^2^, the requirement *v*_f_* = *const.* leads right away to βΔΦ = *const*. or at melting to *T*_mel_ ∼ *mω*^2^*V*_mel_^2/3^/*k*, which is Lindemann’s rule. Obviously, this approach still does not incorporate the two-phase considerations and anharmonicity. However, arguments have been given as why such an approach might still work (section 4).

Chakravarty et al.^60^ discussed Lindemann-type measures to assess the S–L transition in a 343 particle LJ system. For this they used the inherent structures,^61,62^ i.e., the minima of *U*(***x***) corresponding to mechanically stable particle packings with the global minimum of *U*(***x***) being the perfectly ordered crystal lattice, where *U*(***x***) is the multidimensional potential energy function *U*(***x***) of the *N*-particle system with ***x*** the 3*N*-dimensional position vector. Any instantaneous configuration sampled from a suitable ensemble can be quenched to the corresponding inherent structure using a local steepest descent (SD) minimization. The set of instantaneous structures connected by SD mappings to the same minimum constitute the basin of the corresponding inherent structure. If the atomic positions in an instantaneous configuration and the corresponding inherent structure are denoted by the 3*N*-dimensional vectors ***x*** ≡ (***x***_1_, ..., ***x***_*N*_) and ***q*** ≡ (***q***_1_, ..., ***q***_*N*_), respectively, then the configurational return distance Δ of a particular configuration in the ensemble is given by Δ^2^ = (1/*N*)(***x*** – ***q***)^2^. If the deviation in the position of an atom *j* from its position in the corresponding inherent structure is denoted by the vector **δ**_*j*_ = ***x***_*j*_ – ***q***_*j*_, then the normalized single-particle return distance squared distribution is defined by ∏(**δ**^2^) using the **δ**_*j*_^2^ values of all atoms from a sampled set of *M* configurations. The means of the squared single-particle distribution ⟨δ^2^⟩ and configurational return distance distribution ⟨Δ^2^⟩ will coincide, i.e., ⟨δ^2^⟩ = ⟨Δ^2^⟩. To assess melting, the authors studied an *N* = 256 particle system with MC simulations using constant (*N*,*P*,*T*) conditions with periodic boundary conditions and employing a reduced and smoothed LJ potential ϕ_SLJ_.^63^

To measure the extent of local order, local bond orientational order parameters were used. The orientation of a bond vector ***r*** joining an atom with a neighbor lying within a cutoff distance *R*_c_, relative to a space-fixed reference frame, is denoted by the spherical polar angles θ(*r*) and ϕ(*r*). With each bond surrounding a given atom, a spherical harmonic *Y*_*lm*_[θ(*r*),ϕ(*r*)] is associated, and by summing over all the bonds connecting a given atom with its *N*_b_ nearest neighbors within a sphere of radius *R*_c_ = 1.25*r*_eq_ with *r*_eq_ the equilibrium pair separation, the quantity *q*_*lm*_ = *N*_b_^–1^∑_*j*_*Y*_*lm*_[θ(*r*_*j*_),ϕ(*r*_*j*_)] can be defined. A rotationally invariant local order parameter *q*_*l*_ = {[4π/(2*l* + 1)]∑_*m*_|*q*_*lm*_|^2^}^1/2^ with −l ≤ *m* ≤ l can then be constructed. It has been shown that *q*_6_ is large when particles sit in an icosahedral, FCC, or HCP environment.^64^ The authors showed that there is a strong negative correlation between δ^2^ and *q*_6_ in the solid phase; i.e., atoms with large deviations from lattice positions will also tend to be in locally disordered environments with low *q*_6_ values, while in the liquid phase there is no correlation between the single-particle return distance and the local order, the value of *q*_6_ essentially being constant at about 0.46. Taking this value as characteristic for the liquids, at a reduced temperature T* = 0.5 essentially none of the atoms can be regarded as being in a local environment that is sufficiently disordered to be classified as liquid-like. On the other hand, at T* = 0.76, which is approximately 10% greater than the melting temperature *T*_mel_* = 0.67, there is a significant fraction of atoms with *q*_6_ values less than 0.46. For solids with *q*_6_ ≅ 0.46, δ^2^ ≅ 0.06 or δ ≅ 0.24, which is considerably larger than ⟨δ^2^⟩^1/2^ = 0.16 at the reduced temperature T* = 0.76 used. Thus, a *q*_6_ value of 0.46 or a δ value of 0.24 can be taken as the threshold for local disorder for an atom in a solid and atoms with δ > 0.24 can be classified as liquid-like. We note, though, that a proper description of solids often needs *q*_6_ as well as *q*_4_.

In a follow-up, Chakraborty et al.^65^ discussed the use of a Landau-type Helmholtz energy expansion in combination with MC calculations and related the results to classical nucleation theory. As in regular sampling in simulations the transition state region is too infrequently sampled, umbrella sampling was applied. Moreover, rhombic dodecahedral boundary conditions were used since the near spherical shape of that cell reduces the artificial stabilization of the solid due to (regular) periodic boundary conditions. The simulations were done for reduced relative temperatures of T*/*T*_mel_* = 1.00, 1.05, 1.10, 1.17, and 1.20 at *P* = 0.67 bar for which *T*_mel_* = 0.780, using again 343 particles. For all temperatures the so-obtained Helmholtz curves were smooth. At T*/*T*_mel_* = 1.20 the Helmholtz energy barrier disappeared, indicating the limit of superheating and agreeing well with other results. As Δ_m_*H* varies only 3.5% over this range, the classical approximation for the chemical potential Δμ = Δ_m_*H*(*T* – *T*_mel_)/*T*_mel_ is quite reasonable. To be able to use nucleation theory, also the interface energy is required, which was estimated using the estimate of Davidchak and Laird,^66,67^ reading γ_SL_ = 0.617*kT*/σ_eff_ with σ_eff_ the Barker–Henderson estimate for the LJ system and σ_eff_ ≅ σ for the temperatures used. The critical nuclei varied from 20.7σ for T*/*T*_mel_* = 1.05 to 6.5σ (about 900 particles) for T*/*T*_mel_* = 1.20. The results suggested that the bond orientational coordinate *q*_6_ is an important internal coordinate driving the phase transition process. If it would be the only relevant parameter, one expects that *q*_6_ for liquid and solid has the same value at the same value of the committor function; i.e., the function associated with the *q*_6_ coordinate describing the probability that trajectories initiated from an *q*_6_ constrained simulation will terminate in the liquid phase. This, however, turned out not to be the case, but only for a *q*_6_ value larger than about 0.22. This suggests that some configurational properties other than the overall degree of crystalline order must play a critical role in determining the mechanism of melting. Finally, they note that the approximate size of critical nuclei predicted by classical nucleation theory are of the correct order of magnitude for the crystallization process but are comparable to or larger than the simulation cell size used in Landau approaches to study melting. This suggests that a classical nucleation theory pathway is unlikely to be important for melting, at least close to solid–liquid coexistence.

In an approach akin to that of Lindemann, Dunne et al.^68^ estimated the harmonic force constants *k*_f_ from the LJ potential, resulting in *k*_f_ = 72ε/*r*_e_^2^ with ε the well depth and *r*_e_ the equilibrium distance. Equating (1/2)*kT* with the harmonic potential so obtained yields, using the Lindemann assumption, *kT*_mel_ = *k*_f_*r*_e_^2^ξ^2^ or *T*_mel_ = 72*εξ*^2^/*k*. This resulted in a good correlation for Ne, Ar, Kr, Xe, N_2_, O_2_, CH_4_, and CF_4_ of *T*_mel_ with ε, and they sought to explain why this correlation worked well. From thermodynamics *T*_mel_ is given by *T*_mel_ = Δ_m_*H*/Δ_m_*S* = (Δ_m_*U* + *P*Δ*V*)/Δ_m_*S* ≅ Δ_m_*U*/Δ_m_*S*, where Δ_m_*H*, Δ_m_*U*, Δ_m_*S*, and Δ_m_*V* refer to enthalpy, energy, entropy, and volume changes at pressure *P*, and the last step can be made as Δ*V* during melting is relatively small. In a simple nearest-neighbor model Δ_m_*U* = (1/2)*zε*(1 – ρ) = (1/2)*zε*Δ*V*/*V*, with *z* the coordination number and ρ the relative density of the liquid with respect to the solid, and the last step can be made as Δ*V* is small. This leads to
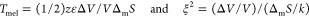
7where the last step is made by combining with *T*_mel_ = 72*εξ*^2^/*k*. Taking data for Ne, Ar, Kr, Xe, and N_2_ resulted in an average value ξ = 0.084, which is close to the value ξ = 0.089 obtained from the fit of *T*_mel_ versus ε.

As a next step these authors employed a one-dimensional model with periodic boundary conditions, where it is supposed that there are three types of species, namely (1) vacancies, (2) ordered cluster of *m* atoms using *r* sites, and (3) vibrationally disordered clusters, also containing *m* atoms but occupying *r* + 1 sites. The constant pressure partition function Ω(*N*,*T*,*P*) was employed to calculate the difference in Helmholtz energy Δ*F* = Δ*U* – *T*Δ*S* between the two types of clusters of atoms. They assume that Δ*F* becoming negative, corresponding to when the disordered clusters dominate over the ordered clusters, indicates melting as a 2D or 3D model along these lines would be prohibitively difficult. The value *r* = 6 was chosen, a value being representative for the volume change upon melting of rare gas solids, while the partition function was evaluated using the maximum term method. This led to the equilibrium value *V* = −*kT* ∂Ω/∂*P*. Further using the parameter values Δ*U* = 0.18 eV and Δ*S* = 25.72*k* reproduces the melting line for Ar below about 5000 bar quite well, and *r* ≅ 15–20 leads to values for Δ*U* and Δ*S* roughly matching the data for Ne, Ar, Kr, Xe, and N_2_. Finally estimating an interaction energy for atoms as −0.11 eV led to a *P*–*V* curve with a clear step in volume at relative volume *V*_rel_ = 1 and *T*_mel_ = 84 K at 1 bar. Although the model is one-dimensional and cannot reproduce the true discontinuity characteristic of a 3D solid, it mimics experimental *P*–*V* curves with a volume change roughly corresponding to values obtained for ξ.

Batsanov^69^ also used an approach akin to the Lindemann approach. To have consistent data, the author first calculated ξ conventionally for 48 metals and the five noble gases He, Ne, Ar, Kr, and Xe from *ξ*_*θ*_ = *c*_1_(*T*_mel_/*m*)^1/2^/θ_D_*r* with θ_D_ the Debye temperature at *T*_mel_, *m* and *r* the atomic mass and radius, and *c*_1_ = 12.06 if *r* is in angstroms and *T*_mel_ in kelvin. The mean values quoted were for the metals *ξ*_*θ*_ = 0.137 ± 0.037 and for the noble gases *ξ*_*θ*_ = 0.108 ± 0.008, where “±” indicates the sample standard deviation.^70^ Thereafter it was argued that, because of the use of different definitions for the various parameters, it appears natural to calculate ξ from thermodynamic data. To that purpose *H*_m_ = Δ*H*_*T*_ + Δ_m_*H* ≡ [*H*(*T*_mel_) – *H*(0)] + Δ_m_*H*, where Δ*H*_*T*_ is the enthalpy to heat the material from 0 to *T*_mel_ and Δ_m_*H* is the melting enthalpy, was used and equated to the energy of an harmonic oscillator as given by *E* = 1/2*f*_m_(*ξr*_m_)^2^ with *f*_m_ the force constant at *T*_mel_. The latter quantity was estimated from the previously derived expression for the force constant at 0 K given by *f*_0_ = 0.009*K*_0_*V*_0_/*zr*_0_^2^^71,72^ with *z* the coordination number and *K*_0_ the bulk modulus at *T* = 0, and the “obvious” translation to *f*_m_ = *f*_0_*K*_m_*r*_m_/*K*_0_*r*_0_, where the subscripts indicate the temperature. This resulted in *ξ*_*H*_ = (*H*_m_/*f*_m_)^1/2^/*C*_2_*r* with *H*_m_ in kJ mol^–1^, *r* in Å, *f*_m_ in mdyn Å^–1^, and *C*_2_ = 54.87, a constant. The resulting average values are *ξ*_*H*_ = 0.150 ± 0.023^70^ for 35 metals and *ξ*_*H*_ = 0.127 ± 0.008 for the noble gases. Clearly, the values so obtained are slightly larger than those conventionally calculated. For both types of calculations reported, the author did not observe the previously reported anomalously high value for He, for which reasons were given, and concluded that the Lindemann rule cannot be used to explain the special features of He solidification.

In an approach taking both solid and liquid states as well as anharmonicity into account, Lawson,^73^ examining 74 distinct elements,^74^ also rationalized the Lindemann rule. The starting point is the entropy difference Δ*S* = *S*_L_ – *S*_S_ between liquid (L) and solid (S). For the liquid with molecules with mass *m*, a Sackur–Tetrode-like expression *S*_ST_ as derived from *F* = −*kT* ln *Z* with *Z* = *z*^*N*^ instead of *Z* = *z*^*N*^/*N*! and the volume taken as the atomic volume Ω = *V*_m_/*N*_A_, was used together with a correlation term *S*_cor_ (for the perfect gas *S*_cor_ = −*k* ln(1/*N*!). Hence, the entropy *S*_L_ per molecule in the liquid phase is given by
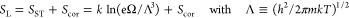
8

For the solid the Wallace expression^75,76^*S* = 3*k* ln(e*T*/θ_0_) with θ_0_ the characteristic entropy temperature^8^ was used. The parameter θ_0_ corresponds with the zeroth moment or, equivalently, the geometric mean of the phonon frequencies, *kθ*_0_ = ℏϖ. Since θ_0_ values are hard to get, Lawson inserted, based on Debye theory and backed up by a strong correlation, the expression θ_0_ = e^–1/3^θ_D_ with θ_D_ the Debye temperature as obtained from either low-temperature heat capacity or elastic behavior data.^8^ For convenience of getting data, an empirical relation, namely 1.3*kθ*_D_ = *kθ*_ela_ = ℏ(*K*_*T*_*Ω*/*m*)^1/2^(6π^2^/Ω)^1/3^ with θ_ela_ the Debye temperature as derived from room temperature elastic data, was introduced. Note that, using the bulk modulus *K*_*T*_, the expression for θ_ela_ ignores the shear modes. They are left out since data for these modes are more difficult to obtain, particularly at high temperature (for the same reason, the temperature dependence of *K*_*T*_ and Ω was neglected). Hence, the entropy per molecule for the solid is given by
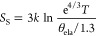
9

Finally, introducing the effect of anharmonicity via the thermodynamic relation *C*_*P*_ – *C*_*V*_ = α^2^*K*_*T*_*ΩT*, the corresponding high temperature entropy term 9γ^2^*k*^2^*T*/*K*_*T*_*Ω* was added with γ = *αK*_*T*_*Ω*/*C*_*V*_ the thermodynamic Grüneisen parameter and α the (volume) thermal expansivity. Taken together this leads, via Δ*S* – *S*_cor_ = *S*_ST_ – *S*_S_ – *S*_anh_, to
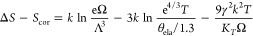
10

**Figure 5:**
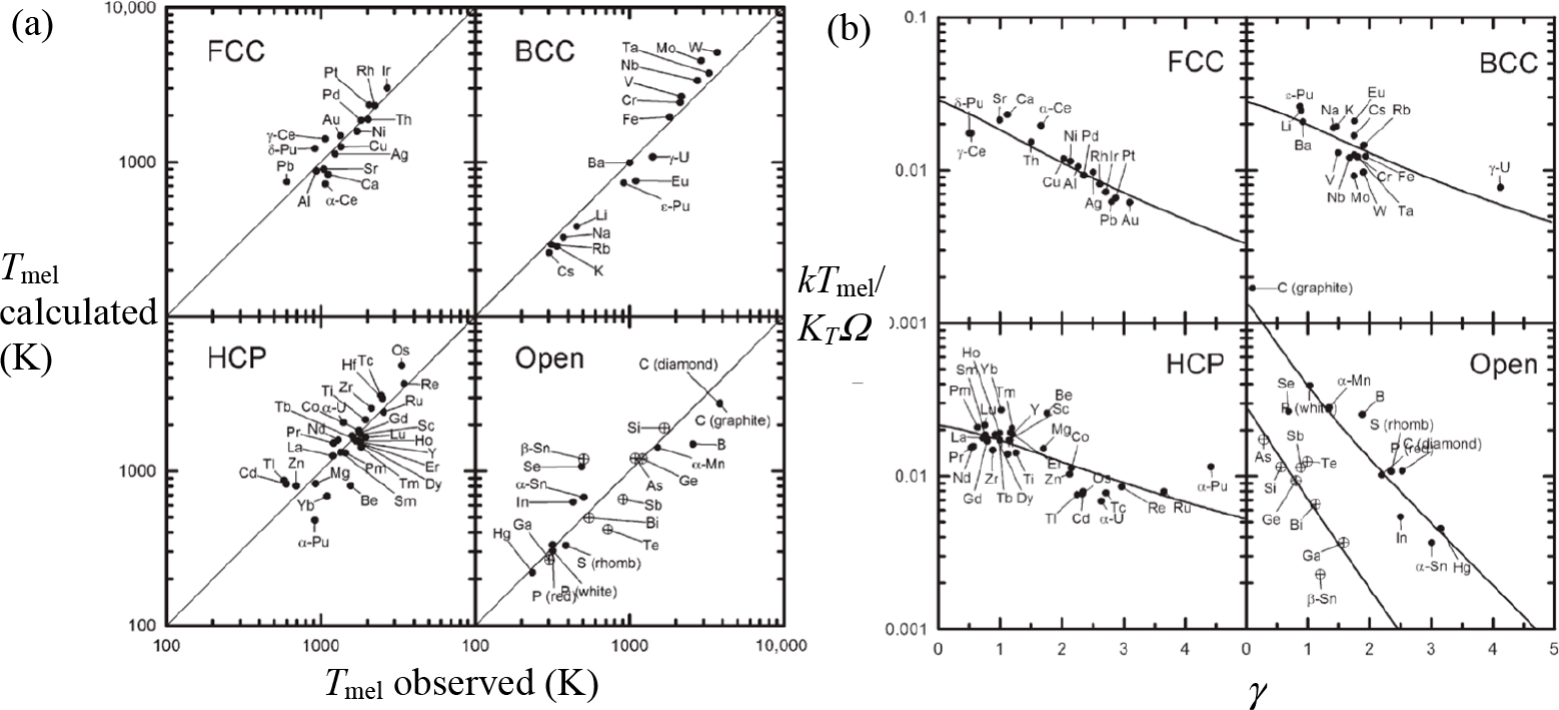
Melting characteristics for elements. (a) Melting temperature *T*_mel_ calculated versus *T*_mel_ observed. (b) The ratio *kT*_mel_/*K*_*T*_*Ω* versus Grüneisen parameter γ. Actually, Lawson divided the open structures into two categories. Hence, the panel labeled “open” shows two correlations. Reproduced with permission from ref (73). Copyright 2009 Taylor & Francis.

Lawson divided the structures examined in FCC, BCC, HCP, and more complex, “open” structures. Fitting −*S*_cor_ = *S*_ST_ – *S*_S_ – *S*_anh_ – Δ*S* by −*S*_cor_ = *a* + *bγ*, given the experimental data for Δ*S*, shows that a strong correlation between (Δ*S* – *S*_cor_) and γ is obtained. As also a strong correlation between Δ*S* and γ is observed, *S*_cor_ can be estimated. Using these fits, *T*_mel_ can be calculated from eq 10. Figure 5a shows the comparison with the experimental data, indicating good agreement in view of the approximations made. As for many elements *S*_anh_ ≪ *S*_cor_, generally *T*_mel_ is determined by a balance between *S*_S_, the Debye entropy for the solid, and *S*_cor_, the correlation entropy of the liquid. The dimensionless ratio *kT*_mel_/*K*_*T*_*Ω* plotted versus γ also shows a good correlation (Figure 5b).

The Gilvarry ratio ξ was calculated as ξ = ⟨*u*^2^⟩^1/2^/2r_*_ (note the “2”) with ⟨*u*^2^⟩ = 3ℏ^2^*T*_mel_/*mkθ*_ela_^2^ and r_*_ = (3Ω/4π)^1/3^, leading to ξ = (0.513*kT*_mel_/*K*_*T*_*Ω*)^1/2^. Inserting numbers, one obtains approximately ξ/2r_*_ ≅ 0.1–0.04γ or ξ/2r_*_ ≅ 0.08 using γ ≅ 2, typical for FCC, BCC, and HCP structures. However, this estimate seems to fail for the open structures. As the considerations are essentially based on Debye’s model, this might have been expected. We also note that, as atomic size measure *r*, the cube root of the atomic volume Ω was used, although for transition metals with a variation in structures the translation from Ω^1/3^ to *r* codepends on the structure. In conclusion, Lawson rationalized Lindemann’s rule and showed that it is well obeyed for simple structures but much less well for less symmetric structures.

### 5.2 Other Rationalizations Using Vibrational Instability

Although the basic idea of Lindemann’s rule is the critical value for the displacement, it can be rationalized in other ways, and one such approach is due to Enderby and March.^77^ For closed-packed metals the authors showed that from the correlation for the vacancy energy *E*_vac_ = *c*_1_*ZE*_F_ (with *c*_1_ ≅ 1/6, *Z* is the valency, and *E*_F_ = 1/2*m*_ele_*v*_F_^2^ is the Fermi energy) and the velocity of sound *v*_s_ = (*m*_ele_*Z*/3*m*_ion_)^1/2^*v*_F_ (with *m*_ele_ the electron mass, *m*_ion_ the ion mass, and *v*_F_ the Fermi velocity), combined with the Debye temperature expression θ_D_ = (*h*/*k*)(3/4πΩ)^1/3^*v*_s_ (with Ω the atomic volume), the relation θ_D_ = (*h*/*k*)(3/4πΩ)^1/3^(2*E*_vac_/3*c*_1_*m*_ion_)^1/2^ follows. Further, they considered that if the energy *U*, required to take an atom from an ordered to a disordered site, is similarly like the vacancy formation energy *E*_vac_ assumed to be proportional to *ZE*_F_, using the Bragg–Williams solution for the order–disorder problem to estimate the melting point leads to *kT*_mel_ ≅ (1/4)*U*_0_ = *c*_2_*ZE*_F_. Here *U* is approximated by *U* = *U*_0_η with η the degree of order, defined as the ratio of the number of atoms on lattice sites to the total number of atoms, and *U*_0_ an energy characteristic of a perfectly ordered crystal. Combining these expressions, the result is the Lindemann expression θ_D_ = *c*(*T*_mel_/*m*_ion_*V*_m_^2/3^)^1/2^ with *V*_m_ the molar volume and *c* = (*h*/*k*)(3/4π)^1/3^(2*k*/3*c*_2_)^1/2^*N*_A_^5/6^ with *N*_A_ Avogadro’s constant. The proportionality constant *c*_2_ = (1/4)*c*_1_ was also estimated from *kT*_mel_/*ZE*_F_ extrapolated to *Z* = 0, resulting in *c*_2_ ≅ 1/30 and *c* ≅ 100 cm g^1/2^ K^–1/2^, to be compared with the quoted experimental value of ∼120 cm g^1/2^ K^–1/2^, while the actual range is more like 130–160 cm g^1/2^ K^–1/2^.

Another approach is by Stacey and Irvine,^78^ who argued that the Lindemann approach can be derived from the well-known empirical relation *αT*_mel_ ≅ *const*., where α is the thermal expansion coefficient. They refer to the Lindemann relation in its differential form as *T*_mel_^–1^(d*T*_mel_/d*P*) = 2(γ – 1/3)/*K* with the thermal Grüneisen parameter γ = *αK*/*ρC*. Here *K* is the (adiabatic or isothermal) bulk modulus, ρ is the density and *C* is the heat capacity (at constant volume or constant pressure). Their approach starts with the expression (∂*P*/d*T*)_*V*_ = *αK* = *γρC*_*V*_, which in its integrated form Δ*P* = ∫*γρC*_*V*_ d*T* ≅ *γρ*Δ*E* is the Mie–Grüneisen EoS. Here Δ*E* is the thermal energy applied to a mass *m* at constant ρ which causes the increase in pressure Δ*P*. Invoking the equipartition theorem in the form Δ*E* = Δ*E*_kin_*+* Δ*E*_pot_ = 2*E*_pot_, where Δ*E*_kin_ is the kinetic energy part and Δ*E*_pot_ is the potential energy part, Δ*P* = 2*γρ*Δ*E*_pot_/*m* results. Making a similar assumption as Lindemann, the thermal energy *mL* for mass *m*, where *L* is the melting enthalpy per unit mass, appears fully as potential energy at the melting temperature for melting at constant volume, which yields Δ*P* = 2*γρL*. Using Δ*P* = *K*(Δ*V*/*V*) = *ρK*Δ*V*, the final result is
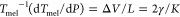
11which resembles closely the differential Lindemann relation. The relation was made more precise by considering a closed cycle connecting liquidus and solidus, from which detailed expressions for *L*, Δ*P*, and *K* along the melting curve and *T*_mel_ in terms of γ and *C*_*V*_ were derived. The authors emphasize (1) the role of anharmonicity as expressed by γ for regular thermal expansion, (2) that Lindemann’s relation is restricted to materials that do not undergo major changes in coordination on melting (as otherwise the regular bond length changes are not represented by γ), and (3) that the starting relation Δ*P* ≅ *γρ*Δ*E* is essentially exact for a temperature not low with respect to θ_D_; apart from that γ is taken to be temperature independent.

Still another rationalization was given by Stillinger and Weber^79^ based on simulations for the Gaussian core model. In its simplest form this model contains only exponential repulsion resulting in that the stable crystal form at *T* = 0 for the reduced density ρ* < 0.179407 is FCC, while for ρ* > 0.179767 it is BCC. To place the Gaussian core model in context, it was deemed useful to interpret the Gaussian potential at a given distance *r* in terms of an effective inverse power potential. By matching the logarithmic derivatives for the two functions, it appears that the exponent of the inverse-power form must be n*(*r*) = 2*r*^2^, which for the nearest-neighbor distance for the BCC crystal at density ρ* results in n*(*a*) = 6(4ρ*)^2/3^ or 6.96 at ρ* = 0.2. As this represents a much softer potential than usually assumed for pair potentials, the authors suggest that the model might be useful for studying matter under compression. Indeed, an approximate correspondence can be established between the thermodynamic states of the Gaussian core model and those of Ar for which ρ* = 0.2 corresponds to about 5.5 Mbar. For MD simulations containing 432 particles, using periodic boundary conditions and starting with the BCC structure, melting (and freezing) was monitored by the pressure, mean potential energy, pair-correlation function, and self-diffusion constant using averages over about 4000 time steps. As hysteresis for such a system is inevitable, *T*_mel_ was estimated as the center of the hysteresis loop, resulting for ρ* = 0.2 in *T*_mel_* = 8.1 × 10^–3^ and entropy change Δ*S*/*Nk* = 0.847. Mean square displacements were calculated for the last half of simulation runs up to t* = 200. Because the positions of any given particle at widely separated times are uncorrelated, ⟨(Δ*r*)^2^⟩ = 2⟨(*u*)^2^⟩, with *u* the displacement from the stable lattice position, which led to the ratio *f* = ⟨(*u*)^2^⟩^1/2^/*a* = 0.160 ± 0.005, substantially above the value *f* = 0.113 given by Shapiro^49^ for BCC alkali metals from lattice dynamics calculations. The latter value is to be compared with *f* = 0.071 calculated for the FCC metals Al, Cu, Ag, and Au and reasonably well agrees with the results for Al and Cu determined by Martin and O’Connor^80^ by Bragg diffraction of Mössbauer X-rays. The Lindemann–Gilvarry ratio is thus structure dependent, an in the meantime well-established conclusion.

**Figure 6:**
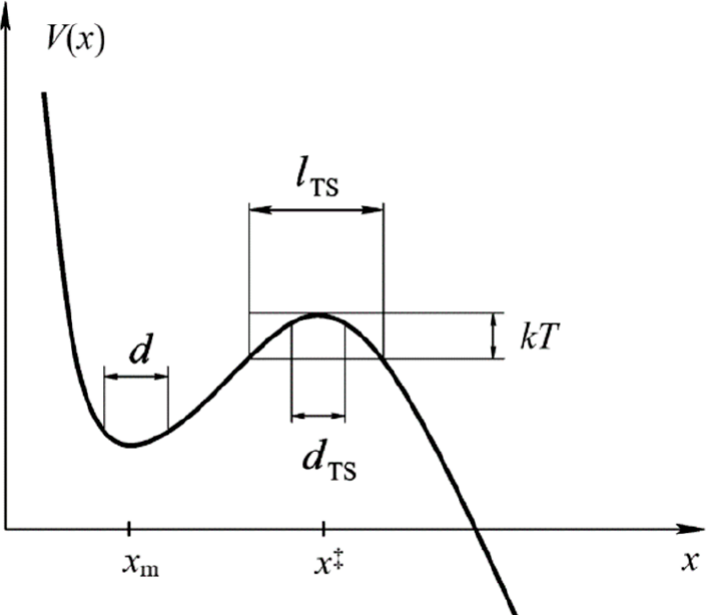
Generic schematic of an escape Helmholtz energy profile, where the size of the transition state *l*_TS_ delineates the vicinity of the saddle point within the thermal energy from the top and *d* and *d*_TS_ are defined in the text. Reproduced from ref (81). Copyright 2006 American Chemical Society.

In an attempt to create a dynamical image, Lubchenko^81^ derived a dynamical Lindemann criterion. The author considered that a proper criterion, “presumably”, should compare some property of both phases and should proceed with reference to processes at the liquid/solid interface. The interface is considered as an “interphase” with a density changing from the solid to the liquid density, but also having a heterogeneity in relaxation times, i.e., the lifetimes of long-living local structures, interpolating between those in the liquid and the solid. The distinction between liquid and solid was made, as usual, via symmetry, and the focus was specifically on the time scale on which the symmetry is broken/restored: For a crystal one can label the molecules based solely on each one being located within a particular, well-defined cell, while for a fluid such labeling is impossible. For the liquid the corresponding translation symmetry is maintained by material transport with τ_0_, the time it takes a molecule to diffuse a distance defining the volumetric density of the liquid. For a compact specific cell in space, whose volume is equal to the volume per molecule in the liquid, the time τ_0_ is significantly longer than the time scale of density fluctuations. A second identical molecule will have visited the chosen cell within time τ_0_ upon the exit of the first molecule, thereby eliminating the possibility to label a molecule by its spatial location. If 1/*a*^3^ ≡ *n* is the molecular concentration in the fluid, where *a* is the average volumetric molecular spacing, the typical collisional or autocorrelation time defining the density fluctuation time scale is τ_auto_ = *m*/ζ, where ζ = 6π*aη* is the friction coefficient, η is the viscosity and *m* is mass of the molecule. Because the time τ_0_ it takes to diffuse a distance *a* is approximately *a*^2^/6*D* with *D* the diffusion constant, and ζ is given by the Einstein relation *D* = *kT*/ζ, the result is
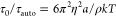
12where ρ is the density of the liquid. The author calculated for this ratio for Co and Na values of 1.3 × 10^3^ and 2.1 × 10^3^, respectively, indicating that it takes about 1000 molecular collisions per molecular volume to establish local thermal equilibrium in a liquid. If, for a region of space occupied by a solid and its melt, at some temperature *T* just above the lowest temperature *T*_sm_ where surface melting is possible, a molecule in that region fails to move a distance *a* in the time τ_0_, it must be regarded as part of the solid. The boundary of any spatially closed set of such molecules is therefore defined as the solid–liquid interface. The inability of a molecule to move the distance *a* in time τ_0_ is equivalent to residing the molecule in a metastable Helmholtz energy minimum; or in other words, a molecule is part of the solid if its escape time τ_esc_ from its current neighborhood exceeds τ_0_. In quasi-equilibrium τ_esc_ = τ_0_, and this expression can be considered as the dynamical definition of a solid–liquid interface. The escape time τ_esc_ can be estimated from standard transition state theory. Because of the frequent collisions, the escape mode is strongly overdamped, so the mean free path *l*_mfp_ is much smaller than the size of the transition state region *l*_TS_ (Figure 6). Hence, the escape rate τ_esc_^–1^ is corrected by a transmission factor κ ≅ 2*l*_mfp_/*l*_TS_ = 2τ_auto_*v*_th_/*l*_TS_ as appropriate in the overdamped Kramers limit. Therefore
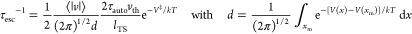
13the “size” of the metastable well. Further, *v*_th_ = (3*kT*/*m*)^1/2^ is the thermal velocity, the factor (2π)^−1/2^ was added so that *d* = ⟨(Δ*x*)^2^⟩^1/2^ if the potential is strictly harmonic at the minimum *x*_m_, while *x*_m_ in the integration limit indicates integration over the size of well. The quantity ⟨|*v*|⟩ = *v*_th_/(2/3π)^1/2^ is the thermally averaged particle speed that enters the expression for the molecular flux at the barrier top and *V*^‡^ ≡ *V*(*x*^‡^) – *V*(*x*_m_) is the barrier height. Because an escape event will have occurred if the displacement of a particle just exceeds the typical thermal vibrational amplitude at the interface, *V*^‡^ = *kT*. Finally, *d*_TS_ was defined as *d*_TS_ = 8^–1/2^*l*_TD_, where the numerical factor is chosen so that if the barrier is parabolic at the top, then *d*_TS_ = ⟨(Δ*x*)^2^⟩^1/2^ in the inverted potential at the saddle point (Figure 6). Using (1/2)*m*(ω*l_TS_)^2^ = *kT* with ω* the underbarrier frequency and recalling that τ_0_ = *a*^2^/6*D* = *a*^2^*m*/6*kTτ*_auto_ = *a*^2^/2*v*_th_2τ_auto_, the final result from τ_esc_ = τ_0_ is
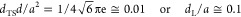
14

The last step is made by using for the amplitude of the reversible motion in the molecular metastable minimum at the liquid–solid interface *d*_L_ ≅ *d* ≅ *d*_TS_, because of the approximate character of the analysis. The author further discussed several aspects of surface melting consistent with his model and the need for “structural” reconfigurations, by entropy arguments ruling out vacancies. The main point is here that a purely kinematic criterion of melting in terms of the ratio of length scales characterizing molecular motions in the interface region, akin to the Lindemann criterion, is obtained.

As indicated at the start of this section, Lindemann’s concept (*T*_mel_ proportional to some characteristic frequency) and Gilvarry’s concept (*T*_mel_ occurs at a certain value of ξ) are generally taken together when referring to Lindemann’s rule. Luo et al.^82^ clearly distinguished between the two and used MD simulations with an LJ potential at constant *N*, *P*, and *T* to differentiate between the two. These authors used the density of (vibrational) states (DoS) to assess various definitions for the mean frequency and showed that the Lindemann assumption is well obeyed if for the characteristic frequency ⟨ω^–2^⟩^–1/2^ is chosen, while the Gilvarry assumption is not of sufficient accuracy for the LJ system. The Gilvarry ratio ξ shows a non-negligible pressure dependence, so caution should be exerted in applying Lindemann’s rule to predict high-pressure data. Moreover, they indicated that discussions on the comparison between various results is clouded with using different definitions for ξ by different authors, as already indicated before, namely for one component, labeled here as ξ_1_, or the average spherical average, here labeled by ξ. They quote as examples the uses of ξ by Hansen,^56^ Stillinger and Weber,^79^ and Hoover et al.^83^ and ξ_1_ by Gilvarry^44^ and Martin and O’Connor.^80^

To detect local differences experimentally, one obviously requires a technique that probes locally. X-ray absorption fine structure (XAFS) is such a technique, probing essentially only the first coordination shell as represented by the first peak in the pair correlation function *g*(*r*). Using an improved background correction procedure for XAFS experiments, Stern et al.^84^ were able to determine for Pb the moments of the first peak in *g*(*r*) accurately. Upon melting the second moment did not change much, while the first moment, essentially the coordination number, decreases and the third moment increases upon melting, thereby indicating an increase in asymmetry. The behavior could be described by a one-dimensional anharmonic oscillator with potential (1/2)*αx*^2^ + *βx*^3^ + *γx*^4^ with *x* representing the displacement about the equilibrium position and which for Pb yielded α = 0.87 ± 0.1 eV Å^2^, β = −0.28 ± 0.09 eV Å^3^, and γ = 0.15 ± 0.1 eV Å^4^. To lowest order, the second moment is linear in *T*, from which a fit to the experimental data gave the Einstein temperature θ_E_ = 66 K, identical or close to the value calculated from θ_E_ = 3/4θ_D_^28,85^ using θ_D_ = 88 K^9,28^ or θ_D_ = 96 K.^10^ The decrease in (apparent) coordination number was proportional to the fraction of the time the atom is diffusing and is pertinent to the interpretation of premelting phenomena around impurities. It was observed for Hg impurities (concentration not given) that above 400 K, well below *T*_mel_ = 601 K, the apparent coordination number starts decreasing already significantly while the second moment saturates, consistent with a microscopic liquid region forming around the impurities. The authors conclude that it is striking that the data are consistent with the probability distribution as calculated from a simple one-dimensional anharmonic oscillator with small anharmonicity, but also that this anharmonic potential could be determined because of the use of a new method of background subtraction in the XAFS spectra which allowed the determination of the signal in regions within the edge of the pair correlation peak which were traditionally thought to be inaccessible.

More generally, since all versions of Lindemann’s rule contains many approximations, one can hope at best only for approximate agreement with experiment, and this has been amply shown by various authors. Nevertheless, using materials with similar structures, a reasonably constant ξ value has been shown to exist. The use of the Lindemann rule (and some other criteria) has been further tested by Saija et al.,^86^ who conclude, using the melting line in phase diagrams resulting from exp-6, inverse-power-law, and Gaussian potentials, that one-phase criteria give, on the whole, reliable estimates of freezing/melting points, with agreement ordinarily being better for an FCC solid than for a BCC crystal. Note, however, that some authors state “good” agreement for complex solids^87^ when appropriate parameters are taken, while others^88^ deny such agreement. Obviously, considering the Lindemann rule as a scaling rule (or as an example of a principle of corresponding states^89^), this depends on which of the aspects not dealt with remain constant over a series of compounds considered. Hoffmann^90^ goes as far as saying that estimating *T*_mel_ from *mθ*_D_^2^*a*^3/2^, or from θ_D_ only for that matter,^91^ is not convincing and for practical purposes it is insignificant, although estimating θ_D_ from *T*_mel_ is not so bad. The reason is clear: while estimating θ_D_ from *T*_mel_ requires the square root of *T*_mel_, thereby damping differences, estimating *T*_mel_ from θ_D_ requires the square of θ_D_, thereby enhancing differences. Nevertheless, in the absence of any experimental melting information, Lindemann’s rule may be the best bet.

### 5.3 Lattice Instability

Another approach, now largely abandoned, is based on the (in)stability of solids, as originally proposed by Herzfeld and Goeppert-Mayer.^92^ Increasing the temperature of a solid will reduce the elastic constants, and it was postulated by Born^93,94^ that at melting the shear elastic constant μ = *C*_44_ would vanish. He calculated an explicit expression for the Gibbs energy of a BCC crystal using (approximate) quasi-harmonic lattice dynamics and determined the variation of the elastic moduli with *T*/θ_D_. Moreover, he derived the stability conditions for cubic crystals at zero pressure. By adding external loading,^95^ in the hydrostatic case by adding −*PI* with *P* the pressure and *I* the unit tensor, the stability conditions are
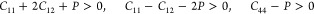
15

With *P* = 0 Born’s theory also leads to a Lindemann-like expression ν_D_ ≅ *c*(*T*_mel_/*MV*_m_^2/3^)^1/2^ with *c* ≅ 1.62 × 10^12^ in cgs units. An obvious drawback is that the theory is a single-phase theory which contains no distinct description of the melt, while both phases must be involved, and thus fails to account for the discontinuous, first-order character of melting. Because it is an essentially homogeneous theory, it does not explain the occurrence of superheating, the metastability of the melt, and the heterogeneous nucleation and growth features of the melting process. Observations show that none of the shear moduli is zero or near zero in the solid state at the melting point.^96^

However, Ida^97^ considered that a lattice instability may also be caused by the combined effect of vibrational and anharmonic bond length expansion *a* – *a*_0_, with *a* the bond length at temperature *T* and *a*_0_ its value at *T* = 0. He argued that for longitudinal phonons there is no bond length expansion but that for transverse phonons bond lengths may be equal to or longer, but never shorter, than the equilibrium distance along the propagation direction at any time. This contribution he called the vibrational contribution *Q*. Using the Debye model for monatomic crystals, he calculated *Q* = *kT*(*c*_1_^–2^ + *c*_2_^–2^)/15*m*, where *c*_1_ and *c*_2_ are the longitudinal and transverse wave velocities, respectively, and *m* is the mass of the atoms. For the anharmonic contribution he expanded the logarithms of the velocities to first order in the lattice expansion, resulting in ln *c*_*j*_ = ln *c*_*j*0_ – *b*[(*a* – *a*_0_)/*a*_0_ + *Q*] or, equivalently, *c*_*j*_ = *c*_*j*0_ exp{−*b*[[(*a* – *a*_0_)/*a*_0_ + *Q*]}, and obtained
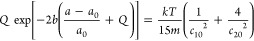
16

The function *Q* exp(−2*bQ*) has a maximum 1/2e*b* at *Q* = 1/2*b*, so eq 16 can only be satisfied below a critical temperature *T*_c__ri_ corresponding with that maximum, which is interpreted as the melting temperature *T*_mel_. To obtain *T*_mel_, an explicit expression for *a* – *a*_0_ is required, which is obtained from the Helmholtz energy in the harmonic approximation *F* = *U*_0_ + ∑_*j*_*ε*_*j*_ = *U*_0_ + ∑_*j*_ℏω_*j*_/[exp(βℏω_*j*_) – 1] ≡ *U*_0_ + *F*_th_, where *U*_0_ is the internal energy at *T* = 0, *ε*_*j*_ and *ω*_*j*_ are the energy and frequency of mode *j*, respectively, and β = 1/*kT*. From *F*, the Mie–Grüneisen EoS can be derived given by
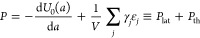
17where the mode Grüneisen parameter *γ*_*j*_ = −∂ln*ω*_*j*_/∂ln*V* in the Debye approximation is independent of the mode *j* and given by γ = (1/3)*ba*[1 + *a*_0_(∂*Q*/∂*a*)_*T*_/*a*_0_. This results in
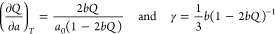
18by approximating *a* by *a*_0_. Substituting ∂*U*_0_/∂*a* = 9*NK*_*T*_(*a* – *a*_0_), with *N* the number of atoms, and γ in eq 17 results in
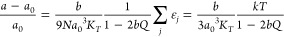
19where in the last step the high-temperature approximation *ε*_*j*_ = *kT* is used. Combining eqs 16 and 19 and using the abbreviations *q* = 2*bQ* and τ = 2*kb*^2^*T*/3*a*_0_^3^*K*_*T*_ results in *q* exp[−τ/(1 – *q*) – *q*] = *zτ* with *z* = *a*_0_^3^*K*_*T*_[1/*c*_10_^2^ + 4/*c*_20_^2^]/5*mb*. This all results after some algebra in
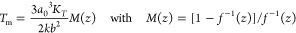
20where *f*(*q*) = [*q*^2^/(1 – *q*)^3^] exp[−*q*^–1^(1 – *q*)^2^ – *q*] with *q* = 2*bQ*. Numerical values for the function *M*(*z*) have been given by Ida.^97^ Furthermore, introducing the usual expressions for *c*_1_ and *c*_2_(9) results in another expression for *z* given by *z* = (1 + ν)(9 – 10ν)/15*b*(1 – ν)(1 – 2ν) where ν is Poisson’s ratio. Finally, from eq 19 he obtained α = *V*^–1^(∂*V*/∂*T*)_*P*_ = *kb*/*a*_0_^3^*K*_*T*_ so that *αT*_mel_ = 3*M*(*z*)/2*b*. Ida calculated the melting points for 10 metals, which resulted for a linear fit (not given in the paper) *T*_mel,calc_ = *cT*_mel,exp_ with correlation coefficient *R*^2^ = 0.884 and slope *c* = 1.744 and as the largest deviation the value for Mo, indicating a serious overestimate of the melting temperatures. The correlation coefficient increased to *R*^2^ = 0.966 with *c* = 1.069 upon deleting the two most deviating values of Mo and Ta, still an overestimate. Similarly, for 13 binary salts the fit resulted in *R*^2^ = 0.984 with *c* = 1.159. The author called the results fairly good in view of the approximations made.

More recently, Digilov and Abramovich^98^ used a rather similar but more detailed approach, however, without referring to Ida. They considered the temperature variation of the bulk modulus *K*_*T*_ = −*V*(∂*P*/∂*V*)_*T*_ and its volume derivative the Anderson–Grüneisen parameter δ = −(∂ln*K*_*T*_/∂ln*V*)_*P*_, thereby also predicting a thermoelastic instability. Assuming that δ = δ_0_*V*/*V*_0_, where δ_0_ and *V*_0_ are the Anderson–Grüneisen parameter and the molar volume at *T* = 0, respectively, results after integration in *V* = *V*_0_ – (*V*_0_/δ_0_) ln(*K*_*T*_/*K*_0_) or *K*_*T*_ = *K*_0_ exp[(*V* – *V*_0_)δ_0_/*V*_0_. From *V* = (∂*G*/∂*P*)_*T*_ and *G*(*P*,*T*) = *G*_0_(*P*) + *kT* ∑_*j*_ ln[1 – exp(−*βhν*_*j*_)], where *G*_0_(*P*) is the part of Gibbs energy *G* at *T* = 0 and *ν*_*j*_ is the frequency of mode *j*, they obtained
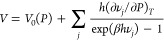
21with, as usual, β = 1/*kT*. Writing (∂*ν*_*j*_/∂*P*)_*T*_ = (∂*ν*_*j*_/∂*V*)_*T*_(∂*V*/∂*P*)_*T*_ = *γ*_*j*_*ν*_*j*_/*K*_*T*_, with as before *γ*_*j*_ the mode Grüneisen parameter and *ε*_*j*_ the mode energy, the result is
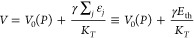
22where the averaging over all modes γ ≡ ∑_*j*_*γ*_*j*_*ε*_*j*_/∑_*j*_*ε*_*j*_ is used. This leads to *K*_*T*_/*K*_0_ = exp[−*γδE*_th_/*VK*_*T*_] or, using the Lambert function *W*, given by *x* = *W*(*x*) exp[*W*(*x*)], to *K*_*T*_/*K*_0_ = −*γδE*_th_/*VK*_0_/*W*(−*γδE*_th_/*VK*_0_) = exp[*W*(−*γδE*_th_/*VK*_0_)]. As *γF*_th_/*V* = *P*_th_, this can also be written as
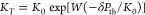
23

Because *W*(*x*) has no real roots for *x* < −e^–1^, the constraint *P*_th_ ≤ *K*_0_/eδ follows. This implies that, at the temperature where this condition is met, a thermoelastic instability occurs, which is identified as *T*_mel_. The thermal part *F*_th_ is given in Debye theory by *F*_th_ = 3*Rθ*_D_*D*(θ_D_/*T*), where *R* is the gas constant, θ_D_ is the Debye temperature, and *D*(θ_D_/*T*) is the Debye function for the energy. The latter can be approximated for *T* > θ_D_ by
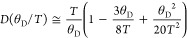
24

Inserting eq 24 into eq 23, carrying out the differentiation, and taking the instability into account, the expression for *T*_mel_ becomes
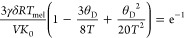
25The results of calculations for 24 metals and 2 salts leads for the linear fit *T*_mel,calc_ = *cT*_mel,exp_ to *R*^2^ = 0.991 and slope *c* = 1.058 with as the largest relative difference 15.8% for Li. Although still an overestimate, this constitutes a serious improvement over the results of Ida.^97^

The most important results of the above study are probably the predictions for *K*_*T*_ and ∂*K*_*T*_/∂*T* for *T* → 0 and for *T* → *T*_mel_. For the former case the results indicate that *K*_*T*_ → *K*_0_ and that ∂*K*_*T*_/∂*T* approaches 0 for *T* → 0, as expected conform the third law. For the latter case *K*_*T*_ → *K*_0_/e, while ∂*K*_*T*_/∂*T* approaches −∞. Hence, the thermoelastic instability occurs for a finite value of *K*_*T*_.

The above result gives support to the attempt to remedy the situation by Tallon,^99−101^ who used the observation that the shear moduli of a solid seem to extrapolate to 0 at *V*_L_. Hence, it was proposed that melting occurs when the solid can transform isothermally to a state of zero shear modulus. Obviously, although this interpretation introduces the required discontinuous nature, it does not apply to crystals with Δ*V*_mel_ < 0. Wang et al.^102^ have shown with MD simulations using LJ potentials for Au that indeed the elastic constants vanish upon increasing the volume, but that it is the constant μ′ = *C*_11_ – *C*_12_ that becomes 0 first. Wautelet and Legrand^103^ indicate that such an instability can be triggered by defect–phonon interactions.

An attempt to reconcile the Born instability approach with the Lindemann relation using a *J*_1_ – *J*_2_ lattice model in combination with a vibrational Hamilton function for an surface-free FCC crystal was given by Zhou and Jin.^104^ Their model invokes interstitials, and in order to do so, they use the NaCl lattice for which at *T* = 0 only one type of site is occupied so that the FCC lattice with octahedral holes is generated. For *T* > 0, the configurations of the atoms over all lattice sites and all octahedral holes were taken, with as nearest-neighbor (NN) interaction *J*_1_ for an atom at a regular lattice site with an atom at a hole site and next-nearest-neighbor (NNN) interaction *J*_2_ for an atom on a regular lattice site with an atom on a nearest regular site or an atom at a hole site with another atom at a nearest hole site. To that the high-temperature vibrational Helmholtz energy 3*NkT* ln(βℏϖ) was added with ϖ the geometric mean frequency of the vibrations of the *N* atoms. Here *h* = 2πℏ is Planck’s constant, *k* is Boltzmann’s constant, and β = 1/*kT*. The equations were solved using a variational approach in the mean-field approximation with periodic boundary conditions and resulted in a discontinuous transition. The authors argue that the configurational entropy is insensitive with respect to sign flip, reminiscent of the lattice gas model where the NNN attraction describes the liquid phase irrespective of the NN repulsions. Their results led to cooperative clusters, or spherical domains of instability, with a size *L* ≠ 0 for *T* → 0 while *L* = 0 for *T* > *T*_mel_, in which atoms behave isotropically. Hence, the difference *C*_44_ – 1/2(*C*_11_ – *C*_12_), where *C*_*ij*_ are the elastic constants, vanishes. Moreover, the average displacement of these atoms exceeds a certain fraction ξ of the interatomic distance *a*, leading for *T* → *T*_mel_ to 3*kT*_mel_ = ⟨ω^2^⟩*ma*^2^ξ^2^ with ⟨ω^2^⟩ the average square frequency. Hence, both the Born and the Lindemann criteria are fulfilled, albeit in a particular way, because as long as the material remains solid, Born’s criterion is only fulfilled within the instability domains. The authors conclude that the most influential factor determining the Lindemann criterion is what they call the profile of the interaction and not the precise details. However, although there is a “universal” value for ξ for van der Waals crystals, there is no guarantee there is a “universal” value for all FCC crystals. With the exception of Gupta’s result,^48^ we have seen from various other results that a “universal” value for ξ for van der Waals crystals (rare gas solids) is reasonable, while for FCC metals another “universal” value is more closely observed.

Finally, we refer to an extensive discussion of instability theory, as known before 1985, provided by Boyer.^105^

### 5.4 Vacancies

**Figure 7:**
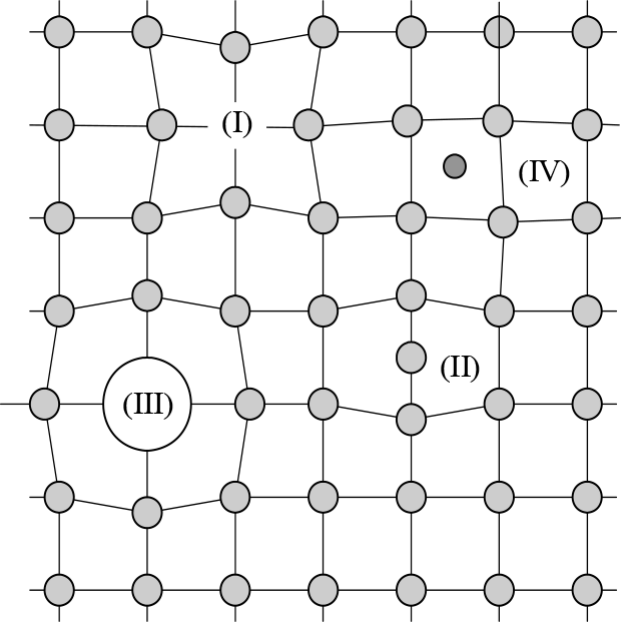
Schematic of various point defects. (I) Vacancy, (II) interstitial, (III) substitutional impurity, and (IV) interstitial impurity.

The first attempts to give a description of liquids with the free-volume concept (i.e., introducing sufficient vacancies (holes) in a solid) were made by Eyring^106^ and Eyring and Hirschfelder.^107^ The (semi)quantitative development by Cernuschi and Eyring^108^ included melting but was criticized by Kirkwood^109^ for neglecting thermal motion. Within that simple picture, Cernuschi and Segre^110^ tried to remedy this defect by introducing vibrations using the Einstein model, which led to qualitatively correct behavior. Frenkel^111^ also recognized that, for the S–L transition to occur, crystals must lose their long-range order and therefore must contain thermally accessible defects. The only thermally accessible defects are vacancies, interstitials, and interstitialcies (Figure 7). From an exposition on fusion,^9,111^ it became clear though that the normal increase of the amount of vacancies in a crystal with temperature cannot account for the volume increase upon melting. If we have *N* atoms and *Nα* holes with α ≪ 1 on a lattice with *N*(1 + α) sites, the number of possible configurations is
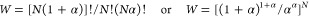
26using Stirling’s approximation *N*! = (*N*/e)^*N*^. Hence, for the entropy we have
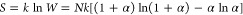
27

The ratio Δ_mel_*H*/Δ_vap_*H* for metals is typically 0.04, and this suggests that α ≈ 0.04 so that *S* ≈ 0.17*Nk* = 0.33 cal K^–1^ mol^–1^. A typical experimental value for entropy of fusion is *S*_mel_ ≈ 2 cal K^–1^ mol^–1^, so a vacancy fraction α ≈ 0.04 cannot account for this. To account for such a value of *S*_mel_, a value of α ≈ 1 would be required, leading to a liquid metal density of about half that of solid metal. Similarly, for molecular compounds, Δ_mel_*H*/Δ_vap_*H* ≈ 0.1 and hence α ≈ 0.1, with the corresponding *S*_mel_ ≈ 0.34*Nk* = 0.66 cal K^–1^ mol^–1^, while experimentally *S*_mel_ ≈ 5 cal K^–1^ mol^–1^ is observed. This leads again to a rather large density change. Since these changes are not observed, other effects, to which we come later, must come into play. Possibly more fundamental is the fact that the concept of vacancies in a lattice as a model for a liquid imply long-range order in a liquid, which is, however, not observed.

Smirnov^112^ also showed that a vacancy model to describe melting of rare gas solids is not applicable and proposed as an alternative an approach using the interaction between icosahedral and FCC structures.

Nevertheless the vacancy theory was elaborated by Gorecki,^113−115^ who, focusing on metals and ignoring the entropy argument, assumed that the mechanism of melting is connected to the introduction of vacancies in the solid phase. It was shown that for a range of FCC, HCP and BCC metals the vacancy concentration *c*, defined by *c* = *Nα*/(*N* + *Nα*), is given by
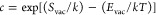
28where *S*_vac_/*k* = 4.1 and *E*_vac_/*T*_mel_ = 80.4 J K^–1^ are the empirically determined entropy and enthalpy (energy) of vacancy formation, respectively. Hence, one easily calculates that *c*_vac_(*T*_mel_) = 0.0037, in good agreement with experimental data for all the metals studied. Such a low bulk concentration of vacancies will not lead to lattice instability. A further correlation of the enthalpy of melting Δ_mel_*H* with *E*_vac_ yielded Δ_mel_*H*/*E*_vac_ = 0.127, sufficient for a vacancy concentration increase of Δ*c* = 0.13 upon melting. A similar correlation using the electrical resistivity yielded Δ*c* = 0.07. Experimentally it appears that the volume increases upon melting are Δ*V*/*V*_S_ ≅ 0.05 for FCC and HCP metals and Δ*V*/*V*_S_ ≅ 0.025 for BCC metals. As it is also known that for FFC and HCP metals the mean atomic volume for a defect lattice *Ω*_vac_ ≅ (1/2)*Ω*,^116,117^ the model implies Δ*c* ≅ 2Δ*V*/*V*s ≅ 0.10. Overall, Δ*c* thus appears to be Δ*c* ≅ 0.10.

Using a factor ζ due to lattice relaxation around a vacancy , *Ω*_vac_ = *Ω* + *ζcΩ*. Considering that *Ω* = 4π*r*^3^/3 and *Ω*_vac_ = 4π(*r* – Δ*r*)^3^/3, one obtains *c* = ζ^–1^[(*Ω*_vac_/*Ω*) – 1] = ζ^–1^{[(*r* – Δ*r*)/*r*]^3^ – 1}. Furthermore, the empirical correlation *x* ≡ Δ*r*/*r* = 3.0*E*_vac_/*EV*_m_ with *E* Young’s modulus, and *x*_FCC_ = 0.24, *x*_HCP_ = 0.21, and *x*_BCC_ = 0.37, is used. Hence, *Ω*_vac_ = (1 – 3*x* + 3*x*^2^ – *x*^3^)*Ω*, which leads to ζ_FCC_ = −0.56, ζ_HCP_ = −0.51, and ζ_BCC_ = −0.75, for FCC and HCP consistent with the experimental estimate Δ*V*/*V*_S_ ≅ 0.05. Interpreting for the liquid *r* – Δ*r* as the radius of the first coordination shell leads to, using the available experimental data, Δ*c* ≅ 0.09, in good agreement with the aforementioned estimates. Note that, although one might expect upon melting an increase in the radius of the first coordination sphere, actually a small decrease occurs. The model was further used to calculate d*T*_mel_/d*P*, with results also in fair agreement with experiment. In conclusion, the approach is quite successful describing melting of metals, but the reason why Δ*c* ≅ 0.1 has not been made clear.

Other authors^118−120^ have also dealt with vacancy theory for melting. Generally, they all take the same general form but differ in detail. An early review on defect-mediated melting by MC studies based on gauge theory is given by Janke.^121^ A somewhat different approach is by Karasevskii et al.^122^ using the Gibbs–Bogoliubov functional for the Gibbs energy in the high-temperature approximation of rare gas solids, dependent, next to *P* and *T*, on two internal variables, the quasi-elastic bond parameter and one for lattice expansion. A Morse potential was used to describe the pairwise interaction of neighboring atoms, while for the van der Waals attraction for non-nearest neighbors ϕ_NN_ = −4ε(σ/*r*)^6^ was employed. The effect of the creation of vacancies upon the local configuration as well as the associated energy changes was taken into account up to the fourth coordination sphere. Comparison with the results for a quasi-harmonic crystal showed that the cubic vibrational anharmonicity is responsible for a dramatic decrease of the vacancy formation energy near *T*_mel_. Thus, at lower temperature divacancies are preferred, while at higher temperature single vacancies dominate. The reason is that the number of bonds to be broken per vacancy in the case of a divacancy is less than that for two single vacancies. However, at higher temperatures, the major gain in the formation energy of structural defects is due to the redistribution of atomic displacements around the vacancy. Creation of a divacancy results in an overlap of relaxed areas, thus reducing the total gain in the defect energy. As a result, at temperatures about 7% below *T*_mel_ the energy of a divacancy becomes greater than the sum of the energies for two single vacancies; i.e., there is some effective repulsion between the vacancies at a high concentration. For the melting point the authors argue that using a two-phase theory is difficult and resort to a previous paper^123^ where it was shown that the effective phonon-mediated interaction between vacancies results in a first-order phase transition in the subsystem of the vacancies. This first-order transition occurs when the Gibbs energy of vacancy formation *G*_v_ reaches *G*_v_ = 5.25*kT* and is accompanied by a discontinuous and large increase in the number of vacancies. Hence it was identified as the melting transition. Summarizing, the incorporation of the cubic anharmonicity of atomic vibrations can be considered as an attraction between the phonons. The magnitude of this attraction and the equilibrium phonon concentration increase with *T*. As the system approaches the critical temperature, where the rate of vacancy creation approaches −∞, the phonon concentration increases at an infinite rate. However, before this critical temperature is reached, the energy for the creation of structural defects drops sharply, resulting in a first-order phase transition to a phase saturated with defects, which is identified as the liquid phase.

In a condensed presentation Tovbin^124^ discussed what he called the “fundamentals of the theory of melting” in the lattice gas approximation using the method of quasi-averages, introduced by Bogoliubov^125^ (for a brief introduction, see ref (126)). Using an LJ potential, allowing for vibrations in the solid state and vibrations and translations in the liquid state, a general scheme was described, to be solved in the quasi-chemical approximation. Unfortunately, no concrete results from any calculation were presented.

Finally, we note that attempts have been made to use two types of defects to explain melting. For example, Liu and Chen^127^ discuss the combined effect of dislocation pairs and point defects. The model led to a discontinuous transition whereby melting occurs due to a discontinuous growth of point defects into dislocation pairs. For five alkali metals the agreement with the calculated transition point and experiment is fair. The enthalpy derived is related to the core parameter and energy of the dislocation, and by adjustment of the core parameter the enthalpy obeys Richard’s rule.

### 5.5 Interstitials

Essentially the vacancy model by Cernuschi and Eyring^108^ is a special form of the order–disorder problem.^128^ The idea of melting being an order–disorder problem was also addressed by Wannier,^129^ who concluded (again) that melting is due to the breakdown of crystalline long distance order, but also that his “method used is not likely to be of value for quantitative purposes”. Another early, but detailed attempt,^33^ essentially employing the interstitial holes in a lattice, uses in its simplest form for both the liquid and the solid state the LJD theory.^130−132^ In that theory the FCC lattice was used for the liquid using the Einstein approximation for the vibrations and the approximate partition function *Z* reading, using β = 1/*kT* and *Λ* = (*h*^2^/2π*mkT*)^1/2^, is given by
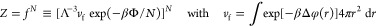
29

Here *f* is the single particle partition function, *Φ* = (1/2)*Nzϕ*_0_ with ϕ_0_ the potential at the center *r* = 0 of the cell, *z* the coordination number, Δϕ(*r*) = ϕ(*r*) – ϕ_0_ the potential at position *r* within the cell with respect to its center, and *v*_f_ the free volume. Further, recall that the FCC lattice contains an equal number of lattice sites (here labeled as α-sites) and interstitial, or hole, sites (here labeled as β-sites), the latter also being arranged in an FCC manner. It is now assumed that in the solid state the molecules occupy the α-sites only, while during melting the β-sites become available. For the fusion process we then have an order–disorder description at hand, for which we can use the conventional zeroth or first approximation. As the zeroth approximation yields essentially the same results as the first and the zeroth is much simpler, the zeroth approximation is used here.

If the relative occupancy of α-sites is given by *X* = *N*_α_/*N*, *X* = 1 represents the ordered state, while *X* = 1/2 denotes complete disorder. In the zeroth approximation in a disordered state, there will be *XN* molecules on α-sites and the α–α interaction becomes (1/2)*Xzϕ*_0_·*XN* (instead of (1/2)*zϕ*_0_·*N* for the ordered, i.e., solid, state). Since the α- and β-lattices are similar, the β–β interaction becomes 1/2(1 – *X*)*zϕ*_0_·(1 – *X*)*N* while for the α–β interaction the result is (1 – *X*)*zχ*_0_·*XN* with χ_0_ the difference in energy between α- and β-sites. The total energy therefore becomes, using ψ = χ_0_ – ϕ_0_
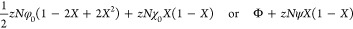
30

Next, the number of ways γ(*X*) of choosing *N*_α_ molecules over the α-sites and *N*_β_ molecules over the β-sites is needed, which is given by
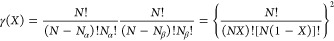
31

Finally, it is assumed that the motion of a molecule on an α-site is the same as that on a β-site. In that case the partition function becomes
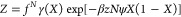
32Minimizing the Helmholtz energy *F* = −*kT* ln *Z* with respect to *X*, one obtains, after some calculation
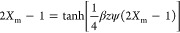
33

**Figure 8:**
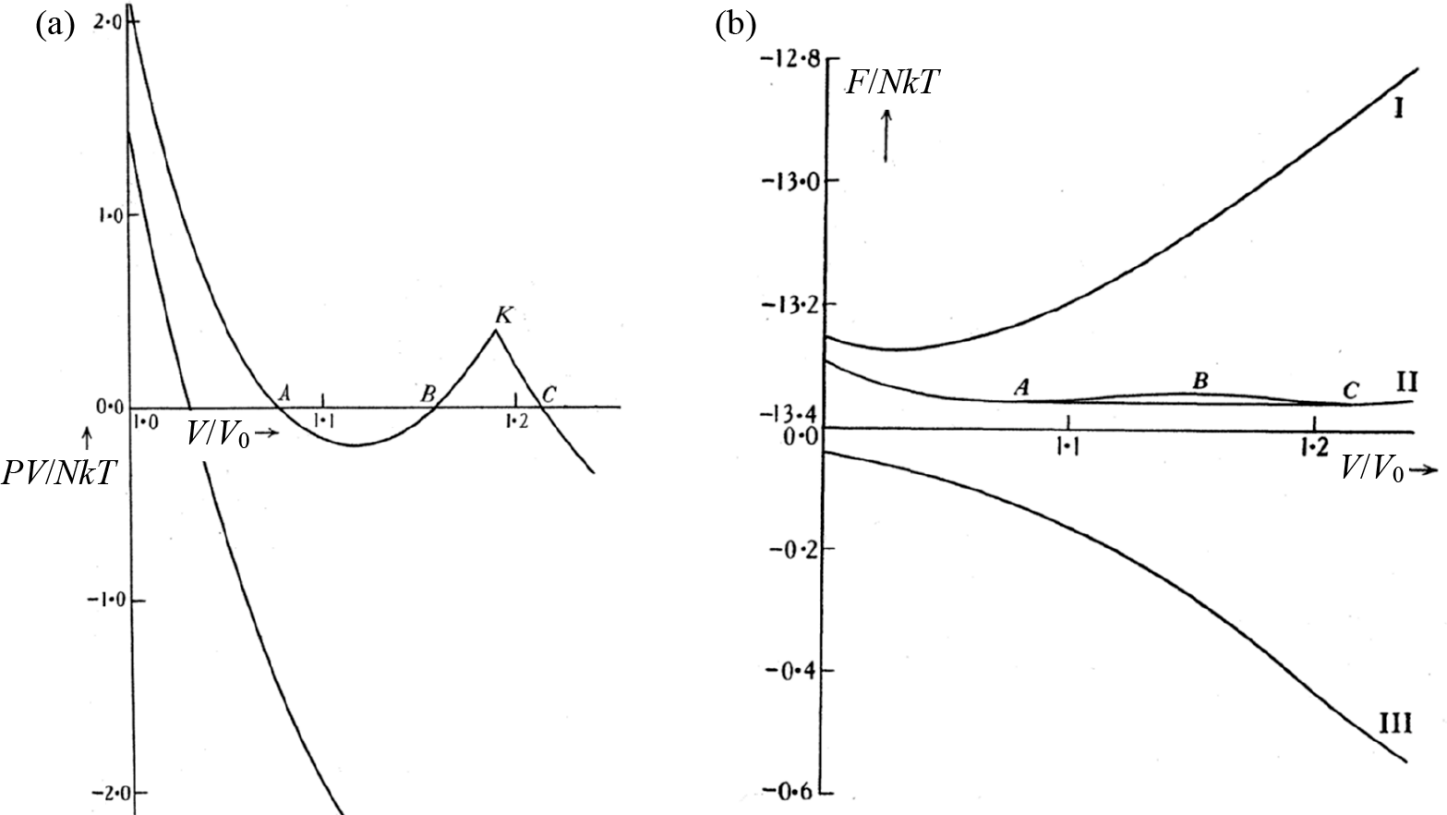
LJD fusion model for Ar. (a) The pressure *P* as a function of volume, where the lower curve represents the pressure for the ordered state and the upper curve represents the total pressure. (b) The Helmholtz energy *F* for the ordered state (I), the disorder contribution (III), and the total (II). The points A, B, and C correspond to the same points as in (a). Reproduced with permission from ref (132). Copyright 1939 The Royal Society.

This equation is always satisfied when *X*_m_ = 1/2, but when *βzψ*/4 > 1 there is another root larger than 1/2, which in that case minimizes *F*. For *βzψ*/4 ≫ 1, *X*_m_ ≅ 1 and the order is nearly perfect (in fact, it describes the solid with a few interstitials). When *βzψ*/4 decreases, the disorder increases, and when *βzψ*/4 < 1, *X*_m_ = 1/2 and there is complete disorder. Substituting the value of *X*_m_ in the partition function, one obtains
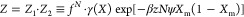
34so that the Helmholtz energy becomes *F* = *F*_1_ + *F*_2_, where *F*_1_ is the Helmholtz energy for the ordered state and *F*_2_ is the additional Helmholtz energy due to disorder (Figure 8b). Hence for the pressure *P* = −∂*F*/∂*V* = *P*_1_ + *P*_2_ with
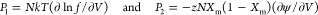
35

If one further assumes that ψ = ψ_0_(*r*_0_/*r*)^12^ = ψ_0_(*V*_0_/*V*)^4^, that is, we assume that only repulsion contributes, *P* can be calculated as a function of *T*, since for a given volume *V*, *Φ*, and ψ are known, while *X*_m_ can be calculated from eq 33. This results in a curve as shown in Figure 8a (the sharp peak *K* is due to the use of the zeroth approximation). The pressure *P* is zero for three values of *V*/*V*_0_. The smallest one corresponds to the volume of the solid at melting, while the largest corresponds to the volume of the liquid. With the use of the Maxwell equal-area rule, ψ_0_ can be determined in such a way that the melting pressure for a particular compound is zero. This has been done for Ar (*T*_mel_ = 83.8 K) and leads to Δ*V* = *V*_L_ – *V*_S_ = 1.214*V*_0_ – 1.078*V*_0_ = 0.136*V*_0_. For the entropy the result is *S* = 1.70*k* and both values are in reasonable agreement with experiment. It was found that for these volumes *kT*/ε ≅ 0.7, where ε is the depth of the LJ potential used. The melting point *T*_mel_ is thus *T*_mel_ ≅ 0.7ε/*k*. With the use of this rule, reasonably good agreement for *T*_mel_ was obtained for Ne, N_2_, CO, CH_4_, and H_2_. With the use of a slightly different formulation including interactions up to the third coordination shell,^133^ agreement between theory and experiment for the melting curves is very good for the rare gases and fair for NH_3_, N_2_, CO_2_, and CH_4_.

Since molecules generally have not only translational (positional) but also orientational degrees of freedom, any theory of melting of molecular crystals should take them into account, as done by Pople and Karasz^134^ in the framework of the interstitial approach discussed here. These authors restricted their discussion to two orientations, but Amzel and Becka^135^ extended the model to *n* > 2 orientations. Such crystals are sometimes called, somewhat confusingly, “plastic” crystals. Using the notation of Ubbelohde,^7^ Tozzini et al.^136^ described the *n* = 2 model by
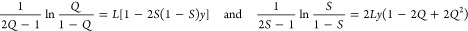
36where *Q* represents the site fraction and *S* represents the orientation fraction. The parameter *L* = (1/2)*βzε* characterizes the barrier for translation, while the parameter *y* = *z*′ε′/*zε* represents the ratio of the barriers for orientation and translation. The model thus deals with the melting temperature *T*_mel_ or *t*_mel_ = 2*kT*_mel_/*zε*, which decreases with increasing *y*, and an orientational disordering temperature *T*_cri_ or *t*_cri_ = 2*kT*_cri_/*zε*, which increases with *y*, until they meet at *t*_mel_ = *t*_cri_ ≅ 0.35 for *y* ≅ 0.595. Once the temperature is scaled via *L*, both *T*_mel_ and *T*_cri_ are functions of *y* and *t*_mel_/*t*_cri_ is a “universal” function of 1/*t*_cri_, for 5 < 1/*t*_cri_ < 20 approximately represented (not given) by *t*_mel_/*t*_cri_ = 0.50/*t*_cri_ – 0.25. The authors indicated that “plastic” crystal phase studies of, e.g., a light halogen, could provide an experimental test.

Bhattacharya et al.^137^ revived the model by using the embedded atom method for the potential for the solid,^138^ a corrected rigid sphere model (CRIS^139−141^) for the liquid, and adding a correction for correlated atomic motion and anharmonicity.^142^ Calculations were done for Ar, from which the need for a correlation correction became clear, and as a function of pressure for Al (3 Mbar), Cu (3.5 Mbar), Ni (3 Mbar), and Pt (2 Mbar). It appeared that to obtain agreement with experiment it was necessary to include an empirical correction of the form *A* + *BT*, where *A* corrects for the difference in binding energies of the solid and liquid phases and *B* accounts for the neglect of correlation in the single particle cell model. These constants can be determined from the zero pressure melting temperature.

The original interstitial approach takes properly into account that both solid and liquid should be involved and results in quite reasonable values for the various properties. Moreover, the model also provides an explanation for Lindemann’s rule. However, it predicts a critical point for the solid–liquid transition, similar as for the liquid–vapor transition, which has never been observed experimentally, but using a compressible lattice model this can be avoided, as shown from thermodynamics^143^ and statistical mechanics of the Ising model.^144,145^ The isobars *S*(*T*) display a monotonic rise instead of being S-shaped, as required for melting being a discontinuous transition. Finally, as well-known, the liquid state is poorly described as insufficient disorder is introduced.

### 5.6 Dislocations

Several melting theories are based on dislocations, for which the basic idea is that a solid becomes a liquid when the solid is saturated with dislocations. Such dislocation theories of melting (DTMs)^146,147^ have appealing aspects. First, although dislocations have an excess energy over the ideal lattice, the Gibbs energy of a solid containing a dense network of dislocations and that of a liquid remain comparable. This leads to a low enthalpy of fusion, comparable with the core energy of dislocations. Second, the fluidity can be ascribed to the mobility of a dense network of dislocations.^148^ Pairs of opposite sign dislocations (loops) can be created thermally and, most importantly, the presence of dislocations reduces the formation energy of additional dislocations, so a cooperative effect creating an avalanche of dislocations can be envisaged to occur. When the Gibbs energy difference Δ*G* = *G*_L_ – *G*_S_ = 0, a discontinuous transition occurs. As both *G*_L_ and *G*_S_ are involved, one can consider DTMs as two-phase theories. There is some evidence for dislocation-like structures in liquids.^149^

The birth of DTMs can be considered to be a paper by Mott,^150^ rapidly followed by others.^146,151−153^ Here we consider mainly a variant originated by Ninomiya^154^ and in slightly modified form applied by Poirier.^155−157^ In this model the energy *U* of a lattice with dislocations contains essentially three terms. The first term is the energy of the core of the dislocations Δ*U*_cor_. This is usually considered to be a constant per unit dislocation length for which the enthalpy of melting appears to be a rather good estimate.^158^ Measured in units *μb*^2^/4π with μ the shear modulus and *b* the Burgers vector length, the core energy per unit length can be expressed as *u*_cor_ = *cμb*^2^/4π with *c* ≅ 1. The total core energy Δ*U*_cor_ is thus *ρV*_m_*u*_cor_ with the dislocation density ρ = *L*/*V*, where *L* is the length of dislocations per unit volume *V* of crystal and *V*_m_ is the molar volume.

The second term is the elastic energy of the dislocations Δ*U*_ela_. Here we use the logarithmic energy expression *u*_ela_ = (*αμb*^2^/4π) ln(*R*/*r*_0_) for the energy unit length, as we need an explicit dependence on ρ. The constant α = 1 for screw dislocations and α = 1/(1 – ν) for edge dislocations; ν is Poisson’s ratio, *r*_0_ is the core radius, and *R* is the average distance between dislocations. The latter is estimated using the average stressed cross section along a dislocation ρ^–1^. As the core is considered to be circular with area π*r*_0_^2^, and *u*_ela_ should become zero when *R* = *r*_0_, the proper estimate is *R* = (πρ)^−1/2^. The total elastic energy is thus Δ*U*_ela_ = *ρV*_m_*u*_ela_. However, the introduction of dislocations leads to a volume strain ε(ρ), so *V* = *V*_0_(1 + ε), thereby reducing μ and increasing *b*. Using d ln μ/d ln *V* = −(2γ + 1/3) as given by Slater,^9^ μ = μ_0_[1 – (2γ + 1/3)ε], where γ is the Grüneisen parameter, while *b* = *b*_0_(1 + ε)^1/3^.

The third term is the energy due to the expansion of the material as a whole given by Δ*U*_exp_ = (1/2)*N*_A_*ΩKε*^2^, where *K* is the bulk modulus and *Ω* = *V*_m_/*N*_A_ is the atomic volume. The total energy change is thus Δ*U* = Δ*U*_cor_ + Δ*U*_ela_ + Δ*U*_exp_ or
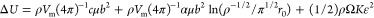
37

The value of ε_dis_ corresponding to the dislocation density ρ is obtained by minimizing Δ*U* with respect to ε. The result is, taking care of the strain dependence of *V*, *b*, and μ and neglecting small terms,^159,160^
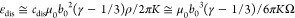
38where *c*_dis_ = *c* + α ln(ρ^–1/2^/π^1/2^*r*_0_). The last step can be made as saturation of dislocations is given by the condition π^1/2^*r*_0_ ≅ ρ^–1/2^ and *c* ≅ 1, so *c*_dis_ ≅ 1, while the maximum dislocation density is estimated as ρ_max_ ≅ *b*/3*Ω*.^161^ The factor *b*_0_^3^/*Ω* depends only on the lattice type and is √2, 3√3/4, and 4/√3 for FCC, BCC, and (ideal) HCP lattices, respectively. The volume change upon melting becomes Δ*V* = *N*_A_*Ωε*_dis_ = *V*_m_ε_dis_, while the internal energy change Δ*U*, obtained by substituting eq 38 into eq 37, reads
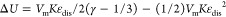
39

The entropy of the dislocations Δ*S* contains configurational and vibrational contributions. The first is generally negligible,^161^ while the vibrational entropy contains a term due to the decrease in lattice frequencies brought about by the dilatation ε_dis_ and a term due to the vibration of the dislocation lines. When a crystal with average (or Einstein) vibration frequency ω contains dislocations, the vibrations associated with the dislocations change frequency to ω′. Hence, in the high-temperature approximation to vibrations, we have, using *f* = *Ω*ρ/*b*
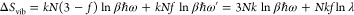
40where λ = ω′/ω, estimated by Ninomiya^154^ to be ≅ 0.13, so that ln λ ≅ −2.0. Moreover, due to the dilatation, the lattice frequency itself changes and using the Grüneisen relation, d ln ω/d ln *V* = −γ with γ the Grüneisen parameter, one obtains ω = ω_0_(1 – *γε*). From the Helmholtz energy Δ*F* = Δ*U* – *T*Δ*S*, the total dilatation is given by ∂Δ*F*/∂ε = 0, and this results in ε = (3*kγ*/*KΩ*)*T* + ε_dis_. Inserting ε in the expression for Δ*F*, the entropy Δ*S* = −∂Δ*F*/∂*T* becomes
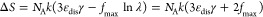
41

**Figure 9:**
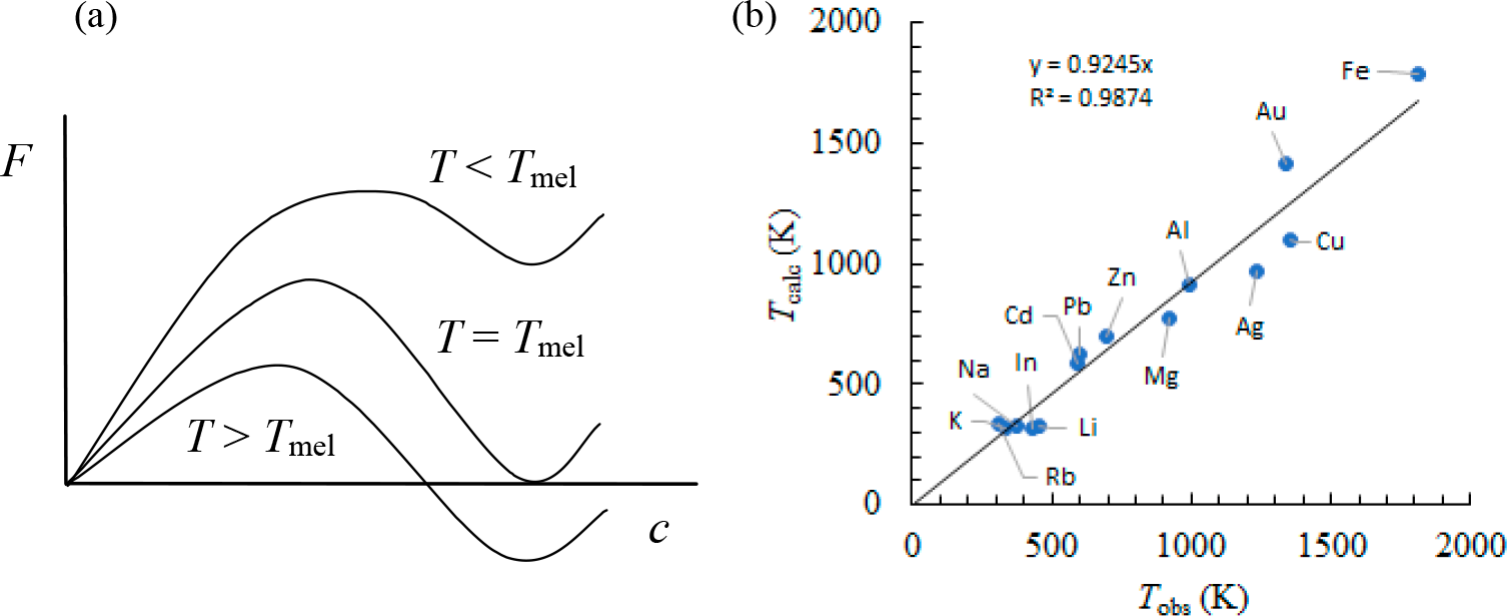
DTM illustrated. (a) Helmholtz energy *F* as a function of dislocation concentration *c* as used in refs (154−156). (b) Correlation between calculated and experimental *T*_mel_ drawn after data from ref (156).

The melting temperature *T*_mel_ is obtained by setting ρ = ρ_max_ and taking the Helmholtz energy Δ*F* = Δ*U* – *T*Δ*S* = 0 (Figure 9a). The final expression becomes
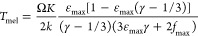
42

From the Clausius–Clapeyron equation d*T*/d*P* = Δ*V*/Δ*S*, one also has
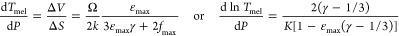
43Equation 43 differs from the differential Lindemann expression d ln *T*_mel_/d*P* = 2(γ – 1/3)/*K* only by the factor [1 – ε_max_(γ – 1/3)]^−1^, which is somewhat larger than 1 for γ > 1/3. The latter condition is always true, and thus the DTM also provides some justification for Lindemann’s rule. Estimates for *c*, ρ_max_, and λ already being made, one needs only estimates for the material properties μ, *K*, and γ, and knowing the crystal structure, *T*_mel_ can be calculated, as illustrated in Figure 9a. For Fe Poirier^155^ obtained *T*_mel_ = 1786 K, while experimentally *T*_mel_ = 1808 K is obtained. A good agreement with experiment is thus observed.

It appeared, however, that the agreement for Fe was a somewhat lucky shot as the results for other metals differ significantly from the experimental data, but taking the experimental values for Δ*V* and Δ*S*, Poirier^156^ obtained reasonable agreement for 14 metals studied (Figure 9b). A recent revival of the DTM by Burakovsky et al.,^160,162,163^ using a slightly different approach, the melting temperatures and latent heats are estimated for about 70 elements with an accuracy of about 20%.

### 5.7 Interstitialcies

**Figure 10:**
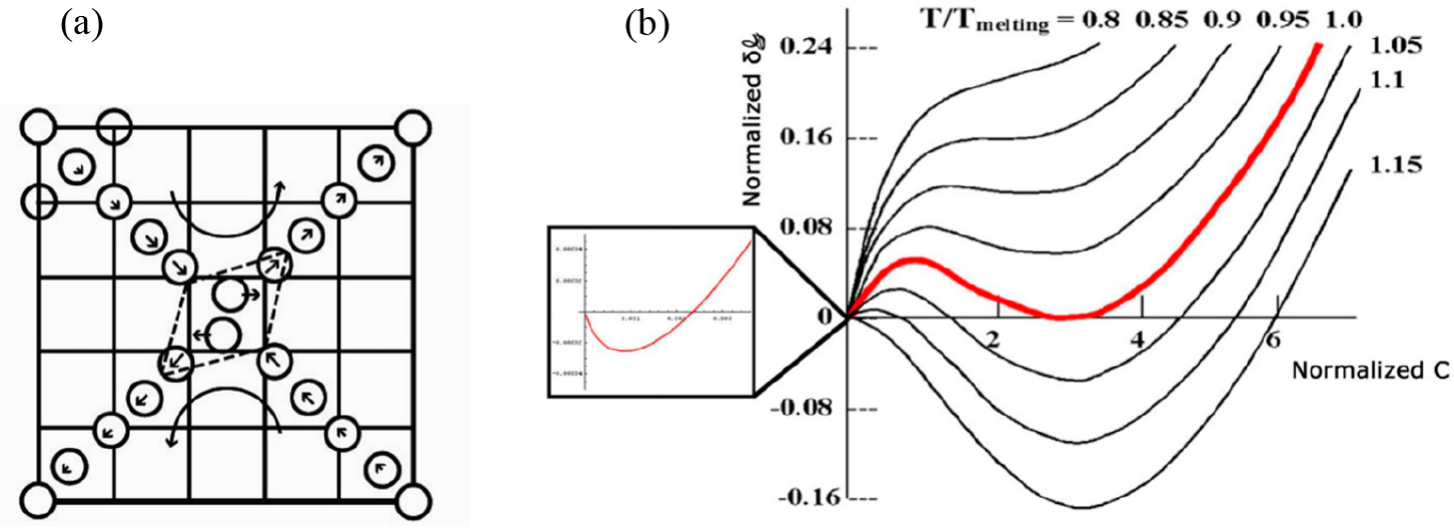
Interstitialcy theory illustrated. (a) Interstitialcy configuration in a [100] direction for an FCC lattice. Arrows indicate displacements of atoms along a close-packed [110] direction on a {100} plane for an applied shear stress and the so-called E_g_ resonance mode. (b) Normalized Gibbs energy as a function of normalized interstitialcy concentration. Reproduced with permission from ref (166). Copyright 2006 Elsevier.

A relatively recent “defect” approach is based on the interstitialcy (or a dumbbell interstitial, that is, two atoms trying to occupy the same lattice site), as studied primarily for FCC metals, in particular Cu, by Granato.^164,165^ It is argued that dislocations have a too high energy per atomic length to be generated thermally. As indicated in section 5.4, thermal accessibility of defects involved in melting is considered to be necessary. While vacancies are thermally accessible, they result in a too low value for the melting entropy *S*_mel_. Interstitialcies are also thermally accessible but have a strong coupling to external shear stress, low-frequency resonance modes, and an extended linear string-like character (Figure 10a), leading to large entropy effects. Based on these considerations, a liquid was considered to be a crystal containing a certain percentage of interstitialcies in thermal equilibrium, for which an interstitialcy concentration dependent Gibbs energy expression was derived.

It is supposed that the volume *V*_0_, the bulk modulus *K*_0_, shear modulus μ_0_, evaluated for the static lattice, and their pressure derivatives *K*′ and μ′ are given, and that the Helmholtz energy *F*_0_(*V*,ε) with ε the shear strain can be expressed in terms of these parameters. Accepting the Einstein model for vibrations at high temperature with frequency ω_E_, the Helmholtz energy *F* at finite temperature for *N* atoms is given by *F* = *F*_0_ + 3*NkT* ln(βℏω_E_), where *k*, β, ℏ, and and ω_E_ have the usual meaning. Introducing *n* defects, we must add the work for creating these defects Δ*F*_for_, the change in vibrational behavior Δ*F*_vib_, and a configurational term Δ*F*_con_.

The first contribution is assumed to be d*f*_for_ ≡ d*F*_for_/*N* = (*α*_*μ*_*μΩ* + *α*_*K*_*KΩ*) d*c* with *Ω* the atomic volume and *c* = *n*/*N* the concentration of interstitialcies, or, equivalently, *f*_for_ = ∫(*α*_*μ*_*μΩ* + *α*_*K*_*KΩ*) d*c*. It is expected, based on simulations, that for the constants *α*_*μ*_ and *α*_*K*_ we have *α*_*K*_/*α*_*μ*_ ≪ 1, so the work of formation is mainly due to shear deformation (a fit for Cu yields *α*_*μ*_ ≅ 0.9 and *α*_*K*_ ≅ 0.03). The second contribution is due to the five low-frequency resonance modes with frequency ω_R_ and the six high-frequency local modes with frequency ω_L_ for each interstitialcy, as described by Dederichs et al.^167^ This leads to d*f*_vib_ = −*kTc*[5 ln(ω_E_/ω_R_) + 6 ln(ω_E_/ω_L_)]. The third contribution reads −*TS*_con_ = −*kTc*[1 + ln(*z*/*c*)] with the degeneracy factor *z* as an interstitialcy that can be oriented along any of *z* directions (*z* = 3 for FCC lattices).

The dependence of *K* on shear strain ε is neglected, and μ is described by μ(*V*,ε,*c*) = μ_0_(*V*,ε) + α_μ_∫(∂^2^μ/∂ε^2^)(*Ω*/*Ω*_0_) d*c* or, equivalently, by ∂μ/∂*c* = *α*_*μ*_(∂^2^μ/∂ε^2^)(*Ω*/*Ω*_0_).^164,165^ As μ must be periodic in displacement *x* with lattice distance *b* for lattice planes separated by a distance *d*, the simplest periodic even function, μ = μ(*V*,*c*) cos(2π*dε*/*b*) with ε = *x*/*d*, is chosen. Hence, ∂^2^μ/∂*c*^2^ = −*ξμ* with ξ = 4π^2^*d*^2^/*b*^2^, with leads to, since *Ω*/*Ω*_0_ ≅ 1, μ = μ_0_(*V*,ε) exp(−*α*_*μ*_*ξc*). For *α*_*μ*_ ≅ 1 and *d* ≅ *b*, ξ ≅ 4π^2^ ≅ 40, so a concentration of, say, 3%, should reduce μ to ≅ 0.3 of its original value. For Cu the dependency of μ on *c* is known and indeed shows a rapid decrease of the elastic constant *C*_44_ with d ln *C*_44_*/*d*c* ≅ 31.

For the Gibbs energy *G* = *F* + *PV*, one has to add a term 3*NkT* ln[ω_E_(*V*)/ω_E_(*V*_0_)]. Also, we might expect that ω_R_ is reduced under constant pressure conditions. As a precise analysis is complex, the behavior is approximated by ω_R_ = ω_R0_(1 + *λα*_*μ*_*ξc*), where one expects λ < 1. Using *r* = (ω_E_/ω_R_)/(1 + *λξc*), ln ω_E_/ω_R_ = 0.87*r* – 1.05 is a good approximation according to Granato. With *V* = ∂*G*/∂*P* and ω_E_(*V*)/ω_E_(*V*_0_) = (*V*/*V*_0_)^γ^, the (normalized) Gibbs energy change *y* can be obtained as a function of the (normalized) defect concentration *x* and (normalized) temperature *t* (after some manipulation) as
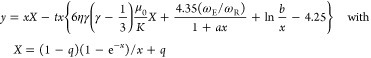
44

Here *y* = [Δ*G*(1 – *q*)ξ]/*NG*_0_*V*_0_, *x* = *α*_*μ*_ξ*c*, *t* = *kT*/*ηGΩ*, *t*_rel_ = *T*/*T*_mel_, *ημV* = *α*_*μ*_*μV* + *α*_*K*_*KV*, 1 – *q* = *α*_*μ*_/η, *a* = λ/η(1 – *q*), and *b* = *zη*(1 – *q*)ξ(ω_E_/ω_L_)^6^. Moreover, μ′ = (2γ + 1/3)μ/*K* where the Grüneisen parameter γ^9^ is used. We show here the full expression for Δ*G* just to indicate that an analytical theory is obtained. ω_L_/ω_E_ = 1.73 was taken from theory.^168^ The other parameters have been fitted on data for Cu^169^ imposing ω_E_/ω_R_ > 1, which allows taking α = 1, and, using 2μ = *C*_44_ + (*C*_11_ – *C*_12_)/2, yields *αμ*_0_*Ω*_0_ = 3.94 eV, in agreement with experiment. As for ξ < 30 no solution could be obtained, ξ = 35 was fixed. Using *S*_mel_ = 1.15*k* at *T*_mel_ with ω_E_/ω_R_ = 5.3 ± 7% led to λ = 0.206 ± 10%, *q* = 0.075 ± 22%, and *x* = 3.0 ± 17%, so *c*(*T*_mel_) = 0.093 ± 17%.

For this choice of parameters Figure 10b, displaying the behavior of *y* (∼Δ*G*) versus *x* (∼*c*), shows overall behavior very similar to that of DTM. The shallow minimum (shown in the inset) at low *c* is due to the fact that interstitialcies are thermally accessible. Figure 10b also shows that undercooling to *t* ≅ 0.85 is possible. For *t* > 1, that is, a temperature above *T*_mel_, there are three solutions of which the one with the highest *x* is interpreted as equilibrium melting. Melting actually occurs for *y* = d*y*/d*x* = 0, providing the relation *h*_for_ = *ημ*_0_*Ω*_0_ ≅ *ξkT*_mel_ between *T*_mel_ and the enthalpy of formation of an isolated interstitialcy *h*_for_. Assuming η = 1, the data for Cu yield ξ = 33.7. Using the Grüneisen relation *c*_*V*_ = *αKΩ*/γ with α the thermal expansivity and γ Grüneisen’s parameter together with the Dulong and Petit high-temperature value *c*_*V*_ ≅ 3*k*, results in *αT*_mel_ ≅ *γμ*/*ξK*. From Poisson’s ratio ν = (3*K* – 2μ)/2(3*K* + μ), one obtains μ/*K* = 3(1 – 2ν)/2(1 + ν) ≅ *const*., so *αT*_mel_ ≅ *C* with *C* a constant. For Cu, with ν = 0.35 and γ = 3.0, *C*_cal_ ≅ 0.030, to be compared with *C*_exp_ ≅ 0.024. This approach thus interprets melting as reaching an interstitialcy-driven shear instability with no relation to a critical vibration amplitude. Because Lindemann’s rule ⟨*u*^2^⟩^1/2^ = *aC* with *a* the lattice constant can be approximated by Δ*a* = *aC*, the correlation (1/*a*)(Δ*a*/*T*_mel_)*T*_mel_ ≅ *αT*_mel_ = *C* results, and therefore his rule can be rationalized by the interstitialcy theory but, as said, without invoking a critical vibration amplitude.

The relation *T*_mel_ = μ_0_*Ω*_0_/*kξ* can be tested independently and yields with μ_0_ = 48 GPa and *Ω*_0_ = 1.26 × 10^–29^ m^3^ for Cu *T*_mel_ = 1303 K, to be compared with the experimental value of 1358 K, and equivalent to d*T*_mel_/d(μ_0_*Ω*_0_) = 386 K eV^–1^. A fit on 62 elements led to d*T*_mel_/d(μ_0_*Ω*_0_) = 228 K eV^–1^, and Granato et al.^168^ suggested that the actual data points represent approximate formation enthalpies of the interstitialcies. In the absence of adequate data, this correlation can easily be off by a factor of 1.5–2.0, though. As ξ is substance-dependent and several metals show a phase transformation below *T*_mel_, thereby changing their values for μ, *K*, ν, and γ, a strict linear relationship *αT*_mel_ ≅ *const.* is not expected anyway.

Furthermore, as an aside, we mention the intriguing question of how the specific heat *C*_*P*_ about liquids behaves.^169^ Typically, *C*_*P*_ decreases with *T* near *T*_mel_ (before increasing again near *T*_cri_ due to the continuous transition at *T*_cri_). From *C*_*V*_ = −*T* ∂^2^*f*/∂*T*^2^ with *f* = *f*_for_ + *TS*(*c*) one obtains, using ∂*f*/∂*c* = 0 which is equivalent to *T* ∂*S*/∂*c* = ∂*f*_for_/∂*c*, Δ*C*_*V*_ = (∂*f*_for_/∂*c*)(∂*c*/∂*T*). For the crystalline state ∂*f*_for_/∂*c* is the formation energy of a single defect and ∂*c*/∂*T* increases exponentially with *T*. For the liquid state ∂*f*_for_/∂*c* = *α*_*μ*_*μ*(*c*)*Ω* + *α*_*K*_*KΩ* and ∂*c*/∂*T* is approximately constant, and *C*_*V*_ follows the *c*- and *T*-dependence of μ. It appears that, at *T*_mel_, μ(*T*_mel_)/μ_0_ ≅ exp(−*α*_*μ*_*ξc*) ≅ exp(−3) ≅ 0.05, so the two factors in Δ*C*_*V*_ are comparable near *T*_mel_. With increasing *T*, *c* increases, μ decreases, and, hence, *C*_*V*_ decreases. Upon undercooling, *C*_*V*_ increases until no further defects are formed and then returns to the classical value 3*Nk*. The prediction *C*_*P*_ = *C*_*V*_(1 + *αγT*) describes the experimental data for several metals well.

**Figure 11:**
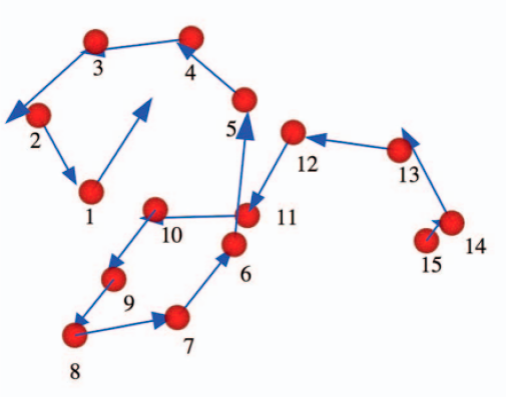
Schematic of a typical string of 15 atoms at *T* = 1840 K, where the arrows indicate the jumps from atom 15 to atom 1. Reproduced with permission from ref (170). Copyright 2013 AIP Publishing.

MD simulations on 13 500 Ni atoms using constant (*N*,*P*,*T*) conditions, a modified EAM potential, and periodic boundary condition by Zhang et al.^170^ confirmed that a small concentration of interstitial defects exert a powerful effect on the crystal stability through their initiation of collective particle motions that ultimately lead to a breakdown of lattice order. The authors showed that the crystal integrity remains preserved for permutational atomic motions in the form of rings exchanges, but at higher temperatures a topological transition in these exchange motions into linear catenations of particle exchange events occurs. To describe the extent of dynamic heterogeneity, they used the van Hove correlation function *G*(*r*,*t*) = *N*^–1^⟨∑_*i*_δ(*r*_*i*_(Δ*t*) – *r*_*i*_(0) – *r*)⟩. Further, mobile atoms *i* and *j* were considered to be within a collective atom displacement “string” (Figure 11) if they remain in each other’s neighborhood, specified by the proximity relationship min[|*r*_*i*_(Δ*t*) – *r*_*j*_(0)|, |*r*_*i*_(0) – *r*_*j*_(Δ*t*)|] < 0.43*r*_0_, where *r*_0_ is the interatomic distance. For these strings the mean “string length” was defined as *n̅*(Δ*t*) = ∑_*n*_*nP*(*n,*Δ*t*), where *P*(*n*,Δ*t*) is the probability of finding a string of length *n* in time interval Δ*t*. String properties were defined at the characteristic decorrelation time Δ*t* = t* at which the mean string length for *G*(*r*,*t*) has a maximum. The distribution of string lengths appeared to be given by *P*(*n*) ∼ exp(−*n*/*n̅*) to an excellent approximation for all temperatures investigated. Study of the topological transition between strings having an open linear chain and a closed ring showed that both distributions show a weak maximum at the temperature where the average string length exhibits a maximum. For higher temperature, the number of rings remains approximately constant while the number of linear strings strongly increases. As the simulations did not show any obvious evidence of aging effects under the conditions investigated, the authors indicated that this peculiar nonmonotonic variation of the string length with temperature is most likely not an artifact. In the absence of experimental data related to melting, they discussed its relation to the behavior of superionic crystals. By and large these simulations support Granato’s model.

Finally, we note that Konchakov et al.,^171^ using MD simulations for 4000 Al atoms, demonstrated a significant increase in the vibrational entropy of formation of interstitialcies Δ_f_*S* near the melting point *T*_mel_, namely from from Δ_f_*S*/*k* ≅ 3.5 at *T*/*T*_mel_ = 0.65 to about Δ_f_*S*/*k* ≅ 7 near *T*_mel_. The concentration of such defects in the melt, as estimated by three independent methods, turned out to be *c* ≅ 0.08, close to the Granato estimate, from which the configurational component of the entropy of the system with defects was determined. It was found that about 70% of the total entropy of melting, and thus of the melting enthalpy, observed in experiments can be attributed to the generation of interstitialcies at *T*_mel_, and therefore such defects play a significant role in the melting process.

### 5.8 Simulations

Nowadays molecular dynamics (MD) simulation is an illuminating tool resulting in detail that otherwise is hard to obtain. In classical MD simulations Newton’s equations of motion are solved numerically as a function of *T* and *V* or of *T* and *P*. Both equilibrium and nonequilibrium data can be obtained, the latter often by applying a (slowly) varying outside “driving force”. Another simulation method is the Monte Carlo (MC) technique, which essentially probes the configuration space in an efficient statistical way. In its standard form it does not yield the dynamics but was (is) often somewhat more efficient for obtaining equilibrium data. In simulations for bulk materials without surfaces, for both methods generally full periodic boundary conditions are used. Both methods are described in detail in refs (11 and 12).

Simulation methods use an interparticle interaction as a function of distance *r*, often described by the Mie potential ϕ = ε[*n*/(*n* – *m*)](*n*/*m*)^*m*/(*n*−*m*)^[(σ/*r*)^*n*^ – (σ/*r*)^*m*^], more in particular by the Lennard-Jones (LJ) potential ϕ(*r*) = 4ε[(σ/*r*)^12^ – (σ/*r*)^6^]. Here *m* and *n* > *m* are constants, while ε represents the well depth and σ is the diameter of the particle at ϕ(σ) = 0. For simulations often scaled quantities are used, where T* = *kT*/ε is the scaled temperature, P* = *Pσ*^3^/ε is the scaled pressure, ρ* = *ρσ*^3^ is the scaled density, E* = *E*/ε is the scaled energy, and τ* = τ(ε/*mσ*^2^)^1/2^ is the scaled time. More limitedly the Morse potential ϕ = ε{exp[α – *r*/*r*_e_)] – 1}^2^ – ε is used, with again ε the well depth, *r*_e_ the equilibrium distance, and α a dimensionless parameter describing the curvature of the potential near the equilibrium distance. The higher the value of α, the shorter the range of the potential and the steeper the curvature (2*εα*/*r*_e_^2^)^1/2^. In the following we first discuss some general aspects and thereafter a number of studies in some detail.

#### 5.8.1 General Aspects

Clearly the nature of the interaction potential is of influence. The effect of the softness of the interaction potential was discussed by Chakraborty et al.^172^ using MC simulations, Morse potentials, and the LJ potential for benchmarking size effects, following up other studies on Morse solids.^173^ For the Morse potential the parameter α was varied between 4, 5, 6, and 7 to mimic various types of materials, with α = 4 representing long-range and α = 5–7 intermediate-range systems, using 343 particles, a number based on the results for the LJ system. Umbrella sampling^11,12^ was used to determine *T*_mel_ via constructing Landau free energy curves from simulations at constant (*N*,*P*,*T*) conditions. The melting temperature and strength of the discontinuous transition were shown to increase only mildly as the range and softness of the potential decrease, but the change in number density Δρ/ρ_S_ on melting for the solid density ρ_S_ and the entropy of melting Δ_m_*S* increase by factors of about 3 and 2, respectively, by increasing α from 4 to 7. The values of Δρ/ρ_S_ and Δ_m_*S* for α = 6 are very similar to those for the LJ system, but *T*_mel_* = 0.780 (at P* = 0.947ε/*r*_e_^3^) of the latter is much higher than those for any of the Morse systems. Because the exponential decrease of the Morse potential is quicker with distance than the power law decrease of the LJ system, the authors concluded that the transition is largely determined by the behavior of the pair interaction near equilibrium separation. Finally, they also showed that the barrier height *B* separating the phases as determined from the Landau expression increases quite sharply with increasing value of α. The value of *B* is expected to correlate with the interfacial energy γ, for which Turnbull’s estimate γ = 0.3Δ_m_*Hρ*_S_^2/3^ (ref (174), see also ref (175)) was used, where Δ_m_*H* is the enthalpy of melting per particle. A fit *B* = *cγ* with *c* a constant for the Morse and LJ systems (not given) yielded *c* = 1.048 and *R*^2^ = 0.995, showing the good correlation.

Efficiency and accuracy for calculating *T*_mel_ were studied using MD simulations by Zou et al.^176^ for five different methods, namely the hysteresis, the two-phase coexistence, the interface pinning, the thermodynamic integration, and the modified void methods. Calculations were done for Cu (FCC structure) and Ni–Zr (B2 structure) using embedded atom potentials with full periodic boundary conditions at constant (*N*,*P*,*T*) conditions. Typically, the number of particles was between 1 × 10^3^ and 15 × 10^3^. In the hysteresis method *T*_mel_ is given by *T*_mel_ = *T*_+_ + *T*_–_(*T*_+_*T*_–_)^1/2^, where *T*_+_ and *T*_–_ are the temperatures where the volume of the system is discontinuous in a heating cycle and a cooling cycle, respectively. It appeared that the results of the hysteresis method strongly depend on the heating/cooling rates used and was deemed by the authors as not suitable for binary systems. In the two-phase system the atoms in half of the simulation box at a certain temperature *T*_es_ and normal pressure *P*_n_ are fixed, while the other half is heated, thereafter cooled to *T*_es_, and relaxed at constant *P*_n_ and at constant enthalpy. For small systems the two-phase method may result in an anisotropic pressure tensor, while the interface pinning method may result in strong interfacial fluctuations and should be used with care for binary systems. The thermodynamic integration method directly employs the difference in Helmholtz energy Δ*F* between the two phases as calculated from Δ*F* = ∫⟨∂*H*/∂λ⟩dλ with *H* the Hamilton function, λ the integration parameter, and the integral running from λ = 0 to λ = 1. Together with the Gibbs–Helmholtz equation *G*(*P*,*T*)/*T* – *G*_*j*_(*P*,*T*_*j*_)/*T*_*j*_ = −∫⟨*H*⟩_*NPT*_/*T*^2^)d*T* with *H* the enthalpy for constant *(N,P,T)* conditions and the relation for the Gibbs energy *G* = *F* + *PV*, the melting temperature can be calculated. In this case a somewhat involved estimate for Δ*F* was used, for which we refer to the original paper.^176^ The method appeared to be suitable for the monatomic and binary systems used. What the authors called the void method is just monitoring the temperature of a perfect lattice system at constant (*N*,*P*,*T*) conditions where the volume of the system increases suddenly and associating that temperature with *T*_mel_. In their modified void method, an initial perfect lattice in equilibrium is used in which a void is created and thereafter equilibrated at constant (*N*,*P*,*H*) conditions. This will result in a fully solid phase, a fully liquid phase, or, with the proper choice of the enthalpy *H*, solid–liquid coexistence. This modified void method was highly efficient for the monatomic systems but needed more simulation time to reach equilibrium for the binary system. Although the authors did not state the conclusion clearly, and, by the way, the paper is overall difficult to read, it seems that both the thermodynamic integration and the modified void method worked the best.

Further efficiency can be obtained by using the shock melting method,^177^ based on the multiscale shock technique,^178^ in which shock loading is done to drive the simulated system to the final Hugoniot end state. Shocking a sample with the shock velocity *U*, the multiscale shock technique keeps it on both the Rayleigh line *P* – *P*_0_ = *U*^2^[1 – (ρ_0_/ρ)]/ρ_0_ and the shock Hugoniot state *U* – *U*_0_ = 1/2(*P* + *P*_0_)(*V*_0_ – *V*) by applying a uniaxial strain to the computational cell. In such a simulation, with conservation of mass, momentum, and energy, the Hugoniot end state of the simulated system is achieved by adjusting *V* and *T* iteratively, and this end state was shown to be consistent with results of nonequilibrium MD simulations.^177,178^ A comparative study using the shock method and the two-phase method for Au using an embedded atom potential^179^ illustrated that, while obtaining comparable results, the number of atoms to be used in the shock method can be considerably smaller than that used for the two-phase method (as long as one is not interested in the details of the shock wave process itself). For example, in this calculation the two-phase method used 20 736 atoms, while the shock method employed only 640 atoms, nevertheless obtaining virtually the same result. The latter can be judged by comparing the parameters *a* and *b* fitted to the Simon–Glatzel equation (section 7), written as *T*_mel_ = *T*_mel,0_(*P*/*a* + 1)^*b*^, and yielding *a* = 28.35 (1.25) and 22.97 (1.41) and *b* = 0.59 (0.01) and 0.55 (0.01) for the two-phase and shock methods, respectively, with the uncertainties as given by the authors in parentheses.

Finally, we note that, for a method based on two phases, the vibrational density of states of in particular the solid phase must be estimated accurately to obtain the melting entropy.^180^ Problems associated with constant (*N*,*E*,*V*) conditions are discussed in ref (181). The effect of polydispersity for LJ systems was studied by Sarkar et al.,^182^ who showed that a crystalline system cannot be realized above a certain dispersity in size, the critical value of which is temperature and density dependent. This critical value saturates to a value of about 0.11 for all temperatures, in good agreement with the experimental value of 0.12. Further, it was shown that the Lindemann rule breaks down for polydisperse systems,^183^ as the increased root-mean-square amplitude of the smaller particles plays a role in the segregation of them prior to melting, although melting itself remain discontinuous.

#### 5.8.2 Specific Systems

Now we turn to various, more specific simulation views, for which in section 4 we already referred to the MC hard sphere (HS) simulations by Hoover and Ree.^34^ Another early MC result includes the effect of dislocations and disclinations in three-dimensional simple cubic crystals using a lattice with 12^3^ and 20^3^ sites and a partition function based on gauge theory that can represent a dislocation-mediated melting mechanism.^184^ Because of the long-range nature of dislocation interactions, direct numerical analysis are cumbersome, but a dual model yielded a local expression that can be handled with standard MC methods. In this formulation the parameters *x* = (*C*_1111_ – *C*_1122_)/2*C*_1212_ and β = *μa*^3^/(2π)^2^*T* were used with μ = *C*_1212_, λ = *C*_1122_, *a*^3^ the volume per site, and the various *C*_*ijkl*_ the elastic constants. The influence of λ seemed to be minor, and all simulations were done for λ = 0. It appeared that β at melting can be described well by β_m_(*x*) = (1/2)(2/*x*)^α^ with α = 0.597 ± 0.002, while the Lindemann rule would give β_m_(*x*) ∼ *x*^–0.5^. The transition entropy appeared also to be nearly independent of λ. For isotropic crystals (*x* = 1) the transition occurs at β_m_ = 0.76 with an entropy change of about 1.4*k* per site, approximately in agreement with experimental values for a number of metals.^185^ The Lindemann parameter was in approximate agreement with the experimental data,^7^ taken with data from ref (186) as was the specific heat. In a later development^187^ the authors claimed that their lowest-order theory has an average deviation of about 12% for *T*_mel_, which is better by a factor 2 than the 22% of the numbers derived from Lindemann’s rule for 18 metals.

In a fairly detailed study Ahmed and Sadus^188^ used combined equilibrium-nonequilibrium MD simulations and discussed the LJ (*n*,6) system with *n* = 7, 8, 9, 10, 11, and 12 by presenting their phase diagrams and analytical expression for the coincidence pressure, and liquid and solid densities as a function of temperature. A system size of 2048 LJ particles was used for five sets of simulations at various temperatures with a cutoff distance of 2.5σ, all using as initial configuration the FCC lattice (so that a free surface is absent). Conventional long-range corrections were used to recover the properties of the full Lennard-Jones fluid. While for the coexistence pressure and densities for the various *n*-values polynomials in β = 1/*kT* were used, for which the authors provide the relevant coefficients, the triple point (melting temperature) is well represented by *T*_tri_(*n*)* = 2.10/*n* + 0.482 and the triple point pressure is represented by *P*_tri_(*n*)* = 0.1104/*n* – 0.0073. For the (12,6) potential *T*_tri_* = 0.661, a value rather similar to other estimates. It also suggests that, for the Sutherland potential with hard sphere repulsion and *r*^–6^ attraction or the (∞,6) potential, *T*_tri_* = 0.482, to be compared with other estimates of 0.572^189,190^ and 0.607.^191^ Similarly, *P*_tri_(∞)* = −0.0073 suggested that *P*_tri_(∞)* = 0 as *P*_tri_ cannot be negative, and is to be compared with another estimate, *P*_tri_(∞)* = 0.079.^189^

The simulation data were also used to obtain parameters for the Raveché, Mountain, and Streett (RMS) and Lindemann melting rules. The Lindemann ratios obtained, estimated as ξ = (⟨⟨*r*^2^⟩ – ⟨*r*⟩^2^⟩)^1/2^/*a* with *a* the nearest-neighbor distance at T* = 1, were ξ = 0.157 for *n* = 12 and an average value ξ = 0.183 for *n* = 11 to 7 with all individual values rather close to the average. The Raveché, Mountain, and Streett (RMS)^192,193^ criterion uses the ratio *I* = *g*(*r*_min_)/*g*(*r*_max_) and states that at freezing *I* ≅ 0.2. Here *r*_min_ is the position of the first minimum and *r*_max_ is the position of the first maximum of the pair correlation function *g*(*r*). At T* = 1, the *I*-values obtained were 0.14 for *n* = 12 and 11 and 0.13 for *n* = 10, 9, 8, and 7. Clearly, *I* is largely constant but low as compared to the experimental value *I* ≅ 0.2,^193^ and the authors suggest that the difference may partially reflect the inability of the LJ potential to fully reflect the properties of real liquids. Another criterion used is the Hansen–Verlet freezing rule.^194^ This rule is a generalization of the long wavelength limit for the structure factor *S*(*q* = 0) = *ρkTκ*_*T*_, with density ρ, Boltzmann’s constant *k*, and compressibility *κ*_*T*_, as obtained from fluctuation theory to any wave vector *q*. Upon freezing the height of the principal peak *S*(*q*_m_) increases and the criterion states that a liquid will freeze when the quasi-universal value *S*(*q*_m_) = 2.85 is reached, approximately corresponding to the balance between the gain in Helmholtz energy *F* due to volume contraction and the loss in *F* due to the change from a uniform density for the liquid to a periodic density for the crystal. However, from the calculated structure factors for the various *n*-values it appeared that the maximum is *n*-dependent. Therefore, this study showed that the Lindemann rule and RMS rule are obeyed by the (*n*,6) potential, but the Hansen–Verlet criterion is not.

Another fairly detailed paper dealing with the LJ system, authored by Klumov,^195^ uses the conventional MD method for 4000, 2048, and 1372 LJ particles at constant (*N*,*V*,*T*) conditions with periodic boundary conditions. The author describes a range of indicators for melting, such as the RDF, the RMS characteristic *I* or *I*^–1^, and various invariants of various distribution functions. A sharp rearrangement of the radial distribution function (RDF) with a loss of long-range order occurs at the transition temperature, for ρ* = 1 given by T* ≅ 1.65. Possibly the clearest indicator given is the angular distribution for the 12 nearest neighbors in the (ϕ,θ) plane with ϕ and θ the polar and azimuthal angles, just below the transition (T* ≅ 1.65) and just above the transition (T* ≅ 1.66). While the distribution for the solid-like phase at T* ≅ 1.65 clearly shows a regular arrangement corresponding to the FCC lattice, for T* = 1.66 the distribution is close to uniform, representing the melt. The parameter *I*^–1^ also shows a strong decrease, in this case at about T* ≅ 1.62–1.63. Another rather clear indicator, advocated by the author, is *w*_6_* = *w*_6_/*w*_6_^FCC^, where *w*_6_ is the third-order rotational invariant of the angular distribution *q*_*lm*_(*i*) = *N*_NN_^–1^∑_*j*_*Y*_*lm*_(*θ*_*j*_,*ϕ*_*j*_) with *Y*_*lm*_(*θ*_*j*_,*ϕ*_*j*_) the spherical harmonics and the sum over *j* runs over the number of nearest neighbors *N*_NN_ of particle *i* (*N*_NN_ = 12 for FCC). The value *w*_6_* rises strongly from about 1 for the solid state to about 3 for the liquid state at T* ≅ 1.62–1.63. The author does not comment on the (small) differences in T* obtained for various indicators but does point out that the value for *w*_6_* can be obtained from just a few snapshots, while the root-mean-square displacement, as used by the Lindemann rule, typically requires more data.

Costigliola et al.^196^ indicated the invariance of several properties of the LJ system along the melting and freezing lines can be interpreted in terms of isomorph theory, as introduced in ref (197) and elaborated in ref (198). That theory considers what were originally called “strongly correlating liquids” but now “Roskilde simple” liquids, i.e., liquids where the virial  and potential energy *Φ* in simulations for *N* particles at density ρ and temperature *T* have a correlation coefficient  > 0.9 for the thermal equilibrium fluctuations ⟨···⟩ in pressure *P* and internal energy *U* at constant (*N*,*V*,*T*) conditions. According to this theory structure and dynamics are invariant to a good approximation along constant excess entropy curves. Such curves were designated as “isomorphs” and offer the possibility to explain some but not all melting/freezing invariants without reference to the actual mechanisms at melting/freezing process itself. The theory revived the idea by Hoover,^27^ Ross,^58^ and Kuramoto^59^ that a configuration ***R***_1_ and a configuration ***R***_2_, where ***R*** denotes the collective of all coordinates, obey *P*(***R***_1_) = *P*(***R***_2_) when ρ_1_^1/3^***R***_1_ = ρ_2_^1/3^***R***_2_. Here *P*(***R***) is the Boltzmann probability of configuration ***R*** of what was called an “isomorph”. As indicated, such a condition can be exactly valid only for Euler homogeneous potential energy functions, like the inverse power laws, but is approximately true for systems dominated by repulsive interactions for which it may be assumed that power law density scaling reflects an underlying effective power law potential.

For such an isomorph the excess entropy *S* and the isochoric specific heat *C*_*V*_ are invariants. Briefly, the reason is that *S* is determined by the canonical probabilities, which are identical for scaled microconfigurations of two isomorphic state points. From Einstein’s formula *C*_*V*_ = ⟨Δ*U*^2^⟩/*kT*^2^ the isomorph invariance of *C*_*V*_ follows by taking the logarithm of the Boltzmann probability and using the isomorph invariance of scaled microconfiguration probabilities. Since *S* and *C*_*V*_ are invariant along the same curves in the phase diagram, *C*_*V*_ = φ(*S*). Thus, *T*(∂*S*/∂*T*)_*V*_ = φ(*S*) or at constant volume d*S*/φ(*S*) = d*T*/*T*. Integrating this leads to an expression of the form ψ(*S*) = ln(*T*) + *k*(*V*), which implies *T* = exp[ψ(*S*)] exp[−*k*(*V*)], or generically using *s* ≡ *S*/*N*, *T* = *f*(*s*) *h*(ρ), indicating a separation between *s* and ρ. For inverse power law interactions, the entropy *S* = *K*(*ρ*^*γ*^/*T*) is a function of *ρ*^*γ*^/*T* where γ = *n*/3. Applying the inverse of the function *K* shows that these perfectly correlating systems obey *T* = *f*(*s*)*h*(ρ) with *h*(ρ) = *ρ*^*γ*^, so γ = d ln *h*/d ln ρ. An isomorph starting from a reference state (ρ_0_,*T*_0_) to another state (ρ,*T*) can be constructed using *h*(ρ)/*T* = *h*(ρ_0_)/*T*_0_. For inverse power laws one can obtain *h*(ρ) = ∑_*n*_*α*_*n*_(ρ/ρ_0_)^*n*/3^, where *n* represents the various exponents, so for the LJ potential *h*(ρ) = (1/2γ_0_ – 1)(ρ/ρ_0_)^4^ – (1/2γ_0_ – 2)(ρ/ρ_0_)^2^ results. Here γ_0_ is the density-scaling exponent which can be obtained from γ_0_(ρ_0_,*T*_0_) = ⟨Δ*V*Δ*U*⟩/⟨Δ*U*^2^⟩|_(ρ_0_,*T*_0_)_. From the internal energy *U*_0_ = ∑_*n*_*U*_*n*,0_ at ρ_0_, one obtains at a new density ρ = (ρ/ρ_0_)^−1/3^, *U*(ρ) = ∑_*n*_*U*_*n*,0_*(ρ/ρ_0_)^*n*/3^. This *U*–*U*_0_ plot for a range of densities ρ yields a linear plot for an isomorph with slope γ_0_.

For the freezing simulations the authors used 1000 particles with a shifted LJ potential having a cutoff at 2.5σ. The simulations using the starting point (ρ*,T*) = (1.132,1.0) yielded γ = 4.9079 and *R* = 0.9955, leading to the correlation *T*_mel_(ρ*)* = Aρ^*4^ – Bρ^*2^ with *A* = 2.27 and *B* = 0.80. In scaled units the radial distribution functions as calculated along the freezing line mapped on a single master curve. This implies that also the structure factor is invariant and that the Hansen–Verlet criterion for freezing is fulfilled.

Limiting the further discussion to melting, the authors performed MD simulations on FCC solids using 4000 particles with a shifted LJ potential having a cutoff at 2.5σ. The simulations yielded γ = 4.8877 and *R* = 0.9985, leading to the correlation *T*_mel_(ρ*)* = Aρ^*4^ – Bρ^*2^ with *A* = 1.76 and *B* = 0.69. In these simulations to calculate the melting isomorph, the starting point was (ρ*,T*) = (1.132, 2.0) and the interface pinning method was used to determine *T*_mel_*. The *T*_mel_*–ρ* curve is thus well-described but underestimates *T*_mel_* at low density and overestimates *T*_mel_* at high density somewhat. Further, it appeared that the mean square displacement is constant and for *T*_mel_* > 1.8 becomes density independent. Hence, also the Lindemann rule is obeyed.

Focusing on bulk melting and using the energy landscape for Cu and Al, Samanta et al.^199^ employed an embedded atom method (EAM) potential and simulations using a sample size of 32 000 atoms (20 × 20 × 20 cells) and 256 000 atoms (40 × 40 × 40 cells). The results showed that in these cases melting occurs via multiple, competing pathways involving the formation and migration of point defects and dislocations. Each path is characterized by multiple barrier crossings arising from multiple metastable states for the solid. At temperatures approaching superheating, melting becomes a single-barrier process, while at the limit of superheating, the melting mechanism is driven by a vibrational instability. Comparing their results with nucleation theory, the authors suggest that classical nucleation theory for melting should be revised.

A detailed analysis of homogeneous melting in crystalline materials modeled by empirical interatomic potentials for MD simulations at constant (*N*,*P*,*T*) conditions and using the theory of inherent structures was presented by Nieves and Sinno.^200^ The authors showed that homogeneous melting of a perfect, infinite crystalline material can be inferred directly from the growth exponent of the inherent structure density-of-states distribution, expressed as a function of formation enthalpy. The presence of only a very few homogeneously nucleated point defects in the form of Frenkel pairs was established to be required and supports that homogeneous melting can be appropriately defined in terms of a one-phase theory. The effect of an applied hydrostatic compression *P* on homogeneous melting showed that the inherent structure analysis used was able to capture the correct pressure dependence for crystalline Si and Al, whereby the coupling between *T*_mel_ and *P* arises through the distribution of formation volumes for the various inherent structures.

Other papers dealing with individual metals and aspects are, e.g., on Si^201^ emphasizing the effect of premelting, on U^202^ using classical and quantum MD methods, and on W^203^ dealing with the effect of applied stress anisotropy.

While most papers focus on relatively simple models and monatomic systems, some papers deal with more complex constituents. Zheng et al.^204^ discussed the melting of nitromethane for 240 nitromethane molecules (1680 atoms) at constant (*N*,*P*,*T*) conditions using a potential that contained a Morse bonding term, an harmonic term for angle bending, a dihedral term for internal rotation, an exp-6 term for nonbonding interactions, and an electrostatic term. The potential was cut off at 10 Å for the van der Waals interaction, while the electrostatic interaction was calculated using Ewald summation. Using the hysteresis method, they obtained *T*_mel_ = 251 K, in excellent agreement with *T*_mel_ = 255 K by Agrawal et al.^205^ and the experimental *T*_mel_ = 245 K. In the melting process, the nitromethane molecules begin to rotate about their lattice positions in the crystal, followed by translational freedom of the molecules. The critical values of the Lindemann parameter for the C and N atoms immediately prior to melting were found to be around 0.155 at 1 atm. The intramolecular motions and molecular structure of nitromethane undergo no abrupt changes upon melting, indicating that the intramolecular degrees of freedom have little effect on the melting.

Finally, it is probably fair to say that the main concern of simulation studies is often of a rather general character dealing with energy and entropy. There are, however, exceptions analyzing the results also in terms of mechanisms. To do so, one of the main problems is to use a characteristic that properly discriminates between the configuration relevant for a certain mechanism and other configurations.

### 5.9 Models and Correlations

Apart from simulations other approaches exist which we mention here. They comprise solid and liquid state modeling, both simple and more complex using a limited number of parameters, and correlations. We discuss them in sequence.

As lattice models play(ed) a significant role in solid–liquid modeling, we start with these. In conventional lattice models the lattice is incompressible, but in an approach by Mori et al.^206^ the lattice is taken compressible, and with an approximate method of taking into account the correlation between the intracellular configurations of molecules, it was found that the hard sphere transition and the triple point of argon could be reproduced fairly well. Despite the fair results obtained, the authors note that their lattice model does not describe the molecular structure of the liquid properly, so the theory is not satisfactory in this respect. Moreover, in the lattice approach long-wavelength fluctuations, which do play a role in the S–L transition, are difficult to take into account.

In a simple approach Kozlovskiy^207^ revived and refined an about a century old idea by Boguslawski, essentially describing melting with an anharmonic oscillator, considered to represent noncoupled, anharmonic oscillations for atoms. With the use of the equations of motion, the mean position and displacement were calculated and used in the Gibbs energy developed in a power series up to the fourth degree. As possibly expected, the results show that at a certain temperature the atom escapes from the well, which is associated with melting. The influence of pressure and the model dimension were discussed. The model predicts that at the beginning of melting about a tenth of the atoms escape from their regular positions.

In a more sophisticated approach by Stroud and Ashcroft,^208^ the Gibbs energy *G* for both the solid and liquid states was calculated. For the solid state they considered *G* to be composed of the internal energy *E* of the static lattice plus the additional Gibbs energy associated with the excitation of phonons at finite temperatures. The internal energy *E* was considered to be the sum of (a) the kinetic, exchange, and correlation energies of the electron gas *E*_eg_; (b) the Madelung energy arising from the Coulomb interaction between the ions *E*_M_; and (c) an additional reduction in energy arising from the redistribution of the electron gas in the presence of the attractive electron–ion interaction or the band-structure energy *E*_BS_. In the relevant expressions for the Madelung and reduction energies a Debye–Waller type of exponential was added to describe the thermal influence on the lattice potential. The vibrational contribution was calculated using self-consistent phonon theory (SCPT) in which the vibrational density of states was approximated by a Debye distribution. This implies that all effects at given *T* and *V* are included in Debye temperature. For the liquid state the same terms are used, but as the summations run over the atomic positions, they cannot be made without knowing the pair correlation function *g*(***r***) or its Fourier transform, the structure factor *S*(***k***). For *S*(***k***) the hard sphere structure factor was taken as calculated in the Percus–Yevick approximation. The liquid Gibbs energy is thus the electron gas terms, as in the solid, plus the potential energy terms, calculated using the hard sphere *S*(***k***), plus the kinetic energy of the ions, plus the entropy term, taken as the entropy of the hard sphere gas in the Percus–Yevick approximation.

The approach yielded a volume- and temperature-dependent effective Debye temperature for the solid and an effective volume- and temperature-dependent hard sphere packing fraction for the liquid. Thermodynamic quantities were computed for both phases and along the melting curves appeared to be in good agreement with available experiment. Lindemann’s law is fairly well obeyed in the solid phase, although not perfectly, and its analogue (i.e., constant hard sphere packing fraction along the melting curve) holds in the liquid.

Matsuura et al.^209^ indicated that the Stroud–Ashcroft calculation is suitable only for other alkali metals since free electron properties were fully taken into account. They constructed a melting model based on vanishing of the velocity of the transverse phonons in a self-consistent harmonic approximation (SCPT) using the nearly free electron model for the conduction electrons. Despite the general doubt on the applicability of the (shear) lattice instability (section 5.3), the authors offer no argument for this. Their calculations resulted in *T*_mel_ = 0.145ℏ^2^*n*_c_/km*R_d_^2^, where m* and *n*_c_ are the effective mass and number of conduction electrons per site, for which the authors indicate that this result is on the order of the Fermi temperature ℏ^2^/km*R_d_^2^ ∼ ℏ^2^*k*_F_^2^/2km* with Fermi momentum ℏ*k*_F_ and that it does not include the ionic mass. The melting temperatures agree approximately with experimental values for alkali and noble metals with *T*_mel,calc_ = *cT*_mel,exp_ with *c* = 1.025 and *R*^2^ = 0.953 (not given), while the Lindemann parameter was estimated as 0.183 and 0.172 for BCC and FCC lattices, respectively.

Next to models, empirical correlations are still useful and, obviously, easy to use. Reynolds et al.^210^ noted a quite good correlation between the surface energy γ (in mJ m^2^), melting temperature *T*_mel_ (in K), and interatomic distance *r*_0_ (in Å) for metals given by γ = 760 + 4.77*T*_mel_/*r*_0_^2^, based on Gorecki’s correlation for *E*_vac_/*T*_mel_ (ref (113), section 5.4) and Couchman’s correlation for *E*_vac_/γ,^211^ although there is no explanation for the presence of the intercept.

Li and Wu^212^ based an empirical relationship on the “universal” bonding curve^213^ and the Debye model for binary intermetallics with the CsCl structure. The universal bonding model describes the bonding curve by *E*(*r*_WS_) = εE*[(*r*_WS_ – *r*_WS0_/*l*] where *r*_WS_ and *r*_WS0_ are the momentary Wigner–Seitz radius and Wigner–Seitz radius at equilibrium, respectively, and *l* is a scaling constant given by *l* = (ε/12π*r*_WS0_*K*)^1/2^ with *K* the bulk modulus. Supposing that in the Debye model for high temperature the longitudinal speed of sound is given by (*K*/ρ)^1/2^, the root-mean-square displacement is ⟨Δ*u*^2^⟩^1/2^ = (0.8278*kT*/*r*_WS0_*K*)^1/2^, where ρ is the mass density. Further supposing that ⟨Δ*u*^2^⟩^1/2^ = *const*. at *T*_mel_, the relation *T*_mel_ = 0.032ε/*k* was obtained^214^ for pure metals. This rule worked moderately well for the 78 pure metals examined, as testified by the correlation *T*_mel_ = *cε*/*k* with *c* = 0.0332 and *R*^2^ = 0.948 (not given). For the 14 BCC pure metals *T*_mel_ = 0.0355ε/*k* with *R*^2^ = 0.970 was obtained. For intermetallics A_*x*_B_*x*–1_, however, the availability of the experimental cohesive energy data was (is) limited and was estimated by ε = *xε*_A_ + (1 – *x*)ε^B^ + Δ_for_*H*, where Δ_for_*H* is the enthalpy of formation. For 27 congruently melting intermetallics the correlation *T*_mel_ = 0.030ε/*k* with *R*^2^ = 0.876 resulted. However, for (binary as well as ternary) compounds with unknown Δ_f__or_*H*’s an estimate must be used, for which Miedema’s model^215^ can be used to advantage. The authors pointed out that a five-parameter cellular model of artificial neural networks^216,217^ essentially provides the same correlation, but using five parameters instead of one. The correlation was expanded in ref (218) to 143 binary Laves phases with *T*_mel_ = 0.0302ε/*k* and *R*^2^ = 0.899, for which an average predicted error of 14.5% resulted, but reduced to 8% (*T*_mel_ = 0.0326ε/*k* with *R*^2^ = 0.935) if only the C15 crystal structure compounds with congruent melting are considered. The Miedema model was used here as well and was shown to be rather accurate for 13 compounds where experimental values of Δ_f__or_*H* were available.

The effect of orientation for metals was assessed by Chatterjee^219^ on the basis of the work function for the (110), (100), and (111) planes for Cu and Ni and the (100) and (111) planes for Pt. Combining the relation γ^1/2^/ϕ = *const*. between surface energy γ and work function ϕ, the relation γ(*hkl*) = *f*[*m*^1/2^,θ_D,sur_(*hkl*)]^220,221^ with *m* the atomic weight and θ_D,sur_(*hkl*) the surface Debye temperature for *hkl* planes, and the relation θ_D,sur_(*hkl*)^2^ = *f*[*T*_mel_(*hkl*),*m*^–1^,Ω^–2/3^] with *Ω* the atomic volume, one obtains ϕ(*hkl*) = *f*[*T*_mel_(*hkl*)^1/4^,*Ω*^–1/6^]. Taking logarithms the relation ln[ϕ(*hkl*)] = ln[*T*_mel_(*hkl*)^1/4^,*Ω*^–1/6^] – *X* with *X* a constant is obtained, resulting in *X* = 2.97 ± 0.04. Estimating ϕ for Ag(111) and Pb(111) resulted in 468 and 327 kJ mol^–1^, respectively, while ϕ for Ag(11) and Pb(110) yielded 458 and 315 kJ mol^–1^, respectively, comparing closely with the experimental results.

With the use of modern solid-state calculations new correlations have been proposed. Using the earlier proposed concept of condensing potential,^222^ Ye et al.^223^ applied VASP calculations^224^ for a series of metals to the (orientation dependent) escaping potential *P* [eV], defined as the potential for an atom to leave a surface vertically. They showed that a fair correlation for the melting temperature *T*_mel_ is given by
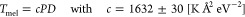
45and *D* [Å^–2^] the surface density of atoms. Whether such a correlation can be obtained for other types of materials has not been investigated.

For organic compounds, group contribution methods to estimate properties have a long history. For the melting points of organic compounds, Yalkowsky and Alantary^225^ provided a group contribution approach based on estimating separately the enthalpy change Δ_m_*H* and the entropy change Δ_m_*S* to obtain *T*_mel_ = Δ_m_*H*/Δ_m_*S*. While Δ_m_*H* is taken to be an additive constitutive property, Δ_m_*S* is not entirely group additive. The latter is primarily dependent on molecular geometry, including parameters which reflect the degree of restriction of molecular motion in the crystal to that of the liquid, and on symmetry, eccentricity, chirality, flexibility, and hydrogen bonding. The authors characterize their approach as a reasonably accurate means of predicting the melting points of 2044 compounds with their melting points ranging from 85 to 698 K and having an average absolute error of prediction of 38.6 K with *R*^2^ = 0.81.

## 6 Thermodynamic or Surface Mediated Melting

In 1935 Peierls^226^ discussed disorder introduced in 1D and 3D lattices by thermal vibrations, followed by discussions on 2D lattices by others.^227,228^ It appears that 1D lattices can have only long-range positional order at *T* = 0 K and that in the thermodynamic limit at any temperature above absolute zero long-range order is destroyed by thermal vibrations. In 3D models, long-range order persists at any temperature, as long as the material is solid. In 2D, possibly expected, long-range order is destroyed by thermal motion at a finite temperature and the mean square displacement grows logarithmically with distance between the molecules, although in view of the weak divergence quasi-long-range order remains. This prompted extensive modeling research on phase transitions in surface films,^229^ which in its turn stimulated both experimental tests and simulations. From this research a 3D melting picture emerged in which surfaces play an important role, as shown, e.g., clearly by van der Veen.^230−232^

The importance of surfaces, already indicated by Tammann^233^ and Stranski^234^ as well as by Frenkel,^111^ is discussed in several reviews.^235,236^ As summarized by Dash,^39^ surface melting is a consequence of wetting of a solid by its melt, occurring when the surface energy of the combined solid–liquid–vapor system is lower than that of the “dry” solid, that is, when γ_SV_ > γ_SL_ + γ_LV_. Although this is usually the case, there are also liquids that do not wet their own crystals, for example, Ga, Hg, *p*-methylaniline, and phenyl salicylate.^237^ The (possibly local) reduction of the surface energy by the liquid renders premelting of the solid energetically favorable. As premelting proceeds, the short-range order of the interface gradually evolves from crystalline order to liquid-like disorder. Approaching the melting temperature *T*_mel_, the disordered or *quasi-liquid* layer grows to a thick film which in its upper layers is indistinguishable from the liquid but retains some solid-like order over a few molecular distances of the solid interface. Premelting does not occur for all surfaces though. For example, for FCC crystals generally the relatively open {110} surfaces do show complete premelting (i.e., δ diverges), while the close-packed {111} surfaces do not show premelting. The {100} surfaces with intermediate packing density show incomplete premelting with a finite thickness (i.e., δ remains finite when *T* → *T*_mel_).

Melting being a discontinuous transition, one expects hysteresis in *T*_mel_ upon heating cooling, i.e., the presence of superheating for the solid and supercooling for the liquid. While the latter effect does occur, the former is absent for most solids. Premelting is instrumental to explaining this absence of superheating as well as the absence of “effective” bulk precursor effects for most solids (see, e.g., ref (7)). To explain this absence, Pietronero and Tosatti^238^ used a simple Einstein model for a cubic lattice with a surface in a self-consistent harmonic approximation (SCPT) for shear displacements parallel to the surface. If at *T* = 0 the force constants *K*_0_’s between nearest-neighbor atoms *n* and *n*′ are taken all the same, at nonzero temperature they become exponentially dependent on the mean square amplitudes of the atoms, *K* = *K*_0_ exp[−λ(⟨*u*_*n*_^2^⟩ + ⟨*u*_*n*′_^2^⟩)], where λ can be calculated from microscopic considerations. Hence, the effective force constants are no longer the same because they depend on the mean square displacements of the atoms that are affected by the surface. Scaling the displacements as *y*(*n*) = λ⟨*u*_*n*_^2^⟩ and the temperature as τ = *kTλ*/*K*_0_, instability for the bulk (*n* = ∞), calculated analytically, occurs at *y*(∞) = τ_B_ exp[*y*(∞)], corresponding to *y*_B_(∞) = 1, τ_B_ = e^–1^ = 0.368, and *K*_B_(∞) = *K*_0_e^–1^. In the presence of a surface, numerical calculations showed that the surface temperature (for the outermost layer) τ_S_ = 0.272, considerably lower than the bulk value. For the next layer a value for the force constant is required, but it is certainly smaller than the value for two layers that are still both solid at τ_S_. Assuming that layer 1 is liquid and layer 2 is solid, τ_2_ was determined numerically as τ_2_ = 0.357, still smaller than the bulk value. Melting is therefore related to the instability of the wet surface. The model explains (1) that surface atoms can have mean square deviations much larger than the value that corresponds to the bulk instability, (2) the possibility of superheating inside the crystal, and (3) the existence of well-defined face-dependent surface precursor effects.

The maximum superheating/supercooling at fixed pressure and overpressurization/overdepressurization at fixed temperature was studied by Luo et al.^239^ with MD simulations using a truncated, smoothed LJ potential for 864 particles, while larger systems were being used for checking the absence of size effects. For a range of (σ,ε) values the maximum superheating/supercooling was quantified and shown to be weakly pressure dependent. The equilibrium value of *T*_mel_ was estimated using the hysteresis method and shown to be a reliable technique in comparison with the much more time-consuming techniques based on solid–liquid coexistence and thermodynamic integration. While the solid–liquid interfacial energy increases with pressure, the Lindemann parameter, here defined as ξ = 2^1/2^⟨*u*_2_⟩^1/2^/(4*Ω*)^1/3^ with *u* the positional fluctuation in the FCC lattice and *Ω* the atomic volume, was predicted to remain constant at about 0.14 at high pressures for the solid at the equilibrium melting temperature.

An early MD simulation by Valkealahti and Nieminen^240^ using the LJ potential with a cutoff distance of 3σ calculated total energies, trajectory plots, mean square displacement functions, diffusion coefficients, vacancy concentrations, and two-dimensional order parameters to analyze premelting. The authors indicate that the (111) surface starts to disorder by vacancy formation, which leads to the premelting of the surface layer far below the bulk melting temperature, and that melting proceeds via a layer-by-layer mechanism, when temperature is further increased, in consonance with the Pietronero–Tosatti model.^238^

Another early paper by Pontikis and Sindzingre^241^ reviewed experimental and computer simulation results on surface roughening and surface initiated melting. From their survey the authors reported that theoretical approaches, MD simulations, and experiments converge to the conclusion that bulk melting is propagated into the solid starting from surfaces, thus preventing superheating effects. They also report that theoretical predictions, based on phenomenological models, indicate the thickness of the liquid layer to be very small (one to five atomic layers) until the temperature reaches values very close to *T*_mel_. Moreover, they conclude that, due to the high (relative) temperatures involved, studies of surface melting are beyond the range of applicability of lattice dynamics.

Trayanov and Tosatti^242^ used a discrete reference lattice, as well as drastic simplifications such as mean-field and free-volume approximations, and developed a lattice theory of surface melting based on minimization of the free energy with respect to two spatially varying order parameters—density and “crystallinity”—for (100) and (110) Lennard-Jones crystal surfaces. It was shown that on the coexistence line a quasi-liquid layer forms on the crystal–gas interface with a triple-point temperature *T*_tm_. When the temperature exceeds *T*_tm_, the crystal melts and there is no crystal–gas interface, but rather a liquid–gas interface, so that *T*_tm_ can be interpreted as *T*_mel_. The thickness of the quasi-liquid layer grows asymptotically as (*T*_tm_ – *T*)^1/3^, in agreement with experiments on Ar films. A change from long- to short-range interparticle attraction reduces the growth behavior to logarithmic, while a switch of the potential tail from attractive to repulsive can block altogether the growth of the quasi-liquid layer. It is further shown that, in cases where no in-plane disorder can arise, no surface melting occurs. Within the model, surface melting is found to be continuous without any singularities below *T*_tm_ in the surface energy, which was explicitly calculated. The decay of the “crystallinity” order parameter at the quasi-liquid–gas interface is predicted to be a “stretched exponential” in the long-range case and a power law in the short-range case.

Finally, we note that premelting in small sodium clusters was dealt with by Hock et al.,^243^ premelting in ionic crystals was dealt with by Matsunaga,^244−246^ superheating in molecular crystals was dealt with by Cubeta et al.,^247^ surface melting within DFT for 2D LJ-like systems was dealt with by Ohnesorge et al.^248^ (see section 10.1, DFT), and premelting dynamics was reviewed by Wettlaufer and Worster.^249^

### 6.1 Melting of Nanoparticles

Surface melting can be explained in a thermodynamic way as follows. For a crystal with melting enthalpy per unit volume *L* and surface area *A* and containing *N* atoms (state 1), the Gibbs energy is given by *G*_1_ = *Nμ*_S_ + *Aγ*_SV_. For a crystal covered with a quasi-liquid layer containing *N*′ atoms (state 2), *G*_2_ = (*N* – *N*′)μ_S_ + *N*′μ_L_ + *AΓ*, where *Γ* depends on the thickness δ of the liquid layer. For δ = 0, *Γ* = γ_SV_ while for δ = ∞, *Γ* = γ_SL_ + γ_LV_, and *Γ* should take an intermediate value accounting for interactions between the two interfaces for a thin layer. Using the *spreading coefficientS* = γ_SV_ – γ_SL_ – γ_LV_, one can write *Γ* = γ_SL_ + γ_LV_ + *S* exp(−δ/ξ). Thus, with Δμ = μ_L_ – μ_S_
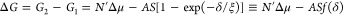
46

Here *f*(δ) = 1 – exp(−δ/ξ) is chosen for short-range interactions (as in metals) with ξ the correlation length. Minimizing Δ*G* with respect to *N*′, meanwhile using *N*′ = *Aρ*_L_δ, yields δ = −ξ ln *t*/λ_sho_ with *t* = (*T*_mel_ – *T*)/*T*_mel_ ≡ Δ*T*/*T*_mel_ and λ_sho_ = −*S*/*ξρT*_mel_Δμ. Here ρ_L_ = ρ_S_ = ρ is assumed. For Δ*T* small, Δμ ≅ −(∂Δμ/∂*T*)Δ*T*. Moreover in equilibrium Δμ = *L* – *T*_mel_(−∂Δμ/∂*T*) = 0, so Δμ = *Lt* and λ_sho_ = *S*/*ξρL*. One can also write δ = −ξ ln[*T*_sur_ – *T*_mel_/(*T* – *T*_mel_)], where *T*_sur_/*T*_mel_ ≅ 1 – (*S*/*ξρL*) signals the onset of surface melting at δ(*T*_sur_) = 0. Therefore, surface melting only occurs when *S* > 0 and the solid is stable without a liquid layer below *T*_sur_. When *T*_mel_ > *T* > *T*_sur_, a liquid surface layer forms with thickness δ, which when *T* → *T*_mel_, diverges; that is, δ → ∞. The logarithmic divergence is consistent with data for metals with ξ ≅ 1.5 monolayer distance, ≅5–6 Å for metals like Al and Pb.^231^ For materials with long-range interaction, such as van der Waals (vdW) crystals, the approximation *f*(δ) = 1 – (ξ/δ)^2^ is more appropriate^250^ and leads similarly to δ = λ_lon_/*t*^1/3^ with λ_lon_^3^ = 2ξ^2^*S*/*T*_mel_ρ(∂Δμ/∂*T*) ≅ 2ξ^2^*S*/*ρL*.^251^

**Figure 12:**
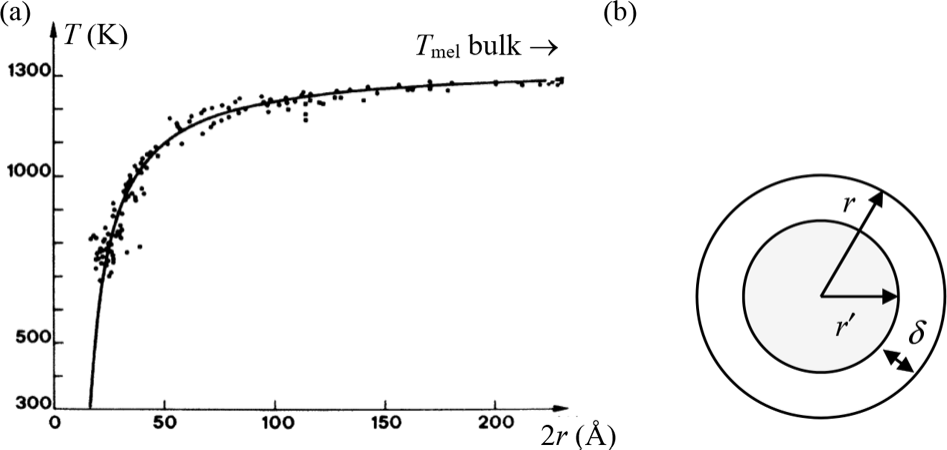
Size dependence of the melting point. (a) *T*_mel_ for Au as a function of the diameter of particles 2*r*. Reproduced with permission from ref (254). Copyright 1976 American Physical Society. (b) Schematic of a solid particle covered with a liquid shell.

A clear indication for the importance of surface melting is the decrease in *T*_mel_ with particle size, predicted by Pawlow in 1909^252^ and first observed by Takagi in 1954,^253^ and for which the example of Au is shown in Figure 12.^254,255^ An extension of the model described above provides a size-dependent *T*_mel_.^256^ The system is supposed to change from a homogeneous solid sphere of radius *r* containing *N* atoms (state 1) to a solid particle of radius *r*′ covered with a surface molten layer with thickness δ containing *N*′ atoms (state 2), so *r*′ = *r* – δ. The Gibbs energy for state 1 reads *G*_1_ = *Nμ*_S_ + 4π*r*^2^γ_SV_, while for state 2 we now have *G*_2_ = (*N* – *N*′)μ_S_ + *N*′μ_L_ + 4π*r*^2^[γ_SL_(*r*′/*r*)^2^ + γ_LV_ + *S*′ exp(−δ/ξ)] with *S*′ = γ_SV_ – [γ_LV_ + γ_SL_(*r*′/*r*)^2^]. With Δ*G* = *G*_2_ – *G*_1_, the result is
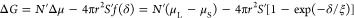
47

This expression generally shows a maximum and minimum, which are interpreted as corresponding to a critical liquid layer thickness δ_cri_ and equilibrium liquid layer thickness δ_equ_, respectively. With increasing *T*, δ_equ_ increases and δ_cri_ decreases, until complete melting occurs when δ_equ_ = δ_cri_. Calculating the minimum of Δ*G*, assuming *L* constant, leads to
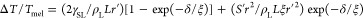
48

For a very small particle size, Δ*G* does not show a minimum, but shows only a maximum which disappears at a certain temperature *T*_0_, suggesting that a solid particle at *T* < *T*_0_ becomes for *T* > *T*_0_ entirely liquid as the barrier height is zero. In other words, the particle above *T*_0_ is liquid always. From this theory, one can estimate an upper bound using δ → 0 (onset instability) as *T*_mel_^(upp)^/*T*_mel_ = 1 – (2γ_SL_/ρ_L_*rL*) as well as a lower bound using Δ*G* → 0 (no driving force) as *T*_mel_^(low)^/*T*_mel_ = [1 – (3/ρ_S_*rL*)][γ_SV_ – γ_LV_(ρ_S_/ρ_L_)^2/3^].^257,258^ It appears that experimental data are limited by these bounds (data not shown). For Pb particles, using ξ ≅ 20 Å, as compared to ξ ≅ 6 Å for a flat Pb surface, the model describes the data well.

Because crystalline nanoparticles inevitably cannot be spheres, the effect of edge and corner atoms was assessed by Shidpour et al.^259^ As expected, their effect is to decrease *T*_mel_ below that of spherical particles that contain only surface atoms. This reduction becomes significant for sizes below 10 nm and is supported by experiments on Au, Sn and Pb.

One might expect that *L* varies with particle size.^19^ Assuming again the two-state model as described above, the volume fraction of molten material is given by *x* = (4π/3)[*r*^3^ – (*r* – δ)^3^] ≅ 3δ/*r* for δ ≪ *r*. For a particle with radius *r*, *L* = (1 – *x*)*L*_S_ + *xL*_L_ = *L*_S_ + (3δ/*r*)(*L*_L_ – *L*_S_), where *L*_L_ and *L*_S_ are the melting enthalpies of the solid and quasi-liquid, respectively. It follows that there is a critical size *r*_cri_ = 3δ(1 – *L*_L_/*L*_S_) for which *L* vanishes. Calorimetric measurements by Sheng et al.^258^ show that (for particles embedded in a matrix) the intercept of a plot of *L*/*L*_S_ versus 1/*r* is ≅1 (as it should be). From the slope (assuming a fixed value δ ≅ 1 nm) the value for *L*_L_ can be estimated. The negative values obtained for *L*_L_ indicate that *L*_L_ cannot be interpreted right away as the pure liquid enthalpy, but that other (structural) effects play a role. Finally, *L* varies not only with *r* but also with *T*. To assess this dependency, an estimate for d*L*/d*T* is needed, but a combined analysis seems not to be available, while the required data are likely unknown. The assumption ρ_L_ = ρ_S_ = ρ is easy to avoid, but ρ_L_ for the quasi-liquid is also typically unknown.

An attempt to describe the size dependence of the cohesive energy was made by Li et al.^260^ using a bond counting model including a correction for relaxation. They based their model on the correlation *T*_mel,0_ = 0.032*E*_0_/*k* given by Guinea et al.,^214^ where *T*_mel,0_ and *E*_0_ = *B*_tot_ε_vol_ are the melting temperature and cohesive energy for bulk material. Here *B*_tot_ indicates the total number of bonds and ε_vol_ is the average bond energy for bulk material. Although there is no clear reason why the same constant should apply, the authors assumed the same relation for a nanoparticle, so the dependence of *T*_mel_(*D*) on the diameter *D* becomes *T*_mel_(*D*)/*T*_mel_ = *E*(*D*)/*E*_0_ = *B*_par_ε_par_/*B*_tot_ε_vol_. Without relaxation ε_par_ = ε_vol_, resulting in
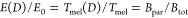
49

They further considered that the energy of a nanoparticle is *E*(*D*) = δ(*E*_0_ + γ) + (1 – δ)*E*_0_ = *E*_0_(1 + *δ γ*) with the surface/volume atom ratio δ = *N*_sur_/*N*, where *N*_sur_ and *N* are the numbers of surface atoms and total atoms, respectively. The surface energy γ was calculated according to γ = −*E*_0_[1 – (*z*_sur_/*z*_vol_)^1/2^] with *z*_sur_ and *z*_vol_ the coordination numbers for surface atoms and volume atoms, respectively.^261^ Hence, assuming *z*_vol,par_ = *z*_vol,bulk_ ≡ *z*_vol_
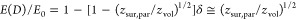
50where the last step can be made for δ → 1. Further, with the number of surface atoms *N*_sur_ = *δN* and the number of volume atoms *N*_vol_ = *N* – *N*_sur_ = (1 – δ)*N*, the ratio *B*_par_/*B*_tot_ = (*N*_sur,par_*z*_sur,par_ + *N*_vol,par_*z*_vol_)/*Nz*_vol_ becomes
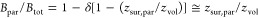
51with the last step again for δ → 1. Therefore, identifying for a nanoparticle with δ → 1, the ratio *B*_par_/*B*_tot_ with *z*_sur,par_/*z*_vol_ results in
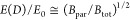
52

So far, relaxation was neglected, and the authors suggested that an approximate way to take this into account is using the average of eqs 49 and 52.
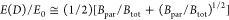
53so that if *B*_par_/*B*_tot_ is known, *E*(*D*) and therefore *T*_mel_(*D*) can be estimated. Clearly, although not unreasonable, eq 53 is an arbitrary assumption. Further, they argue that a suitable estimate for the shape of a nanoparticle is the cuboctahedron, for which *B*_par_/*B*_tot_ was determined by Mirjalili and Vahdati-Khaki^262^ as
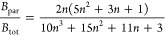
54where *n* is the number of shells around the central particle, related to the diameter *D* = *h*(1 + 2*n*) with *h* the interatomic distance. For other polyhedra, such as the icosahedron, this ratio was also calculated, but their numerical values do not differ greatly. As indicated by the authors, their model, being based on the results of Guinea et al.,^214^ is a one-phase model, while the effect of defects (vacancies) is not considered.

With the use of bulk values for *h*, reasonably good agreement with the experimental data for Al and simulation data for Ar was obtained, but the agreement decreases for Au and even further for Pb. For Al the authors also compared their results with results calculated from a model of Attarian Shandiz et al.,^263^ showing considerable disagreement. Both papers mentioned also discuss *T*_mel_ based on bond counting but with different models. Attarian Shandiz et al.^263^ use a model based on the average coordination number, while Sun et al.^264^ employ a cohesive plus vibration energy equilibrium model. For Al Sun et al.^264^ themselves show in their paper good agreement with experimental results, the discrepancy with the Li et al. results^260^ being due to using different experimental data sets.

Other types of models for discussing melting behavior of nanoparticles exist. A Landau-type model was presented by Chernyshev,^265^ Xue et al.^266^ provided a model based on pressure differences for curved surfaces, and Liu et al.^267^ discussed *T*_mel_ based on model using the Lindemann rule and a thermal phonon contribution.

Clearly, simulation studies on individual nanoparticles also have been conducted. These studies resulted in papers using a generic LJ potential, e.g., refs (268−270); and papers on metal clusters, e.g., Na,^271^ Co,^272^ and Cu;^273^ on alloys, e.g., Li–Cu^274^ and Au–Ag;^275^ on inorganics, e.g., GaN;^276^ and on organics, e.g., benzene, chlorobenzene, heptane, and naphthalene.^277^

The studies mentioned above all use conventional equilibration procedures. Hou^278^ considered that a “real” atmosphere, such as gases at low pressure in which heat transfer is different from the usually assumed energy and volume exchange mechanisms by Nosé and Andersen, is relevant and used a simplified Langevin model yielding d*T*_ato_/d*t* = α(*T*_ato_ – *T*_ele_). In here *T*_ato_ is the atomic temperature, *T*_ele_ is the electronic temperature assumed constant, and α is a constant that within the Sommerfeld theory of metals is given by α = θ_D_*T*_ele_*Lne*^2^*kZ*/2*m*_ele_*κE*_F_ with θ_D_ the Debye temperature, *L* the Lorenz number, *n* the electron density, *m*_ele_ the electron mass, *Z* the valency, κ the thermal conductivity, and *E*_F_ the Fermi energy. With this heat transfer mechanism, the author studied the melting and solidification of metallic nanoparticles of Co, Ni, Pd, Pt, Cu, Ag and Al with an FCC structure in their solid states by MD simulations for 512–12 934 atoms using an embedded atom potential. Caloric curves were constructed starting with low-temperature truncated octahedral particles and applying a heating and cooling cycle. The profiles of these curves were similar for the metals studied and showed two structures, associated with melting in the heating branch and solidification in the cooling branch, taking place at temperatures differing by up to several hundred kelvin. Melting was found to occur via nucleation at the surface for which a state could be identified, suggested to be metastable, having a liquid shell coexisting with an inner crystalline region. Consistently, the melting temperature scales with the surface-to-volume ratio. Solidification was found to occur via nucleation close to the center of a particle and propagating toward the surface. The mechanism is not the reverse of the melting mechanism as several solid seeds may emerge simultaneously at any location in the particle, including the surface, and grow according to a pattern of spinodal decomposition. Depending upon their relative orientations, these seeds coalesce or form coherent interfaces, which were stable over the MD simulation time and resulted in a polycrystalline particle. It appeared that the time needed for the liquid–solid transition to occur was size-independent, which is consistent with a discontinuous transition. It also appeared that solidification was accompanied by a large, sudden configurational energy release. For isolated particles this energy results in a large increase in temperature. Solidification is therefore to occur when the temperature reached is lower than the temperature at which melting is triggered, allowing establishment of a simple relationship between the melting and solidification temperatures that involves the latent heat of fusion and the heat capacity of the liquid. When the configurational energy is released in the usual way to a thermal bath, the solidification temperature is not significantly different, indicating that the activation energy for spinodal decomposition is not sensitive to the presence of a thermal bath, consistent with the scenario that assumes undercooling is mainly determined by the latent heat of fusion.

The possible impact of machine learning and data-driven simulation and characterization on such simulations was shown Zeni et al.^279^ These authors provided transferable machine learning force fields for Au nanoparticles based on data gathered from DFT calculations. These force fields were used in MD simulations to investigate the thermodynamic stability of 1–6 nm Au nanoparticles containing up to 6266 atoms with the solid–liquid phase change in mind, showing melting temperatures in good agreement with available experimental data. The solid–liquid phase change mechanism was characterized employing an unsupervised learning scheme to categorize local atomic environments, thereby providing a data-driven definition of liquid atomic arrangements in the inner and surface regions of a nanoparticle, showing that melting initiates at the outer layers. Another aspect is that, as for bulk solids, the melting of nanoparticles is influenced by impurities. Mottet et al.^280^ showed by MD simulations that a single Ni or Cu impurity in Ag icosahedral clusters considerably increases *T*_mel_ even for sizes of more than 100 atoms. The authors consider that such a small central impurity causes a better relaxation of the strained icosahedral structure, which becomes more stable against thermal disordering.

Related to simulation studies are the discussions given by Berry^281−284^ on the structure of small clusters and the relation to freezing and melting. Also related to simulations is the perspective on freezing and melting by Oxtoby,^285^ emphasizing that small changes in the potential can change the symmetry of the crystal, quoting as an example the crystallization of LJ particles to the FCC structure, but failing to show the FCC–HCP transformation at low temperature. Further, the transition of a homogeneous fluid to a crystal for atomic systems was discussed in DFT terms (see section 10.1, for reviews, see refs (286−288)). Results using a truncated expansion of the Helmholtz energy *F* in the local density to second order, the “perturbation” approximation, and one version of the “weighted density” approximation,^289^ with a nonlocal density functional reproducing the direct correlation function, were dealt with. The former approximation predicts the phase line between solid and liquid fairly well for LJ systems as compared to MD simulations, in spite of the large local density at a lattice site as compared with the density in a homogeneous fluid. The same is true for the latter approximation, which also yields a nearly constant Lindemann parameter of ≅0.12–0.13 along the whole S–L coexistence line. For the L–G transition the density difference between both phases for the former approximation is too large, but the latter approximation deals with that also quite nicely.^290^

To conclude this section, we note and illustrate a few general aspects, the first being the variability of *T*_mel_ for really small particles. As for clusters and nanoparticles there is inevitably a size distribution, for their melting transition does not occur at a sharp temperature but in a certain temperature range of solid–liquid coexistence. For example, for ionized Na clusters containing 70–200 atoms, the melting points are on average 33% (120 K) lower than for the bulk material. Furthermore, variations in *T*_mel_ as large as 630 K were observed with changing cluster size, rather than any gradual trend.^291^ Another study on Na clusters containing about 50–360 particles shows maxima in energy and entropy change upon melting, and modulation of the photoelectron spectra, that were interpreted as being due to geometrical shell closings.^292^ The entropy change of melting, calculated from a simple hard sphere model that assumes that atoms in incomplete outer layers are mobile at least down to 20–30 K below *T*_mel_, was in good agreement with the experimental data. The authors concluded that Na clusters do show magic numbers of electronic origin in general, but that the thermodynamic properties near *T*_mel_ seem to be governed by geometric shell closings, thus showing two completely different kinds of magic numbers, depending on the property studied. Still another example is that for 2–5 nm Au particles supported on carbon films direct TEM evidence of a core–shell structure^293^ was given, while the particles show evidence of size-dependent melting point suppression. The core melting temperatures are significantly greater than predicted by existing models for free clusters. Large-scale ab initio simulations to investigate the influence of the support were done, showing good agreement with experiment. A similar result was obtained earlier by van Hoof and Hou.^294^

Hence, one should distinguish clearly between a scalable regime, where melting is described by power law expressions such as Pawlow’s law, and a small-size nonscalable regime, where melting temperatures vary irregularly and very strongly with size and composition, as discussed above.

The second general aspect is that there is a variety of possible premelting phenomena, from isomerization to surface melting, to two-stage melting and freezing in unary as well as binary systems. For example, tin cluster ions with 10–30 atoms remain solid at about 50 K above the melting point of bulk tin,^295^ possibly related to the fact that the structure of the clusters is completely different from that of the bulk element. Also Huang and Balbuena^296^ showed a two-stage process for bimetallic Cu–Ni 343- and 1000-atom nanoclusters of compositions Cu_0.25_Ni_0.75_ and Cu_0.5_Ni_0.5_ by MD simulations using the Sutton–Chen many-body potential. A similar two-step mechanism was shown by Nelli et al.^297^ for the solidification of AgCo, AgNi, and AgCu nanodroplets in the size range of 2–8 nm by MD simulations. Another important phenomenon is that for supported nanoparticles the melting temperature strongly depends on its wetting angle, hence the substrate.^298^ Moreover, the interpretations of experimental results obtained for clusters or nanoparticles given by different authors do not always agree; see, e.g., ref (299) and the follow-up discussion.^300^

Finally, it appears that for small clusters of a few hundred atoms, in experiments as well as simulations, melting does not occur, as is often assumed, by a surface-mediated mechanism, but rather show a dynamical and changing coexistence between different phases. A few examples will suffice to illustrate this. For Ar such a type of mechanism was suggested by Smirnov.^112^ Matsuoka et al.^301^ showed for 79 atom Ar clusters studied by MD simulations that the cluster exhibits a “dynamical coexistence” of solid and liquid states over an intermediate range of total energy, in which the cluster fluctuates between solid and liquid states. The authors proposed that, for medium-sized clusters, the existence of low-energy solid and high-energy liquid structures leads to this dynamical coexistence, which they considered as a finite-size effect of a bulk melting transition. A somewhat similar, but differing in details, scenario was given by Cleveland et al.^302^ for Au_75_, Au_146_, and Au_459_ clusters, also using MD simulations. Experimentally, the dynamical behavior for 5 nm Pb particles embedded in silica has been observed by high resolution electron microscopy by Ben-David et al.^303^ Spontaneous structural fluctuations between various orientations, with preferred angular changes as measured by the angle change between succeeding configurations of the ⟨111⟩ atomic planes, were observed. Clear transitions involving the vanishing and appearance of twins were detected and twin related transformations, in which the particles rotate by a few degrees, gave a good fit to the observed angular correlation, which excludes complete particle melting during the transition between successive configurations. The authors attribute the instability phenomenon of small metallic particles to the existence of two time scales in the system: a long one, during which the structure is crystalline and stable, and a short one, during which the structure undergoes a fast transition. Moreover, the observed memory effect after transition of the original crystalline orientation is not compatible with complete melting of the cluster. Further examples are the 0.1–10 nm thick discontinuous In films formed by evaporation on amorphous silicon nitride, as investigated by an ultrasensitive thin-film scanning calorimetry technique by Zhang et al.^304^

Similar effects were shown for binary systems. Kuntová et al.^305^ predicted by MD simulations using a many-body tight-binding potential that core–shell Ag–Ni and Ag–Co nanoclusters having the anti-Mackay icosahedron structure are especially stable for those compositions at which the external shell is completely made of Ag, while the inner core is either made of Ni or Co. The simulations clearly show that the external one-layer thick Ag shell melts first, while the inner core is still solid, whereafter the whole cluster melts at a temperature that can be considerably higher than the *T*_mel_ of the external shell, with the width of the temperature interval in which the shell is melted while the core is still solid strongly depends on the system. Pavan et al.^306^ showed by combination of CALPHAD calculations and MD simulation for CuPt nanoparticles containing up to 1000 atoms (or about 3 nm) that the morphology adopted by the nanoparticles causes the icosahedral CuPt particles to melt at temperatures 100 K below that of the other morphologies if the Pt concentration is less than 30%. Settem et al.^307,308^ studied, by parallel tempering MD simulations complemented by harmonic superposition approximation calculations and global optimization searches, for Au_90_, Au_147_, and Au_201_ clusters the equilibrium structures in the whole temperature range from 0 K to *T*_mel_. The results reveal several temperature-dependent structural motifs in these Au clusters. The most important conclusion is possibly that the equilibrium structures at finite temperature cannot be predicted on the basis of the global minimum alone, even below room temperature.

To conclude, we note that, as for bulk solids, homogeneous melting can be initiated from the interior with high heating rates. Chen et al.^309^ showed for Au nanoparticles that melting can start from the surface with the formation of a usual premelting layer under conditions of a suitable particle size and a sufficiently fast heating rate, but that the premelting layer does not extend to the interior under certain conditions. Instead, liquid nucleation occurs in the core of the nanoparticle. This unexpected interior melting is connected to the slower melting kinetics related to heat transfer near the premelted surface.

Nevertheless, despite all the differences, the trend and overall agreement with experiments as predicted with various models are quite good, considering all the experimental difficulties. The above discussion, however, indicates that care must be exercised when either comparing different results or using a model-based estimate. Krishna Goswami and Nanda^310^ reviewed thermodynamic models for size-dependent melting of nanoparticles, as did Hasa et al.,^311^ while Ganguli^312^ provided a brief, narrative summary of the effect of surfaces and size on melting. The effect of size and temperature on vacancy concentration in nanomaterials was discussed by Goyal and Goyal^313^ based on what they call the “Jiang” model,^314,315^ which is actually due to Shi.^316^ Karasevskii and Lubashenko also discussed the melting of rare gas crystals^317^ and nanocrystals^318^ based on their self-consistent statistical method.^319^ Discussions on other aspects of nanoparticles, such as superheating, either when embedded in a matrix or covered with an (insoluble) layer, and the effects of shape, size distribution, and applied stress are available.^21,320^ The thermodynamics of nanoalloys have been reviewed by Calvo^321^ as well as by Guisbiers.^322^

### 6.2 Vacancies Revisited

A relatively simple, but attractive model by Mei and Lu^320^ for thermodynamic melting (of metals) assumes that vacancies are the most probable defects responsible for melting, uses the correlations as given by Gorecki,^113^ and tries to clarify why there is a critical vacancy concentration *c*_cri_ ≅ 0.1, why and how the lattice becomes unstable at *c*_cri_, and how surface (pre)melting is related to vacancy concentration and migration.

Recalling from section 5.4 that the vacancy concentration in the bulk of the lattice *c*_lat_ is given by *c*_lat_ = exp(4.1 – *E*_lat_/*kT*), and assuming a similar relation for the vacancy concentration at the surface of the lattice *c*_sur_, estimates for *E*_lat_ and *E*_sur_ are needed. Note that we denote the *vacancy* energy (*E*_vac_ in section 5.4) here by *E*_lat_ as we need values for the energy of vacancy formation in the bulk *E*_lat_ and at the surface *E*_sur_. To estimate *E*_sur_, one considers that the vacancy formation energy is related to the bonding energy of the atoms. While for FCC and HCP metals one has 12 nearest neighbors in the bulk, at the surface there are only eight. Hence, it is reasonable to estimate that *E*_sur_ = 2*E*_lat_/3. One might expect for FCC(111) nine nearest neighbors in the surface leading to a factor 3/4, but Gorecki used eight (possibly with FCC(100) in mind), and this was taken over by the authors of ref (320). The estimate 3/4 instead of 2/3 will decrease the correlation somewhat. It is known already that *E*_lat_/*T*_mel_ = 80.4 J K^–1^. Therefore, taking Al (*T*_mel_ = 1235 K) as an example, at *T*_mel_ one has *c*_lat_ ≅ 0.0033 and *c*_sur_ ≅ 0.086. In fact, it appears that *c*_sur_ ≅ 0.1 considering all metals for which reliable data are available. The doubt about the “universal” value of the bulk concentration *c*_lat_ ≅ 0.0037 (section 5.4) being too small to induce a lattice instability may thus be answered by considering the surface concentration *c*_sur_ ≅ 0.1, comparable to the concentration increase Δ*c* upon melting. Defining *T*_0.1_ as the temperature at which *c*_sur_ reaches the critical value c* = 0.1, it appears empirically that *T*_0.1_ = *T*_mel_, valid for the FCC, HCP, and BCC metals considered. Hence, close to *T*_mel_, although the bulk is still a crystal, the surface is already molten. The authors^320^ state that the vacancy formation energy at the solid–liquid interface and solid–air interface are similar, but this is arguable in view of the similar solid and liquid densities. Nevertheless, assuming that the vacancy formation energy at the solid–liquid interface is comparable to *E*_sur_, once surface melting has occurred, additional vacancies will be formed at the interface until the whole crystal is molten. This answers the question as to how and why *c* increases from *c* ≅ 0.0037 in the solid state to *c* ≅ 0.1 in the liquid state.

**Figure 13:**
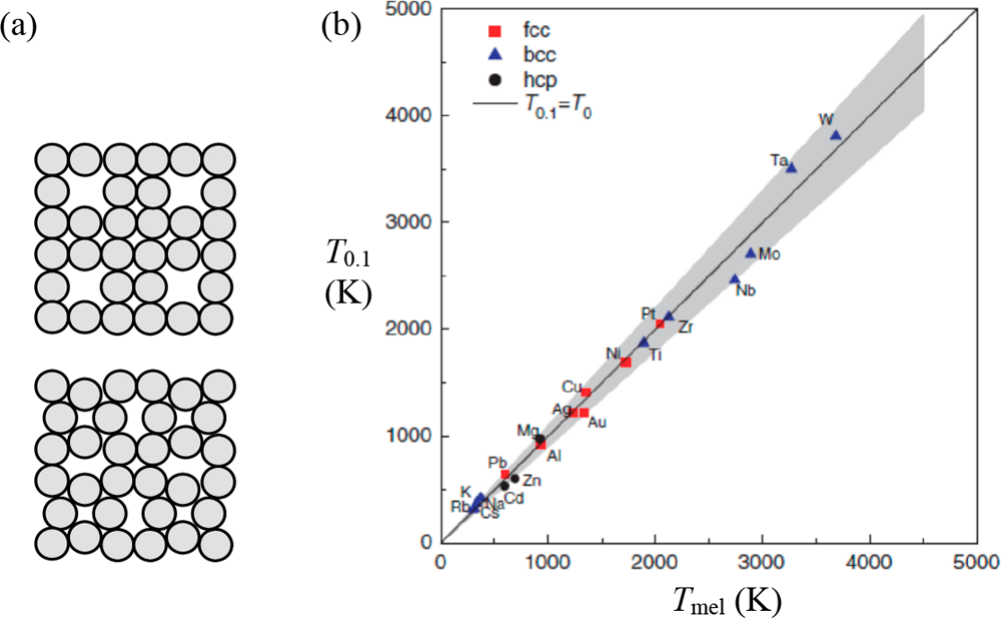
(a) Schematic of a lattice with about 10% vacancies, showing the original lattice (upper part) and the restructured lattice (lower part). (b) Correlation of *T*_0.1_ with *T*_mel_. Reproduced with permission from ref (320). Copyright 2008 Taylor & Francis.

To understand why the lattice becomes unstable at *c* ≅ 0.1, note that the crystal can be viewed as an aggregation of clusters of atoms, each of which has about 10 nearest neighbors around a vacancy (Figure 13a). However, such a configuration of clusters is unstable because local disordering can take place if sufficiently high mobility exists (leading to a configuration akin to an interstitialcy), as first suggested by soap bubble raft simulations.^323^ This local disorder will eventually lead to the collapse of the whole crystal. It was found that the local disordering around a single vacancy can occur when the concentration of migrating atoms *c*_mig_ reaches a critical value *c*_mig_* ≅ 0.17 for FCC and HCP lattices. As *c*_mig_* ≅ 2c*, this indicates that one needs at least two mobile atoms simultaneously. Considering the mobility of atoms, the concentration of mobile atoms at the surface is estimated as *c*_mig_ = exp(4.1 – *E*_mig_/*kT*), where the migration energy of a vacancy *E*_mig_ at the surface is taken, similarly as for their formation, by 2/3 of the experimental bulk values.^320^ Using the Al example again, one calculates that, at *T*_mel_, *c*_mig_ ≅ 0.34, so restructuring indeed can take place. It is also easy to calculate that, at *T* = 820 K, *c*_mig_ ≅ *c*_mig_* ≅ 0.17 and *c*_sur_ ≅ 0.04, which implies that at this temperature surface disordering (roughening) occurs. This disordered layer becomes a liquid when *c*_sur_ ≅ 0.1 at *T*_mel_. In fact, considering several metals, this prediction appeared to be in good agreement with experiment (Figure 13b).

In summary, vacancies are more easily formed at the surface than in the bulk and the mobility of atoms at the surface is much larger than in the bulk. Premelting (roughening) of the surface occurs if the concentration of mobile atoms *c*_mig_ reaches a critical value, estimated as *c*_mig_* ≅ 0.17. If the concentration of surface vacancies *c* reaches a critical value, estimated as c* ≅ 0.10, the lattice becomes locally unstable and restructures around a vacancy. This disordered domain acts as a liquid nucleus. Once both these critical values are reached, disordering proceeds through the whole crystal, eventually leading to a molten crystal. A similar analysis using a defective lattice and employing equality of lattice and liquid entropies as the melting criterion has been given by Fecht^324^ and led to c* ≅ 0.08.

### 6.3 Dislocations Revisited

The DTMs, as discussed in section 5.6, refrained from incorporating surfaces. In a somewhat different form of DTM, Kristensen et al.^325^ took the liquid–solid interface into account. They estimated the rate of dislocation formation as *ċ*_+_ = *A*exp[−β(*E*_for_ + *E*_mig_)] with *A* a constant, *E*_for_ and *E*_mig_ the formation and migration energies of a dislocation segment, and β = 1/*kT*, as usual. For the annihilation rate of dislocations, similarly *ċ*_–_ = *B*exp(−*βE*_mig_)*ρ*^*a*^, with *B* another constant, ρ the dislocation density, and *a* a constant that would be 2 if segments can be considered as small compared to their distance. The energy of a dislocation segment of unit length may be expressed, similarly as before but more condensed, as *U* = (*αμb*^2^/4π)ln(*ζR*/*b*), where ζ = (*b*/*r*_0_)exp(4π*U*_cor_/*αμb*^2^) with *U*_cor_ and *r*_0_ the core energy and radius, respectively. Introducing the dimensionless dislocation density *c* = *ρb*^2^, the excess energy of the crystal with volume *V* may be expressed as *U*_*V*_ = (*αμVc*/4π)ln(ζ/2*c*^1/2^). Here ρ^–1/2^ = 2*r*_0_ was used instead of ρ^–1/2^ = π^1/2^*r*_0_ as used before, but the effect of this difference is negligible. Ignoring lattice relaxations, the formation energy *U*_for_(*c*) as a function of density *c* may thus be estimated as *U*_for_(*c*) = (*αμV*_dis_/4π)ln(ζ/2*c*^1/2^) with *V*_dis_ = *Lb*^2^, where *L* is the dislocation length. It is assumed that this elastic energy expression is valid up to the maximum concentration of dislocations *c*_0_ = ζ ^2^/4e at which *U*_for_ will attain its maximum. In equilibrium *ċ*_+_= *ċ*_–_, and this leads to
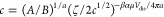
55

**Figure 14:**
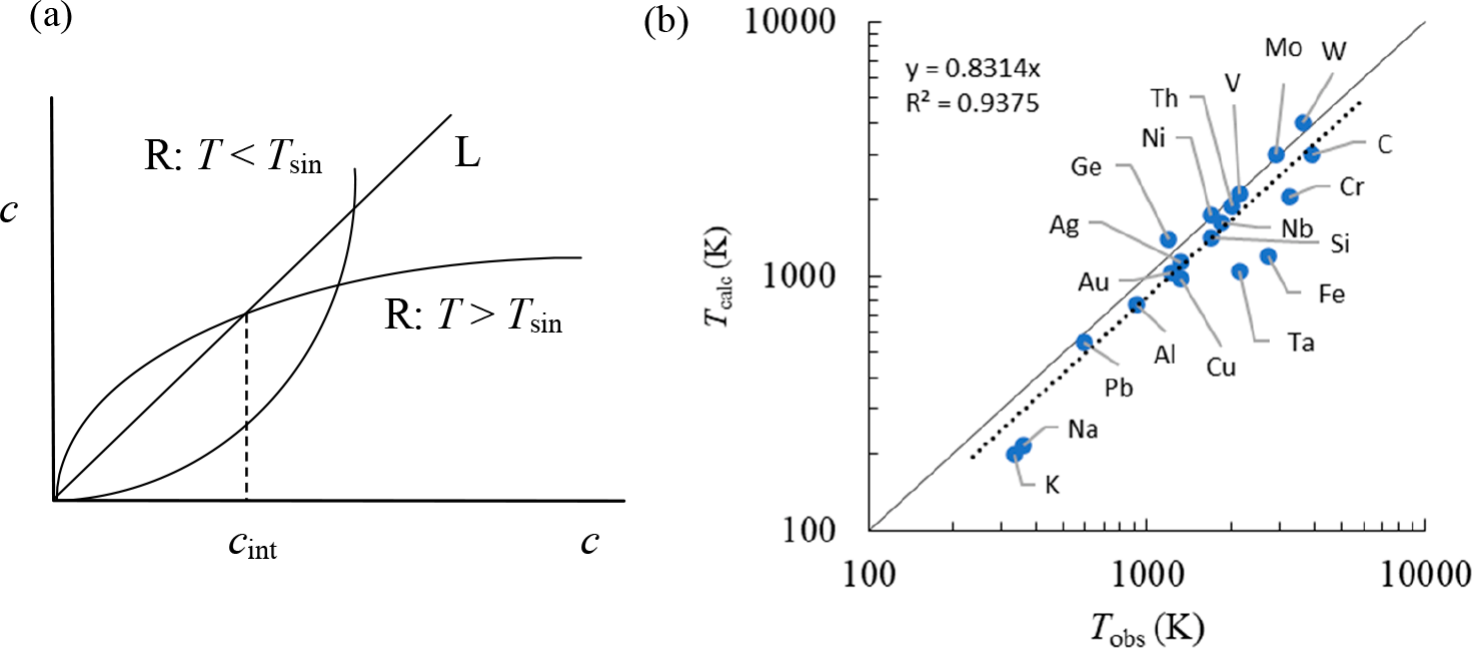
DTM according to ref (325). (a) The left-hand side (L) and right-hand side (R) for *T* < *T*_sin_ and *T* > *T*_sin_ of eq 56 with *T*_sin_ = *αGV*_d_/8π*ka* showing that for *T* > *T*_sin_ melting occurs for *c* > *c*_int_; redrawn after ref (325). (b) The correlation between calculated and experimental *T*_mel_. The solid line represents equal temperatures, while the dotted line represents the fit. Redrawn using data from ref (325).

This expression has the form *z* = *Zz*^*η*^, with as solutions *z* = 0 and *z* = *Z*^1/(1−η)^. Hence
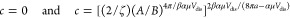
56and the latter expression has a singularity for the temperature *T*_sin_ = *αμV*_dis_/8π*ka*. The behavior of eq 55 is shown in Figure 14a. For *T* < *T*_sin_, the inevitably present fluctuations will lead ultimately to the first solution, describing the solid without any dislocations. For *T* > *T*_sin_, they will lead to the second solution, describing the solid saturated with dislocations. Hence, this temperature is interpreted as an instability temperature, that is, the melting temperature in the absence of an interface.

The presence of an interface will affect the balance between creation and annihilation of dislocations as it can act as a sink or source of dislocations. Assuming a planar interface at *x* = 0 with the liquid phase being described by a dislocation density *c*_0_, for local equilibrium to exist one must have
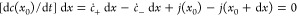
57where *j*(*x*) is the flux at *x*, assumed to be given Fick’s first law *j* = −*D*d*c*/d*x* with *D* = *D*_0_exp(−*βE*_mig_) the diffusion constant for dislocations. Hence, it follows that
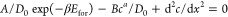
58or, using *E*_for_(*c*) = (*αμV*_dis_/4π)ln(ζ/2*c*^1/2^) and *T*_sin_ = *αμV*_dis_/8π*ka*, that
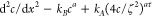
59where *k*_*A*_ = *A*/*D*_0_, *k*_*B*_ = *B*/*D*_0_, and τ = *T*_sin_/*T*. This expression may be integrated to
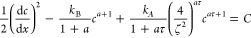
60with as solution
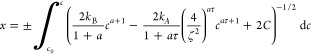
61

The integration constant *C* must be chosen as *C* = 0 to ensure that d*c*/d*x* = 0 for *c* = 0, while the negative root must be chosen to avoid a physically unacceptable negative dislocation concentration. Some analysis of these expressions shows that equilibrium at the interface can be only attained if, for the relative temperature τ = τ_0_
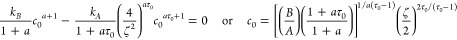
62and consequently τ_0_ is interpreted to represent the melting temperature via τ_0_ = *T*_sin_/*T*_mel_. Estimating values for *A*, *B*, *a*, and ζ, meanwhile using *c*_0_ = ζ^2^/4e, yields an implicit expression for τ_0_. It appeared that, for diamond-type (D) lattices with ζ ≅ 4, *aτ*_0_ is close to 3.8, while for FCC, HCP, and BCC lattices with ζ ≅ 2, *aτ*_0_ ranges from 4.9 to 6.1, so the average 5.5 was used. To estimate *V*_dis_, the details of the dislocation formation process should be considered. The energetically most favorable process is the formation of a dislocation dipole,^326^ and assuming this process occurs with a Burgers vector length of the nearest-neighbor distance *d*_nn_ and a dislocation line length of ≅2*d*_nn_, we have *V*_dis_ = 2*d*_nn_.^3^ Hence, the expression for *T*_mel_ becomes
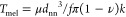
63with *f* = 22.0 for FCC, HCP and BCC and *f* = 15.2 for D structures. Figure 14b shows that a fair correlation between experimental and calculated values of *T*_mel_ is obtained for 19 metals. Apart from that, the above approach takes into account the interface, thereby differentiating between the instability and melting temperature.

Finally, we note that also an approach that employs both dislocations and point defects to explain melting has been proposed.^127^ The treatment of dislocations is 3D, while the treatment of the vacancies is 2D. Using the grand potential *Ω*, the transition between *Ω*_dis_ and *Ω*_vac_ is taken as *T*_mel_, which for the series Li, Na, K, Rb, and Cs shows good agreement with experiment.

### 6.4 Simulations Revisited

As stated in section 5.8, MD and other simulation techniques are illuminating tools resulting in detail that otherwise is hard to obtain. In simulations for bulk materials without a surface, generally full periodic boundary conditions are used. In order to be able to do simulations in the presence of a surface, the simulation box is usually filled with a liquid layer surrounded by vapor and only either the vapor or liquid layer is periodically connected. Here we describe a few of these calculations.

**Figure 15:**
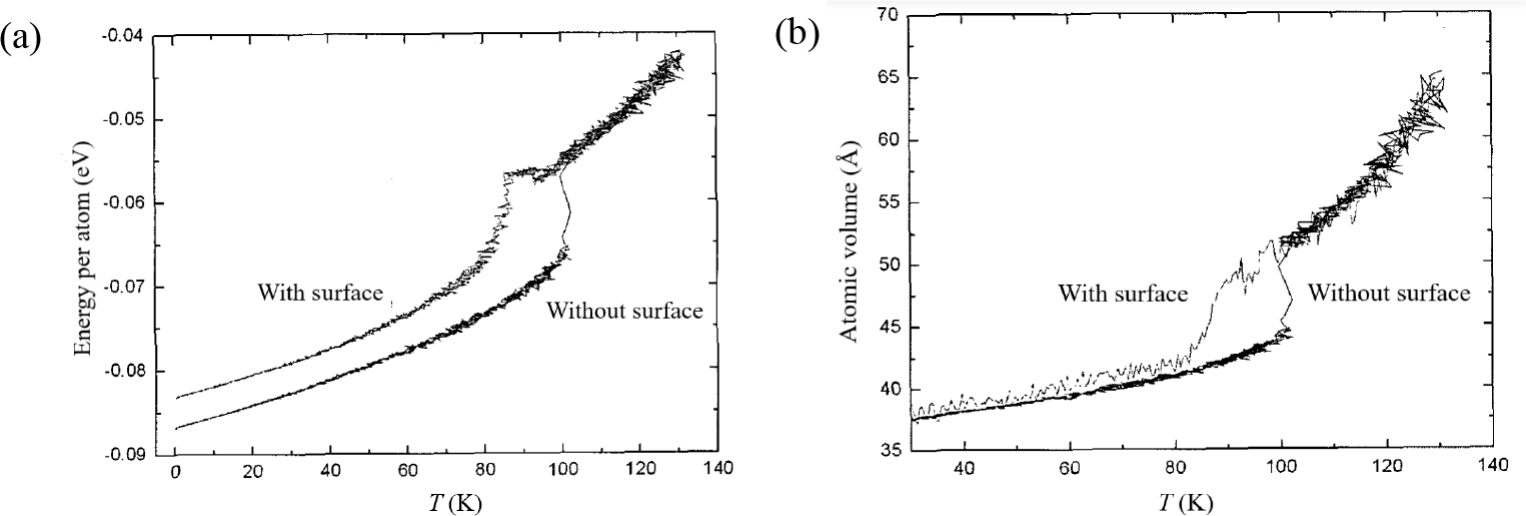
MD simulations for Ar. (a) Energy per Ar atom for a simulation box with and without surface. (b) Volume per Ar atom for a simulation box with and without surface. Reproduced with permission from ref (327). Copyright 2007 The Korean Ceramic Society.

Using such MD simulations with a LJ potential for Ar, Han^328^ clearly showed the difference between bulk and thermodynamical melting (Figure 15). The author mainly analyzed the volume and energy per atom, both in the bulk and at the surface, from which this difference is clearly visible.

In a recent study Fan et al.^329^ showed, using MD simulations with an embedded atom potential for Ta, that the details of a melting process are for more complex than the Lindemann rule can catch. They used three different characteristics, namely (1) the reduced kurtosis (RK) α = 3⟨Δ*r*^4^⟩/5⟨Δ*r*^2^⟩^2^ – 1, where ⟨Δ*r*^2^⟩ and ⟨Δ*r*^4^⟩ are the averages of the second and fourth moments of the atomic displacement distribution Δ*r*; (2) the structure factor (SF) *S*_*i*_(*k*) = ⟨*N*^–2^∑_*j*_ exp(i***k***·***r***_*ij*_⟩, with ***k*** the wave vector along the 110 directions in the BCC structure; and (3) the bond orientational order (BOO) *Q*_*n*_(*i*) = ⟨*N*^–1^∑_*j*_ exp(i*nθ*(*ij*)⟩, where θ(*ij*) is the bond angle formed between the nearest-neighbor distances *r*_*i*_ and *r*_*j*_. They also calculated the parameter ξ for the whole sample as well as for slices with thickness of 1.6 Å cut parallel from the surface.

For the 100 BCC Ta surface it appeared that a steep increase in α, *S*_*i*_(*k*), and *Q*_*n*_(*ij*) occurs at *T* = 1641 K, while the simulated bulk melting temperature is *T*_mel,calc_ = 3094 K, to be compared with the experimental *T*_mel,exp_ = 3290 K and the simulated bulk temperature without surface *T*_mel,calc_ = 3430 K. The parameter α increases up to about 1911 K, whereafter it decreases to become zero at *T*_mel_, while ξ for the bulk continuously increases from *T* = 1641 K onward until *T*_mel_ at 3094 K.

**Figure 16:**
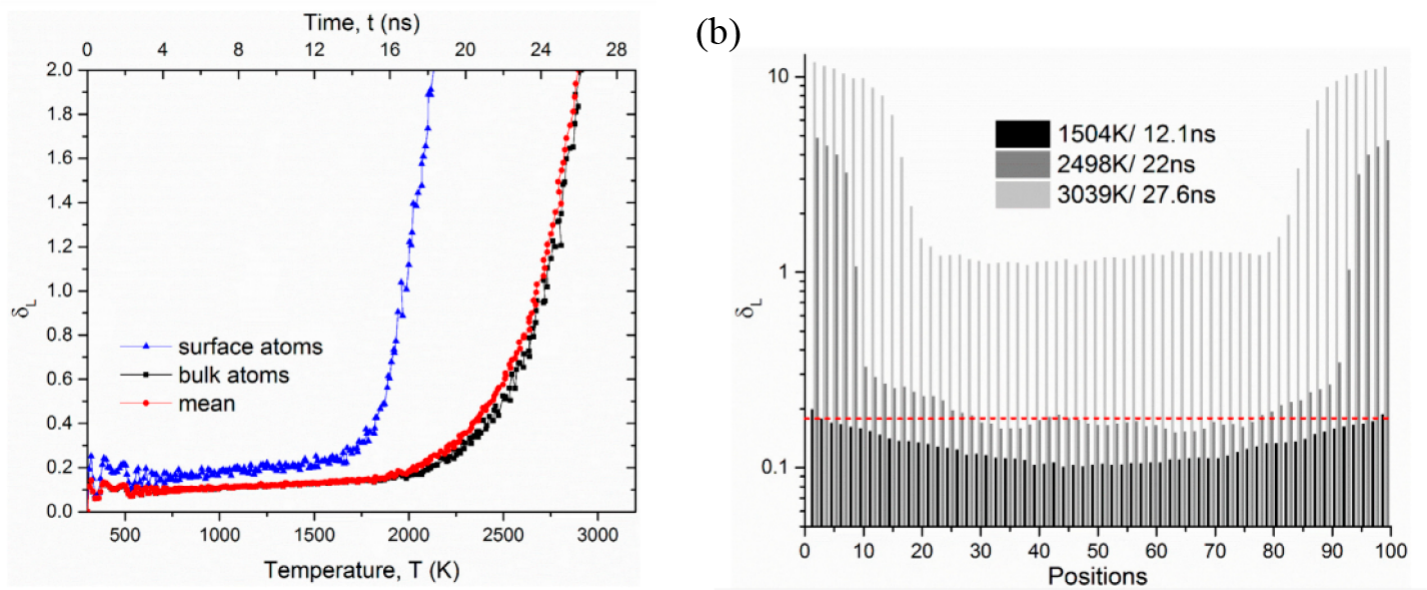
(a) The parameter ξ (labeled in the figure as *δ*_L_) versus *T* for Ta(100) for the surface atoms, bulk atoms and their (weighted) mean. (b) The ξ-profile as a function of depth in the surface and solid for Ta(100). The dashed line shows the predicted value of ξ = 0.19 for the bulk. Reproduced with permission from ref (329). Copyright 2020 Elsevier.

The sudden decrease of the SF at *T*_mel_, the steep decrease of α, and the steep rise of ξ are taken together as indicating a discontinuous transition. Differentiating between various layers, it appears that ξ increases much more strongly at the surface than in the bulk, although the two-dimensional surface pair correlation function shows the cubic symmetry until *T*_mel_ is reached (Figure 16). For the other surfaces ⟨110⟩ and ⟨111⟩ a similar analysis was done. The bulk melting temperatures for the (111), (100), and (111) surfaces occur at *T*_mel_ = 3082, 3094, and 3115 K, respectively, in consonance with expected (111) > (100) > (111) order based on the surface packing densities 1.4/*a*^2^, 1/*a*^2^, and 0.58/*a*^2^ with lattice constant *a* = 3.306 Å (although the difference between (111) and (100) seems small as compared to the difference in packing density). The RK, SF, and ξ all showed similar behaviors as for (100). It also appeared that, although ⟨Δ*r*^2^⟩ increases steadily above 1641 K, the mean position remains at the lattice sites even in the surface layers until bulk melting occurs, as illustrated by the two-dimensional pair correlation function and the fact that the SF remains well in the crystalline region. The authors indicated that the increasing disorder above 1611 K is due to correlated atomic contributions to ⟨Δ*r*^2^⟩ due to chains and loops.^330,331^ Moreover, they conclude that the premelting is a disordering process that does not lead to complete melting until the bulk melting temperature is reached, so that the surface melting appears in synchronization with bulk melting as a discontinuous transition.^332^ The conclusion is that the Lindemann rule using bulk values for ⟨Δ*r*^2^⟩, although reasonably capable of catching the bulk melting temperatures, cannot be related to the surface and its associated ⟨Δ*r*^2^⟩, although (pre)melting starts at the surfaces. Remarkably, though, the authors do not even mention anharmonicity, although non-Gaussian behavior for ⟨Δ*r*^2^⟩ is clearly related to that, besides being related to disordering.

We end with mentioning four other approaches. First, Holian^142^ used the LJD model as an ingredient in a hybrid approach. The LJD model, in either the angular or smeared form and when corrected by the classical harmonic correlational entropy, gives a satisfactory model of a classical solid. The Helmholtz energy of the fluid was obtained from the Hansen–Ree analytic fit to MC EoS calculations for the LJ potential.^333,334^ It appeared that at high densities along the melting curve, the anharmonic correction to the correlational Helmholtz energy approaches a small constant compared to the harmonic contribution and the resulting predictions of the solid–fluid coexistence curves are in excellent agreement with computer experiments. This hybrid model demonstrates that both anharmonicity and long-wavelength-correlated motion must be properly incorporated.

Second, a comparable route was followed by Bhattacharya et al.^137^ using the cell model and employing embedded atom method (EAM) potentials to account for many body interaction effects. The Helmholtz energy obtained was used to determine melting curves of FCC metals. For this purpose, the liquid phase Helmholtz energy was calculated using the corrected rigid spheres model of Kerley.^139^ In this modified perturbation theory, the energy of a fluid molecule is defined by a function which depends upon the local configuration of its neighbors which does not need explicit knowledge of the interaction potential. For Al, Cu, Ni and Pt, the results match well with the available experimental/theoretical data.

Third, we recall the rather different quantum cluster equilibrium (QCE) simulation approach of Weinhold,^335,336^ based on weakly interacting clusters of molecules that do interact strongly within a cluster. While the intracluster interactions are calculated using sophisticated quantum chemistry software, the intercluster interactions are taken into account as perturbations. The model focuses on water and predicts the phase diagram quite well.^337^

Fourth and finally, an interesting result about melting and superheating was given by Belonoshko et al.^338^ Using MD simulations under constant (*N*,*V*,*T*) conditions for 4 × 10^3^ and 32 × 10^3^ particles employing a LJ potential with ε/*k* = 119.8 K and σ = 3.41 Å representing Ar, the authors calculated three isochores for unit cells with lattice constant *a* = 4.2 Å, *a* = 4.4 Å, and *a* = 5.37 Å, verifying that their results are size-independent and not volume specific. As indicated in section 5.8, in simulations a solid can be substantially overheated up to a temperature *T*_LS_, where above which one cannot heat a solid without transforming it into a liquid. The authors noticed that, for all volumes used, when *T* approaches *T*_LS_, a very small increase in the initial kinetic energy leads to melting and that, unexpectedly, the temperature *T* to which the system evolves drops down to *T*_mel_. A similar drop was noticed for an EAM potential for Cu.^339^ Because of the constant (*N*,*V*,*T*) conditions, the energy *U*_S_(*V*,*T*_LS_) of the solid (S) at *T* = *T*_LS_ equals *U*_L_(*V*,*T*_mel_) of the liquids (L) at *T* = *T*_mel_. After reaching *T*_LS_ the temperature decreases because of Δ_m_*H*. The interpretation is that homogeneous melting occurs when the internal energy of the atoms in the solid state is sufficient to explore the potential energy landscape of the liquid state. To demonstrate that the absence of the states with high entropy is the reason for superheating, the authors performed two-phase MD simulations where the LJ parameters for the liquid were chosen quite differently from those for the solid. In these two-phase MD simulations, the solid melted without superheating, confirming that the heterogeneity itself, i.e., the solid–liquid interface, is sufficient to ensure equilibrium melting of the solid. The authors also explained the increase of *T*_LS_ by about 20–30% above *T*_mel_ by considering that the effect of pressure *P*. As the melting curves of simple solids are rather pressure-independent because of the small difference between *V*_L_ and *V*_S_ due to the high pressure, one can write
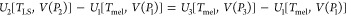
64where *U*_*j*_ is the internal energy for the pressures and temperatures indicated. Assuming that *C*_*V*_ ≅ 3*k*, the standard Dulong–Petit high-temperature estimate, and that Δ_m_*S* ≅ *T*_mel_ ln 2, which is the asymptotic value of Δ_m_*S*,^13^ one can write
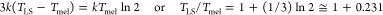
65thereby nicely explaining the order of magnitude increase and confirmed by experiment by Luo et al. for Al.^340^

### 6.5 Surface Transitions

**Figure 17:**
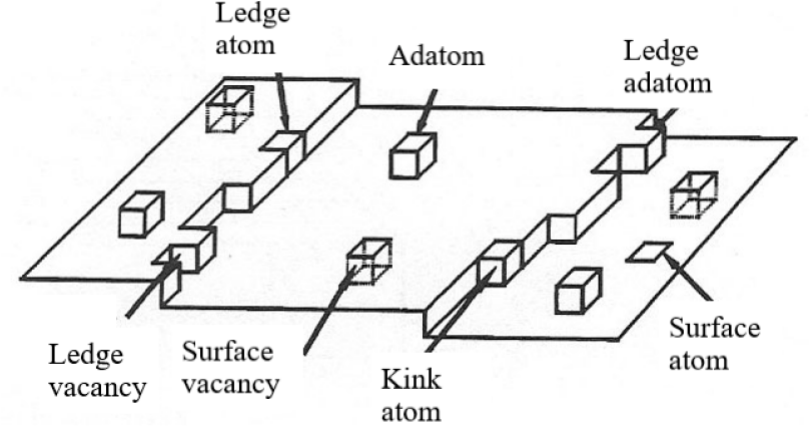
TLK model showing three terraces and two ledges with kinks having ledge vacancies and ledge adatoms and terraces with adatoms and surface vacancies.

Related to surface melting is surface roughening, which describes the deviations from the ideal or bulk-like surface, the structure of which is like that of the corresponding lattice plane in the bulk. Although such ideal surfaces do occur, primarily in metals, generally, low index solid surfaces (for crystals often called facets) are only nominally planar and have steps or ledges. In the steps, kinks (Figure 17) are present representing a deviation from the overall step direction. The planar area between the steps is called a terrace. On both terraces and ledges atoms can absorb (adatoms) and vacancies can arise. Together these features represent the terrace–ledge–kink (TLK) model. Moreover, to lower their surface energy, atoms in the surface region exhibit in general a relaxation from the ideal lattice positions, leading in several cases to reconstruction. For a relaxed surface the overall structure is still like an ideal surface, while for a reconstructed surface a clear symmetry break occurs.

Solid surfaces often show a roughening transformation at a certain *T*_R_, below which they remain nominally smooth but above which they become rough. To model this phenomenon, both continuum and atomistic models have been used.

**Figure 18:**
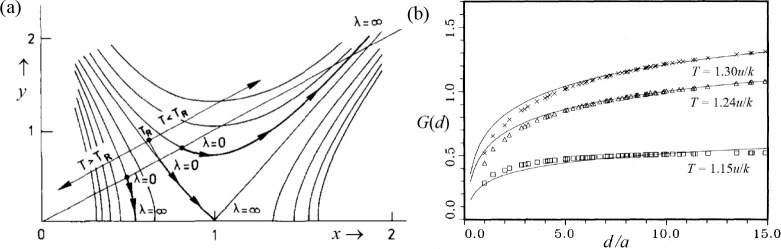
Kosterlitz–Thouless transition. (a) Trajectories as a function of *x*, where all starting points for iteration lie on the straight line *y* = *x* corresponding γ_0_ and *V*. Reproduced with permission from ref (344). Copyright 1994 Elsevier. (b) The height correlation function *G*(*d*) as a function of *d*/*a* (—) fitted to MC simulation results at various temperatures, where *T*_R_ corresponds to *T* = 1.24*u*/*k*. Reproduced with permission from ref (345). Copyright 1978 American Physical Society.

The continuum model for solids uses capillary wave theory with stabilization provided by a periodic lattice potential *V*(*z*) = *V*[1 – cos(2π*z*/*a*)], preferentially locating the surface on planes *z* = *na* above the nominal surface, with *a* the lattice constant and *n* an integer. Assuming periodic boundary conditions in the *x–y* plane with position vector ***r*** and a square area of linear size *L* = *A*^1/2^, one has
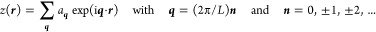
66where ***q*** is a 2D wave vector. The Fourier transform of the energy becomes
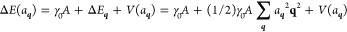
67where γ_0_ is the surface internal energy for the flat surface and Δ*E*_***q***_ is the roughening contribution. The lattice potential leads to a coupling between the modes, complicating the calculation of the partition function *Z* = ∫exp(−βΔ*E*(*a*_***q***_)d*a*_***q***_. To calculate *Z*, one normally applies a renormalization procedure in real space. In brief, by defining a new cutoff ***q***_cut_, one divides the variables into a group having a large wave vector ***q***_lrg_ (***q***_cut_ < ***q***_lrg_ < ***q***_max_ = π/*L*) and one having a small wave vector ***q***_sml_ (***q***_min_ = π/ξ < ***q***_sml_ < ***q***_cut_). Integration of *Z* over the variables with ***q***_lrg_ yields *Z* = ∫exp(−βΔ*E*(*a*_lrg_,*a*_sml_)d*a*_***q***_ ≡ exp[**−***βE*^(1)^(*a*_sml_), where *E*^(1)^(*a*_sml_) is the effective energy for the small wave vector components. By expanding *V*(*a*_***q***_) to second order, one can show that Δ*E*^(1)^(*a*_sml_) has the same form as Δ*E*(*a*_***q***_) but with renormalized variables γ^(1)^(*a*_cut_) and *E*^(1)^(*a*_cut_). This procedure is iterated by defining a new cutoff ***q***_cut_ + d***q***_cut_ and calculating γ^(2)^(*a*_cut_ + d*a*_cut_) and *E*^(2)^(*a*_cut_ + d*a*_cut_) until convergence with solutions γ* and V* is reached. In terms of the dimensionless variables λ = ln(***q***_min_/***q***_cut_), *x* = 2*βγ*(λ)*a*/π, and *y* = 4π*βV*(λ)/***q***_cut_^2^, one obtains after some algebra in the continuum limit
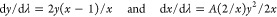
68with *A*(2/*x*) a slowly varying function.^341^ These equations define the Kosterlitz–Thouless class^342,343^ of phase transitions, the solutions of which (Figure 18a) show two types of behavior, dependent on whether *T* < *T*_R_ or *T* > *T*_R_. For *T* < *T*_R_ there is always a ***q***_cut_ above which the renormalized potential V* goes to infinity, fluctuations are pinned, and the surface remains smooth. For *T* > *T*_R_, V* always vanishes on a large scale and the surface becomes rough. Hence, *T*_R_ = 2γ*a^2^/π*k* is identified as the critical temperature given by the fixed point at *x* = 1 and *y* = 0. Assuming an isotropic surface (which {100} SC is not, but {111} FCC is), the height correlation function becomes
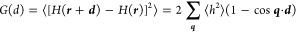
69
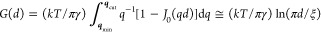
70

The last step is made by approximating the zero-order Bessel function *J*_0_ with a Heaviside step function at *qr* = 1 and is only valid for large *r*, as we must have *G*(0) = 0. The extrinsic width is given by ⟨*h*^2^⟩ = (*kT*/πγ_∞_*) ln(*L*/ξ) with γ_∞_* = γ*(λ=∞). As an expansion of *V* is used, *V* must be small and fails for *T* < *T*_R_ when V* → ∞. Although this renormalization procedure clearly shows the universal nature of the roughening transition, atomistic models are evidently needed.

The classic atomistic model is by Burton, Cabrera, and Frank^346^ using for this order–disorder problem the quasi-chemical solution as well as the exact Onsager solution for a three-level nearest-neighbor bond model with bond energy *u* which includes surface atoms, surface adatoms, and surface vacancies. The latter for a square lattice yields *T*_R_ = *u*/*k* ln(1 + 2^1/2^) ≅ 1.13*u*/*k*, but even that is still a poor estimate: when a large cluster is formed, another cluster can easily build on top of its surface.

**Figure 19:**
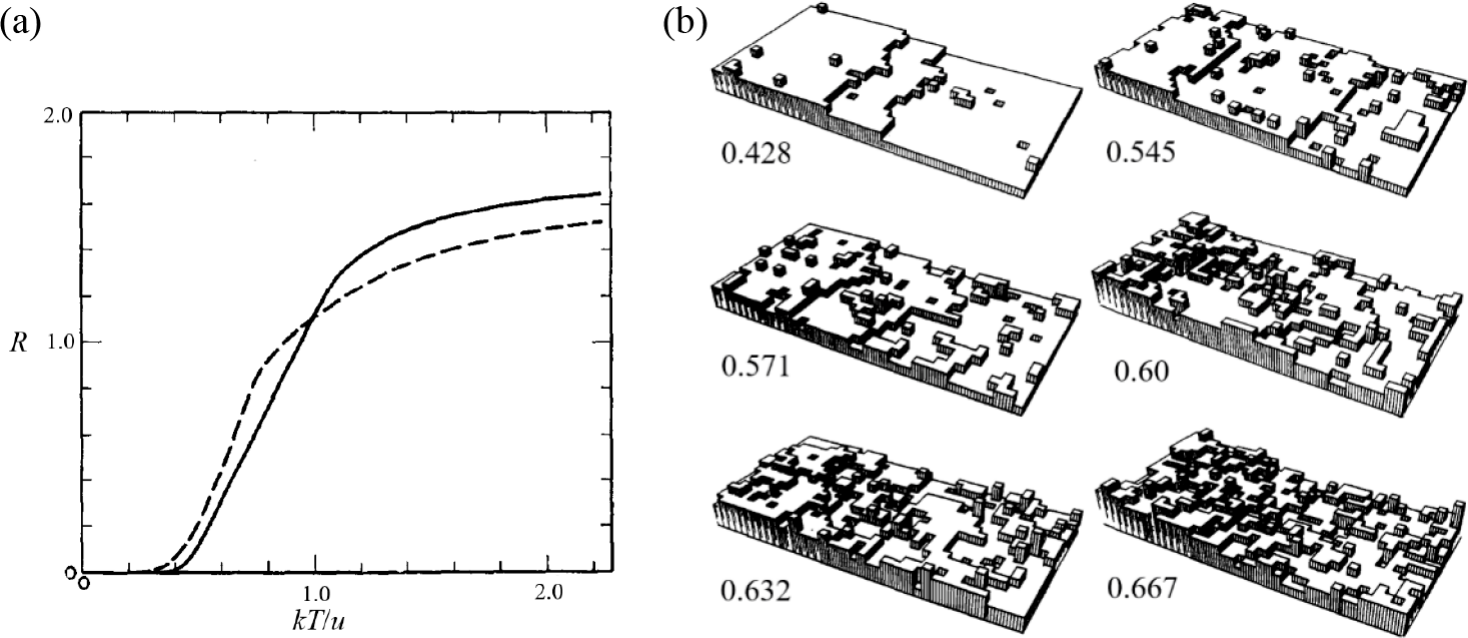
(a) Surface roughness *R* for {100} SC surfaces as a function of 1/*βu* for a three-layer model according to an approximate solution by Mullins^347^ (—, not discussed) and the exact solution by Burton et al.^346^ (− −). Reproduced with permission from ref (347). Copyright 1959 Elsevier. (b) Perspective images of representative (20,1,0) surface configurations at various values of 2*kT*/*u* with 2*kT*_R_/*u* ≅ 0.64. Reproduced with permission from ref (348). Copyright 1974 Elsevier.

Hence, the three-level model is insufficient, but the three-level approximation can be avoided by using the solid on solid (SoS) model. Considering a SC lattice with forbidden overhangs, the model contains columns of height *z* relative to the flat surface at *T* = 0 K, or *z*_*j*_ = *z*(*x*_*j*_,*y*_*j*_) where (*x*_*j*_,*y*_*j*_) are the coordinates of the column. The excess energy *U*(*z*_*j*_) is related to the number of free vertical faces of the cubes in the column and given by *U*(*z*_*j*_) = (1/2)*u*∑_*j*,δ_*f*(*z*_*j*_ – *z*_*j*–δ_), where δ runs over the neighboring columns. Using the bond model, the function *f*(*z*_*j*_ – *z*_*j*–δ_) = **|***h*_*j*_ – *h*_*j*–δ_**|**. This defines the absolute SoS (ASoS) model,^345^ from which we recover the three-level model if we restrict *z*_*j*_ to −1, 0, and 1. This model must be solved numerically, which can be done by the MC method in the (μ,*T*,*V*) ensemble using as probability exp[−β(Δ*U* – μ)]. From the results one can obtain for a distance *d* between columns, the height correlation function . The results of such an ASoS calculation for the SC lattice (Figure 19b) shows that, for *T* < *T*_R_, *G*(*d*) tends to a constant value for a large value of *d*, while for *T* > *T*_R_*G*(*d*) diverges weakly. The height correlation function can be described by *G*(*d*) = (*kT*/πγ_0_*)(ln *d* + *c*), where *c* is a constant. By fitting the *G*(*d*) curves with this expression, it was found that *T*_R_ ≅ 1.24*u*/*k*. Images from MC simulations on (20,1,0) SC surfaces at various temperatures^348^ show that considerable roughness is already present below *T*_R_. A body centered SoS (BCSoS) model^349^ describing the {100} and {111} FCC surfaces can be solved analytically and the condition that *h*_*i*_ – *h*_*j*_ = 0 or ±1/2, resulting in *T*_R_ = *u*/*k* ln 2 ≅ 1.44*u*/*k*.

We note that the SoS roughening transitions belong to the Kosterlitz–Thouless class.^342,343^ The nonsingular part of the Helmholtz energy as a function of *T* when approaching *T*_R_ follows *F* ∼ exp[−*c*′/(|*T* – *T*_R_|^1/2^)] with *c*′ another constant and vanishes at and above *T*_R_. All thermodynamic quantities and their derivatives are continuous to any order, thereby explaining why the roughening transition is rather smooth as compared to the order–disorder continuous transition model. The disappearance of crystal facets is a characteristic of roughening and has been observed experimentally. For example, for Pb below 323 K the bounding facets are (111), (110), (100), and (112). For 323 K < *T* < 393 K the (112) facets are absent, while above 393 K up to *T*_mel_ the only remaining facets are (111) and (110). The latter planes are the most close-packed, and for them *T*_R_ > *T*_mel_.

For the melting transition in 2D crystals Kosterlitz and Thouless^342^ suggested that it is a continuous process mediated by the dissociation of dislocation pairs. The resulting phase of this continuous transition was shown by Nelson and Halperin^350−352^ not to be an isotropic phase because it still has a quasi-long-range orientational order. Young^353^ pointed out that a second transition, which is induced by the formation of disclinations, would drive this so-called hexatic phase into a liquid. This theory is now known as the Kosterlitz–Thouless–Halperin–Nelson–Young (KTHNY) theory and thus proposes that a 2D solid first shows a transition to a hexatic phase with a quasi-long-range orientational order and thereafter another transition from the hexatic phase to a liquid.

A large number of experiments and simulations has been performed to verify KTHNY theory; see, e.g., refs (354−356). The results for simulations remain somewhat controversial, which may be caused by the size effect because long-wavelength fluctuations play an important role in KTHNY theory, while they are cut off in finite-sized simulations. Large-scale simulations of LJ systems do seem to provide evidence for the existence of the hexatic phase,^357^ although it was argued that this transition might depend on the specific properties, such as the interparticle potential of the studied systems.^358,359^ However, a series of experiments was performed to calculate the elastic moduli and the dislocation interactions in 2D colloidal crystals,^360−363^ from which the renormalized Young’s modulus *K*_R_ of the crystals was found to be consistent with KTHNY theory, while the dissociation of dislocations was observed experimentally.

## 7 The Influence of Pressure

Melting is influenced by pressure, as indicated by the Clapeyron–Clausius equation. For example, for water at 0 °C, Δ_mel_*H* = 80 cal g^–1^, *V*_L_ = 1 cm^3^ g^–1^, and *V*_V_ = 1.09 cm^3^ g^–1^_,_ resulting in d*T*_mel_/d*P* = −0.0074 K atm^–1^, to be compared with the experimental value of −0.0075 K atm^–1^. Consequently, the triple point is *T*_tri_ = 0.0075 °C. In this section we discuss the influence of pressure. First, we deal with the thermodynamics and thereafter with some Lindemann-based and related approaches. We limit the discussion here to papers related to model approaches for high-pressure experiments, while in section 8 we deal with individual compounds. For a review of experimental studies on molecular materials, see ref (364), while first-principles modeling of Earth and planetary materials is dealt with in ref (365). Many aspects of EoSs for solids at high pressures and temperatures have been presented by Zharkov and Kalinin.^366^

### 7.1 Thermodynamic Approach

The effect of pressure on the melting point over a wider pressure range is almost universally described by the empirical *Simon–Glatzel equation*(367) Δ*P*/*a* = (*T*/*T*_0_)^*b*^ – 1, where *a* and *b* are parameters; Δ*P* = *P* – *P*_0_ with *P*_0_ and *T*_0_ a reference pressure and temperature, often taken as the triple point. In many cases *P*_0_ is also neglected, as normally *P*_0_ ≪ *P*. Equivalently, in differential form, we can write d[d*P*/d(ln *T*_mel_)]/d*P* = *b*. The Simon–Glatzel expression fits experimental fusion curves reasonably well by a proper choice of the two parameters *a* and *b* for molecular solids but overestimates *T*_mel_ for metals and ionic compounds.^368^ A few examples for molecular compounds are given in Table 4. From the data sets given for Ar, it will be clear that the precise values depend on the pressure range used. The results depend also on the experimental techniques used, as high-pressure experiments are loaded with pitfalls. For a discussion of these problems, we refer to the literature, e.g., ref (369). The data for cyclohexane indicate that a small amount of impurity can lead to significantly different parameters. Many data, mainly for molecular compounds, have been collected by Babb.^370^ However, the Simon–Glatzel equation can only describe increasing melting points with increasing pressure (normal melting), while experimentally decreasing melting points with increasing pressure (anomalous melting) do occur.

**Table 4:** Simon–Glatzel Parameters for Various Materials

molecule	*P*- and *T*-ranges	Δ^a^ (K)	*T*_0_ (K)	*P*_0_ (kPa)	*a* (MPa)	*b*
Ar FCC^365^	1.3–6.3 GPa	–	82.9^b^	–	210	1.556
	296–740 K					
Ar FCC^369^	0–6.3 GPa	2%^c^	83.80	68.90	224.5 (3.2)	1.5354 (0.0044)
	83–840 K					
Ar FCC^371^	1.3–4.2 GPa	21^d^	83.80	69	244	1.476
	294–495 K					
CH_4_^372^	2.4–3.2 GPa	20^d^	90.69	117	208	1.698
	410–558 K					
benzene 99.8%^372^	1.3–174 MPa	0.044^e^	278.24	–	347.9	2.7111
	280–323 K					
cyclohexane 99.5%^373^	0.1–85 MPa	0.040^e^	278.88	–	289.8	1.7838
	280–323 K					
cyclohexane 99.95%^373^	0.1–85 MPa	0.040^e^	279.55	–	280	1.8262
	280–323 K					
water (ice VII)^373^	2–13 GPa	50^d^	354.8^f^	2170^f^	1253	3.0
	350–750 K					
methanol 99.5% (water 0.01%)^369^	9.1–265 MPa	0.12^e^	175.17	–	358.6	3.2443
	176–208 K					
ethanol 99.7% (water 0.2%)^374^	8.5–199 MPa	0.12^e^	158.37	–	436.9	2.6432
	160–183 K					

a Deviation.

b Calculated from data given.

c Average deviation.

d The rms deviation.

e Maximum deviation.

f Triple point ice VI–ice VII–liquid.

The approach to rationalize the Lindemann rule, as described in section 5.1, can also be used to rationalize the Simon–Glatzel equation. As at high pressure repulsion is dominant, using a potential with repulsion only, ϕ = ε(*r*_0_/*r*)^*n*^ with ε and *n* constants, for a lattice with coordination number *z* may suffice. Neglecting thermal energy, the reduced potential in the harmonic approximation using *x* = *r*/*V*^1/3^ becomes
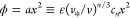
71with v_*_ = *r*_0_^3^/γ (γ = 2^1/2^ for FCC), *c*_*n*_ a number dependent on the crystal structure, and *n* the repulsion exponent. Hence, here the force constant *a* is specified in terms of volume. Again assuming *v*_f_* = *const*. leads via *βϕ* = const at melting to (*zε*/*kT*_mel_)(v_*_/*v*_mel_)^*n*/3^*c*_*n*_ = *const*. Writing *V*_mel_ = *V*_0_ – Δ*V* with Δ*V* = *V*_0_ – *V*_mel_ and *V*_0_ a reference volume at reference temperature *T*_0_, the result is
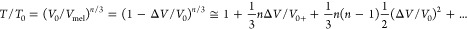
72

Using eq 71 and the reduced Helmholtz expression *F* = (1/2)*NΦ*(0) – *NkT*(ln*Vv*_f_*), one obtains *P*(*T*,*V*) = *P*(0,*V*) + 3*γkT*/*V* with γ = (*n*/6 + 1/3) the Grüneisen parameter for this model. Substituting eq 41 in the pressure expression leads to
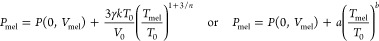
73with *a* = 3*γkT*_0_/*V* and *b* = 1 + 3/*n*, in which the Simon–Glatzel equation can be recognized. Referring to his calculations on Ar, Ross^58^ indicated that neither *P*(0,*V*_mel_) nor γ is really a constant, with as conclusion that the Simon–Glatzel equation cannot be extrapolated with any confidence, contrary to the opinion of Stishov.^375^

The rationalization given above improves on the one given by Salter^376^ in which the Lindemann rule and the Grüneisen EoS were used to derive the Simon–Glatzel expression. The Simon–Glatzel relation can also be justified^377^ by the Lennard-Jones–Devonshire theory.^33^ Neglecting second- and higher-order terms in eq 72 leads to the so-called *Kraut–Kennedy relation*. This equation is not without dispute,^368,378,379^ as it has been defended as a nonapproximated equation, although the various data and rationalizations, such as the one in section 5.1, show otherwise.

One of the reasons that the Simon–Glatzel curve should not be used as an extrapolation equation is that repulsion, as described by ϕ_LJ_ = ε(*r*_0_/*r*)^*n*^, is too steep for small distances, i.e., at high pressure, and is, possibly, better described by ϕ_M_ = *A* exp(−*αr*).^369^ As for power-law repulsion *n* = −(*r* d ln ϕ_LJ_/d*r*), using the same expression for ϕ_M_ leads to an effective *n*-value reading *n = αr*. Approximating the lattice energy by *U* = *zϕ*_M_ = (1/2)*zA* exp(−*αr*_1_) with *z* the coordination number and *r*_1_ the nearest-neighbor distance, the pressure *P* = −∂*U*/∂*V* for a close-packed configuration becomes
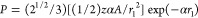
74

As shown above, power-law repulsion leads to *P* = *aT*^1+3/*n*^, so d*P*/*P* = (1 + 3/*n*) d*T*/*T* = (1 + 3/*αr*_1_) d*T*/*T*. Integrating we obtain
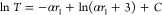
75The latter two equations together describe the melting curve parametrically as a function of *r*_1_. For *P* → ∞, *r*_1_ → 0 and the exponential repulsion predicts a limiting maximum temperature *T* = exp(ln 3 + *C*), whereas the power-law repulsion leads to a divergence. Hence, the results deviate, to a degree depending on the pressure range used. However, this can only be part of the story, as ln *T* does not decrease for *P* → ∞.

The Simon–Glatzel equation can be derived from the Clapeyron equation d*T*/d*P* = Δ*V*/Δ*S*, as has been shown by Boguslavskii.^380^ Taking the first order changes
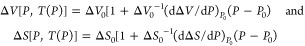
76rewriting the nominator by Δ*V*[*P*,*T*(*P*)] = Δ*V*_0_/[1 – Δ*V*_0_^–1^(dΔ*V*/d*P*)_*P*0_(*P* – *P*_0_)] and integrating the expression for
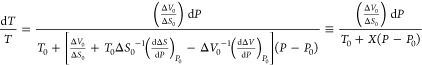
77under the condition that for *T* = *T*_0_, *P* = *P*_0_ leads to the Simon–Glatzel equation
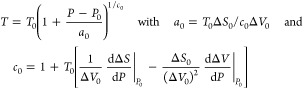
78

This expression is valid for the pressure range *P*_0_ < *P* < *T*_0_/*X*. Data for the alkali metals and H_2_ were fitted within this range, which led to *a*_0_ and *c*_0_ values differing by 1 to 12% from previous results.^370^ This was attributed to the fact that these values were obtained by the least-squares method including data outside the pressure validity range.

Kechin^381−384^ also showed that the Simon–Glatzel equation can be derived from the Clausius–Clapeyron equation, written as d(ln*T*_mel_)/d*P* = Δ*V*/*L* with Δ*V* the volume change and *L* the enthalpy for melting, using a first-order Taylor series in Δ*P*. Later he extended the Simon–Glatzel equation by developing d(ln*T*_mel_)/d*P* in a Taylor series to second order. This leads to
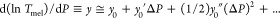
79where the single and double primes indicate the first- and second-order derivatives, respectively. However, in numerical analysis it is well-known that a truncated power series of a function is an unsatisfactory approximation. A more sophisticated method is the use of Padé approximants^385^ which represent the function by a ratio of polynomials. The coefficients are found by expanding the ratio and requiring the coefficients to represent the first *k* Taylor coefficients correctly. For example, *f*(*x*) ≅ *c*_0_ + *c*_1_*x* + ... + *c*_*k*_*x*^*k*^ is approximated by the [*n*,*m*] approximant *f*(*x*) ≅ (*a*_0_ + *a*_1_*x* + ... + *a*_*n*_*x*^*n*^)/(1 + *b*_1_*x* + ... + *b*_*m*_*x*^*m*^). Obviously, *n* + *m* + 1 should equal *k* + 1. A matrix recipe is given by Ree et al.^386^ For a second-order Taylor expansion *f*(*x*) = *c*_0_ + *c*_1_*x* + *c*_2_*x*^2^, the (1,1) Padé approximant reads *f*(*x*) = {*c*_0_ + [*c*_1_ – (*c*_0_*c*_2_/*c*_1_)*x*]}/[1 + (*c*_0_/*c*_1_)*x*]. In this case the result can be written as
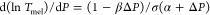
80with the parameters σ, α, and β given by
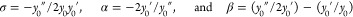
81

**Figure 20:**
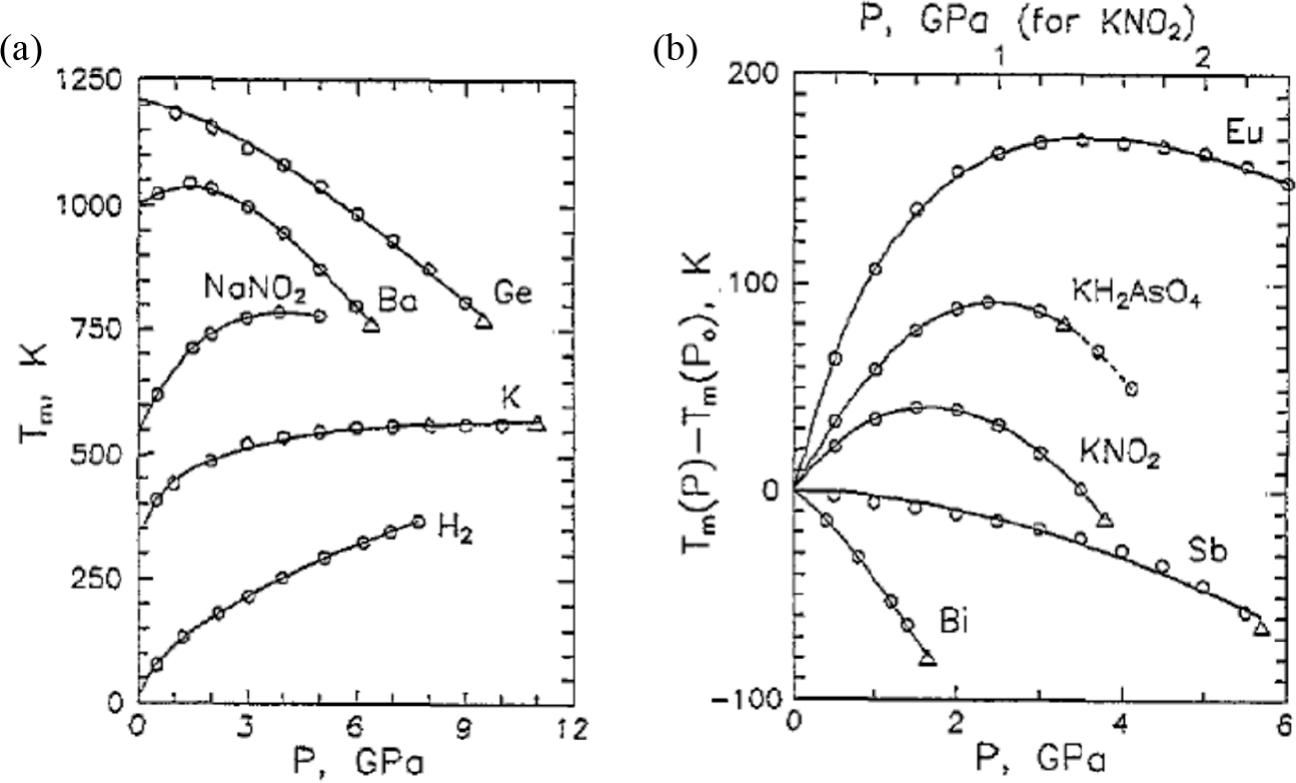
Melting curves for various compounds with (○) experimental data points, (△) the high-pressure triple point, and (—) the fit to the Kechin equation. Reproduced with permission from ref (384). Copyright 1995 IOP Publishing.

This expression can be integrated to
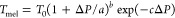
82with *a* = α, *b* = (1 + *αβ*)/σ, and *c* = β/σ = −*y*_0_′α – *y*_0_, which can all be given an interpretation in terms of thermodynamic quantities. If *c* = 0, the *Kechin equation*, eq 82, reduces to the Simon–Glatzel equation with 1/σ equal to *b*. As *y*_0_′ < 0, the parameter *b* > 0, and Δ*P* = 1/β corresponds to a maximum in the melting curve. The hypothesis that all materials show maximums in their melting curves was first suggested by Tammann^387^ and refined by Kawai and Inokuti^388,389^ by postulating that the maximum could occur at positive as well as negative pressure. The latter case corresponds to a decreasing-with-pressure melting curve. As for the Kechin curve, the maximum corresponds to a negative pressure if β < 0; the equation can represent both increasing-with-pressure and decreasing-with-pressure melting curves (Figure 20).

It may be useful to explain the occurrence of a maximum in the melting curve by a simple model,^390^ in which it is supposed that the short-range structure in the liquid is similar to that of the solid near the melting line. If a second structure appears in the solid as a high-pressure phase, in the liquid phase a second structure with a short-range order similar to that of the high-pressure solid phase may also appear, though at lower pressure. Because of the disorder, the transition to the higher density phase occurs continuously in the liquid, while it can only occur with a jump in the solid phase. The liquid may thus become denser than the solid in a certain *P*–*T* range and the melting curve will show a maximum. Similar remarks were made by Stishov.^375^

The relationship between anomalous (reentrant) melting and the features of the repulsive part of the intermolecular potential were studied in detail for one-component systems with radially symmetric interactions by Malescio and Saija.^391^ By making use of the LJD cell model, the authors derived a single-phase criterion for the occurrence of a temperature maximum in the melting line using analysis of the (repulsive) potential in combination with MC simulations. For the analysis they used a Lindemann fraction defined by ξ = *d*^–1^⟨*N*^–1^∑_*j*_(Δ*r*_*j*_)^2^⟩^1/2^, where *d* is the nearest-neighbor distance, *N* is the number of particles, and the brackets denote the average over the dynamic trajectories of the particles. To evaluate ξ they employed the LJD cell model with the displacements calculated in the harmonic approximation *U*(*r*) = *U*(*r*_0_) + (1/2)κΔ*R*^2^, where Δ*r* = *r* – *r*_0_ is the displacement of the atom from its static equilibrium position *r*_0_ and κ is the reduced force constant:^392^
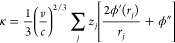
83where the pair potential was written as *u*(*r*) = *εϕ*(*r*/σ). As usual, ϕ′(*r*) = dϕ(*r*)/d*r* and ϕ″(*r*) = d^2^ϕ(*r*)/d*r*^2^, while *v* = *V*/σ^3^*N* is the reduced volume, *z*_*j*_ is the coordination number of the crystal lattice, *r*_*j*_ = *c*_*j*_(*v*/*c*)^1/3^, and *c*_*j*_ and *c* are constant depending on the geometry of that crystal lattice that, for the chosen interaction model, corresponds to the most stable solid phase.

If κ/*t* ≪ 1, where *t* = *kT*/ε is the reduced temperature, one can write ξ ≅ (3*t*/κ)^1/2^ or *t* ≅ (1/3)ξ^2^κ, and a melting curve maximum will occur if κ has a maximum as a function of volume. Accordingly, for the volume *v*_max_ corresponding to the maximum melting temperature, the condition ∂κ/∂*v*|_*v*=*v*_max__ = 0 should hold, which leads to
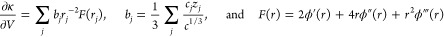
84

Because both *b*_*j*_ and *r*_*j*_^–2^ are always positive, *F*(*r*) should change sign over its definition domain in order for the sum in the above equation to be able to become zero. This provides a simple criterion, called the *n*-criterion, that expresses a necessary condition for the occurrence of anomalous melting. For inverse-power potentials with *n* > 1, *F*(*r*) is everywhere negative and thus the criterion is not satisfied. Hence, a modified inverse power (MIP) potential was used, given by
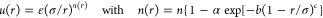
85

Here α is a real number with 0 < α < 1, *b* is a positive real number, and *c* is an even positive integer. The parameter α controls repulsion softening: the larger the value of α, the more significant the softening effect. The parameter *b* controls the width of the interval where *n*(*r*) is significantly smaller than *n*: the larger the value of *b*, the smaller this interval. The exponent *n*(*r*) attains its smallest value *n*_min_ = *n*(1 – α) for *r* = σ. For the various values, *n* = 12, *c* = 2, and *b* = 5 were chosen. As α approaches 1, *u*(*r*) develops in a certain range of *r* a downward concavity, a feature that is typical of core softening potentials. In the region where for the interparticle potential *u*(*r*) the derivative *u*″(*r*) ≤ 0, the strength of the two-body force *f*(*r*) = −*u*′(*r*) reduces or at most remains constant as two particles approach each other. For hard-core repulsion at small distances going to zero sufficiently fast at large distances, such behavior gives rise to two distinct regions where the repulsive force increases as *r* gets smaller. Hence, two distinct repulsive length scales emerge: a smaller one (“hard” radius), dominant at the higher pressures, and a larger one (“soft” radius), effective at low pressure. In the range of pressures where the two length scales compete, the system behaves as a “two-state” fluid. The conditions for core softening, as given by Debenedetti et al.,^393^ require that, in some interval *r*_1_ < *r* < *r*_2_, Δ[*rf*(*r*)] < 0 for Δ*r* < 0, together with *u*″(*r*) > 0 for *r* < *r*_1_ and *r* > *r*_2_, which are satisfied if, in the interval (*r*_1_,*r*_2_), the product *rf*(*r*) reduces with decreasing interparticle separation. This requirement is less rigorous than the condition *u*″(*r*) ≤ 0, and can be met by a strictly convex potential, provided that in a range of interparticle distances the increasing rate of *f*(*r*) is sufficiently small with respect to the adjacent regions.^394^ For the MIP potential used, a downward concavity is present for α ≥ 0.72 and the Debenedetti condition is satisfied for α ≥ 0.68, while according to the *n*-criterion anomalous melting is possible for α ≥ 0.47.

For the MIP potential, MC simulations using constant (*N*,*P*,*T*) conditions, the Metropolis algorithm with periodic boundary conditions and nearest image convention were done. The simulations were done with *N* = 686 for a BCC crystal and *N* = 864 for a FCC crystal, for which finite-size effects appeared to be negligible. From the results obtained, it appeared that, as α increases starting from α = 0, the MIP potential goes gradually from an inverse power 1/*r*^12^ form to a typical core-softened form for α > 0.72. Around α = 0.55, i.e., for a softening much weaker than that leading to a region with downward concavity, a reentrant portion appears in the melting line. The consequent ordering–disordering transition upon pressure increasing at constant temperature is reflected in the behavior of the peaks of pair correlation function *g*(*r*) that, however, shows yet no hint of the existence of two distinct length scales. Thermodynamic, dynamic, and structural anomalies are absent or are restricted to an extremely reduced portion of the *PT* plane. Only at higher α, where the core-softening condition is satisfied, the two-scale behavior typical of core-softened systems is shown by *g*(*r*) and these anomalies fully develop. Other potentials used were the Yoshida–Kamakura potential^395,396^ and the repulsive-step potential, consisting in a hard core plus a finite square shoulder at a larger radius, the latter smoothed by a tanh function.^397^ For these potentials comparable results were obtained. These results show that the class of isotropic systems that can result in anomalous behavior is much wider than commonly assumed.

While such approaches are reasonably successful from a practical point of view for not-too-high pressures, Stacey^398,399^ indicated that most *T*_mel_(*P*) expressions proposed do not satisfy the thermodynamic constraints for really high pressures, as occur in the Earth’s interior. Emphasizing the role of the higher order derivatives *K*′ ≡ d*K*/d*P* and *K*″ ≡ d^2^*K*/d*P*^2^ of *K* = d*P*/d ln ρ, Stacey and Davis^400^ derived the identity
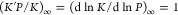
86that all *T*_mel_(*P*) expressions must satisfy. Here and in the following the subscripts “0” and “∞” indicate zero and “infinite” pressure, respectively. At really high pressure, *K*′ is closer to *K*_∞_′ than to *K*_0_′. It was pointed out that there are thermodynamic bounds *K*_∞_′ > 5/3^401^ and γ_∞_ > 2/3,^398^ that many of the 34 expressions examined^398^ failed this test, and that there is no corresponding test for *K*_0_′.^399^ Stacey and Hodgkinson^402^ introduced a constraint using the thermodynamic Grüneisen parameter γ reading
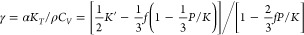
87

Stacey^399^ indicated that the difference between the isothermal bulk modulus *K*_*T*_ and adiabatic bulk modulus *K*_*S*_ can be neglected. While true for geological materials, for molecular solids this is not so. Further, *f* is a parameter that depends on the details of the thermal vibrations and is not necessarily constant. Using *f* = 0 results in the Slater expression γ = (1/2)*K*′ – 1/6,^9,403^ while *f* = 1 yields the Dugdale–MacDonald expression.^404^ Barton and Stacey,^405^ based on FCC MD simulations with various potentials, obtained *f* ≅ 2.31. Assuming random, uncorrelated thermal vibrations, *f* = 2,^406^ as first derived by Vashchenko and Zubarev^407^ in another way, but this assumption is not valid.^405^ Keeping *f* general, it follows from eqs 86 and 87 that
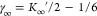
88and is thus, not containing *f*, generally valid. Another identity, namely
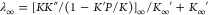
89can be derived^402,408,409^ for which the nominator remains finite negative although both *KK*″ and (1 – *K*′*P*/*K*) vanish for *P* → ∞. The infinite pressure asymptotes of derivatives of *K* can all be represented in terms of the two constants *K*_∞_′ and λ_∞_ and suggest a Taylor expansion of *K*′ as a function of *P*/*K*, evaluated at *P* = ∞. However, although all coefficients can be expressed in terms of *K*_∞_′ and λ_∞_, they are all also found to be indeterminate except the first. The latter can be written, as using eq 86, as
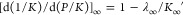
90

Because λ_∞_ < *K*_∞_′, this derivative must be positive, which provides another constraint. Finally, Stacey and Hodgkinson^402^ noted that both *K*_∞_′ and λ_∞_ depend on the type of material, suggesting that a “universal” EoS does not exist, in contrast to some other claims.^410,411^

After a detailed discussion Stacey concludes in the indicated papers^398,399^ that only three expressions have built in the freedom to choose *K*_∞_′ and thus are particularly relevant to high-pressure work. These three are the generalized Rydberg equation, the Roy and Roy equation, and his own reciprocal *K*′ equation. He further argues that progress is limited by having insufficient data for, say, *P* > 30 GPa. Clearly, all these thermodynamic discussions are relevant to situations with really high pressure as occurring in the Earth’s interior, but they seem to be not well-recognized in the materials science community.

### 7.2 Lindemann-Based and Related Approaches

Several other approaches, either based on the Lindemann concept or based on thermodynamic arguments, exist and will be discussed in this section.

In an approach by Arafin et al.,^412^ the Lindemann relation *T*_mel_ = ξ^2^(*k*/3ℏ^2^)*mθ*_D_^2^*r*_0_^2^ is augmented by the use of the Grüneisen relation γ = ∂lnθ/∂ln*Ω*, in combination with the bulk modulus *K*_*T*_, leading to d(ln *T*_mel_)/d*P* = 2(γ – 1/3)/*K*_*T*_. Expanding both γ and *K*_*T*_ to second order in the pressure *P* evaluated at *P* = 0, and integrating, an explicit expression for *T*_mel_ was obtained (see also refs (413−415)). Experimentally for Li, K, Rb, and Cs, a maximum in *T*_mel_ as a function of *P* is observed, and calculating results for Li, K, and Rb, good agreement was found with the available experimental data, using both the first-order and second-order expansions.^416^ For Cs only the second-order expansion can describe the experimental data well, which may be not so surprising as Cs is a most compressible metal. Experimentally, the *T*_mel_–*P* curves for these metals show an inflection point above the maximum, which is often interpreted as a sign of a structural transformation. Synchrotron diffraction experiments for Na resulted in a maximum *T*_mel_ ≅ 1000 K at *P* ≅ 31 GPa and indeed suggest a transformation from BCC to FCC.^417^ For Na the same model^418^ yielded *T*_mel_ ≅ 961 K at *P* ≅ 31 GPa in reasonably good agreement with the experimental data.

However, as noted by Shanker,^419^ the second-order expression for γ and *K*′ leads to negative values at really high (infinite) pressure which is unphysical. The value γ = 1/2 is the minimum value for that pressure,^420,421^ a result based on Thomas–Fermi theory.^422^ The authors conclude that the second-order expansion in pressure cannot be used, and that in this way the Lindemann criterion cannot explain the maxima in the melting curves *T*_mel_(*P*). While the second conclusion is most likely correct in principle (see later), the first is not. As discussed in section 7.1, a second-order Taylor expansion evaluated at *P* = 0 can be used for a limited range only and a truncated power series of a function is an unsatisfactory approximation for extrapolation, in this case to infinite pressure, while a Padé approximant would be a much better choice. The maximum for Na can be explained using Lindemann’s law from θ_D_ as calculated from the phonon spectrum by ab initio density-functional perturbation theory via mapping on the Debye spectrum.^423^ Alternatively, the elastic constants can be used. It appeared that *C*_44_ shows a maximum at 43 GPa and thereafter decreases. Although the other shear characteristic (1/2)(*C*_11_ – *C*_12_) increases, it cannot compensate for the decrease in *C*_44_, so overall, the shear modulus first increases, shows a maximum, and thereafter decreases. This leads to the same behavior for θ_D_ in the same pressure range and an effective negative Grüneisen parameter above the maximum if θ_D_ is taken constant. This explains the results of Arafin et al.^412^ The melting curve, as derived from the elastic constant data, can be described well by the Kechin expression with the maximum at about 25 GPa. The maximum was thus not attributed to a BCC–FCC phase transformation, which occurred at 63 GPa.

This criticism was iterated by Ashwini et al.^424^ These authors offered another approach based on that, in the limit *P* → ∞, the ratio (*P*/*K*)_∞_ = 1/*K*_∞_′ with *K*′ = ∂*K*/∂*P* remaining finite. Using the expression for 1/*K*′ = 1/*K*_0_′ + [1 – (*K*_∞_′/*K*_0_′)], as first used by Stacey,^401^ and 1/γ = *A* + *BP*/*K* with *A* = 1/γ_0_ and *B* = *K*_∞_′(1/γ_0_ – 1/γ_∞_), as proposed by Shanker et al.,^425^ the authors eventually arrived after integration at
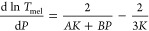
91

The parameter *K*_∞_′ was estimated using *K*_∞_′ = 3*K*_0_′/5,^398,426^ while for the γ_∞_ estimate γ_∞_ = (1/2)*K*_∞_′ – 1/6^398,400^ was used. From this expression using the Euler finite difference method the *T*_mel_(*P*) curves for 10 metals were determined, which are in reasonable agreement with experiment, with Al and Ag yielding the largest differences. Comparable results were obtained using the alternative expression
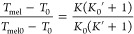
92with *T*_0_ a reference temperature as given by Kholiya and Chandra^427^ using the same parameters. The authors suggested that the discrepancies are possible due to the inadequacy of the Lindemann rule for some metals.^419^ Stacey obtained from thermodynamic considerations d ln *T*_mel_/d ln *V* = 2γ,^398^ which leads to *T*_mel_^–1^(d*T*_mel_/d*P*) = 2/(*AK* + *BP*). Solving this expression in the same way, the results for Cu, Mg, Zn, Au, and Ag agree to a large extent with experimental and ab initio data, while for Al the agreement is better than the results of both Ashini et al. and Kholiya and Chandra.

Clearly, the Grüneisen parameter γ plays an important role in pressure-dependent melting. In particular for transition metals, a proper estimate of the pressure dependence of γ is required. For example, the expression γ = γ_∞_ + γ_1_(*V*/*V*_0_)^1/3^ + γ_2_(*V*/*V*_0_)^*n*^, where γ_1_, γ_2_, and *n* > 1 are constants, was used by both Errandonea^428^ for Mo, Ta, and W and Burakovsky and Preston^420^ for 20 metals. Kushwah et al.^429^ used the only common metal, Mo, in these sets to calculate *T*_mel_ from the differential Lindemann relation d*T*_mel_/d ln *V* = −2(γ – 1/3) after inserting this expression for γ and integrating for both parameter sets. Comparing the calculated result with the experimental result^430^ shows that that Burakovsky–Preston estimate is far too high, while the Errandonea estimate fits the experimental data better (but see ref (431)).

Proposing a four-parameter EoS, Li et al.^432^ indicated (but not showed) that their EoS is capable of describing the *T*_mel_(*P*) curve with fairly satisfying results. Using *x* = (*V*/*V*_0_)^1/3^ this EoS is given by
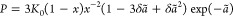
93and associated energy
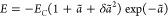
94

In these relations the dimensionless parameters
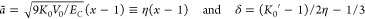
95are used, where, as before *K* = −*V*(∂*P*/∂*V*)_*T*_ and *K*_0_′ = (∂*K*/∂*P*)_*T*_. The expression for *P* and *E* reduces to that of Rose et al.^213^ if one takes δ = 0.05 and to that of Vinet et al.^410^ if one takes δ = 0. The latter two equations were derived from the so-called universal potential that was originally obtained from the scaled *E*(*r*) curve of several metals as obtained from ab initio calculations. This potential is given by *E*(a*) = *εϕ*(a*), where ε is the well depth and a* = (*r*_WS_ – *r*_WSE_)/*l* = *u*/*l* with *r*_WS_ the Wigner–Seitz radius, *r*_WSE_ the Wigner–Seitz radius at *P* = 0, and *l* = [ε/*E*″(*a*)_0_]^1/2^ = (ε/12π*r*_WSE_*K*_0_)^1/2^, and where it appeared that ϕ(a*) can be described by ϕ(a*) = −(1 + a* + 0.05a^*3^)exp(−a*).^213^ Li et al. showed for Lu a good agreement with the ab initio DFT *E*(*V*) curve and that ε, *V*_0_, *K*_0_, and *K*_0_′ all showed a rather constant value as a function of density, contrary to the values as obtained from the Rose or Vinet equation. The average δ-value (not given) for the 38 metals indicated is 0.021 (0.014) ± 0.080 (0.069) where “±” denotes the sample standard deviation and the numbers in parentheses refer to the same set without In as that metal has a substantially larger value, δ = 0.28. They also (only) indicated that a calculation for *T*_mel_ using the Guinea criterion for melting resulted in a good agreement with the experimental data. In a subsequent paper Li et al.,^433^ in which incidentally the authors surprisingly did not refer to their previous paper and which uses the same data and parameters, elaborated a bit. In this paper they show good agreement for the EoS and for ε, *V*_0_, *K*_0_, and *K*_0_′ with the ab initio data for Sr and Eu as well as for Lu. The melting criterion proposed by Guinea et al.^214^ states that melting occurs when the mean square displacement ⟨*u*^2^⟩^1/2^ exceeds the inflection point of the *E*(*r*) curve, i.e., at *E*″(*r*) = d^2^*E*(*r*_WS_)/d*r*_WS_^2^ = 0. This leads to *δã*^3^ – 6*δã*^2^ + 6*δã* + *ã* – 1 = 0, which the authors approximated by *ã* ≅ 1 – δ – 3.3δ^2^. Inserting this in the Debye expression for ⟨*u*^2^⟩^1/2^^8^ leads to the approximation *T*_mel_ ≅ 372(1–2δ)ε, where ε is in eV/atom. The agreement between calculated and experimental *T*_mel_-values is called “very satisfying”. A linear fit of *T*_mel,calc_ = *cT*_mel,exp_ (not given but calculated from their digitized picture) yields *c* = 0.933 with *R*^2^ = 0.983, very much in range with other correlations.

An important area of research where high-pressure melting studies are relevant is earth science, as experimentally only a fraction of the thickness of the Earth’s crust can be probed, while the lower lying shells, such as the mantle and core, cannot be probed, except with ultrasonic (seismic) waves. It seems that the solid inner core is crystallized from the liquid outer core, which is largely composed of Fe and Fe-rich alloys. Hence, for the core studies on Fe and its sulfur and oxygen compounds, as done, e.g., by Boehler^434,435^ and Sinmyo et al.,^436^ are particularly relevant, but for the mantle also studies on Mg–Si perovskite and magnesiowüstite are important.^435^ For further information on this interesting topic, we refer to the reviews by Stacey^398,400^ and the books by Anderson,^437^ Poirier,^10^ and Stacey.^438^

## 8 Molecular, Inorganic, Metallic, and Polymeric Solids

After having discussed melting along the line of mechanisms and the effect of pressure, we now take another brief look along the line of materials, including papers on experimental and calculational information for materials at high pressure without, however, providing a full review of this aspect.

### 8.1 Molecular Solids

Molecular solids comprise small molecules (atoms) that interact mainly via van der Waals interactions. The prototype of molecular solids are the rare gas crystals for which many papers have been published.

An early MD simulation study by Stillinger and Weber^439^ used 128 particles and periodic boundary conditions starting from a BCC configuration with as pair potential ϕ = *A*(*r*^–6^ – *r*) exp[(*r* – *a*)^−1^] for *r* < *a* and ϕ = 0 for *r* ≥ *a* with *A* = 3.809745436 and *a* = 2.0. This generic potential has a depth −1 and is zero at *r* = 1. For the BCC structure the minimum lattice energy *Φ*/*N* = −6.578015 at reduced density ρ* = 0.73051 for which melting occurs at *T*_mel_* ≅ 0.43. At low temperature the system is at one of the permutation-equivalent absolute minima, as expected, while at high temperature a rapidly changing sequence of positions results that predominantly correspond to amorphous random packings. From the results the elementary structural excitation out of the crystalline absolute minimum that leads to melting appeared to be the creation of a vacancy, split-interstitial defect pair. The system exhibits a defect-softening phenomenon, or mean attraction between defects, which influences the spectrum of normal-mode frequencies at the local minima and led to defect softening that was considered as basic to the fact that the solid–liquid phase transition is discontinuous.

Smirnov^112^ preferred a Morse potential above an LJ potential and, based on calculations for clusters of atoms with short-range interaction and closed shells, i.e. Ar_13_, Ar_55_, and Ar_147_, rejected the vacancy model.^440^ He proposed an alternative that describes melting as the interaction of structures. By limiting the discussion to FCC and icosahedral structures, he argued that the icosahedral structure is not favored at *T* = 0 because the nearest-neighbor distances differ from the optimal by about 2.5%. With increasing temperature, the vibrational amplitude increases, and when this increase approaches 2.5%, both structures can exist, and their “interaction” (I presume that coexistence is meant) leads to a local phase transition. Based on bond energy considerations for the aforementioned clusters without relaxation and taking into account nearest-neighbor interactions, he predicted the (scaled) melting temperatures of the rare gas solids in quite good (general) agreement with the simulation data in spite of the simplicity of the model. He argued that the change from an FCC cluster to an icosahedral cluster can lead to vacancies in view of the small differences in structure, but these were not further considered because relaxation was not considered. Transition between these structures, in his view, therefore, cause nonlinearity of the corresponding vibrations at relatively small energies of excitation and the creation of vacancies. He indicated that such a mechanism does not contradict (is consistent with) the results for Ar cluster simulations^441,442^ that indicate the phase transition of the clusters is not the softening of the surface layer but the collective motion of most surface atoms accompanied by the creation of vacancies in the surface layer. Finally, he made clear that additional evidence would be required, but no follow-up seems to have been published.

Using the interstitial defect approach, Robinson et al.^133^ discussed the rare gas solids, N_2_, CO_2_, CH_4_, and NH_3_, and some other compounds like halogens F_2_, Cl_2_, Br_2_, and I_2_, HCl and HI, and methanol. Using LJ potentials with parameters as given by Hirschfelder et al.,^443^ these authors used the modification of the interstitial approach given by Wentorf et al.^444^ which includes interactions to the third coordination shell and the approximation ψ = ψ_0_(*r*_0_/*r*)^12^ = ψ_0_(*V*_0_/*V*)^4^. Rewriting eq 33 as *Cu* = tanh *u* with *u* = (1/4)*βzψy*(2*X* – 1) with *C* = 4*kT*/*zψ*, this transcendental equation was solved graphically. The authors noted first that, for the compounds mentioned, but excluding HCl, CO_2_, CH_3_OH, and NH_3_, and which they considered as near perfect LJ gases, ψ_0_/*k* = 1.408*T*_mel_ at atmospheric pressure. The predicted *P*(*T*_mel_) curves for the rare gas solids are in excellent agreement with the experimental data available in the range of 8 kbar (Xe) to 25 kbar (Ar), and the principle of corresponding states was well obeyed. For N_2_ and CH_4_ the calculated *P*_calc_ data underestimate the experimental data by about 50 and 30%, respectively, at *P*_exp_ = 20 kbar, while for CO_2_ the curves cross at about 9 kbar. For N_2_ the authors advanced that the potential parameters may be different in the solid and gas states, that a “smeared” potential may not be warranted, and that polarizability was neglected. More generally, for the deviations from the corresponding states principle they argue that anisotropic molecules must be freely rotating in the solid state, that intermolecular forces must be weak, that the potential energy must be a function of distance only, and that the potential must be expressible as a conformal potential, i.e., ϕ = *εf*(*r*/σ), where ε and σ are scaling parameters for energy and distance, respectively. Overall, the authors commented that the interstitial model (1) contains long-range order for the liquid state and (2) neglects short-range correlation of neighboring molecules, in combination not providing an adequate picture. Finally, they suggested, as the proper LJ parameters may be different in the solid and gaseous states, that the ε-value should be taken as the value that yields the correct *T*_mel_ at atmospheric pressure, while the σ-value should be scaled by the value that represents the density of the liquid properly, both at atmospheric pressure.

Diamond anvil cell (DAC) experiments on the melting curve of Xe reported a clear flattening of the melting curve above 25 GPa.^445,446^ However, density functional calculations (DFT) show a steady increase of the melting curve of Xe with pressure. Shulenberger et al.^447^ attempted to resolve this discrepancy. They indicated that the cause of the discrepancy for conventional DFT calculations using (local) functionals, such as the local density approximation (LDA), might be due their overestimate of van der Waals interactions due to self-interaction of the electrons at low density, while more modern generalized gradient approximations (GGA) remove this self-interaction but lead to no binding energy at all. Hence, they argue that these significant theoretical challenges necessitate the application of a complementary technique whose approximations are not tied to the local behavior of electrons and considered diffusion quantum MC (DMC)^448,449^ a promising candidate. The method was benchmarked by calculating *T*_mel_ of Al at 120 GPa, as for this material at the chosen condition shock compression experiments, diamond anvil cell experiments, and DFT calculations all agree as to the melting temperature.^450^ Below 25 GPa their calculations on Xe showed agreement with experimental and calculational data, but above 25 GPa a steady increase resulted, in line with other theoretical calculations. They concluded that the shock compression high-pressure curve is well described by Lindemann behavior up to 80 GPa, in contrast to the diamond anvil results. However, the phase transformation between the FCC and HCP structures was not considered, in spite of previous suggestions.^445^

As already alluded to, DAC experiments are experimentally complex. Wiebke et al.^451^ indicate that difficulties with the extrapolation from DAC experiments are very well exemplified by substantial discrepancies in the melting curve of Fe above 100 GPa as relevant for the Earth core and lower mantle,^452^ but also that the situation is similar even for most simple substances such as Ar. Freezing to an FCC crystal at 83.8 K and ambient pressure, the melting curve of Ar has been established to follow a Simon law up to a few gigapascals (see, e,g., refs (369 and 445)). Beyond, however, lies a regime where data sets and extrapolated curves diverge.^453^ These authors used ab initio MC simulations under constant (*N*,*P*,*T*) conditions with periodic boundary conditions of the solid and liquid phases of 256 Ar atoms employing an accurate analytic many-body potential derived from rigorous relativistic electronic structure calculations. This implies that the solid superheats to a temperature *T* > *T*_mel_ before structural collapse occurs at the “critical superheating temperature” *T*^+^ well above *T*_mel_. This critical superheating temperature *T*^+^ was converted to *T*_mel_ via lim_*P*→∞_(*T*^+^/*T*_mel_) = 1 + 2 ln 2^1/3^ ≅ 1.231, as derived by Belonoshko et al.^338^ From simulations up to 100 GPa they derived the Kechin expression
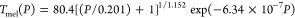
96(with *P* in GPa, estimated uncertainty of ±2.6% in *T*_mel_). At normal pressure the bulk *T*_mel_ = 80.4 ± 2.1 K is only slightly smaller than the NIST-recommended value of 83.8 ± 0.3 K.^454^ The small Kechin exponent of only 6.34 × 10^–7^ GPa^–1^ effectively renders their melting curve a Simon–Glatzel law essentially up to 100 GPa, thus obeying the PoCS. In fact, the deviations from corresponding-state behavior as observed by Boehler et al.^445^ have been suggested to be an experimental artifact.^455−457^ Quantitative agreement with an analysis of many experimental data by Ferreira and Lobo^458^ exists, for which as example the authors quote *T*_mel_ = 4409 ± 132 K at 100 GPa, to be compared with the extrapolated *T*_mel_ range of 4357–4451 K from the Ferreira–Lobo data.

Gal and Friedlander^459^ assumed that solid rare gases are quasi-harmonic Debye solids, for which in combination with the Lindemann–Gilvarry rule the melting temperature is given by *T*_mel_ = *CV*^2/3^θ_D_^2^ with *C* a material dependent constant. Using the Grüneisen parameter γ = −∂lnθ_D_/∂ln*V* and *V*_0_/*V* = ρ/ρ_0_, where *V*_0_ and ρ_0_ are a reference volume and density, respectively, integration leads to
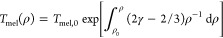
97where *T*_mel,0_ is the melting temperature at the reference density. The assumption γ = γ_0_(ρ_0_/ρ)^*q*^, with *q* = 1 and γ_0_ the γ-value at ambient conditions, leads to
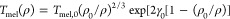
98

This expression states that if ρ(*P*), *T*_mel,0_, and γ_0_ are known, the melting curve *T*_mel_(*P*) can be determined assuming that the relation between *P* and ρ is known. For this relation the authors used what they called the “well accepted” expression:
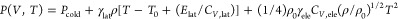
99where *P*_cold_ is the cold pressure, *C*_*V*,lat_ is the lattice specific heat above the ambient temperature *T*_0_, *C*_*V*,ele_ is the electronic specific heat, *E*_lat_ is the lattice energy at *T*_0_, and γ_lat_ and γ_ele_ are the lattice and electronic Grüneisen parameters, respectively. Here *P*_cold_ is the pressure applied at room temperature, while the other terms represent the thermal pressure to reach *T*_mel_. For ρ(*P*_cold_), one uses, e.g., the Murnaghan, Birch–Murnaghan, or Vinet equation from which values for the bulk modulus *K*_0_ and its pressure derivative *K*_0_′ at room temperature can be obtained. However, the fitted values are nonunique and depend on the EoS used, thereby explaining the often rather widely varying results by different authors.

Using this approach and demanding that bulk modulus parameters will simultaneously fit the equations of state and the melting curves of solid rare gases, the authors obtained for He, Ne, Ar, Xe, and Kr values for *K*_0_ typically a factor of 3 higher and values for *K*_0_′ generally a factor of 2 lower than the literature data. Unfortunately, the best fit obtained depends on the EoS used, which varies with the type of compound. While for Ne and Kr the Murnaghan expression provided the best fit, for Ar it was the Birch–Murnaghan expression, and for Xe it was the Vinet expression.

Further, these authors discussed the solid-state phase transformation for the rare gas solids. With increasing pressure, He, Ar, Xe, and Kr exhibit a discontinuous crystallographic phase transformation from FCC to HCP structures at about 10, 20, 25, and 20 GPa, respectively, with Ne being an exception maintaining the FCC phase up to 208 GPa. For Ar, Kr, and Xe the explanation for this transformation relates to the difference in electronic structure from that of Ne, as the energy band gap of the latter between the filled 2p-valence states and the 3d-conduction state is large. This difference is also probably the cause why the principle of corresponding states (PoCS) based on data for Ne is not obeyed for Kr and Xe for pressures above about 30 and 20 GPa, respectively. Such an explanation was also already advanced by Boehler et al.,^445^ but they include Ar as well for *P* > 40 GPa. In the latter case different experimental data were included, however. For He the melting curve shows unexpected linear behavior up to at least ∼100 GPa, which could be explained by van der Waals attraction in balance with the Coulomb repulsion. However, such an argument neglects the 1s^2^ electrons, which partially penetrate the nucleus and are affected by the increasing pressure. The authors suggest that this is perhaps the reason why solid He under extreme pressures will not exhibit the insulator-to-metal transition.

The effect of pressure on the atomic mean square displacement, extended X-ray absorption fine structure (EXAFS) Debye–Waller factor, and *T*_mel_ of solid krypton were investigated in within the statistical moment method scheme in quantum statistical mechanics. The statistical moment technique essentially expands the potential in terms of the displacements *u*_*j*_ to fourth order which in combination with force balance results in a differential equation for the first moment of the displacement *y*(*T*) = ⟨*u*_*j*_⟩_*p*_,^460−462^ which reads *γθ*^2^ d^2^*y*/d*p*^2^ + 3*γθy* d*y*/d*p* + *αy* + *γθ*(*X* – 1)/α – *p* = 0. Here θ = *kT*, *x* = ℏω/2θ, and *X* = *x* coth *x*; *p* is a supplementary force acting on the zeroth central atom in the lattice due to the thermal lattice vibration effects, α is the harmonic, and γ is the anharmonic force constant. The solution of this differential equation provides the average atomic displacement *y*(*T*) = *y*_0_(*T*) + *A*_1_*p* + *A*_2_*p*^2^, where the functions *y*_0_, *A*_1_, and *A*_2_ are somewhat long expressions given in detail by the authors, which take into account the anharmonicity effects of thermal lattice vibrations at temperature *T*.^463,464^ The average nearest-neighbor distance between two atoms at temperature *T* can be calculated from *r*(*T*) = *r*_0_ + *y*_0_(*T*), where *r*_0_ is the value of the nearest-neighbor distance at 0 K. In combination with a modified Lindemann rule,^465^ this approach yields a relatively simple method for qualitatively calculating the high-pressure melting temperature. By assuming that the interaction between the Kr atoms can be described by the Buckingham potential, numerical calculations for Kr up to pressure 120 GPa were done. The calculations show that the atomic mean square displacement and EXAFS Debye–Waller factor of Kr crystal depend strongly on pressure and the results are in reasonable agreements with available experimental data.

For molecular compounds two calculational papers follow the interstitial approach, as commented on by Tozzini et al.^136^ (section 5.5). First, some further calculational results on NaNO_3_ were given by Akdeniz and Tosi,^466,467^ indicating that increasing *n* lowers both transition temperatures. For NaNO_3_ the number of orientations *n* = 4, and taking *T*_cri_/*T*_mel_ ≅ 0.95 from experiment, they obtained *z*′ε′/*zε* ≅ 0.55. At such values of the reorientation energy barrier, the two disordering transitions are very close to each other, and the authors expected that orientational disordering strongly influences the melting process.

Second, Matthai et al.^468^ attempted to explain the melting behavior of AX_4_ compounds like GeI_4_, SnI_4_, and CCl_4_. Their model is based on experiments that indicate that structural disorder in GeI_4_ sets in at about 12 GPa, accompanied by a molecular association process,^469^ on experiments that show for SnI_4_ that a crystal to amorphous transition takes place at about 8 GPa^470^ accompanied by a drastic reduction in its resistivity and (for both GeI_4_ and SnI_4_) suggesting metallization under high pressure with the SnI_4_ molecules forming dimers.^471^ Both the Simon–Glatzel and Kechin equations (section 7.1) can describe this transition well up to about 2.5 GPa. For pressures above this critical pressure, the data are not well described by either of these equations. A significant finding was the existence of two liquid states with the transition between the two characterized by a change in the density.^470^ In the structural model for the AX_4_ molecular solids under pressure proposed, there is a breakdown of the structural order with increasing pressure, as observed by Fujii et al.^470^ and consistent with data on CCl_4_.^472^ As the pressure is increased further, the X–X bonds between adjacent AX_4_ molecules become stronger and the amorphous structure comprises disordered AX_4_ molecules in equilibrium with polymer chains. As the pressure is increased still further, the ratio of polymer chains to the free molecules increases until all the isolated molecules become polymerized. The solid–liquid transition on increasing temperature results in two liquid phases differentiated by density. At low pressures well below the amorphous transition pressure, melting is from a crystalline phase, as described by the Simon–Glatzel or Kechin equation. At higher pressures, the solid becomes a polymeric solid which transforms to liquid at roughly the same temperature, independent of pressure, and *T*_mel_ is determined solely by the energy required to break the interpolymer bonds. As this energy is independent of *P*, it results in a flattening of the melting curve. The polymerized liquid state is of higher density than the monomeric liquid state, while the polymerization results in increased electrical conductivity.

The behavior of SnI_4_ was examined experimentally by Fuchizaki^473^ as well, showing that the low-pressure crystalline phase of SnI_4_ has a rising melting curve that breaks abruptly around 1.5 GPa, beyond which it becomes almost flat, with a slight maximum at about 3 GPa. The Kraut–Kennedy relationship,^378^ stating on an empirical basis that *T*_mel_ of a substance is proportional to Δ_0_*V*/*V*_0_ at room temperature, where Δ_0_*V* = *V*_0_ – *V* with *V* measured along the room temperature isotherm, appears to be valid in the low-pressure region where the melting curve is rising. Similarly, the Magalinskii–Zubov relationship^474^ appears to be valid in the low-pressure region where the melting curve is rising. This criterion states that *V* of a substance along a solidus is proportional to ln(*T*_mel_) and is derived from a generalized melting law using constant excess entropy along the melting line and assuming that the excess internal energy of the solid is constant along the solidus. Their breakdown at larger pressures suggests, according to the author, a qualitative change in the intermolecular interaction upon compression, thereby making the melting behavior unusual.

In relation to “Lindemann–Gilvarry–Grüneisen-type” models, it may be useful to refer to a possible redefinition of the mode Grüneisen parameter for polyatomic substances by Hofmeister and Mao.^475^ The authors pointed out that the thermally average mode Grüneisen parameter ⟨*γ*_*j*_⟩ = −⟨∂ln*ν*_*j*_/∂ln*V*⟩ = (*K*_*T*_/*ν*_*j*_)(∂*ν*_*j*_/∂*V*) is up to 25% lower that the thermodynamic parameter γ_th_ = *αK*_*T*_*V*/*C*_*V*_. For example, for γ-Mg_2_SiO_4_, the IR modes give ⟨*γ*_*j*_⟩ = 0.96,^476^ which is substantially lower than γ_th_ = 1.25 as calculated from thermodynamic data.^477^ The longitudinal acoustic (LA) and transverse acoustic (TA) modes have associated Grüneisen parameters given by
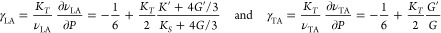
100and the average γ_ac_ = (γ_LA_ + 2γ_TA_)/3 can be defined. In a rigorous determination, the average should include both acoustic and optic modes and be averaged over the complete Brillouin zone, but the acoustic modes do not contribute to the zone center sum. Because of the limited availability of dispersion data for more complex solids, the Grüneisen parameters of the acoustic modes, which can be calculated using the Debye model, have been postulated to represent the thermodynamic average.^478^ In the Debye model the acoustic modes are used to represent all vibrational energy. The mode Grüneisen parameters of longitudinal acoustic modes for many solids resemble γ_th_, whereas the Grüneisen parameters of the transverse acoustic modes do much less so. This departure of γ_ac_ from γ_th_ was attributed to failure of the Debye model for some classes of solids.^478^ In the literature the failure of the Debye model for γ-Mg_2_SiO_4_ has further been attributed to discrepancies between the averages of *γ*_*j*_ and γ_th_,^477^ which, however, cannot be true because the calculation for the optic modes is unrelated to the Debye model. Instead, in its simplest form the optic modes are represented by an Einstein-type dispersion-free model, that, at least for γ-Mg_2_SiO_4_, appeared to be insensitive to slightly different weighing. The authors realized that discrepancies arose because −∂ln*v*_*j*_/∂ln*V* does not account for differential compression in structures with functional groups. Consequently, they redefined −∂ln*v*_*j*_/∂ln*V* by −∂ln*v*_*j*_/∂ln*V*_a_, = (*K*_*X*_/*ν*_*j*_)(∂*ν*_*j*_/∂*V*_a_), where where *K*_*X*_ is the bulk modulus associated with the volume vibrating *V*_a_. For monatomic solids and many structures with two types of atoms, *K*_*X*_ = *K*_*T*_, but for many of the optic modes in polyatomic structures, *K*_*X*_ will be a polyhedral bulk modulus: *V*_a_/[d*V*_a_/d*P*].^479^ From an extensive survey of minerals, the authors showed that ⟨*γ*_*j*_⟩ ≅ γ_th_ and concluded that the volume of the vibrating unit is relevant to the mode Grüneisen parameter, and not the volume of the whole crystal, as also supported by approximate rough agreement with γ_LA_. Close correspondence is not expected anyway because the Debye model is not rigorous.

For somewhat larger molecules various nonintrinsic factors usually influence the melting behavior significantly. For example, sugar crystals^480^ show melting that often occurs at low temperatures with time- and temperature-dependent characteristics, which can be accounted for by the presence of impurities and defects. Sugar crystals also contain noncrystalline regions that may undergo decomposition and subsequent dissolution at the decomposition interface and acceleration of decomposition reactions. Such processes with melting establish a supersaturated condition for the remaining crystals, leading to a time-dependent melting point depression and subsequent melting of the remaining crystals. Decomposition of sugars as well as dissolution and melting of sugar crystals are separate phenomena, although they are commonly found to coincide. Decomposition of sugars requires the presence and mobility of molecules for reactions outside the crystal lattice, i.e., the molecular mobility of amorphous or molten regions is a prerequisite for decomposition, whereas melting of sugar crystals occurs as a separate thermodynamic process with no chemical change of the molecules.

### 8.2 Inorganic Solids

For melting of inorganic solids the use of empirical pair potentials has been pursued for over a century.^481^ Frequently the Born–Landé potential ϕ = *A*/*r* + *B*(*r*_0_/*r*)^*n*^ or the Born–Mayer potential ϕ = *A*/*r* + *B* exp[−(*r* – *r*_0_)/ρ] is used, often extended with van der Waals terms *C*/*r*^6^ and *D*/*r*^8^ (Born–Mayer–Huggins) and sometimes with three-body interactions. As this is a one-phase approach, it must be augmented with a melting criterion. As mentioned already, often the Ross criterion assumes a constant configuration along the binodal, or equivalently, assumes a constant scaled excess Helmholtz energy *F*. This is expressed by
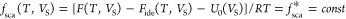
101where *F*_ide_ is the perfect gas Helmholtz energy and *U*_0_ is the potential energy of the static lattice. Another criterion is a constant configurational entropy *S* along the melting curve, as assumed by Stishov^13^ and Magalinskii and Zubov,^474^ and expressed by
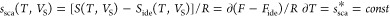
102

Such constancy was proposed by Stishov^482^ and discussed by Tallon.^483^ Still another criterion is assuming a constant potential energy *U* given by
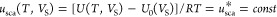
103Clearly, the relation *f*_sca_ = *s*_sca_ + *u*_sca_ holds.

An elaborate attempt along these lines was made by Soulayman^484,485^ for several halides using the formalism by Zubov^486^ for describing strong anharmonic monatomic crystals, each ion of a crystal being described by its one-particle probability density, distinct from those of the other atoms. In thermodynamic equilibrium, the spatial parts of the one-particle functions obey a set of nonlinear integral equations^487^ that must be solved numerically. Born–Mayer–Huggins potentials with parameters as given in the literature, and extended by three-body interactions,^488,489^ were used. The contributions to the Helmholtz energy *F* were taken to be a harmonic term *F*_0_, a perturbation theory based anharmonic term *F*_1_, and a first-order quantum correction term *F*_Q_.

Alkali halides (KCl, KBr, KI, RbCl, RbBr, Rbl, and CsCl) have two crystalline modifications: a low-pressure structure (NaCl structure) and a high-pressure one (CsCl structure). Some alkali halides change their structures at relatively low pressures, for example, KCl at about 20 kbar. Others, like NaCl and CsCl, do not show a transformation up to 100 kbar. To assess the configuration, entropy, and energy criteria, the equilibrium nearest-neighbor distance *a* for the crystalline phase versus the experimental (*T*_mel_)_exp_ at the experimental melting pressure (*P*_mel_)_exp_ was obtained numerically by solving the pressure equation *P* = −(*a*/3*V*)(∂*F*/∂*a*)_*T*_ along the experimental melting curve in relation to the volume *V* of the crystalline phase. The equation for *P* has two real roots: *a*_1_(*T*_mel_,*P*_mel_) < *a*_2_(*T*_mel_,*P*_mel_). The lower root represents the stable thermodynamic solution, (∂*V*_1_/∂*P*)_*T*_ < 0, while the upper root represents the unstable one, (∂*V*_2_/∂*P*)_*T*_ > 0. To obtain these roots, the equations for the chemical potentials μ_1_(*T*,*P*) = μ_2_(*T*,*P*) and pressures *P*_1_ = −(*a*_1_/3*V*_1_)(∂*F*_1_/∂*a*_1_)_*T*_ and *P*_2_ = −(*a*_2_/3*V*_2_)(∂*F*_2_/∂*a*_2_)_*T*_ were solved numerically at *T* = 298 K, taking the stability criteria (∂^2^*F*_1_/∂*a*_1_^2^)_*T*_ > 0 and (∂^2^*F*_2_/∂*a*_2_^2^)_*T*_ > 0 into account. The *V*(*P*) curves calculated up to 100 kbar showed excellent agreement with the experimental data, available up to about 45 kbar, including the phase transformation for KCl at about 20 kbar, in agreement with experimental data. For both NaCl and CsCl no phase transformation resulted. As Wallat and Holder^490^ concluded that the experimental data on phase transitions demand larger values of the potentials than the traditional sets and Shanker and Agrawal^491^ indicated the significant role of van der Waals potentials in studying the structural phase transitions of ionic crystals, the van der Waals interactions were included using the London–Margenau formulation.^492^ The *P*_mel_(*T*_mel_) melting curves, which were calculated without three-body interaction for CsCl using the energy criterion, showed excellent agreement with the experimental data, while the configuration and entropy criteria agreed much less. Accordingly, for NaCl, NaBr, NaI, and KCl the melting curves were calculated with the energy criterion, which all showed excellent agreement with the experimental data.

Another approach is using the EoS, as done by Shanker et al.^493^ Expanding the lattice potential *U* to third order in the volume expansion *x* = *V* – *V*_0_ with *V*_0_ the volume at the reference temperature *T*_0_ = 300 K, and using the pressure equation *P* = −d*U*/d*V* + Δ*P*_the_ with Δ*P*_the_ the vibrational energy, the thermal EoS
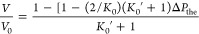
104was derived. Here *K*_0_ and *K*_0_′ are the bulk modulus *K*_*T*_ and its pressure derivative d*K*_*T*_/d*P* at *T* = *T*_0_ and *P* = 0. This expression shows good agreement with experimental data for some minerals^494^ and alkali halides.^495^ For Δ*P*_the_ > *K*_0_/2(*K*_0_′ + 1), eq 104 yields imaginary values and the corresponding temperature is interpreted as the melting temperature *T*_m0_ at *P* = 0. Anderson^478^ has shown that above the Debye temperature θ_D_ the approximation Δ*P*_the_ = *C*(*T* – *T*_0_) with *C* = *αK*_*T*_ holds good. Moreover, it was shown that for higher pressures Δ*P*_the_ in eq 104 should be replaced by Δ*P*_the_ – *P*,^495^ leading to Δ*P*_the_ = *P* + *K*_0_/2(*K*_0_′ + 1). Alternatively, it was assumed that *V*/*V*_0_ can be calculated from *K*_*P*_ = *K*_*T*_(*P*,*T*_0_) and *K*_*P*_′ = d*K*_*T*_(*P*,*T*_0_)/d*P* instead of *K*_0_ and *K*_0_′. Combining these results, one obtains, respectively
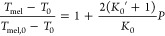
105-1and
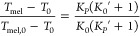
105-2

The values for *C* for NaCl, KCl, MgO and CaO appeared to agree with the experimental data given by Anderson.^478^ While eq 105-1 predicts a much faster increase than experimental data indicate, eq 105-2 yields satisfactory results. In particular for NaCl for which the linear dependence on pressure is quite small, *T*_mel_ = 1329 K at Δ*V*/*V*_0_ = 0.04 and *T*_mel_ = 1983 K at Δ*V*/*V*_0_ = 0.12, to be compared with the experimental values extracted from the data by Anderson, 1300 and 1950 K, respectively. Finally, for NaCl the ratio Δ*V*/*V*_0_ ≅ 15% at *T*_mel_, consistent with results from simulation studies and empirical potentials.

Using the relation between temperature and thermal pressure, Wang et al.^496^ developed a simple model to estimate *T*_mel_. The pressure *P*(*V*,*T*) can be divided in a static part, *P*(*V*,0), and a thermal part, *P*_the_, and assuming that *αK*_*T*_ = *const*., the increase in thermal pressure Δ*P*_the_ can be calculated from
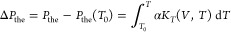
106or, assuming that *P*_the_ is independent of *V*, as made plausible by Anderson,^478^ that Δ*P*_the_ = α*K*_*T*_(*T* – *T*_0_), where the overbar denotes the average over the range, and, hence, *T*_mel_ = Δ*P*_the_/α*K*_*T*_ + *T*. For Δ*P*_the_ the expression, as given by Anderson,^478^ was used.
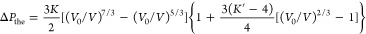
107

**Figure 21:**
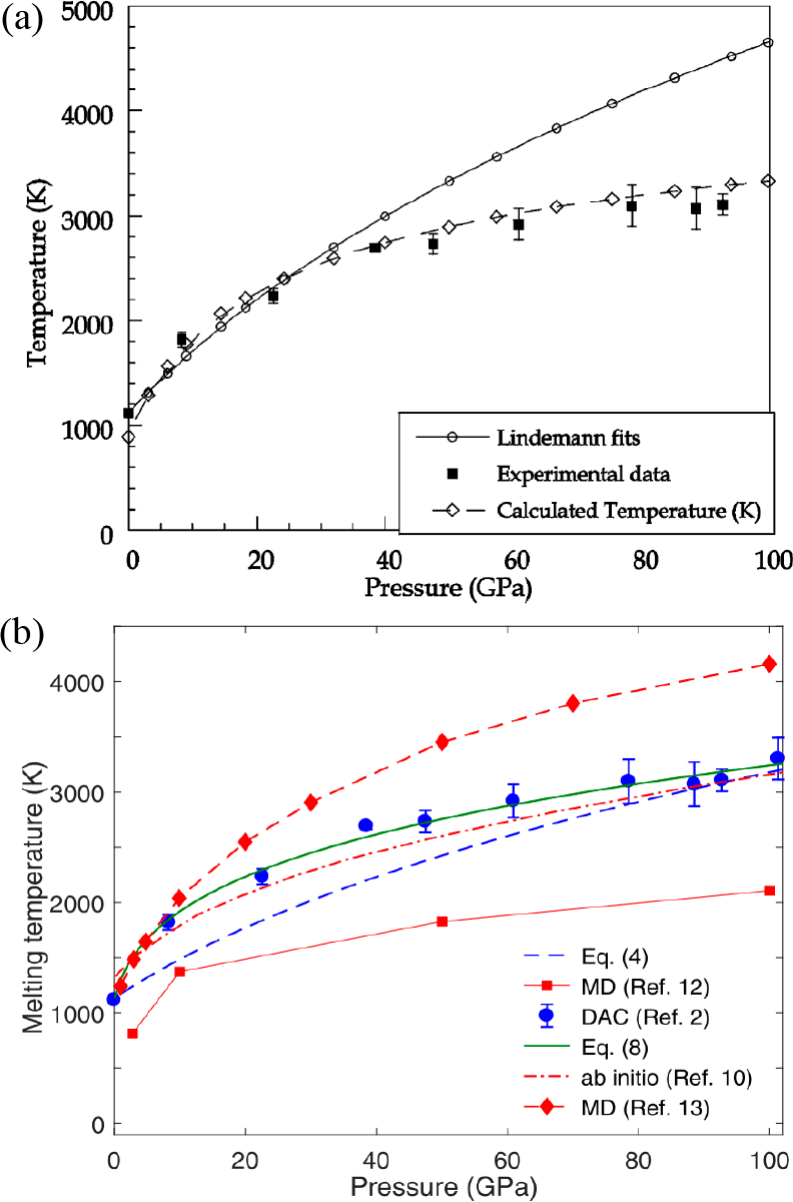
(a) The melting curve of LiF according to Wang et al.^496^ (dotted line) in comparison with experimental results of Boehler et al.^497^ (◇) and Jackson^498^ (■), as well as the prediction by the Lindemann rule. Reproduced with permission from ref (496). Copyright 2001 Elsevier. (b) The melting curve of LiF according to Nghia et al.^499^ (eq 8), the Lindemann extrapolation (eq 4), and two experimental and two calculational results. Reproduced with permission from ref (499). Copyright 2020 Elsevier.

Because LiF remains in the B1 (NaCl) structure up to at least 100 GPa, this material provides a nice test over a wide pressure range without a phase transformation interfering. The calculated result for *T*_mel_ shows excellent agreement with the experimental data (Figure 21a). Also shown is the Lindemann extrapolation based on low-pressure data, Slater’s Grüneisen parameter, and the assumption γ_0_ρ_0_ = *γρ*, showing that only below about 25 GPa the experimental data are reasonably described, albeit with a curvature less than that for the experimental data. Results for FeO and CaMg_2_Si_2_O_6_ were also calculated, but these compounds show a phase transformation at, respectively, 20 and 18 GPa. Below these pressures, *T*_mel_ is predicted correctly as well.

Based on lattice potential calculations using the Born–Landé and Born–Mayer potentials with parameters derived by Smith and Cain^500^ using Hildebrand’s EoS *P* = −d*Φ*/d*V* + *αTK*_*T*_ and ultrasonic data for thermoelastic properties, Chauhan and Singh^501^ calculated values for the interionic distances *r*_mel_ at the melting temperature *T*_mel_ for 16 alkali halides. This distance is given by *r*_mel_ = (*r*_inf_ + *r*_inf_′)/2, where *r*_inf_ is the distance for the inflection point of the potential curve ϕ and *r*_inf_′ is the distance corresponding to ϕ(*r*_inf_′) = ϕ(*r*_inf_). For the Born–Landé and Born–Mayer potential the values for *r*_inf_ are given by
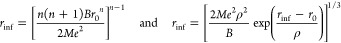
108respectively, with *M* = 1.7476 the Madelung constant for NaCl- or B1-type crystals and *e* the unit charge, while the values for *r*_inf_′ are obtained from solving ϕ(*r*_inf_′) = ϕ(*r*_inf_). Another way to determine *r*_mel_ was the use of the Anderson expression α = α_0_[1 – α_0_*δ*_*T*_(*T* – *T*_0_)]^−1^,^478^ valid for *T* > θ_D_. Assuming that *αK*_*T*_ = *const*. leads to (α/α_0_) = (*V*/*V*_0_)^*δ_T_*^ = (*r*/*r*_0_)^3*δ_T_*^, so that the interionic separation is
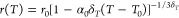
109which was shown to hold good for alkali halides^502^ close to *T*_mel_. The correspondence between these two estimates is excellent. For the Born–Landé potential *r*_mel,A_ = *c*_BL_*r*_mel,BL_ with *c*_BL_ = 1.0033 and *R*^2^ = 0.999977, while for the Born–Mayer potential *c*_BM_ = 1.0006 and *R*^2^ = 0.999978 (calculated from the data). The largest deviation occurs for LiF, for which θ_D_ = 751 K is much higher than room temperature and therefore the assumptions used do not hold well. With the Hildebrand EoS, written for *P* = 0 as d*Φ*/d*V* = 6*r*^2^*αTK*_*T*_, the Born–Landé and Born–Mayer potentials at *T* = *T*_mel_ with *r* = *r*_mel_ are
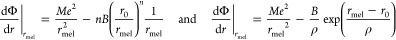
110respectively. The assumption *αK*_*T*_ = *const*. appeared to be fulfilled reasonably well with (*αK*_*T*_)_BL_ = *c*_BL_(*αK*_*T*_)_RT_, where *c*_BL_ = 1.1563 and *R*^2^ = 0.9898 for the Born–Landé potential and *c*_BM_ = 1.1428 and *R*^2^ = 0.9887 for the Born–Mayer potential (calculated from the data).

Chauhan et al.^503^ compared the derivative *T*_mel_^–1^(d*T*_mel_/d*P*) as calculated from the differential Lindemann–Gilvarry model and Stacey–Davis model, given by
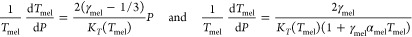
111respectively, where the symbols have their usual meaning, for the same 16 alkali halides minus LiI and LiBr. This led to a poor correlation for both: [*T*_mel_^–1^(d*T*_mel_/d*P*)]_LG_ = *c*_LG_[*T*_mel_^–1^(d*T*_mel_/d*P*)]_exp_ with *c*_LG_ = 1.4277 and *R*^2^ = 0.9253 for the Lindemann–Gilvarry model and *c*_SD_ = 0.9726 and *R*^2^ = 0.9439 for the Stacey–Davis model (calculated from the data).

Using *q* = (∂lnγ/∂ln*V*)_*T*_, Nie^504^ used *q* = *q*_0_*η*^*n*^, where η = *V*/*V*_0_,, and obtained γ = γ_0_ exp[*q*_0_(*η*^*n*^ – 1)/*n*]. From data for NaCl at 300 K (up to 3 GPa), 550 K (up to 1 GPa), and 800 K (up to 1 GPa), he obtained *n* = 1, but as indicated in parentheses for relatively low *P*. Kumar et al.^505^ pointed out that the expression for γ does not satisfy the boundary condition for *P* → ∞. They modified the γ expression by inserting the high-pressure limit γ_∞_ = γ_0_ exp(−*q*_0_/*n*) with the γ_∞_ estimate γ_∞_ = (1/2)*K*_∞_′ – 1/6,^398,400^ where *K*_∞_′ = (∂*K*_*T*_/∂*P*)_*T*_ = 5/3 is used with as result that γ_∞_ = 3/2. This replaces *q*_0_/*n* by ln(γ_0_/γ_∞_), and this expression described the experimental volume dependence of γ down to η = 0.64 for NaCl well (as well as for Fe down η = 0.60).

Sheelendra and Vijay^506^ also studied the EoS, thermoelastic properties, and melting behavior of NaCl at high temperatures and high pressures, but used somewhat more acceptable expressions for γ. From the Lindemann–Gilvarry differential rule, d ln *T*_mel_/d ln *V* = −2(γ_mel_ – 1/3), in combination with
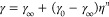
112-1or
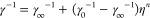
112-2due to refs (507) and (508), respectively, and where η = *V*/*V*_0_, the authors obtained by integration
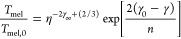
113-1or
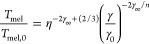
113-2

Here *n* is a constant (*n* = 2.47 for eq 112-1 and *n* = 2.0 for eq 112-2 in the case of NaCl), the subscripts “0” and “∞” represent the values at zero pressure and in the limit of infinite pressure, respectively, and λ_∞_ = (*K*_∞_′)^−1^[*KK*′/(1 – (*K*′*P*/*K*)]_∞_ + *K*_∞_′. The latter parameter follows from Stacey’s reciprocal *K*′ EoS.^401^ The calculated results for both equations are close, but they underestimate *T*_mel_ for *P* ≲ 11 GPa and overestimate *T*_mel_ for *P* ≳ 11 GPa.

Similarly, Nghia et al.^499^ studied LiF using also the Lindemann–Gilvarry formulation, but in combination with γ = −(1/2)(d ln μ/d ln *V*) – 1/6, as given by Burakowsky et al.,^509^ and γ/γ_0_ = *η*^*n*^, with again η = *V*/*V*_0_ and *n* > 0 a parameter. Integration leads to
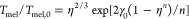
114Using *n* = 0.842 as obtained by Liu et al.^510^ by fitting experimental shock wave data, this expression underestimated *T*_mel,exp_ (Figure 21b). The experimental data are quite well described by the Simon–Glatzel equation in the form *T*_mel_ = *T*_mel,0_[1 – (*P*/*a*)]^*b*^, with *a* = 1.2306 and *b* = 0.2384, in the available range of 0–100 GPa. For LiF, ab initio path integral MC and DFT MD calculations by Driver and Militzer^511^ yielded a more close agreement with experiment but still underestimate *T*_mel_ typically by some 50–100 K. Comparing Figure 21a with Figure 21b, one notices a rather different prediction based on the same premises (Lindemann rule + Slater’s γ + γ/γ_0_ relation). For binary oxides calculations similar to those by Nghia et al.^499^ have been done, e.g., for MgO,^512^ combining d ln *T*_mel_/d ln *V* = 2(1/3 – γ) with eq 112-2, resulting in good agreement of *T*_mel_(*P*) up to 50 GPa with experimental data using *n* = 2.2.

For binary oxides also simulation studies for high pressures have been done, such as for MgO^513^ using density DFT calculations in the LDA in combination with thermodynamic integration, for CaO^514^ using shell-model MD calculations with Born–Mayer–Huggins potentials employing thermal instability analysis. Also, for more complex oxides studies have been made, e.g., for pyrope (Mg_3_Al_2_Si_3_O_12_)^515^ using the Hartmann EoS,^516,517^ as often used for polymers.

### 8.3 Metallic Solids

As for most materials, various approaches have been used for metals. Most of the studies on individual metals deal with pressure dependence. In Table 5 studies for several metals are enumerated, briefly indicating the method used, just to show the variety of methods used, while Parisiades^518^ reviewed the melting curves of transition metals at high pressure using static compression techniques. Hence, we limit the discussion to papers that illustrate the different approaches followed. We first discuss equation of state models and thereafter theoretical models and simulations.

**Table 5:** Some Studies on Metals^a^

metal	method	*P*-range (GPa)	ref
Al	L + Debye + *αK*_*T*_ = *const*.	77	(522)
Al, Cu, Ni	DFT + quasi-harm. lattice dynamics	∼100	(523)
Al, Ni, Pt	simple model + thermal pressure eq.	120	(524)
Au	laser-heated DAC + synchrotron XRD	110	(525)
Cd	DAC + in situ XRD	10	(526)
Cd, In, Sn, Th, U	L + γ(*P*) + third-order Birch–Murnaghan EoS	140	
Co, Cr, Mo, Ni, Ta, Ti, V	laser-heated DAC	100	(527)
Cu	EAM potential MD	200	(339)
Cu	L + γ(*P*)	100	(528)
Cu, Mn, Ni, Pd, Pt	L + statistical moment method	140	(465)
Cu, Fe, Ni	theoretical using other data		(529)
Cu, Ni, Pd, Pt	DAC	(Cu, Ni) 60, (Pd, Pt) 30	(530)
Cu, Ni, Pd, Pt	L + γ(*V*)	70	(531)
Cu, Ni	laser-heated DAC	Cu 96, Ni 60	(532)
Fe	L + γ(*P*)	350	(528)
Fe	L + moment exp of anharm. Helmholtz energy	350	(533)
Fe	statistical moment method	360	(534)
Fe	in situ XRD + nuclear reson. inelastic X-ray scatter.	171	(535)
Na	L + γ(*P*)	65	(536)
Na	ab initio MD	120	(537)
Mo	laser-heated DAC + synchrotron radiat.	∼100	(538)
Mo, Ta	theoretical using other exptl data	100	(539)
Nb, Ta, V	ab initio electronic calc.	100	(540)
Ni, Pd, Pt	L + statistical moment method + defects	100	(541, 542)

a L = Lindemann; DAC = diamond anvil cell.

#### 8.3.1 Equation of State Models

An early somewhat general discussion on alkali metals was given by Makarenko et al.^519^ The authors emphasized the similarities between rare gas solids and the alkali metals, although for the metals the “rule” Δ*S* ≅ *const*. for *P* → ∞ is approximately obeyed but the “rule” Δ*V*/*V*_S_ = *const*. for *P* → ∞ is not, while for the rare gas solids both are approximately fulfilled.

For Na, Hieu^520^ presented an analysis for volume and pressure effects on thermodynamic quantities including the Grüneisen parameter and *T*_mel_ of up to 65 GPa. Combining the result of Burakovsky et al.,^521^ γ = 1/2 + γ_1_η^2/3^ + γ_2_*η*^*q*^ with γ_1_, γ_2_, and *q* constants and *q* > 1, and the differential Lindemann–Gilvarry criterion d ln *T*_mel_/d ln *V* = 2(1/3 – γ) leads upon integration to
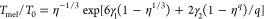
115where η = *V*/*V*_0_ and *T*_0_ (*V*_0_) is the ambient temperature (volume). The constants γ_1_, γ_2_, and *q* were fitted to the *T*_mel_(η) data by Boehler^543^ up to 3 GPa, which led to γ_1_ = 0.4801, γ_2_ = 0.2291, and *q* = 13.7253. For transforming the pressure data, Vinet’s EoS *P* = 3*K*_0_η^–2/3^(1 – η) exp[3(*K*_0_′ – 1)(1 – η^1/3^)/2] (ref (410), section 7.2) with *K*_0_ the bulk modulus and *K*_0_′ = d*K*_0_/d*P* was used employing *K*_0_ = 5.35 GPa and *K*_0_′ = 5.0 as taken from the literature. Up to 30 GPa the final expression matches the experimental data well but is incapable of showing the maximum in *T*_mel_ at about 35 GPa and its decrease with increasing pressure thereafter. The experimental data for Na were fitted by Arafin and Singh^418^ yielding *T*_mel_(*P*) = 417.6186 + 33.5913*P* – 0.5883*P*^2^ + 0.002511*P*_3_, which exhibits a maximum *T*_mel_ ≅ 976 K at *P* ≅ 38 GPa. Hieu and Ha^544^ also studied Ag, Au, and Cu along the same line as for Na with also reasonable agreement as a result.

Sheelendra and Vijay^531^ used in their study on Cu, Ni, Pd, and Pt the same approach as in their study on NaCl,^506^ based on the differential Lindemann–Gilvarry differential expression dln*T*_mel_/dln*V* = −2(γ_mel_ – 1/3) in combination with eqs 112-1 and 112-2, from which for *q* they calculated
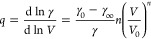
116-1and
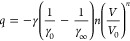
116-2while for λ they calculated
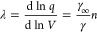
117-1and
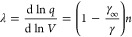
117-2

At *P* = *P*_0_, *q*_0_ = 1 and *V* = *V*_0_ and eq 116-1 yields *n* = γ_0_/(γ_0_ – γ_∞_). For this condition *q* = *n*(1 – γ_∞_γ^–1^). This expression predicts correctly that *q*_∞_ → 0 for γ → γ_∞_ when *P* → ∞. For the same conditions eq 117-1 results in *λγ* = λ_∞_γ_∞_ = *const*., so that λ increases with *P* since γ decreases with *P*. This is, however, in contradiction with thermodynamic constraints (section 7.1) and the authors conclude that eq 112-1 is not physically acceptable. Similarly, eq 116-2 predicts for *P* = *P*_0_ that *n* = γ_∞_/(γ_0_ – γ_∞_) and *q* = *n*(*γγ*_∞_^–1^ – 1), consistent with *q*_∞_ → 0 for γ → γ_∞_. Equation 117-2 results in λ/γ = λ_∞_/γ_∞_ = *const*., which is acceptable. From eqs 112-1 and 112-2, eqs 113-1 and 113-2 follow.^545^ Both eqs 113-1 and 113-2 were used to predict *T*_mel_. As for NaCl, these two equations yielded very similar results. While for Ni the calculation overestimates the melting curve, for Cu, Pd, and Pt it underestimates it as compared with the experimental data from Errandonea.^530,546^

Cu, Mn, Ni, Pd, and Pt were also studied by Hieu et al.^465^ by the statistical moment technique (see section 8.1) in combination with the Lindemann rule, the latter three metals also with defect-containing structures.^541,542^ Like for Kr, the results for the metals mentioned up to 100 GPa are in reasonable agreement with available experimental data.

**Figure 22:**
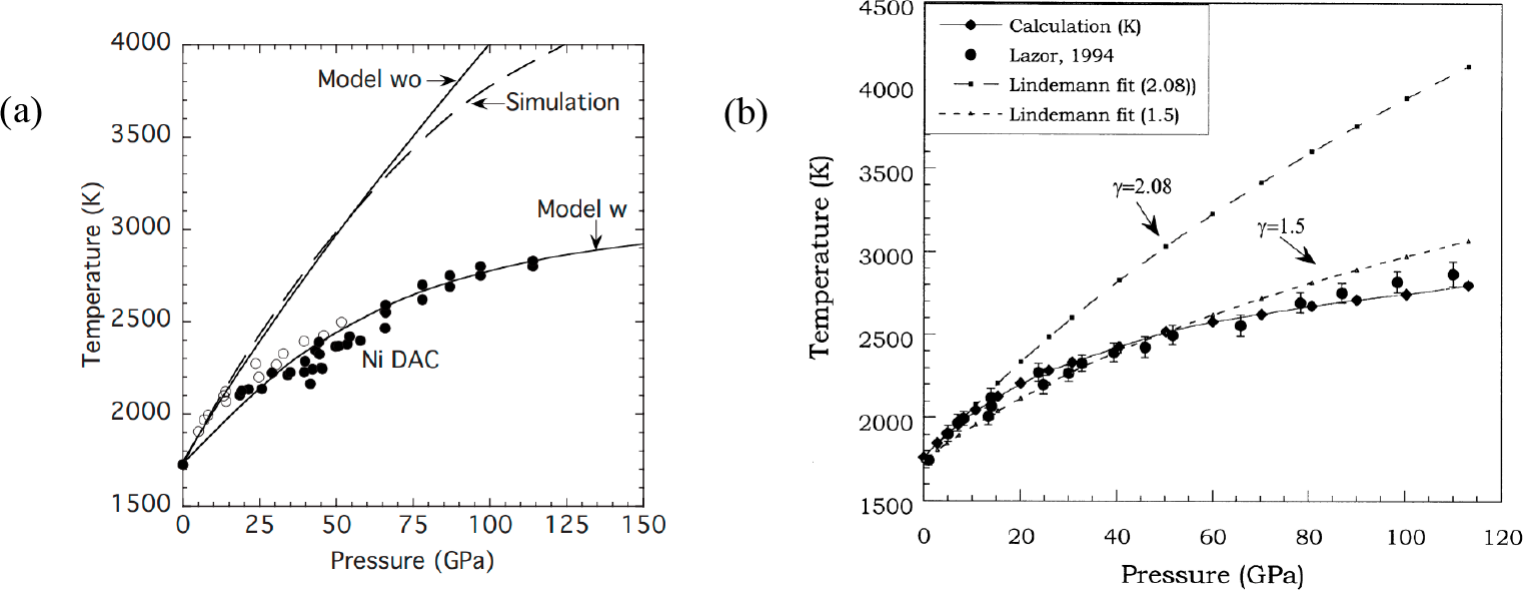
(a) The melting curve of Ni according to Ross et al.^529^ (dotted line) in comparison with simulation results (Model w) as well as their prediction without clusters (Model wo). Reproduced with permission from ref (529). Copyright 2007 American Physical Society. (b) The melting curve of Ni according to Wang et al.^524^ (eq 8), the Lindemann extrapolation (eq 4), and two experimental and two calculational results. Reproduced with permission from ref (524). Copyright 2001 Elsevier.

Ross et al.^529^ used for Ni, Cu, and Fe a model based on the inverse power law ϕ = *B*/*r*^*n*^. This allows the excess Helmholtz energy *F*^E^ and all the thermodynamic properties to be expressed as a function of the scaled inverse temperature *Γ* = *βBa*^*n*^, where β = 1/*RT*, *n*_0_ is the atom number density, and *a* is the Wigner–Seitz radius as calculated from 4π*a*^3^*n*_0_/3 = 1. The Helmholtz energy *F*^E^ contains the lattice energy *E* and the thermal contribution *F*_*X*_ for both the solid (*X* = S) and liquid (*X* = L), given for the inverse power law in ref (547). The value *n* = 9 was chosen for all three metals. According to the authors, the rather large difference in the Cu and Ni melting curves observed can be thought of as a consequence of “withdrawing” an electron from the filled Cu d shell, to “create” Ni, which now has a partially filled d shell with the capacity to form locally preferred structures in the liquid. They further remark that the presence of low melting slopes has been proposed to be due to the presence of local structures in the melt (refs (548 and 549) based on ref (550); see also ref (375)). An icosahedron, made up of four-atom tetrahedra, has a lower energy per atom and is denser packed than BCC or FCC and HCP structures for clusters of up to several hundred atoms. Although it is impossible to create a crystal with icosahedral symmetry, randomly packed clusters with icosahedral short-range order (CISRO), or polytetrahedra of varying sizes, may evolve continuously and be interconnected throughout the liquid. Since icosahedral structures in a liquid are well matched to the 5-fold symmetry of d-electron bonding and they are in effect impurities in the liquid, they are very likely to influence transition metal melting. While for Cu the effect of clusters is limited, for Ni it appeared to be considerable (Figure 22a). Their model including clusters described the experimental data well, while simulations did not. The reason for the failure of the EAM simulations to agree with the Ni diamond anvil cell (DAC) measurements is that the EAM potential does not include the strong directional bonding arising from an incomplete d-electron valence band and, thereby, lacks the capacity to form chemically preferred structures.

Wang et al.^524^ also studied Ni using their thermal pressure model as used for inorganics.^496^ Ni was chosen for the same reason as LiF, namely that it has a stable FCC lattice up to at least 100 GPa without interference of a phase transformation (ref (551), but easier, ref (552)). The calculated results agree excellently with the experimental data (Figure 22b). Estimates based on the Lindemann rule in combination with Slater’s expression for γ and the assumption γ_0_ρ_0_ = *γρ* are unable to describe the data, except below 10 GPa, even if the expression γ/γ_0_ = (*V*/*V*_0_)^*n*^ with *n* > 0 a parameter is used.

As a last example of an EoS approach, we refer to the model of Goyal and Gupta.^553,554^ Their EoS is given by
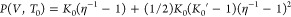
118where η = *V*/*V*_0_, *K*_0_ = −*V*(∂*P*/∂*V*)_*T*_, and *K*_0_′ = ∂(*K*_0_/∂*P*)_*T*_ and the subscript “0” indicates that the values are calculated at *P* = 0, while *T*_0_ denotes room temperature. This equation can be easily inverted^555^ and yields imaginary values for η above a certain temperature, which is identified as *T*_mel_. Further, *P*(*V*,*T*_0_) can be replaced by *P*(*V*,*T*) – *P*_the_, where the thermal pressure *P*_the_ is calculated from *P*_the_ = ∫_*T*_0__^*T*^*αK* d*T* with as usual α the thermal expansivity. Assuming constant *αK* values, *P*_the_ = *αK*(*T* – *T*_0_) so that for *P* = 0 the result is *P*_the_ = α_0_*K*_0_(*T* – *T*_0_). Combining this leads to *T*_mel,0_ – *T*_0_ = *K*_0_/2α_0_*K*_0_(*K*_0_′ – 1), but to obtain *T*_mel_ at pressure *P*, *K*_0_ and *K*_0_′ must be replaced by *K* and *K*′ and thus *T*_mel,*P*_ – *T*_0_ = *K*/2*αK*(*K*′ – 1). The relation between *T*_mel,*P*_ and *T*_mel,0_ consequently becomes
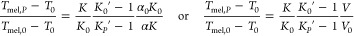
119after using the relation *αKV* = α_0_*K*_0_*V*_0_ which follows from the constancy of *αK*.^556^ The basic EoS uses only *K*_0_, *K*_0_′, and *T*_mel,0_ as input and results in *K*_∞_′ = 2. It thus satisfies Stacey’s criterion of *K*_∞_′ > 5/3. Data for Cu, Au, Ag, Zn, Cd, In, and Pb were calculated and compared with experimental data up to ∼12 GPa, mainly from Errandonea.^557^ The calculated values for Cu overestimate *T*_mel_ as compared with experimental data above ∼4 GPa^558,559^, those for Au, Ag, and Cd overestimate *T*_mel_ over the whole range, and those for Pb overestimate *T*_mel_ above ∼8 GPa. While the data for Zn agree with experiment over the whole range, for In *T*_mel_ is underestimated over the whole range. The agreement with results of other calculations is variable.

#### 8.3.2 Theoretical Models and Simulations

Sushko et al.^560^ attempted to reconcile the often-observed discrepancies between the results of MD calculations using EAM potentials and experimental data. They indicated that the long-range interaction notably influences the melting behavior, and a modification of the force field to weaken these interactions beyond the equilibrium distance was proposed. Using a modified potential, MD calculations were done for Ti, Mg, Au, and Pt for clusters of 300–80 000 atoms without periodic boundary conditions. The authors claim that their modified potential has a general nature that can be applied to other metals as well.

The melting curve as calculated with density functional theory using generalized gradient corrections was discussed by Alfè et al.^561^ for Al in the pressure range 0–150 GPa and for Fe in the pressure range 50–350 GPa. The melting curve agreed quite well with the shock wave data of Brown and McQueen^562^ and the point obtained from the measurements of Nguyen and Holmes.^563^ It also agreed with the low-pressure DAC experiments of Shen et al.,^564^ but a considerable discrepancy with the DAC data reported by Boehler^565^ existed.

Binary FCC and BCC alloys were treated using lattice dynamics in combination with the Lindemann rule by Hung et al.,^566^ content-wise reproduced in ref (567). The lattice dynamical expression for the Debye–Waller factor *W* for a binary alloy with *s* atoms of type 1 (mass *m*_1_) and *p* – *s* atoms of type 2 (mass *m*_2_) in the high-temperature approximation *T* ≫ θ_D_, given for the reciprocal lattice vector ***q*** by eq 120 was used.
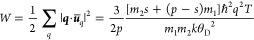
120

Combining the mean square displacement ∑_***q***_|***u̅***_*q*_|^2^ = *p*^–2^[*s* + (*p* – *s*)*m*]^2^|*u̅*_1*q*_|^2^, where *u*_2*q*_ = *mu*_1*q*_ and *m* = *m*_1_/*m*_2_, with the mean thermal energy ⟨*E*⟩ = ∑_*n*,*k*,*q*_*m*_*k*_*ω*_*k*_^2^|*U*_*nkq*_|^2^, where *ω*_*k*_ is the circular frequency and *U*_*nkq*_ is the amplitude of the modes *k*, *q* for cell *n* of the *N* cells in total, results for a nearest-neighbor distance *d* in the relative mean amplitude
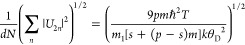
121Inverting, meanwhile using the abbreviation χ = *R*_m_^2^*kθ*_D_^2^*d*^2^/ℏ^2^, where *R*_m_^2^ = (*Nd*^2^)^−1^∑_*n*_|*U*_2*n*_|^2^, results in
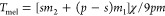
122

If *x* represents the mass fraction of atom type 1, *s* = *px*/[*sm*_1_ + (*p* – *s*)*m*_2_] and the average of the parameter *m* yields *m̅* = [*s*(*m*_2_/*m*_1_) + (*p*– *s*)(*m*_1_/*m*_2_)]/*p*. This equation can be solved by iteration using the expression for *s*, which leads to first order to
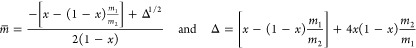
123and to be used in the expression for *T*_mel_. For χ the average χ = [*sχ*_1_^1/2^ + (*p* – *s*)χ_2_^1/2^]^2^/*p*^2^ was used with as boundary conditions χ_2_ = 9*T*_mel2_/*m*_2_ for *s* = 0 and χ_1_ = 9*T*_mel1_/*m*_1_ for *s* = *p*. From d*T*_mel_/d*x* = 0, the melting point was calculated for Cs_1–*x*_Rb_*x*_ and Cu_1–*x*_Au_*x*_ and found to be in reasonable agreement with experiment. Also, the eutectic composition was in good agreement with the experimental one. The results for the continuously increasing, respectively, decreasing melting points of the systems Cu_1–*x*_Ni_*x*_ and Cr_1–*x*_Cs_*x*_ are well described. The approach was extended to HCP crystals by Toan et al.,^568^ and results for Cd_1–*x*_Zn_*x*_, Zn_1–*x*_Tl_*x*_, Cd_1–*x*_Ti_*x*_, and Co_1–*x*_Zn_*x*_ showed equally good agreement with experiment. This is all probably not unexpected as the pure metal melting points were used as calibration points and the change of *T*_mel_ with composition is largely controlled by the mixing process, usually reasonably well described by a simple mixing rule.

Binary alloys have been dealt with in MD simulations as well. A good example is the paper by Akbarzadeh and Abbaspour^569^ where the effect of pressure *P*, size, and mole fraction on the melting of (Ir–Pt)_*N*_ clusters with *N* = 32, 108, and 256 was studied. While for the Ir mole fraction *x*_Ir_ = 0, 0.1, 0.3, 0.5, 0.7, 0.9, and 1 was taken, the pressure was varied between 0 and 90 kbar, for which the Lindemann ratio ξ, enthalpy change Δ_m_*H*, volume change Δ_m_*V*, radial distribution function (RDF), and self-diffusion coefficient *D* were assessed. Apart from the expected effect that *T*_mel_ increases with *P*, *T*_mel_ also increases with increasing *x*_Ir_ and size. The enthalpy change of fusion Δ_m_*H* decreases with increasing *P*, while Δ_m_*V* decreases with increasing *P* and Δ_m_*V* for the smaller cluster is larger than for the larger cluster. The volume change Δ_m_*V* decreases with increasing *x*_Ir_, which was attributed to the greater Ir–Ir interaction than the Rh–Rh interaction. Finally, with increasing *P* and increasing *x*_Ir_, the RDF peaks become more pronounced, both probably expected.

Rather complex single crystal and polycrystalline (Nb, Mo, Ta, W, V) high-entropy alloys have been discussed in the framework of MD simulations using a second NN modified EAM potential by Ju et al.^570^ For the single crystal, the density profile displayed an abrupt drop from 11.25 to 11.00 g cm^–3^ at *T* = 2910–2940 K, indicating all atoms show a significant local structural rearrangement. For the polycrystalline material, a two-stage melting process was found. In the first melting stage, melting of the grain boundary regions occurs at a premelting temperature lower than the corresponding system melting point. At the premelting temperature, most grain boundary atoms have sufficient kinetic energy to leave their equilibrium positions and then gradually induce a rearrangement of grain atoms close to the grain boundary. In the second melting stage at *T*_mel_, most grain atoms have enough kinetic energy to rearrange, resulting in chemical short-range order changes of all atom pairs.

An issue in general with melting simulations of clusters and nanoparticles is the problem of correctly identifying the equilibrium structure at the solid state. While in an infinite crystal it is usually known what the equilibrium lattice is, in a finite object this is not the case, since many different structures are in competition and starting from a nonequilibrium structure may cause artifacts. Moreover, it should be noted that evidence for vacancies in simulations of nanoparticles and clusters is meager (except for the very stable vacancies in the central part of icosahedra), as it is for bulk simulations, which is probably related to their relatively large activation energies. Bulk simulations with a free surface can, dependent on the surface, show in some cases no roughening or premelting, while in other cases surface roughening or premelting does occur. In both roughening and premelting vacancies are involved. Anyway, this brief discussion and the data in Table 5 clearly illustrate the myriad of methods that have been applied to deal with *T*_mel_ for metals.

### 8.4 Polymeric Solids

So far, we have largely discussed melting of small molecule or monomeric compounds. Polymers generally do not completely crystallize, and their melting behavior is more complicated than that of low molar mass compounds. However, for polymers single-chain single crystals do exist and such crystals melt by simple consecutive detachment of chain segments from the crystalline substrate and their diffusion into the melt. Complications in the melting process occur for a semicrystalline polymer where chains are shared between different crystals. The distribution of entanglements is highly heterogeneous as the entanglements are mostly confined to the amorphous regions, whereas the crystalline regions are devoid of them. This will influence the process of detachment from the surface. Experimentally, a clear distinction in different melting processes can be observed by considering the differences in the activation energies required for the consecutive detachment of chain segments or of segments having topological constraints. The consecutive detachment of free chain segments starts at the melting temperature predicted from the Gibbs–Thomson equation, whereas higher temperature or time is required if the chain has to overcome the constraints.^571^ Usually, the heterogeneous distribution of entanglements is lost on melting, and the entanglements are uniformly distributed along the chain, characterized by the molar mass between entanglements. With increasing molar mass *M*, the number of entanglements increases, and the melt viscosity follows the relationship η_0_ ∼ *M*^3.4^. However, for polymers with a low number of entanglements per unit volume the melting kinetics strongly influence the resulting melt state.^572^ On slow heating a long-lived heterogeneous melt state can be generated that shows a long-lasting low plateau modulus, high crystallization rate, and enhanced solid-state drawability. An early review on these aspects was given by Baur,^573^ while the influence of an amorphous component on the melting of semicrystalline polymers was discussed by Pandey et al.^574^

As usual, the equilibrium melting temperature is determined by the balance of enthalpy and entropy. For example, for polyethylene Δ_mel_*H* = 4.142 kJ mol^–1^ and Δ_mel_*S* = 9.9 J K^–1^ mol^–1^, resulting in *T*_mel_ = 145.5 °C. Similarly, for poly(1,4-*cis*-isoprene) Δ_mel_*H* = 4.393 kJ mol^–1^, Δ_mel_*S* = 14.2 J K^–1^ mol^–1^, and *T*_mel_ = 35.5 °C.^575^ Peculiar to polymers is that their melting and crystallization temperatures are not the same. The melting point *T*_mel_ observed is always larger than the crystallization temperature *T*_cry_ and a plot of *T*_mel_ versus *T*_cry_ is usually rather straight. However, generally a melting trajectory is present, and the melting temperature depends on the crystallization temperature used before and on the heating rate q*. Nevertheless, the concept of equilibrium temperature *T*_mel_^∞^ is introduced representing the melting temperature of an infinite crystal.

**Figure 23:**
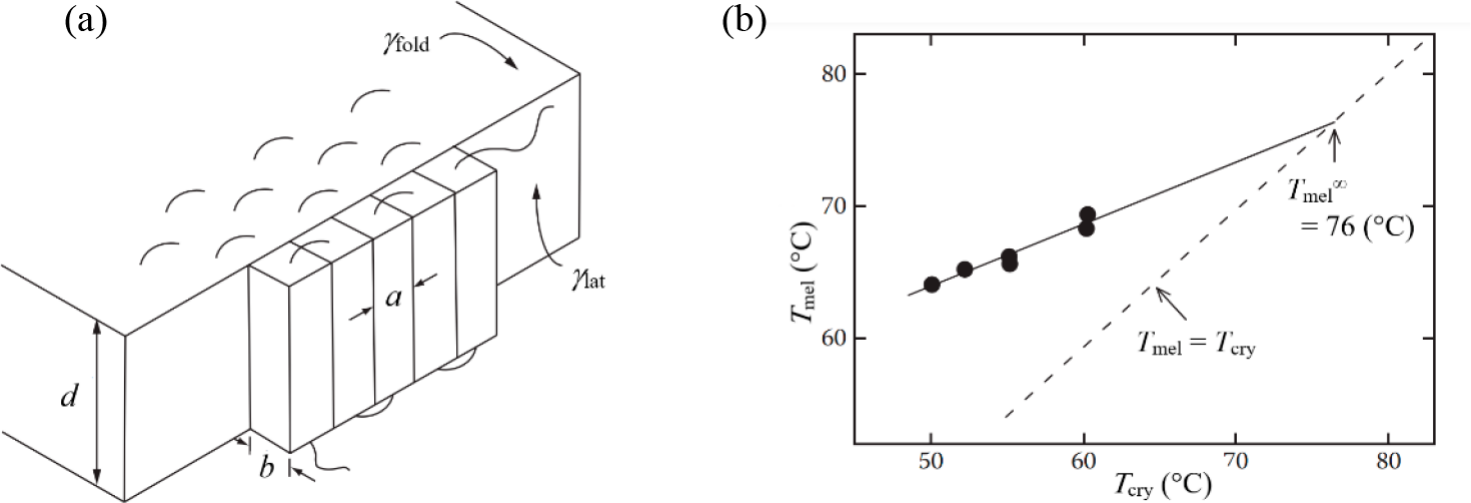
Polymer melting. (a) The Hoffman–Lauritzen model. (b) Melting temperature *T*_mel_ versus crystallization temperature *T*_cry_ for poly(dl-propylene oxide) with the equilibrium melting temperature *T*_mel_^∞^. Reproduced with permission from ref (577). Copyright 2011 Taylor & Francis.

Although devised for crystallization, the most often used model is that of Hoffman and Lauritzen,^576^ which builds on the nucleation model for small molecules. It covers several aspects of melting, and therefore we discuss it briefly. An embryo is supposed to have a cylinder-like shape, which grows to a platelet shape with thickness *d* and to which monomers with cross section *ab* add (Figure 23a). They are supposed to do that with an area *ad* parallel to the lateral surface of the platelet and fold sequentially over the lateral surface of the platelet having a surface energy γ_lat_. The surface energy of the planar surface of the platelet, the *fold plane*, is γ_fold_. The change in surface Gibbs energy is then given by Δ_sur_*G*_*n*_ = *2bdγ*_lat_ + 2*nabγ*_fold_, where *n* is the number of strands. The change in bulk Gibbs energy is Δ_cry_*G*_*n*_ = −*nabd*Δ_fus_*G*, where Δ_fus_*G* is estimated from Δ_fus_*G* = Δ_fus_*H* – *T*_mel_^∞^Δ_fus_*S*. If Δ_fus_*S* is not very temperature dependent, Δ_fus_*G* = Δ_fus_*H* – *T*Δ_fus_*H*/*T*_mel_^∞^, and introducing the undercooling Δ*T* = *T*_mel_^∞^ – *T*, the result is Δ_fus_*G* = Δ*T*Δ_fus_*H*/*T*_mel_^∞^. At given *n*, the expression for Δ*G*_*n*_ = Δ_cry_*G*_*n*_ + Δ_sur_*G*_*n*_ shows that it is maximal when *d* is small and decreases as *d* increases. The equilibrium value *d*° is obtained at Δ*G*_*n*_ = 0. Normally *n* is large, the term 2*bdγ*_lat_ in Δ_sur_G_*n*_ can be neglected, and combining Δ*G*_*n*_ with Δ_fus_*G* yields *d*° ≅ 2γ_fold_*T*_mel_^∞^/Δ*T*Δ_fus_*H*. This is, although a simplified model, indeed the experimentally observed proportionality.

The intercept between the melting and crystallization curves is taken as *T*_mel_^∞^ (Figure 23b). Upon forming a platelet with width *x* and length *y*, the change in surface energy is Δ_sur_*G* = 2(*x* + *y*)*dγ*_lat_ + 2*xyγ*_fold_, while the change in bulk energy is Δ_vol_*G* = 2*xyd*Δ_fus_*G* and thus the overall change Δ*G* = 2*xyd*Δ_fus_*G* – 2(*x* + *y*)*dγ*_lat_ – 2*xyγ*_fold_ ≅ 2*xyd*Δ_fus_*G* – 2*xyγ*_fold_. In equilibrium Δ*G* = 0, therefore *T*_mel_ = *T*_mel_^∞^ – 2γ_fold_*T*_mel_^∞^/*d*Δ_fus_*H* and the melting temperature of a finite size crystal is always less that for an infinite crystal *T*_mel_^∞^. If the relation between *T*_mel_ and *d* can be determined experimentally, a fit provides *T*_mel_^∞^ and γ_fold_ if Δ_fus_*H* is known from, for example, calorimetry. Upon annealing just below *T*_mel_ so that sufficient mobility is present, the polymers relax due a decrease in Δ_sur_*G*, resulting in an increase in thickness *d* and decrease in area *xy*. Upon melting, solidification, and remelting, *T*_mel_ thus increases. It also explains the dependence of *T*_mel_ on the heating rate q* as q* codetermines the amount of relaxation that can take place.

Various factors determine the *T*_mel_ of a polymer. Most important is the chemical structure. We mention a few factors. First is the stiffness of the chains. Groups such −O–, −O–O–, and −CO–O– increase the flexibility and lead to a lower *T*_mel_, while phenyl groups in the main chain decrease the flexibility and lead to a higher *T*_mel_. Second, the presence of polar groups such as amide groups −CONH– allows intermolecular hydrogen bonding, thereby increasing *T*_mel_. The third factor is the presence of side groups. For example, polypropylene, which can be considered as ethylene with regular CH_3_ side groups, has a reduced chain flexibility as compared to polyethylene and has a higher *T*_mel_. Bulky side groups will do the same, but long and flexible side groups generally reduce *T*_mel_. Also, the amount of branching is important as more side chains and chain ends reduce the packing density, thereby lowering *T*_mel_. The melting point thus can be manipulated significantly, but these changes also influence the glass transition temperature, which in its turn might influence crystallization.

Experimentally the melting behavior of polymers is often studied by thermal methods. The insights that can obtained by scanning calorimetry have been reviewed by Toda et al.^578^ and Furushima et al.,^579^ while the combination with time-resolved X-ray scattering has been discussed by Melnikov et al.^580^

The study of melting of the prototype polymer polyethylene has a long history. An early report by Weeks^581^ discussed the effect of time on the melting temperature and change of lamellar thickness for bulk polyethylene. By interpreting the melting points as characteristic of a given lamellar thickness, he concluded that the thickness of crystals of appreciable age increased linearly with the logarithm of their time of existence. In blends this thickening is slowed down as shown, e.g., by Barreiro et al.^582^ Another early report is by Fatou and Mandelkern^583^ dealing with molecular weights ranging from 3 × 10^3^ to 1.5 × 10^6^. Above 5 × 10^3^ the density decreases monotonically, while the XRD patterns broaden and a halo appears, which was interpreted in terms of the chain length relative to the crystal size and the amorphous regions occurring for larger molecular weight. The melting temperature showed the asymptotic value of 138.5 °C, which was explained, over the complete range, by assuming crystallite sizes in the chain direction comparable to those of the nuclei from which they are formed. In later reports Mandelkern et al.^584,585^ reported on both the experimental and theoretical difficulties encountered when determining the equilibrium melting temperature of a long chain molecule and indicated a higher asymptotic value of about 146 °C, as analyzed using the theory of Flory and Vrij.^586^ Derivatives of polyethylene have been studied in detail as well, e.g., isotactic polypropylene by Yamada et al.^587,588^ and syndiotactic polypropylene by De Rosa et al.^589^ For other polymers, even for relatively simple ones from a chemical point of view, the equilibrium temperatures are usually less clear due to dispersity and stereoirregularity. Such studies often result in approximate equilibrium temperatures, e.g., in the study by Okeda et al.^590^ for aliphatic polyesters such as poly(dodecamethylene dodecanedioate) and poly(tetradecamethylene tetradecanedioate). An overview of the thermodynamic factors that govern the melting behavior of crystalline homopolymers has been given by Mandelkern and Alamo.^591^ For polymers the size of the crystallites in relation to the (extended) chain length is important as well, and a study by Metatla et al.^592^ indicated that the surface energy of various nanocrystals was widely different for various experimental systems, thereby demonstrating the significance of the environment on thermal properties of nanocrystals.

In all these studies the dispersity and stereoregularity of the samples is crucial, and a somewhat extreme example showing this clearly can be found in a study by Miao et al.^593^ These authors studied the melting of high-density polyethylene crystals deposited on ultra-high-molecular-weight polyethylene fibers, which showed double melting peaks. By partial melting experiments they ascribed this to the bilayer components existing in the induced crystals, comprising an inner layer of more regularly folded chain crystals induced by the fibers and an outer a layer formed in the inner one with a lower ordered crystal structure.

An aspect for polymer melting that is not present for small molecular mass molecules is the possible presence of a mesomorphic phase, i.e., a state of matter intermediate between solid and liquid. In particular Strobl advocated this aspect for the understanding of crystallization, the detailed arguments being provided in his reviews.^594,595^ If true, this clearly implies varying melting temperatures, dependent on the conditions used. It seems that not many attempts to use the theory have been published, but for poly(ε-caprolactone) Sheth^596^ did so. The lamellar crystal thickness as a function of *T* was consistent with both the Hoffman–Lauritzen and Strobl models. However, in contrast to the predictions of Strobl’s model, the value of the mesomorph-to-crystal equilibrium transition temperature was very close to the zero-growth temperature. Moreover, the lateral block sizes (obtained from wide-angle X-ray diffraction) and the lamellar thicknesses were not found to be controlled by the mesomorph-to-crystal equilibrium transition temperature. Hence, Sheth concluded that the crystallization of poly(ε-caprolactone) is not mediated by a mesophase.

Notwithstanding the extra difficulties encountered when simulating long-chain molecules as compared to low molecular weight materials, many simulation papers on polymers appeared, often using a coarse-grained united atom model in some sense. An example is the paper by Iyer,^597^ who used Langevin dynamics simulations. They showed that melting of single crystals occurs via a globular metastable state, followed by an expansion to a more random coil-like state. Multichain crystals, however, showed a two-step mechanism where a long-living partially molten metastable state is formed followed by the second step where chains are peeled off form the crystalline core to a free state. Ramos et al.^598^ provided a review on predicting experimental results including the melting point for polyethylene by computer simulation.

## 9 Other Aspects

Some materials show deviations from the general behavior. Here we discuss two such deviations: first, a puzzle for almost 130 years on two different melts from the same solid and, second, the odd–even effect for chain molecules. Thereafter we discuss ultrafast experimental methods. In section 10, a few other approaches are indicated.

### 9.1 History-Dependent Melting

Although most small molecule compounds do show a clear-cut melting point, there are exceptions. Acetaldehyde phenylhydrazone (APH, C_8_H_10_N_2_), first prepared by Fischer in 1877, seemed to have two distinct forms with melting points 56 and 98 °C. The low melting solid can be converted to the high melting form by slurrying in weakly alkaline solution or by allowing ammonia vapor to permeate the solid for a few minutes. Conversely, the high melting solid can be converted to the low melting solid by slurrying in a weakly acid solution or by treatment with acidic vapors. Intrigued by the possible structural differences and reasons for the sensitivity to trace acid or base exposure, Bernades et al.^599^ investigated this phenomenon with diffraction, IR, DSC, NMR, calorimetry, microscopy, and simulations. All samples had identical IR and solid-state NMR spectra and identical crystal structures, but they exhibited sharp melting points varying from 56 to 101 °C. NMR studies of the melts provided the key to understanding this behavior: differently melting samples did so because they initially melted to liquids with different proportions of the *Z* and *E* isomers, although given enough time they all tended to the same equilibrium proportion. It might be useful to recall that *E* isomers have the substituents preferably on the opposite sides of a double bond, while *Z* isomers have them preferably on the same side. The letter *E* stems from “entgegen” (German, meaning “opposite”) and the letter *Z* stems from “zusammen” (German, meaning “together”). For relatively simple compounds *E* means a trans isomer and *Z* means a cis isomer, but for more complex compounds the *E*–*Z* designation is more refined. Anyway, the thermodynamic equilibrium state for the melt of APH is one with an isomer ratio *E*/*Z* = 1.7, while that for the solid state corresponds to a pure *Z* compound. For instantaneous isomerization in the melt, the melting temperature should be found at the thermodynamic melting temperature, but for a relatively slow isomerization, it will result in a long(er) lifetime of the metastable melt. This isomerization process is catalyzed by traces of acid or base as usual. Therefore, in polymorphism different structures melt to the same liquid, but in this case, the same structure melts to different metastable liquids.

### 9.2 The Odd–Even Effect

**Figure 24:**
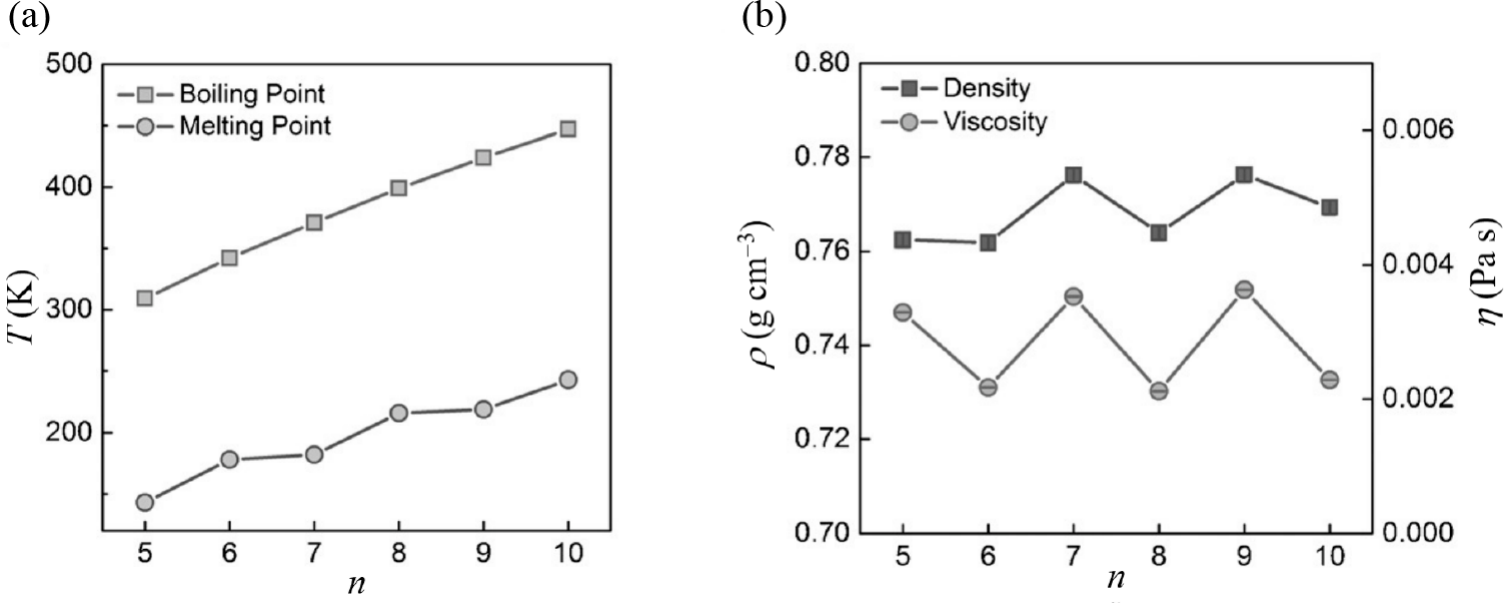
Odd–even effect. (a) Melting point and boiling of the first few alkanes. (b) Density and viscosity of the same first few alkanes. Reproduced with permission from ref (604). Copyright 2016 John Wiley & Sons.

Several molecules do show the odd–even effect, *n*-alkanes (C_*n*_H_2*n*+2_) being a well-known example.^600^ The melting points of *n*-alkanes show a zigzag pattern rather than a monotonic trend as a function of the number of carbon atoms (Figure 24a). This odd–even effect also holds for most of the α- and α,ω-substituents,^601−603^ with the latter showing a larger effect.^601^ The effect becomes more significant for short chains: the shorter the chain length, the larger the difference in melting points between two *n*-alkanes differing in length by one carbon atom. Other physical properties, such as sublimation enthalpy, solubility, and modulus, display a similar odd–even effect.^601−603^ Although the odd–even effect was discovered about 150 years ago,^600^ its molecular origin was revealed only relatively recently by Boese et al.^603^ by using controlled crystal growth and single-crystal diffraction to solve the crystal structures of several *n*-alkanes.

Kitaigorodskii^605^ considered the packing of such molecules purely geometrically but indicated that the interaction potential would play a role. From the study by Boese et al. it appeared that the intermolecular distances between the CH_3_ end groups are responsible for the alternation in the densities and melting points. The even-numbered *n*-alkanes have optimal intermolecular contacts at both ends, whereas the odd ones possess this optimum only at one end, while at the other end their distances are larger. This leads to a less dense packing for the odd *n*-alkanes, and their densities are lower with relatively lower melting points as a result. The effect diminishes with increasing chain length because the van der Waals attraction becomes dominating.

Broadhurst^606^ also studied long alkanes (14 paraffins, *n* = 44–100) and showed that the melting point can be described well by *T*_mel_ = *T*_mel_°[(*n* + *a*)/(*n* + *b*)], where *T*_mel_° = 141.1 °C, *a* = −1.5, and *b* = 5.0. Associated thermodynamical data as well as some refinements are available as well.^607^ Structure studies on *n*-alkyl carboxylic acids (*n* = 6–16)^608^ showed that molecules form hydrogen-bonded dimers arranged into bilayers with a rectangular packing arrangement in the plane perpendicular to the dimer long axes. In all structures the carboxyl groups are identically disposed and the packing density within bilayers is comparable so that the alternating crystal density can be attributed solely to alternating packing density between bilayers. The odd–even effect has also been observed for adsorbed layers.^609,610^

In contrast, the boiling points of *n*-alkanes increase monotonically as a function of the molecular weight (Figure 24a), thus suggesting that the odd–even effect does not exist in the liquid state. Yang et al.^604^ showed, possibly somewhat surprisingly, that the odd–even effect also exists in the liquid state for the translational diffusivity. They used quasi-elastic neutron scattering experiments to measure the relaxation dynamics of the liquid *n*-alkanes. It appeared that odd-numbered *n*-alkanes exhibit up to 30 times slower dynamics than even-numbered *n*-alkanes near their respective melting points. Hence, an odd–even effect does exist in the liquid state (Figure 24b). The effect is more prominent in dynamic quantities than in thermodynamic quantities, and the authors suggest that mechanisms other than periodic packing should be scrutinized to gain a more thorough understanding. Further, they speculate that because the symmetries of odd and even *n*-alkane molecules in an all-trans configuration are different, although this is not the favorable configuration in the liquid state, an extra CH_3_ group switches the structural symmetry of neighboring *n*-alkanes and thus affects the local packing structures of the *n*-alkane molecules. The effect should thus diminish with increasing temperature.

### 9.3 Ultrafast Experimental Methods

Although this paper is focusing on models, we cannot avoid discussing at least briefly some experimental results using nonconventional methods. Usually, experimental studies of melting comprise calorimetry and/or microscopy and physical property measurements in some form. Such methods use normal heating rates. Developments since about 2003 in ultrafast heating using laser pulses combined with ultrafast electron diffraction have led to some further insights in the melting process, with experiments mostly done on thin films. For conditions far from equilibrium (hot electrons and still cold lattice), they offer a unique opportunity to get a better understanding of the electron–ion interplay. For covalent materials, electronically driven phase transitions were reported, also referred to as nonthermal melting. This mechanism occurs when the laser-induced electronic excitation rearranges the positions of the ions in a liquid-like disordered configuration before the lattice reaches the melting temperature, i.e., to changes in the potential-energy landscape of the lattice by the excited electrons.^611^ In metals, a thermal process, which results from a rise of the lattice temperature above the material’s melting point, is generally considered to be driven by the progressive energy transfer from the electrons to the lattice.^612^ Typically, films with a thickness smaller than the range of the ballistic energy transport by the excited electrons (e.g., ∼100 nm for Au) are used, so a uniform electron temperature distribution throughout the film thickness is established before any substantial lattice heating, rendering the interpretation of the experimental observations more straightforward.

**Figure 25:**
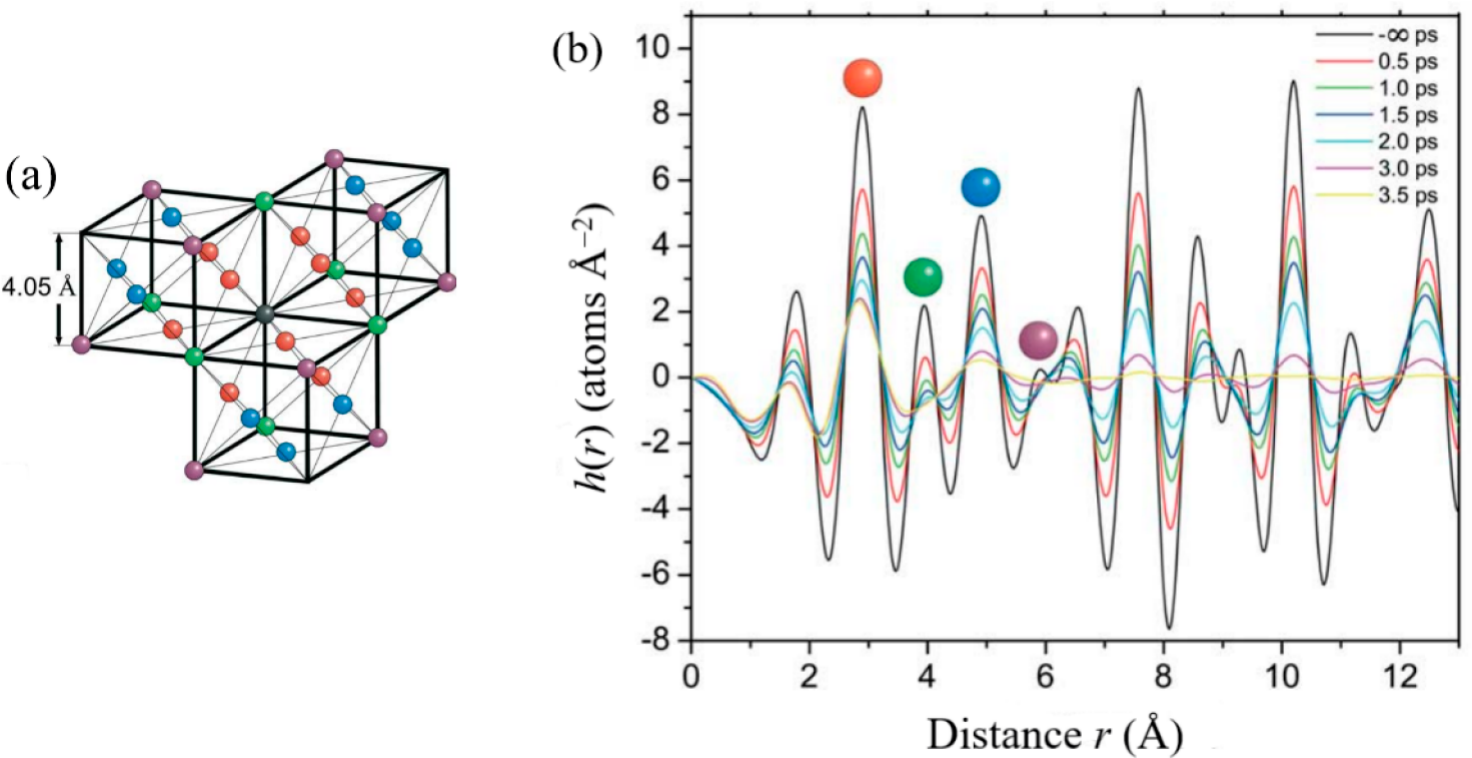
(a) The FCC structure of Al where atoms in the structure have been color coded such that each color represents a given distance from the central black atom. (b) The time-dependent pair correlation function *h*(*r*) where the correspondence between the peaks in *h*(*r*) and the interatomic spacings present in the FCC Al lattice are shown for the first four peaks by labeling with the same color as in (a). The peak at *r* = 1.8 Å is due to data termination at *s*_max_ ≅ 1.4 Å^–1^. Reproduced with permission from ref (614). Copyright 2004 Elsevier.

Possibly the first detailed investigation using these methods is by Siwick et al.,^613^ providing full experimental details in ref (614). These authors used 600 fs laser pulses to study the structural evolution for 20 ± 2 nm thick Al samples as they were subjected to an excitation fluence of 70 mJ cm^–2^ by 120 fs near-infrared (775 nm) laser pulses. As configured for these experiments, the detector collected scattering vectors *s* up to a magnitude of *s* = 2 sin(θ)/λ = 1.35 Å^–1^, encompassing the first 10 rings of the powder diffraction pattern of Al. The loss of long-range order that was present in the FCC crystalline phase and the emergence of the liquid structure where only short-range atomic correlations are present with only a single broad diffraction ring were complete within 3.5 ps. This time scale is primarily determined by the magnitude of the electron–phonon coupling constant in Al and makes electronically driven disordering unlikely. Considering the fast electron redistribution times in metals and the thermalization rate of the hot electron energy redistribution into lattice phonons, the Al lattice is expected to achieve the melting point temperature *T*_mel_ = 933 K within 750 fs after excitation under the strongly driven conditions of the experiment. This corresponds to a heating rate of more than 800 K ps^–1^. By 1.5 ps, the projected lattice temperature *T*_1_ is 1400 K (*T*_l_/*T*_mel_ ≅ 1.5), which suggests significant superheating during the phase transition. The direct correlation function *h*(*r*) = *g*(*r*) – 1, describing the deviation in atomic density from the average value as a function of the radial distance *r* from an atomic origin, was calculated at various time delays after the excitation pulse. The positions of the first three coordination shells after 6 ps [*r*_1_ = 2.85 ± 0.05 Å, *r*_2_ = 4.9 ± 0.1 Å, and *r*_3_ = 7.6 ± 0.1 Å] are in approximate agreement with values obtained from X-ray diffraction studies of liquid Al (*r*_1_ = 2.9 ± 0.1 Å, *r*_2_ = 5.2 ± 0.1 Å, and *r*_3_ = 7.6 ± 0.1 Å).^615^ All peaks shift slightly to larger distance between 6 and 50 ps, suggesting that the liquid structure has not fully equilibrated within 6 ps. The first coordination number *N*_1_ just before the phase transition (−1 ps) appeared to be *N*_1_ = 12.2 ± 0.3, within error equal to the expected number for an FCC lattice. The loss of lattice structure and the subsequent atomic rearrangements reduce *N*_1_ to 10.0 ± 0.3 at 6 ps with no observable change on longer time scales. The time development of *h*(*r*) is shown in Figure 25. Overall, the authors conclude that the transformation is a thermal process, contrary to an earlier conclusion that the transformation is a nonthermal process, based on permittivity measurements at 800 nm reaching the value for liquid Al after 500 fs.^616^

The former conclusion was confirmed by Kandyla et al.,^617,618^ who measured the reflectivity of Al during the laser-induced solid-to-liquid phase transition over the wavelength range from 350 to 730 nm with a time resolution of 65 fs. These authors showed that, at all excitation intensities over the entire range of wavelengths used, it took 1.5–2 ps for the transition to be complete, so the phase transition in optically excited Al is thermal and mediated by heat transferred from the excited electronic population to the lattice through electron–phonon interactions.

A more recent study by Meng et al.^619^ on Al films with a thickness of 133.4 nm irradiated by the femtosecond laser with 200 fs duration and 150 J m^–2^ absorbed fluence showed that the melting process goes through two stages: first, a rapid melting stage dominated by homogeneous melting within the first 2 ps, followed by a slow melting stage dominated by heterogeneous melting within 20 ps. The molten aluminum gradually develops an interface between the liquid zone and the melting zone, after 20 ps a clear interface between the liquid zone and the melting zone. Simulations with an optimized embedded atom method (EAM) potential showed that the size of the two melting zones can be controlled by the electron–phonon coupling factor and electron thermal conductivity.

The authors suggest that for the fast processes involved it seems feasible to control the melting phenomenon by adjusting the laser parameters and then changing the material properties, which might help to improve the quality of additive manufacturing, controllable surface modification processing, and the control of the phase change process and morphology under laser processing.

Apart from ultrafast electron diffraction, also other methods have been used. Jourdain et al.^620^ used time-resolved XANES experiments to study the phase transition dynamics in femtosecond laser heated Cu with samples of 80 ± 5 nm thickness deposited on 0.9 μm of Mylar. Exploiting features associated with van Hove singularities in the electron structure, the loss of the lattice periodicity was observed in the picosecond or even subpicosecond time scale, in the range of specific energy density from 1 to 5 MJ kg^–1^. The overall observations were fairly well reproduced by a two-temperature model—a model that employs a lattice temperature *T*_lat_ and an electronic temperature *T*_ele_(621)—provided that *T*_ele_-dependent coefficients are considered^622^ and assuming that the melting occurs when the ion temperature exceeds the melting temperature.

Modeling on superheating has been done as well. Hwang and Levitas^623^ used a multiphysics model that includes the phase field model for surface melting, a dynamic equation of motion, a mechanical model for stress and strain simulations, interface and surface stresses, and a thermal conduction model including thermoelastic and thermophase transformation coupling as well as a transformation dissipation rate. As the external surface of metallic particles is usually covered by a thin and strong oxide shell, which significantly affects superheating and melting of particles, the effects of geometrical parameters and heating rate on the characteristic melting and superheating temperatures and melting behavior of Al nanoparticles covered by an oxide shell were studied. The results showed that, for heating rates of <10^9^ K s^–1^, the melting temperatures (surface and bulk start and finish melting temperatures, and maximum superheating temperature) are independent of the heating rate. For a heating rate of >10^12^ K s^–1^, in comparison with a bare particle, the pressure generated in a core due to the different thermal expansivities of the core and shell and volumetric expansion during melting increases *T*_mel_ with 60 K GPa^–1^, leading to an increase of these melting temperatures and the temperature for shell fracture. For such rates, heterogeneity in the temperature distribution results as well as wave propagation within the core, which causes oscillation in pressure and temperature due to thermoelastic coupling, although the effect on the melting temperatures and the maximum attainable temperature of Al at the interface attained before the fracture of the oxide shell appears to be relatively small for the heating rates used.

Experiments on Au films with a relatively weak electron–phonon coupling were done by Arefev et al.^624^ This leads to an increased time scale of lattice heating and a separation of nonthermal effects defined by the electronic excitation from thermally driven atomic dynamics and phase transformations. The authors also discussed the effect of the amount of superheating, as this affects the melting time significantly. The threshold energy density for complete melting ε_mel_ can be evaluated by integration of the temperature-dependent heat capacity *c*_*P*_(*T*) from 300 K to *T*_mel_ and addition of the enthalpy of melting Δ_m_*H*. Consequently, the superheating energy density ε is often expressed in terms of ε_mel_. The following discussion is largely taken from the corresponding one by Arefev et al.^624^

Measurements performed for 20 nm Au films irradiated by 200 fs laser pulses ε = 1.5ε_mel_ and ε = 1.7ε_mel_ revealed a melting process that starts at about 7 ps and takes approximately 3 ps to complete.^625^ These results are consistent with a melting time of about 7 ps reported for 35 nm Au film irradiated by a 90 fs laser pulse at ε = 1.8ε_mel_.^626^ The melting time further shortens as the deposited energy density increases by more than an order of magnitude above ε_mel_,^627^ where the interpretation of the results involves a consideration of transient bond hardening in Au under conditions of strong electronic excitation as predicted by ab initio calculations.^628^ The decrease of the energy density down to the values approaching ε_mel_, on the other hand, leads to a gradual increase in the melting time, e.g., up to ∼15 ps for a 10 nm Au film irradiated by a 90 fs laser pulse at ε = 1.1ε_mel_.^626,629^ Such observations are consistent with the physical picture of homogeneous melting proceeding through massive nucleation and growth of liquid regions in a crystal superheated up to the limit of thermodynamic stability of the crystal lattice (e.g., refs (39 and 340)). Classical nucleation theory suggests that the phase transformation should occur within ∼10 ps when the temperature reaches the level of T* = 1.25*T*_mel_, although the above sketched picture is unlikely to remain valid at temperatures approaching T*. Another mechanism of melting is the heterogeneous nucleation of liquid at free surfaces of the irradiated film followed by the propagation of the melting fronts toward the center of the film. However, a quantitative analysis of the kinetics of melting and the results of two-temperature MD simulations^626,629^ suggest that, in the case of ultrashort pulse laser interaction with thin Au films, the melting front propagation becomes dominant only below the threshold for complete melting, which was estimated as 1.29*T*_mel_ and thus (a little bit) higher than T*. Also, the distance the melting fronts can propagate during the short time the electron–phonon coupling heats the film from *T*_mel_ to T* is far below the thickness of the films used in the experiments. The two-temperature MD simulations performed for 20 nm Au films suggest that the homogeneous nucleation of liquid regions and heterogeneous propagation of melting fronts from the free surfaces make a comparable contribution just above the threshold for complete melting, at the deposited energy density of 1.02ε_mel_.^630^ The melting proceeding through the propagation of melting fronts alone, without the contribution of the homogeneous nucleation, is only observed in simulations of partial (incomplete) melting, at 0.84ε_mel_ for 20 nm Au films and at 0.97ε_mel_ for a 10 nm film.

Experiments performed for single-crystal 35 nm Au films irradiated by 130 fs laser pulses,^612^ however, are in sharp contrast to the above computational predictions. These experiments suggest a large increase in the melting time as the deposited energy density decreases below 1.9ε_mel_, which is interpreted as an indication of the transition to the regime of heterogeneous melting. At an energy density of 1.7ε_mel_, the presence of the diffraction peaks corresponding to the crystalline Au is reported to persist up to 800 ps, and the melting time exceeding 2 ns is reported for 1.5ε_mel_. The apparent disagreement of the above results with the results of the two-temperature MD simulations, where the melting time remains below 100 ps as the deposited energy density decreases down to ε_mel_, has been attributed to the inaccuracies of interatomic potentials and overestimation of the strength of the electron–phonon coupling.^612,631^

The aim of Arefev et al.^624^ was to check the hypothesis that the discrepancy between the time of complete melting observed in the experiments and that predicted in earlier simulations can be eliminated by using an improved interatomic potential and assuming a lower strength of the electron–phonon coupling. However, their calculations indicate that the long melting times in the vicinity of the melting threshold and the contribution of the heterogeneous melting inferred from the experiments cannot be reconciled with the atomistic simulations by any reasonable variation of the electron–phonon coupling parameter. Thus, the authors suggest further coordinated experimental and theoretical efforts aimed at addressing the mechanisms and kinetics of laser-induced melting.

The field is active at present, and many other papers appeared, e.g., the experimental ones by Gelisio et al.^632^ on 100 nm thickness Pt films and by Shin et al.^633^ on 100 nm Au nanospheres covered by a 30 nm thick SiO_2_ shell. Also, many modeling papers appeared, e.g., by Xiang et al.^634^ on modeling for polycrystalline effects, by Wang et al.^635^ on hard sphere crystals, and by Zier et al.^636^ on ab initio MD simulations of Si. Forsblom and Grimvall,^637^ using atomistic simulations relevant for Al, focused on atomistic details to show that the thermal fluctuation initiating melting is an aggregate typically with six to seven interstitials and three to four vacancies, a mechanism differing from those that have traditionally been proposed. An extensive review on ultrafast electron diffraction methods has been presented by Filippetto et al.^638^

Overall, from these studies it has become clear that for ultrafast heating experiments bulk or mechanical melting prevails if the energy density ε supplied is larger than the threshold energy density for complete melting ε_mel_. Otherwise, surface-mediated or thermodynamic melting occurs. Clearly, such fast homogeneous melting is only possible for relatively thin films and the critical thickness for this is determined by the range of the ballistic energy transport by the excited electrons, ∼100 nm for Au.^639^ Still, the effect of surface of interface atoms may be substantial. For example, metals typically have an exponential decaying “interphase” thickness exp(−*z*/ξ) with a characteristic distance ξ of ∼1.5 monolayers (∼0.6 nm, section 6.1). If the “interphase” effect is less than 10%, we require exp(−2.5), corresponding to 1.5 nm. For a 20 nm thick film of Al (metallic radius 0.143 nm), we thus have 3/20 of all atoms within the “interphase”.

## 10 Other Approaches

Apart from the various mechanisms discussed, there are a few other ways of dealing with melting, which we discuss here. We start with other one-phase approaches and deal thereafter with two-phase approaches.

### 10.1 Other One-Phase Approaches

In this category we discuss models based on lattice dynamics, energy balance, and scaling, followed by various other models.

#### 10.1.1 Lattice Dynamics Models

Many discussions within the framework of one-phase approaches on the contribution of thermal motion to the energetics relevant for melting employ the Debye model in some way or another. Lattice dynamics is in principle a better alternative with as the most frequently form used the self-consistent phonon theory (SCPT^640,641^) with the vibrational frequencies still determined by harmonic force constants which are, however, taken as *T*- and *V*-dependent. The reason to believe that SCPT can be applied near *T*_mel_ is that the displacements *u* near *T*_mel_ are still generally small, although this is disputed by some.^241^ However, the resulting equations cannot be solved explicitly, and therefore still often the Debye approximation ω = *c*_0_*q* leading to a frequency spectrum ∼*q*^2^ is made. Doing so and assuming that all sound velocities are given by *c* (and making a few other approximations), Fukuyama and Platzman^642^ showed that for cubic crystals this leads to *T* = *mc*_0_^2^*t* exp(−12*t*) with *t* = *T*/12*mc*^2^. The right-hand side shows a maximum, and because *c*^2^ > 0, this expression will have no solution if *T* > *T*_mel_ = *mc*_0_^2^/12e = 0.031*mc*_0_^2^. This describes the overall physics of the situation well: with increasing temperature, *u* increases, and the potential softens in an exponential way so that *c* decreases, which on its turn influences the potential, until a temperature is reached where the potential runs away and transverse waves cannot longer exist.

A much more elaborate analysis of SCPT was given in a very clear presentation by Rastelli and Cappelluti^643^ spanning the whole phase diagram versus *V* and *P*. They used the Einstein approximation for the frequency spectrum and a double Gaussian potential, one term representing repulsion and the other representing attraction, to keep the analysis as far as possible analytical. The Helmholtz energy *F*(*V*,*T*) was calculated from the resulting partition function, as was the pressure *P* = −(∂*F*/∂*V*)_*T*_ so that the Gibbs energy *G*(*P*,*T*) = *F*[*T*,*V*(*T*,*P*)] + *PV*(*T*,*P*) could be assessed. Two different kinds of mechanisms were identified: one mainly relevant at constant *V*, associated with the vanishing of the SCPT solution, and one related to the disappearing at the spinodal temperature of the solid phase as a metastable energy minimum. The authors showed how the first mechanism occurs at extremely high temperatures and it is not reflected in any singular behavior of the thermodynamic properties. In contrast, the second one appears at physical temperatures which correlate well with the melting temperature, and it is signalized by the divergence of the thermal compressibility and of the lattice expansion coefficient. The authors suggested that inclusion of higher order anharmonic terms and the development of models beyond the Einstein approximation might further reduce *T*_S_ toward the empirical range for overheating *T*_max_ ∼ 1.5*T*_mel_.^340^

#### 10.1.2 Energy Balance Models

Another idea, namely that melting is related to an energy balance without invoking two phases as in thermodynamics, is essentially already contained in the Lindemann approach. Vaidya^119,644−646^ postulated an energy balance principle, which claims that a crystal is stable when a certain fraction *f* of the stability interaction energy per atom *E*_pot_(*T*) is less than the vibrational energy *E*_vib_(*T*), i.e., *E*_vib_(*T*) < *fE*_pot_(*T*). Here *E*_pot_(*T*) = (1/2)∑_*l*≠0_⟨ϕ(***r***_0_ – ***r***_*l*_)⟩ ≡ (1/2)∑_*l*≠0_⟨*ϕ*_*l*_⟩ with ⟨ϕ(***r***)⟩ a thermally averaged or effective pair potential over lattice sites *l*. Therefore, although called energy, *E*_pot_(*T*) is essentially Helmholtz energy. The fraction *f* is of the order of, but less than, 1, as the transition is from the solid to the liquid state. Clearly, as expected and explicitly shown by lattice calculations in the self-consistent harmonic approximation (SCHA),^640,641^ ⟨ϕ(***r***)⟩ decreases with increasing temperature. Calculations were done for cubic monatomic lattices using for the vibrational energy the high-temperature approximation *E*_vib_(*T*) = 3*kT* to the Debye expression. The critical temperature for the energy balance is thus *T*_EB_ = *fE*_pot_(*T*)/6*k*. This led to
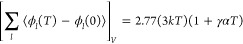
124with γ and α the Grüneisen parameter and thermal volumetric expansivity. By taking the representative values *αT*_mel_ ≅ 0.66 × 10^–2^ and γ ≅ 1.6, the value 1/[2.77(1 + *γαT*)] = 0.326 was obtained, while calculatiion of ∑_*l*≠0_⟨*ϕ*_*l*_⟩ was done numerically by taking the sum over all reciprocal lattice space, which led for the rare gas solids to ∑_*l*≠0_⟨*ϕ*_*l*_(*T*)⟩/∑_*l*≠0_⟨*ϕ*_*l*_(0)⟩ ≅ 0.6. Overall, this means that 3*kT*_mel_ = *f*·(1/2)∑_*l*≠0_⟨ϕ(*T*_mel_)_*l*_⟩ with *f* ≅ 0.43. Estimates using the LJ potentials, limited to second-nearest neighbors only, yielded *f*_Ne_ = 0.337, *f*_Ar_ = 0.429, *f*_Kr_ = 0.430, and *f*_Xe_ = 0.321.

A comparable approach was presented by Doi and Kamigaito^647^ for simple inorganic compounds. The authors calculated the potential energy *Φ* by adding the ionic (electrostatic) interaction, proportional to *C*_ion_*r*^–1^, and covalent interaction, proportional to *C*_cov_*r*^–*p*^, thereby neglecting the repulsive interaction as that interaction is much smaller than the other two. The exponent *p* was first considered as a parameter that was fitted on the melting points of Si, Ge, and C (taken at 1 GPa as diamond sublimes at normal pressure). The resulting value *p* = 2.3 is consistent with the Phillips value *p* = 2.5,^648^ obtained from grouping 80 A_*n*_B_8–*n*_ compounds rather accurately into 4-fold and 6-fold structure type compounds by considering ionic and covalent contributions to their average band gap energy. The latter value, considered as being more “desirable”, was used further on. Thereafter the melting points were calculated assuming that *kT*_mel_ = Δ*Φ*, where Δ*Φ* is the change in potential energy from *T* = 0 to *T* = *T*_mel_. Because the ionic positions at *T*_mel_ are generally unknown, the values of *C*_ion_ and *C*_cov_ were determined for a series of similar compounds by fitting to the experimental *T*_mel_ values. The fractions of the ionic and covalent contributions were calculated with Pauling’s method^649^ as well as Sanderson’s method^650^ for dealing with electronegativity. While both the molecular and crystal parametrizations of Pauling yielded (partially) negative and thus physically impossible *C*_cov_ values for the alkali halides (minus the Li halides), Sanderson’s parametrization resulted in reasonable values and were further used. This resulted in an average absolute value |Δ*T*_mel_| = 20 °C for the alkali halides, if grouped according to their anions, i.e., labeling them as fluorides, etc. When grouped according to their cations, the calculations resulted in the comparable value |Δ*T*_mel_| = 17 °C, but with larger *C*_cov_ values by a factor of about 5.7 and smaller *C*_ion_ values by a factor of about 2.5, thereby suggesting that the nature of the anions is important in the melting. Values for MO-type and M_2_O_3_-type oxides were calculated as well but resulted generally in much larger |Δ*T*_mel_| values, the reasons advanced being the presence of different structures and nonstoichiometry.

Much later, Ma et al.^651^ presented a model based on what they call the “force-heat equivalence energy density principle”, for which they refer to a paper by Li et al.^652^ The latter authors assumed, because breaking bonds between atoms of a material involves either applying work or heat transfer, a kind of equivalence between heat energy and strain energy to break bonds with a constant maximum storage of energy that includes both the strain and the corresponding equivalent heat energy. It essentially states that the maximally stored energy (i.e., the internal energy) *E* equals the sum of the strain energy (i.e., the mechanical work supplied) *E*_*σ*_ and the “heat energy” (i.e., the heat supplied) *E*_*T*_. Based upon this, a temperature-dependent fracture strength model was developed for ultra-high-temperature ceramics. This principle was claimed to be used by Ma et al., but strangely they wrote *E*^*n*^ = *αE*_KE_ + *βE*_PE_ with α and β called “equivalent” coefficients and *n* called an “equivalent” index. Further, equipartition was assumed resulting in *W*_KE_ = *W*_PE_ = 3/*N*_A_*kT*/2*M* with *N*_A_ Avogadro’s constant and *M* the molar mass. Identifying *W* with −∫*P* d*V* and inserting the Murnaghan equation *P* = (*K*_0_/*K*_0_′)[(*v*_0_/*v*) – 1], with *K*_0_, *K*_0_′, and *v*_0_ denoting the bulk modulus, its pressure derivative, and the specific volume at *P* = 0, the sum α + β can be determined. Finally inserting that result and the Murnaghan equation in *E*^*n*^ = *αE*_KE_ + *βE*_PE_ results in an explicit expression for *T*_mel_, which upon fitting data for 10 metals as determined by various other authors yielded *n* = 1/2. Using this fitted *n*-value, one requires only *K*_0_ and *K*_0_′ to estimate the pressure dependence of *T*_mel_. For another 12 metals the calculated results agree well with the experimental results. The statement by the authors that the model does not include any adjustable parameter is, however, misleading as introducing the *n*-value was ad hoc, while its value was determined empirically.

After having briefly discussed the results of Ma et al.,^651^ we note that the “force-heat equivalence energy density principle” did not originate from Li et al.^652^ A rather similar approach in connection with fracture was already used earlier by Ivanova and Ragozin in 1965^653,654^ as well as by Cherepanov in 1979,^655^ the latter denoting it as the “method of thermal transformation”, and applied since by others; see, e.g., ref (656). Moreover, Vaidya^644^ postulated the energy balance principle in 1984.

Magomedov^657^ also used an energy based approach and proposed a localization criterion for the S–L phase transformation. Defining *E*_del_ as the energy of atom delocalization, the transformation begins, according to this criterion, when the *E*_del_/*kT* ratio reaches a boundary value *E*_del_(*T*_mel_)/*kT*_mel_ such that a solid phase is present above it and a liquid phase is present below it in a phase diagram. The author showed that his criterion contains both the Lindemann criterion for melting and the Löwen criterion^658^ for crystallization and can be applied both to normally and anomalously melting solids.

#### 10.1.3 Scaling

Another approach is based on scaling considerations. Already indicated is the argument by Hoover and Ross^27,58^ that, if repulsion dominates, a one-phase model still might work, later described by Dyre^659,660^ as “hidden scale invariance”. This approach was applied by Pedersen et al.^4^ in a form for which properties of the coexisting crystal and liquid phases at a single thermodynamic state point provide the basis for calculating the Δ_mel_*P*(*T*), Δ_mel_ ρ(*T*), and Δ_mel_*S*(*T*) along the melting line using this scaling concept. The change of the (reduced) Lindemann ratio (as well as the liquid’s diffusion constant and viscosity) along the melting line could also be calculated. The theory quantifies the deviations from predicted hard sphere melting-line invariants and is validated by simulations of the standard 12–6 LJ system. It is claimed that the theory applies for the sizable class of systems characterized by hidden scale invariance, but mechanisms are not discussed.

**Figure 26:**
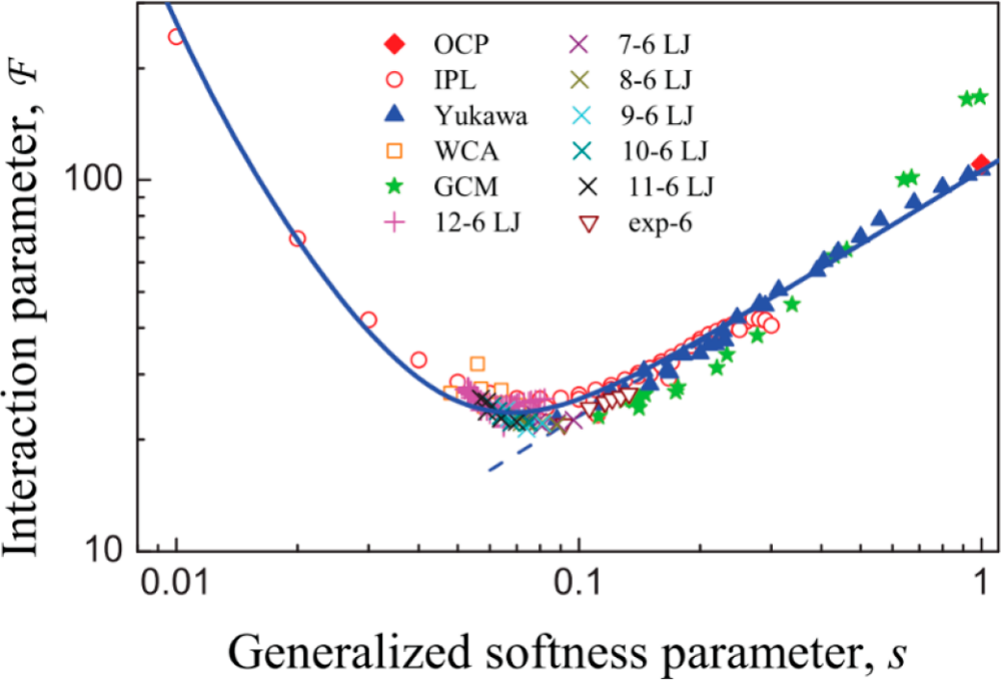
Universal melting curve represented by *F*(*s*) with *F* the interaction for softness parameter *s*. The symbols represent the various numerical data, while the solid curve represents the fit. Reproduced with permission from ref (661). Copyright 2011 AIP Publishing.

In a related approach Khrapak et al.^661^ attempted to obtain a universal melting curve by proper scaling for a wide range of potentials. These authors used also as reference potential the inverse power law *U*(*r*) = ε(σ/*r*)^*n*^ with ε and σ as scaling parameters for the energy and length, respectively, and *n* a constant. From the work of Hoover et al.^83^ it is known that the single parameter *Γ* = (*T*/ε)(*Nσ*^3^/*V*)^*n*/3^ for *N* particles in volume *V* can describe inverse power liquids. However, the authors used the parameter *nΓ* to characterize the force at the mean interparticle distance Δ = (*V*/*N*)^1/3^. Further, for an arbitrary potential *U*(*r*) they required that *U*″(*r*) = *U*′(*r*), where *U*′ is the first derivative and *U*″ is the second derivative with respect to distance *r* at *r* = Δ. This leads to a (generalized) “softness parameter” *s* = [−1 – *U*″(Δ)Δ/*U*′(Δ)]^−1^ and a (generalized) “interaction parameter” *F* = −*U*′(Δ)Δ/*T*. Writing *U*(*r*) = *εu*(*r*/σ) and *x* = *r*/σ, the inverse power law becomes *u*(*r*) = *x*^–*n*^. Similarly, they used the Yukawa potential *u*(*r*) = *x*^–1^ exp(−*x*), the LJ potential *u*(*r*) = 4(*x*^–12^ – *x*^–6^), the exp-6 potential, and the Gaussian form *u*(*r*) = exp(−*x*^2^). It appeared that all data fall almost on a single curve, labeled as the universal melting curve. Using *F*_HS_ ≅ (ln 2/*s*)(1.041)^1/3*s*^, describing the hard sphere limit, and *F*_SS_ ≅ 106*s*^2/3^, describing the soft sphere limit, the authors showed that *F* = (*F*_SS_ + *F*_HS_)^1/ν^ as a function of *s* with ν = 8/5 can be used as a reasonable interpolation formula over the whole range of *s* (Figure 26). In the expression for *F* the hard sphere diameter was approximated by exp[−*εu*(*x*)/*T*] with *T* the temperature. In view of the “remarkably good job” done by this expression, to quote the authors, they suggest that, although this expression cannot replace a proper thermodynamic description, it can with little effort predict the melting transitions for a wide range of conditions.

#### 10.1.4 Density Functional Theory

The theoretical approaches to bulk melting and freezing start from either the liquid phase or the solid phase. Generally, if the liquid phase is taken as a starting point, such an approach deals primarily with freezing. Starting from the solid side, the focus is on melting or freezing; see, e.g., refs (662 and 663). Combining aspects from both the liquid and solid sides remains difficult.

A rather different way to discuss structures and dynamics of liquids and solids is using DFT. Briefly, following the outline by Löwen^17^ for simple systems, i.e., one-component systems consisting of particles with mass *m* interacting via pairwise forces derivable from a spherical symmetric potential *V*(*r*) and where *r* denotes the mutual particle distance, the starting point is a trial grand canonical energy functional *Ω*_tri_(*T*,μ,*w*) = tr{*w*[*H* – *μN* + *kT* ln *w*]}. Here the trace “tr” indicates tr{·} = (*h*^3*N*^*N*!)^−1^∫(·) d***r*** d***p***, dependent on the *N* coordinates ***r*** and momenta ***p*** of the particles, μ is the chemical potential, and *w*(***r***,***p***) is a distribution function. The Hamilton function *H*(***r***,***p***) = *H*_kin_ + *H*_int_ + *H*_ext_ = ∑_*j*_*p*_*j*_^2^/2*m* + (1/2)∑_*j*≠*l*_*V*_int_(*r*_*j*_ – *r*_*l*_) + ∑_*j*_*V*_ext_(*r*_*j*_) is representing the kinetic energy, the internal interaction potential, and an external potential. In equilibrium *w* is given by *w*_0_ = *Ξ*^–1^ exp[−β(*H* – *μN*)], where *Ξ* is the grand canonical partition function. Gibbs–Bogoliubov can be invoked to minimize via *Ω*_tri_(*T*,μ,*w*) – *Ω*_tri_(*T*,μ,*w*_0_) = *kT* tr(*w* ln *w*) – tr(*w* ln *w*_0_) > 0.

For a fixed internal potential *V*_int_(*r*_*j*_ – *r*_*l*_), the distribution function *w*_0_ is determined entirely by the external potential *V*_ext_(*r*). One can show that *V*_ext_(*r*) is uniquely determined by the equilibrium density ρ_0_(*r*). This implies that *w*_0_ is a functional of ρ_0_(*r*), which we indicate by *w*_0_[ρ_0_]. It can be proven that that any positive density ρ(*r*) can be viewed as an equilibrium density for a system in a suitable external potential *V*_ext_(*r*).^664^ Consequently, the Helmholtz energy *F*(*T*,[ρ]) = tr{*w*_0_[ρ](*H*_kin_ + *H*_int_ + *kT* ln *w*_0_[ρ])} is a well-defined functional of ρ[*r*] so that one can construct another functional, *Ω*(*T*,μ,[ρ]) = *F*(*T*,[ρ]) + ∫*ρV*_ext_(*r*)d***r*** – μ∫ρ(*r*)d***r***. If one takes the latter at the equilibrium density, one obtains *Ω*(*T*,μ,[ρ_0_(*r*)]) = *Ω*(*T*,μ). As the equilibrium density ρ_0_ minimizes the functional *Ω*(*T*,μ,[ρ(*r*)]) to *Ω*(*T*,μ,[ρ_0_(*r*)]), one obtains the functional derivative δ*Ω*(*T*,μ,[ρ])/δρ|_ρ=ρ_0__ = 0. This variational principle is used to optimize a density ρ having some parameters and to estimate at the minimum *Ω*(*T*,μ,[ρ(*r*)]). A full account of DFT is given in various papers by Evans^288,665,666^ to which we refer for further details.

As for practical calculations, the functional is not known exactly; one needs to use an approximate functional with an educated guess for the density, which corresponds to an experimentally realizable density. If two solutions with equal grand canonical energy for a given *T* and μ result, this is interpreted as the coexistence of the two realizable densities. In this way, one obtains a mean-field-like solution which is useful to study phase transitions, as one gets approximated Helmholtz energies for the solid and liquid states that can be used to construct the bulk phase diagram. Often used approximations are the local density approximation (LDA), where the excess energy over the ideal fluid result is taken to be local, and the LDA plus a nonlocal mean field approximation where the density is taken as quadratic. Such an approximation can be used if the inhomogeneity is not too strong.

The first practical calculations were made by Ramakrishnan and Yussouff,^667^ rapidly reformulated by Haymet and Oxtoby.^663^ Thereafter better but more complicated functionals were constructed in such a way that they reproduce the direct correlation function for any density in the homogeneous limit. In particular, a so-called weighted density approximation (WDA) has been proposed by Tarazona^668,669^ and Curtin and Ashcroft.^289^ The modified weighted density approximation is computationally simpler than the original WDA but is not applicable to interfacial situations. Thereafter many results were obtained for HS, soft core, Yukawa, and LJ systems, early reviews being refs (666 and 670). Application to the solid state has been discussed by Lutsko and Schoonen.^671^

DFT was applied to interfacial systems as well. In relation to interfaces the best procedure would be to calculate the density profile and interfacial tension from a minimization of the energy functional *Ω*(*T*,μ,[ρ]) using two coexisting phases. However, this is still numerically challenging and therefore approximations like the gradient expansion and (generalized) Landau type models are used, forming a basis for the van der Waals type models, which in the end are relatively simple.

Full interfacial DFT calculations can yield substantial insights. For example, Ohnesorge et al.^248^ investigated by WDA-DFT the equilibrium structure of planar crystal–fluid interfaces in HS and LJ systems, the latter potential approximated by two Yukawa potentials for numerical reasons. The authors computed surface tensions as well as interfacial density profiles and studied for various rare gas crystal orientations the onset of surface melting. A comparison with previous constrained variational calculations demonstrated that unconstrained minimization of the energy functional is indispensable to obtaining reliable values for the surface tension.

In their work the interfaces between the fluid and the FCC crystal for HSs were found to have a width of typically seven hard sphere diameters, while for the surface tensions γ(100) = 0.35*kT*/σ^2^, γ(110) = 0.30*kT*/σ^2^, and γ(111) = 0.26*kT*/σ^2^ resulted, where σ is the diameter. For the interface between a planar hard wall and the liquid or (111) solid, γ_cw_(111) = −2.80*kT*/σ^2^ and γ_fw_ = −2.50*kT*/σ^2^ were obtained, respectively (for the negative sign, see ref (672)), resulting in complete wetting of the hard wall by the (111) surface.

For a LJ system the authors showed that the (111), (100), and (110) FCC crystal–gas interface exhibited complete surface melting near the triple point *T*_tri_ for different surface orientations. The width of the interfacial quasi-liquid layer depended significantly on temperature and surface orientation, and lateral order in the (110) surface is strongly anisotropic. As discussed, the melting process in thermal equilibrium starts below *T*_tri_ by wetting the crystal–vapor interface with a quasi-liquid film. A growth law upon approaching *T*_tri_, as estimated from the interfacial Helmholtz energy,^673^ is given for τ = 1 – *T*/*T*_tri_ → 0 by *l*(τ) = *C* ln(τ_0_/τ), and is predicted to hold on a scale determined by the decay length of the residual crystallinity in the film. With increasing width, a crossover to power law *l*(τ) ∼ τ^–1/3^ is expected because the vdW attraction governs the interaction between the crystal–liquid and the liquid–vapor interfaces of a thick film. From the results for three τ-values the constant *C* was estimated to be 1.3 ± 0.2σ_BH_ or about 2 layers, where σ_BH_ is the effective temperature dependent diameter according to the Barker and Henderson perturbation theory.^674^ At *T*_tri_, γ(100) = 0.29ε/σ_BH_^2^, γ(110) = 0.27ε/σ_BH_^2^, and γ(111) = 0.23ε/σ_BH_^2^, in reasonable agreement with simulation data, while the liquid–gas surface tension was γ = 0.40ε/σ_BH_^2^. Clearly, the essentials of the melting process for simple particles at planar interfaces are well-caught by DFT.

Although a great deal of progress has been made, it might be good to keep in mind that Evans^288^ warned that for an arbitrary proposed EoS it is easy to forget that the underlying Hamilton function is probably unknown or does not exist. For several attempts, see, e.g., the papers by Löwen.^17,675^ More recent are the reviews by Lutsko^676^ on DFT theory, Emborsky et al.^677^ on polymers and polyatomic molecules, and te Vrugt et al.^678,679^ on dynamical DFT.

#### 10.1.5 Various Other Models

An interesting attempt to quickly estimate *T*_mel_ by Prestipino^680^ is based on the Lindemann rule and a description of the solid as an elastic medium. It is able to capture, with negligible computational effort, the overall characteristics of the melting line of a system. The author also describes in a compact way all the required details of the relevant theory, including an account of linear elasticity and the Mansoori–Canfield theory,^2^ which is as such a useful result. Further, Khrapak and Saija^681^ examined various phenomenological freezing and melting indicators for the exp-6 and Gaussian core potentials, while the application of cell models to the melting and sublimation lines of the Lennard-Jones and related potential systems in the light of lattice models was investigated by Heyes et al.^682^

A somewhat deviating view is presented by Garai,^683^ who describes melting as resonance between the energy of the vibrating atoms in the surface layer and, if this “uniform” energy is higher than the energy corresponding to a metastable transition state, supposes that then all the surface atoms lose their position stability.

In a rather different approach, largely based on the different symmetry considerations for the solid and liquid state, Yukalov^684^ presented a general theory taking into account fluctuations in both the solid and liquid phases. The discussion is of a general mathematical–physical nature, and the theoretical results were not applied to concrete materials.

A somewhat esoteric approach is based on the observation that high-temperature heat capacity data reported by different authors can differ from each other more than reasonably can be attributed to experimental errors. Köbler and Bodryakov^685^ showed for the metals V, Nb, Ta, Mo, and W that the individual data sets could be described by a “critical” power function ∼(*T*_mel_ – *T*)^*a*^ which seems to hold for data above the classical Dulong–Petit value of 3*R*. They argue that the large validity range of the critical power function is not of atomistic origin but must be attributed to a field of guiding bosons, the nature of which is not specified. These postulated bosons are supposed to be “evidently” excitations of the continuous solid with energies much larger than the atomistic excitations, i.e., the phonons. While the value of *T*_mel_ is not predicted, the variability of *C*_*P*_ could be explained by a mean free path for the bosons on the order of magnitude of the size of the sample. However, the increase of *C*_*P*_ above 3*R* can also be well explained by the interstitialcy theory^169^ and the phonon theory of liquids.^686,687^ A paper by Harrison^688^ can be considered as a conceptual and qualitative precursor of these ideas.

Another somewhat esoteric approach is by Novikov^689^ showing that at sufficiently high temperature a crystal lattice is unstable with respect to transition into a space with constant negative curvature representing the melting of a crystal. This curvature is proportional to the density of disclinations in real physical space. The melting temperature was obtained as a functional of the interatomic pair potential and to a good approximation depending only on the second derivative of the potential at its minimum, i.e., on the bulk elastic modulus. It was shown that the Lindemann criterion is satisfied with the mean square atom displacement at *T*_mel_ expressed in terms of the first zero of the interatomic potential. For the LJ and Morse potentials approximate values of ξ = 0.135 and 0.15, respectively, resulted, while for various metallic glasses a range of 0.14–0.17 followed. The disclination density and the size of the regions in which the short-range crystalline order in the melt is preserved were estimated as well.

A very different approach for the melting of elements was given by Hoffmann.^91^ The author argued the molar specific heat capacity *C*_*P*S_ of the solid elements just below *T*_mel_ shows a rather broad distribution ranging from about 3*R* to 7.5*R*, which does not change significantly when *C*_*V*S_ is calculated from *C*_*V*S_ = *C*_*P*S_ – α^2^*VK*_*T*_*T*, and of which a major part of *C*_*V*S_ exceeding the value 3*R* is due to the contribution of the electrons to the specific heat, as estimated from *C*_*V*e_ ≅ *γT*. Both corrections used room temperature data in the absence of the required data near *T*_mel_. Further, he argued that the conduction bands with a strong influence of atomic wave functions with d or f character are narrow and possess very large densities of states, whereas bands made up of wave functions with s and p characters are broad on the energy scale and possess a lower density of electronic states per energy interval. Consequently, the large molar heat capacity exceeding 3*R* near the melting point is essentially attributed to contributions of the electrons in bands built up of wave functions with d and f symmetries. Roughly, the Fermi energy *E*_F_ shifts with increasing temperature to lower (higher) energy if the effective density of unoccupied states above *E*_F_ is larger (smaller) than the effective density of occupied states at or below *E*_F_, and levels occupied at lower temperature become depleted, and other levels with larger energy will be occupied with increasing temperature. The wave functions in both cases are different, resulting in different probability distributions of the respective electrons. In addition, wave functions of unoccupied states may form hybrids with neighboring occupied states. Thus, the surrounding electrical potential of the core ions is modified, and the resulting potential gradients transfer a considerable momentum to the core ions. If the forces are strong enough and act long enough, the core ions will relax to new positions with lower energy. This relaxation is favored if the agitation of the core ions is large, and thus relaxation occurs preferentially at higher temperatures. The distribution of the electrons over the states, however, changes simultaneously with as a result the core ions continuously relax to new positions. If the relaxation is fast enough, i.e., if it occurs during the lifetime of a specific distribution of the electrons at sufficient different locations in a solid, the position of the core ions changes continuously with time, which is a description of a fluid. In this view it seems quite improbable that a core ion can surmount the energy barrier to a new site if the electronic configuration and the charge distribution remain approximately constant. Instead, it will relax to its former position, since the unchanged charge distribution favors a shift to its energetic minimum. Thus, the large deviations from the previous charge distribution (which is stable at low temperatures) will facilitate the transition to new sites with lower energy, thereby providing an explanation of melting.

Finally, we note that the so-called “set/liq” model for melting was proposed by Galwey.^690,691^ The starting point is that the enthalpy and density changes that occur on melting are relatively small, leading to the assumption that, at *T*_mel_, no significant changes in component sizes or shapes, intercomponent bonding, constituent packing control, or other physical properties (except viscosity) occur. In this model the liquid is represented by dynamic equilibria between small domains composed of particles packed in alternative lattice arrays that rapidly and continually interconvert. These domains are supposed to be small, say, a few nanometers, so that they escape XRD detection. Each domain is bounded by less ordered interfaces that maintain coherent contact with all contiguous domains, analogous to a grain boundary, and provides lubrication for ready relative movements of the domains, thereby accounting for fluidity. The essential feature is thus the continual rapid interconversion of small zones, representing equilibrium between all possible, locally ordered domains. The model resembles strongly the significant liquid structures (SLS, see section 10.2) model and the “hole” model of Frenkel,^111^ although no references were made. With respect to SLS theory, the differences are that the ordered domains contain all the various lattice types a crystal can have instead of only the most stable one, and the interfacial zones are also densely but randomly packed instead of being gas-like. The “hole” model describes a liquid, in the words of Frenkel, as particles “distributed in a non-uniform way forming more or less compact ‘bunches’, separated from each other by fluctuating cracks, or, to be more exact—a relatively compact mass with a density but slightly below that of the corresponding crystal by fluctuating fissures”. The only difference with the “set/liq” model seems to be the presence of only one type of ordered domain, based on the most stable lattice type. With this qualitative model at hand, many aspects of melting for various types of materials were discussed. Han and Kim^328^ also proposed a similar model containing rotationally free atomic clusters, however, referring neither to Frenkel nor to Galwey.

### 10.2 Two-Phase Approaches

A two-phase approach by Vorob’ev,^3^ already indicated in section 5.2, used tractable models for both phases. He used the Debye model for the vibrational part of both the solid and liquid states. Another option used is based on using significant liquid structure (SLS) theory for the liquid phase. The SLS theory^692,693^ is based the following considerations and experimental facts: (a) For a normal phase transition from solid to liquid, there is always an expansion in volume, water (and a few other compounds) being considered an exception. (b) At the triple point the vapor pressures of both solid and liquid states are the same. (c) X-ray diffraction studies have shown that the intermolecular spacing (time average) among nearest neighbors in a liquid is similar to that found in the solid. The long-range order in the solid, however, disappears in the liquid, but there still is some short-range order remaining, which, upon increasing the temperature, gradually disappears. In fact, a quasi-lattice model for the liquid is accepted. The basic idea of SLS theory is that only those structures which make the major contribution to the thermodynamic properties of the liquid are singled out and any others are ignored. Three significant structures are considered: (a) The first is the solid-like degrees of freedom possessed by molecules having only other molecules as nearest neighbors. In its simplest form SLS theory uses the Einstein model for these degrees of freedom. (b) The second is the gas-like degrees of freedom possessed by molecules having a vacancy or vacancies as nearest neighbors. They will have three-dimensional translational degrees of freedom by virtue of their ability to move into the neighboring hole(s). (c) The third is the positional degeneracy of solid-like molecules: because of the existence of molecular size holes, a solid-like molecule will have a positional degeneracy other than its most stable equilibrium lattice position. This positional degeneracy is proportional to the number of neighboring holes which exist and inversely proportional to the energy required to preempt the neighboring hole from the competing neighboring molecules.

For Ar, Tuerpe and Keeler,^694^ using SLS theory with the Einstein model for the solid-like domains of the liquid state as well as the Einstein model for the solid state, studied its high-pressure melting transition. While at *P* = 0, the predicted values for *T*_mel_ (calculated 80.0 K, experimental 83.9 K) and *V*_m_ (calculated 27.8 cm^3^ mol^–1^, experimental 28.0 cm^3^ mol^–1^) were reasonable, the pressure dependence deviates for *P* > 3 kbar substantially from the experimental curve. The authors indicate as the main reason the use of the same model for the solid state and the solid-like part for the fluid. With increasing pressure, the fraction of solid-like molecules increases, possibly rendering discrimination between an increase of solid-like molecules and the formation of a second phase of solid molecules impossible.

Using the Debye model for the solid state as well as the solid-like part of the liquid phase but allowing for a different value of θ_D_ and meanwhile using for both phases a slightly different but constant Grüneisen parameter and a simple expression for the potential energy, Kanno^695^ was able to describe using SLS theory the pressure dependence for Ar much better. He also derived several other thermodynamic data, such as the change in melting entropy upon fusion and the internal energy and entropy for both phases as a function of *P*, also in good agreement with experiment, but clearly states the approximate nature of his calculations.

Using a modified SLS theory, Levitt and Hsieh^696^ discussed the melting of solids based on the assumption that the coordination number changes upon melting. In the brief description of this theory given above, the Δ*V* upon melting is solely attributed to the introduction of extra holes, while the coordination number remains the same. The authors now assumed the coordination number does not remain the same upon melting. They defined a “coordination” factor κ = 1 + Δδ/*V*_S_ with Δδ = δ_L_ – δ_S_, so the product *κV*_S_ represents the molar volume of a newly coordinated liquid quasi-lattice with a “dead” space δ_L_, i.e., the interstitial space in the lattice inaccessible to molecules, different from δ_S_ of the solid lattice. In these considerations the volume as occupied by the molecules is calculated from the molar refraction *R*_m_ = (*n*^2^ – 1)*M*/(*n*^2^ + 2)ρ_S_. In the latter expression, *n* is the refractive index, *M* is the molecular weight, and ρ_S_ is the density of the solid, and because *R*_m_ represents the volume actually occupied by the molecules, the relation *R*_m,S_ = *R*_m,L_ was used. In SLS theory the mole fraction of holes is *V*_h_/*V*_S_ = (*V*_L_ – *V*_S_)/*V*_S_ and the mole fraction of solid-like molecules is *V*_S_/*V*_L_, and if *n*_h_ is the number of equilibrium sites accessible to a solid-like molecule in addition to its single most stable position, then *n*_h_ = *z*(*V*_L_ – *V*_S_)/*V*_L_ with *z* the number of nearest-neighbor sites, or the coordination number, of the solid. But *n*_h_ should also be proportional to the mole fraction of holes, so *n*_h_ = *n*(*V*_L_ – *V*_S_)/*V*_S_ with *n* another proportionality constant. Eliminating *n*_h_ yields *n* = *zV*_S_/*V*_L_, which means that *n* is the number of nearest-neighboring sites occupied by molecules (not holes) in the liquid and that *n*_h_ + *n* = *z*.

Further they defined a “vacancy” factor χ = *N*_ext_/*N*_0_, where *N*_ext_ is the total number of “extra” new holes formed in the liquid phase containing *N*_0_ molecules, assuming that Δ_m_*H* can raise the solid temperature to *T*′ = *T*_mel_ + Δ_m_*H*/*C*_*P*_ without melting it, given by
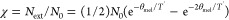
125

This is also the number of holes in the liquid state at *T*_mel_ because before melting the number of holes (vacancies) is negligible. Accordingly, the number of holes being *V*_h_ = *κV*_S_χ = *V*_L_ – *χV*_S_, the relation κ = *V*_L_/*V*_S_(1 + χ) results.

Accepting the Lindemann rule, the characteristic (Einstein) temperature was estimated as θ_E_ = *c*(*T*_mel_/*mV*^2/3^)^1/2^ using the value *c* = 135 but for the noble gases the value *c* = 163, as given by Clusius.^697^ The authors could have reversed this procedure using experimental θ_E_ values, thereby estimating *T*_mel_, but with Hoffmann’s remarks in mind (section 5.2) this would not have been wise. From calculations for Ar, Kr, Xe, N_2_, H_2_O, and Hg, it appeared that κ ≅ 0.68–0.9. This suggests that the packing for the quasi-lattice of the solid-like molecules in the liquid is denser than in the solid. Taking the quasi-lattice concept literally, this is difficult to imagine for close-packed lattices like FCC, but it should be noted that for the quasi-lattice concept to be acceptable only a relatively dense packing, regular or random, with a well-defined coordination number is required. An independent check is given by a comparison of *n* as calculated with the value as determined by XRD. For Ar, Kr, Xe, and H_2_O, these values agree remarkably well (experimental *n*-values for N_2_ and Hg were unavailable to Levitt and Hsieh). Unfortunately, a promised follow-up never seems to have been published.

A two-phase approach was also given by Mansoori and Canfield^698^ based on their variational approach to liquids^2^ using a perturbation approach for hard spheres (HSs) with as perturbation attractive interaction as described by a LJ potential. Their variational expression is rather similar to one derived from the Gibbs–Bogoliubov inequality, but apparently derived independently. Because the HS pair correlation function in the Percus–Yevick approximation is known analytically in the Laplace domain,^699^ the authors were able to express their results analytically.^700^ Their results for the liquid–vapor phase diagram for LJ particles are in rather good agreement with MC simulation data. To discuss the solid–liquid transition, the authors used the LJD cell theory^130,131^ in combination with the pair correlation expression.^701^ Both the sharp change in the (only) variational parameter and the equality of Gibbs energy for liquid and solid as a function of *T* were used. While for the first method the densities of coexisting liquid and solid phases versus *T* were in poor agreement with the experimental data for Ar as given by McDonald and Singer,^702^ the second method did show reasonable agreement. McDonald and Singer’s MC simulation data for LJ particles representing Ar did agree reasonably as well with experiment.

Weeks and Broughton^703^ discussed a two-phase approach based on the vdW equation, depending on whether a clear separation of the intermolecular force into a short-ranged and repulsive part operative at small separations and a longer-ranged, more slowly varying attractive part operative at larger separations cam be made. Using the Weeks–Chandler separation of a LJ potential ϕ_LJ_ = 4ε[(σ/*r*)^12^ – (σ/*r*)^6^] in a reference system with potential ϕ_0_ and a perturbation with potential *u*, i.e., ϕ_LJ_ = ϕ_0_ + *u* (with ϕ_0_ = ϕ_LJ_ + ε for *r* ≤ *r*_0_, ϕ_0_ = 0 for *r* > *r*_0_, *u* = −ε for *r* ≤ *r*_0_, *u* = ϕ_LJ_ for *r* > *r*_0_, and *r*_0_ = 2^1/6^σ), the Helmholtz energy *F* can be written as
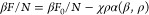
126while the internal energy *U*, pressure *P*, and chemical potential μ become
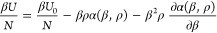
127
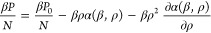
128and
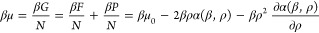
129

Here *N* is the number of particles, ρ = *N*/*V* is the density, *G* is the Gibbs energy, and β = 1/*kT*, while the subscript “0” refers to the reference system. These equations are exact and require knowledge of α(β,ρ). Weeks and Broughton^703^ now argue rather plausibly that, at least for qualitative discussion, α(β,ρ) can be considered as constant. Good agreement with the full LJ pair correlation function *g*(*r*) for liquids and reasonable agreement for *g*(*r*) for solids was obtained. If α(β,ρ) = *const*. is assumed, attractive interactions have no effect on the entropy of the system, i.e., *S* = *S*_0_. To determine the coexisting densities ρ_L_ and ρ_S_ of the fluid and solid phases, equality of *P* and μ is required. If it is further assumed that δρ_*X*_ = ρ_*X*_ – ρ_*X*0_ and defining *K*_*X*_ = (∂*βP*/∂ρ)_*T*_ as derived from the thermodynamic identity (∂*βμ*/∂ρ)_*T*_ = *K*/ρ where *X* denotes either “L” or “S”, a first-order expansion of the relations *P*_L_ = *P*_S_ and μ_L_ = μ_S_ leads after solving to
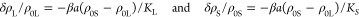
130

Near the fluid–solid transition the system is rather incompressible so that *K* ≫ 1 (e.g., *K* > 50 for the LJ fluid) and the assumption of a small δρ_*X*_ is reasonably accurate. Equation 130 shows that the effect of attractions is to widen the density change on melting, increasingly so as the temperature is decreased. Although in principle a full calculation could be done, such a calculation seems not to be available.

A two-phase approach based on the Bogoliubov–Born–Green–Kirkwood–Yvon (BBGKY) equation^704^ was advocated by Jacobs.^705^ Although the BBGKY equation was devised for liquids, it can be applied to solids as well, given that a proper decoupling approximation and pair correlation function *g*(*r*) can be obtained. Overall, the theory is involved but in detail clearly explained, in particular in its extended form given by Jacobs and Cheung,^706^ to which we refer for details. The latter presentation takes into account a better decoupling approximation and a better *g*(*r*). It also takes into account the communal entropy correction by using the estimates from Hoover and Ree,^34^ which, although small, is not negligible. Moreover, for the volume change Δ*V* = *V*_L_ – *V*_S_, needed for converting the Helmholtz energy *F* to Gibbs energy *G* = *F* – *C*, it was assumed that λ = −(∂*V*/∂*P*)_*T*_ is independent of *V* with its value taken from the experimental EoS (although it could be calculated in principle from the potential used). Given λ, the energy correction *C* = −(1/2)(Δ*V*)^2^/λ was calculated iteratively. Jacobs and Cheung^706^ dealt only with Ar as at that time the EoSs for the other rare gas solids were unknown to them. Using the LJ potential, they calculated *T*_mel_, the melting entropy Δ_m_*S*, the Lindemann ratio ξ, and the volume change Δ*V*. Table 6 shows their results from the original calculation and from its extended form. Clearly very good agreement with experiment is obtained, although clearly for one solid only, while the approach would be rather difficult to extend to more complex compounds, so maybe it is no surprise that no follow-up seems to exist.

**Table 6:** Various Data for Ar as calculated by Jacobs and Cheung

	*T*_mel_ (K)	Δ_m_*S*/*k*	1/ξ	Δ*V* (cm^3^ mol^–1^)
exp.	84	1.77	7	3.61
extended calc,	79	2.33	9.5	3.85
original calc,	63	4.0	13.6	–

Finally, we refer to the most sophisticated two-phase approach, hierarchical reference theory (HRT), which was introduced by Parola and Reatto as a new and accurate method to evaluate the EoS of fluids.^707^ Because this theory is relatively complex and two detailed reviews^708,709^ exist, we limit the discussion to qualitative remarks. HRT was inspired by the momentum-space renormalization group,^710^ and as this method came from field theory, with HRT one adds for a given temperature Fourier components (wave vectors) to the Fourier transform of the perturbing interaction ϕ_pt_ until the full interaction is obtained. Further the pair correlation function is assumed to be of the Ornstein–Zernike (OZ) form^711^ with a free parameter to be determined from a self-consistent partial differential equation between the corresponding expressions for the Helmholtz energy and compressibility. From numerical work accurate results for the EoS of continuum fluids resulted, where in particular the critical region was well described with good results for the critical exponents.^708,709^

Another accurate approach to obtain the equation of state is the self-consistent Ornstein–Zernike approximation (SCOZA) by Høye,^712^ where the OZ equation of fluid theory in combination with the mean spherical approximation (MSA) is used. In the MSA the direct correlation function outside hard cores is assumed to be −*βϕ*_pt_ with, as usual, β = 1/*kT*. With SCOZA, β is replaced by an effective inverse temperature as a free parameter to be determined via thermodynamic self-consistency between the internal energy and compressibility. Again, very accurate results came out, also in the critical region, and various generalizations have been dealt with.^713^

A unification of HRT and SCOZA was made by Høye and Reiner^714,715^ and further analyzed in the critical region^716^ where the two free parameters were found to be essentially determined by the sum of the HRT and SCOZA problems. Since these two theories qualitatively have somewhat different critical behaviors, the problem to reconcile them implied the presence of subleading scaling terms. This was investigated by analytic and numerical work^717^ by solving the HRT partial differential equation. It was found that the HRT critical indices were simple rational numbers. By direct investigation of the HRT partial differential equation^713^ using both analytical and numerical methods, the critical properties of HRT were also determined by the solution of a transformed HRT partial differential equation that was expanded in leading and subleading scaling contributions that fulfill ordinary differential equations. These contributions are connected via simple powers of the cutoff parameter of renormalization, leading again to simple rational numbers for the critical exponents. However, the derivations are not fully valid near the fixed-point solution due to some divergence problems, but it was expected that this uncertainty is insignificant for the expansion in powers of the cutoff parameter and thus for critical properties.

The application of HRT to melting seems so far to be limited. Although providing flat isotherms in the two-phase region, an expression for the Helmholtz energy still must be provided. Parola et al.^708^ used for the solid the second-order Barker–Henderson perturbation theory^674,718^ in which the second-order term was estimated by the “macroscopic-compressibility” approximation. Comparison with Monte Carlo simulations on depletion potentials^719^ showed that, although this second-order estimate deviates substantially from the exact value, the sum of first- and second-order terms is nevertheless generally quite close to the simulation results for the full Helmholtz energy. In fact, the first-order approximation has been shown to be more accurate in the solid than in the fluid^720^ provided that the solid under study has the same lattice structure as the reference. The Helmholtz energy was obtained by integrating with respect to density the equation of state of the HS solid.^721^ The fluid–solid phase boundary was determined by equating *P* and chemical potential μ of the solid to those of the fluid as given by smooth cutoff HRT using the hard-core Yukawa potential ϕ = −ε exp[*−z*(*r* – σ)]/*r* with inverse range *z*. The freezing line becomes wider with increasing *z*, and if the freezing line is tangent to the fluid–fluid coexistence curve at the critical point, the stable fluid–fluid transition regime is separated from the metastable regime. This occurs at *z* = 5.6, to be compared with *z* = 5.7 for SCOZA and *z* = 6 for MC simulations. Similar calculations were done earlier with sharp cutoff HRT using the hard-core Yukawa^722^ and a two-Yukawa potential with competing attractive and repulsive interaction.^723^

HRT has been developed and used mainly by Parola and Reatto and has found its application so far mainly in colloidal systems; see, e.g., refs (720, 722, and 723.) The existing theory is limited to spherical potentials, so for molecular systems essentially only atomic compounds can be handled. Overall, the amount of work involved and the relative complexity of the theory, which limit its general use, may not outweigh the relatively easy use and wide availability of simulation methods. The latter is associated with less (conceptual) complexity and can incorporate (much) more realistic potentials, also for nonatomic compounds.

### 10.3 Colloidal Systems

While hard spheres always played a prominent role in modeling and simulations, nowadays hard sphere interactions can be realized by colloidal particles.^36^ This implies that colloidal systems can be used as models for atomic systems, quite apart from their intrinsic interest. To mimic repulsion both charge and steric stabilization can be used, but we limit the discussion here mainly to steric stabilization. To realize steric stabilization, the colloidal particles are coated with polymer brushes, which leads to an “entropic” repulsion if polymer brushes of two neighboring particles overlap. Since the length of a polymer chain typically is much smaller than the colloidal diameter σ, one can describe this repulsive force by the pairwise hard sphere potential. To suppress the ever-present van der Waals interaction, one usually matches the refractive index of the dispersing medium and that of the colloidal particles, as the van der Waals interaction is directly reflected in the refractive index. In this way the hard sphere interaction dominates the interparticle forces. The prototype materials for such colloidal particles are PMMA and PS, and careful experiments on the structure and the phase diagram reveal that the interaction between such particles can be well described by excluded volume effects only. Similarly as for atomic systems, the density ρ = *N*/*V*, the packing fraction η = π*ρσ*^3^/6, and the pair correlation function *g*(*r*) are the characteristic parameters. The main determining characteristic is the packing fraction, which can be varied from a few percent to about 74%, the latter being the limit for close-packed crystals of monosized particles. For colloidal hard sphere systems, the temperature *T* is in principle only determining the kinetics, as long as the stability is not ruined by temperature effects.

Atomic and colloidal systems show similarities and dissimilarities. The main similarity between atomic and colloidal systems is that they both can be represented by classical statistical mechanics using interactions that can be described in terms of a pair potential. Another similarity is that they pack in well-known lattice types, such as FCC and BCC. One dissimilarity is that the interactions in colloidal systems can be tuned, while for atomic systems they are fixed. Another dissimilarity is the length scale involved, which is in the angstrom range for atomic systems and in the nanometer range for colloidal systems. The latter implies that diffraction effects are in the optical range for colloidal systems while X-rays are relevant for atomic systems. It also implies that the time scale is rather different, typically 0.1 ps for atomic systems and several thousand nanoseconds for colloidal systems. For colloidal systems the matrix (solvent) also plays an important role as hydrodynamics become important for more dense systems. Finally, for colloidal systems obviously size dispersity must be tightly controlled if they are to be used as a model for atomic systems. A short introduction is given by Murray and Grier,^724^ while the book edited by Caruso^725^ discusses many aspects of colloids and their assemblies. A detailed review of the design and structure and hierarchy of colloidal systems, both 3D and 2D, has been given by Vogel et al.^726^ The field is large, and below we address some typical results for both bulk melting and melting of (near) monolayer systems.

#### 10.3.1 Melting in 3D

Zahorchak et al.^727^ studied colloidal suspensions in the presence of ions by MC simulations at *T* = 298 K using constant (*N*,*V*,*T*) conditions, periodic boundary conditions, a screened Coulomb (or Yukawa) potential, and a perfect crystal initial configuration (*N* = 250, BCC; *N* = 256, FCC). The pair correlation function *g*(*r*), the total potential energy *U*, and the mean square displacement ⟨*u*^2^⟩ were determined as a function of ion concentration *n*_*j*_. The authors showed that the parameters *g*_max_ (the maximum of the first peak in *g*(*r*), *S*_max_ (the maximum of the first peak in the structure factor *S*(*q*) as calculated by Fourier transforming *g*(*r*)), Δ*r* (the half-width at half-maximum of the first peak in *g*(*r*)), and *U* all show discontinuous behavior at a certain concentration value *n*_*j*_^mel^, which was interpreted as melting. Slightly different values were obtained for *g*_max_ for both the BCC and FCC simulations for the liquid and solid phases. For the FCC crystal the value obtained was *g*_max_ ≅ 2.85, but for the BCC crystal it was somewhat lower with *g*_max_ ≅ 2.72. The Wendt–Abraham parameter, *R*_*g*_ = *g*_min_/*g*_max_,^728^ where *g*_min_, the minimum value of *g*(*r*) following the first peak of *g*(*r*), is somewhat more sensitive and showed a linear behavior as a function of *n*_*j*_ with a discontinuity at *n*_*j*_^mel^. For the BCC crystal the slope *m* of *R*_*g*_ versus *n*_*j*_, *m* ≅ 0.76, is somewhat larger than *m* ≅ 0.61 for the FCC crystal. The value of *S*_max_ at *n*_*j*_^mel^ in the crystalline state is ≅3.25 for the BCC crystal but decreases to ≅2.76 for the FCC crystal. The corresponding *S*_max_ values in the liquid state are ≅2.52 for the BCC crystal and ≅2.39 for the FCC crystal. Indeed, liquid metals which freeze into the BCC structure give a larger value, *S*_max_ ≅ 3.1, than those that freeze into the FCC structure, *S*_max_ = 2.8, the latter value being approximately consistent with Hansen–Verlet criterion. The authors also compared for a particle in the crystal phase ⟨*u*^2^⟩, as calculated in the usual way by tracking particles, to the approximation ⟨*u*^2^⟩ ≅ 3(Δ*r*)/2 ln 2. The Lindemann ratio, defined as ξ = *n*^–1/3^(⟨*u*^2^⟩)^1/2^, where *n* is the particle concentration, appeared to be ξ = 0.19 ± 0.01 for both the FCC and BCC lattices, similar to other simulation values, but slightly higher than the experimental value ξ = 0.16 obtained by optical ultramicroscopy and image processing^729^ and much higher than the often quoted bulk value ξ = 0.10.

Sulyanova et al.^730^ studied the structural evolution of colloidal crystal films containing 40–50 monolayers of polystyrene (PS) spherical particles, with a size dispersity as measured by dynamic light scattering of 2.1%, from *T* = 293 K to *T* = 381 K by XRD. The Bragg peak position, integrated intensity, and radial and azimuthal widths were analyzed as a function of temperature. A quantitative study of the colloidal lattice distortions and mosaic spread as a function of temperature was carried out using Williamson–Hall plots based on the mosaic block model. A significant increase of lattice distortion and domain misorientation parameters occurred around the annealing temperature *T*_a_ = 355 K. The temperature dependence of the diameter of polystyrene particles was obtained from the analysis of Bragg peaks, and the resulting thermal expansion coefficient was in good agreement with literature data. The form factor contribution was extracted from the diffraction patterns, which resulted in four stages of structural evolution upon heating: steady state, preannealing, shape transformation, and crystal melting. Both the nanoscopic length scale (about the size of a colloidal particle, here a few hundred nanometers) and the mesoscopic length scale (the size of a coherently scattering domain, here a few micrometers) need to be considered. On the nanoscopic length scale, linear growth of the average lattice parameter for *T* < *T*_g_, with *T*_g_ = 373 K the glass transition temperature of PS, was observed, which is directly related to thermal expansion of the PS. For *T* > *T*_g_, the PS particles softened and changed their shape by flattening in the directions where they touched each other, leading to a decrease of long-range order in the crystalline film, observed as a decrease of intensity of higher-order Bragg peaks with increasing temperature. Moreover, the lattice parameter rapidly decreased, indicating a fast shrinkage of the lattice until the crystalline structure completely disappeared at *T*_mel_ = 381 K. On the mesoscopic length scale, no particular changes were observed below the preannealing temperature *T*_pa_ = 323 K, while for *T*_pa_ < *T* < *T*_g_ the structure of the colloidal film showed significant changes. The authors suggested that, due to the presence of cracks and other microscopic defects in the colloidal crystal film, the orientational correlations of mosaic blocks increase for *T* < *T*_a_ and that for *T* > *T*_a_ the crystal lattice becomes more relaxed and a partial annealing process occurs, while for *T* > *T*_g_ coalescence of the PS particles occurs, as evidenced by the sharp decrease of the lattice parameter and integrated intensities of Bragg peaks. This study clearly shows, apart from the intrinsic aspect of such a colloidal system, that a transfer of resulting models to atomic systems should be handled with caution.

Wang et al.^731^ superheated and melted the interior of thermal-sensitive *N*-isopropylacrylamide (NIPA) colloidal single and few-grain crystals, and investigated their homogeneous melting by means of video microscopy at single-particle resolution. By changing the temperature slightly, the volume fraction of the particles, which have an approximate size of 0.76 μm at 26.4 °C to 0.67 μm at 30.6 °C, could be varied, thereby enabling repetitive melting and solidification. The crystalline structure and the good refractive-index matching between particles and water enabled the authors to see through all the about 150 layers of the crystal by means of bright-field microscopy. Local particle-exchange loops surrounded by particles with large displacement amplitudes rather than any defects were observed as nucleation precursors. Under weak superheating, the nucleation kinetics essentially followed classical nucleation theory. Under strong superheating the critical size, incubation time, and shape and size evolution of the nuclei measured deviated from classical nucleation theory predictions, mainly because of the coalescence of nuclei. In an earlier stage Jin et al.^732^ showed via simulations that it seems the Lindemann and Born criteria strongly correlate as melting is initiated by local lattice instabilities governed by both, as put in perspective by Cahn.^733^ In the experimental study by Wang et al.^731^ the authors conclude that the superheating limit for homogeneous melting indeed agrees with both criteria, and in a short perspective paper Weeks^734^ highlighted their work on how colloidal crystals melt “from the inside out”, i.e., by thermodynamic melting.^735^

In a follow-up Wang et al.^736^ divided the nucleation process of homogeneous melting into three stages: (1) an incubation stage in which the superheated crystal remains metastable without forming critical liquid nuclei, although nucleation precursors such as defects^637,737^ or particle swapping loops^330,731^ may form and trigger the formation of liquid nuclei; (2) the formation of critical nuclei; and (3) the growth stage of postcritical nuclei. In this paper the authors focused on stage 3 using a similar experimental setup as described in ref (731) and studied the effects of nucleus size, shape, coalescence, and surface tension on the nucleus growth rate from the melting point to the superheating limit. For the latter the value Δϕ_super_ = ϕ_mel_ – ϕ = 0.09 was used, where ϕ_mel_ = 0.545 is the volume fraction at melting, conform their earlier paper.^731^ To describe their results, they modified classical nucleation theory according to Frenkel^111^ for the nucleus growth in crystallization to the case of melting using Wilson’s results^738^ by adding surface tension and the nonspherical shape effects. This modified theory fitted the measured nucleus growth rates well at weak superheating (Δϕ < 0.025). At stronger superheating, effects not considered in classical nucleation theory were observed. At intermediate superheating (0.025 < Δϕ < 0.05), the growth rate was higher than predicted by the modified Wilson–Frenkel theory. The nonspherical nuclei rotated due to the anisotropy of the crystal surface tension and the shape fluctuated, each contributing to a rate increase up to 10%, even for small postcritical nuclei. At strong superheating (0.05 < Δϕ < 0.06), coalescence of the nuclei through neck formation further increased the growth of nuclei, while at very strong superheating (0.06 < Δϕ < 0.09), the authors observed multimer attachment to nuclei, which again promoted the growth rate significantly. At the liquid–solid interface the Lindemann ratio ξ, as determined from mean square displacement measurements, was about ξ ≅ 0.18, while in the bulk it varied somewhat from ξ ≅ 0.08 at ϕ = 0.528 via ξ ≅ 0.06 at ϕ_mel_ = 0.545 to ξ ≅ 0.05 at ϕ = 0.555. From these results the authors conclude that ξ ≅ 0.18 appears to be physically significant for both heterogeneous (thermodynamic or surface-mediated) melting and homogeneous (mechanical or bulk) melting.

The kinetics of crystal growth and melting of body centered cubic (BCC) and face centered cubic (FCC) crystals colloidal crystals was studied experimentally by Hwang et al.^739^ Particle motion was tracked, and by introducing a structural order parameter, they measured the jump frequencies of particles to and from the crystal and determined from these the Helmholtz energy difference between the phases and the interface mobility. The interface was observed to be rough for both BCC and FCC crystals, while the jump frequencies correspond to those expected for a random walk of the particles, which translates to collision-limited growth in metallic systems. The mobility of the BCC interface is greater than that of the FCC interface. In addition, and contrary to the prediction of some early computer simulations, they showed that there is no significant asymmetry between the mobilities for crystallization and melting.

Other effects have also been studied for colloidal systems. We mention only a few examples. Medina-Noyola and Ivlev^740^ calculated the explicit form of the colloidal particle interaction in colloidal systems of highly charged particles in solution. Based upon the exact interaction, the effect of buckling of the monolayer of colloidal particles in the middle of an electrolyte film was considered and the melting condition of colloidal crystals was determined. The authors showed that thermal fluctuations of inner degrees of freedom of edge dislocations affect the condition of the dislocation-mediated melting.

A study by Peng et al.^741^ on confined particles between two glass plates with tunable interactions for layers of varying thickness revealed different behaviors for thick and thin films. Thick films (>4 layers) melt from grain boundaries in the polycrystalline solid films and from film–wall interfaces in single-crystal films. A liquid–solid coexistence regime is observed in thick films that vanishes at a critical thickness of four layers. Thin solid films (two to four layers) melt into the liquid phase in one step from both grain boundaries and from within crystalline domains. Monolayers melt in two steps with an hexatic phase in between, conform KTHNY theory.

Shear-induced melting and crystallization were investigated with confocal microscopy in concentrated colloidal suspensions of hard-sphere-like particles by Wu et al.^742^ The authors used silica and PMMA suspensions and sheared those with a constant rate in either a countertranslating parallel plate shear cell or a counterrotating cone–plate shear cell, which made it possible to track particles undergoing shear for longer times in a plane of zero velocity. On large scales the flow profile was not linear, but the crystal flowed in an aligned sliding layer structure at low shear rates. Higher shear rates caused the crystal to shear melt, but the transition was not sudden. Although the overall order decreased with shear rate, this melting range was due to an increase in the nucleation of localized domains that temporarily lost and regained their ordered structure. Even at shear rates that were considered to have melted the crystal as a whole, ordered regions kept showing up at times, giving rise to very large fluctuations in 2D bond orientational order parameters. Applying low shear rates to initially disordered suspensions led to crystallization with the order parameter increasing gradually in time without large fluctuations, indicating that shear-induced crystallization of hard spheres does not proceed via a nucleation and growth mechanism. The authors concluded that the dynamics of melting and crystallization under shear differ dramatically from their counterparts in quiescent suspensions.

Colloidal systems have been reviewed several times. An early review focusing mainly on simulations for 2D systems of LJ particles is given by Abraham.^743^ A review by Li et al.,^744^ and dealing assembly and phase transitions, notes (among many other aspects) that (1) melting and freezing for HS colloidal systems occur at different densities, as already predicted by the simulation of Hoover and Ree^34^ in 1968; (2) defects do play a role; and (3) expansion of the liquid nucleus leads to lattice strain, so that under mild superheating there may be no kinetic pathway for melting. Further, they note that, as in atomic systems, melting only occurs in defect-free systems without surfaces in the neighborhood of interest. Moreover, they note that, although 2D systems have been extensively studied, the kinetics of HS systems received limited attention, as did attractive systems. Finally, the use of the KTHNY scenario requires infinite, defect-free crystal systems, while in practice both 2D and 3D are expected to premelt and melt from surfaces. In another contribution Li et al.^745^ discussed surface premelting in colloidal crystals composed of attractive particles, while Alsayed et al.^746^ discussed premelting at defects within bulk colloidal crystals. Wang et al.^747^ reviewed experiments and simulations conducted on superheating, melting, and premelting of colloidal crystals.

In 2022 Bini et al.^748^ also reviewed the phase behavior of colloidal systems in a rather detailed way. They indicate that a common modeling strategy to study the phase behavior is to represent nanoparticles as spheres interacting via effective potentials implicitly accounting for solvation effects. They consider nanoparticles as colloidal particles, albeit with more complex interactions including both attraction and repulsion, and review first studies exploring the phases of such systems having only attractive or repulsive interactions, so the general feature of the potentials can be in focus. Thereafter potentials with competing short-range attractions and average-long-range repulsions, better representing nanoparticles, are dealt with enabling interpretation of the appearance of novel phases, characterized by aggregates with different structural characteristics. The behavior discussed is interesting as such, but of more limited significance for atomic systems.

#### 10.3.2 Melting in 2D

According to the Kosterlitz–Thouless–Halperin–Nelson–Young (KTHNY) theory a 2D crystal melts in thermal equilibrium by two continuous phase transitions into an isotropic liquid state with an intermediate phase, commonly known as the hexatic phase. The KTHNY theory is a reoccurring feature in many 2D melting discussions.

Qi et al.^749^ studied the orientational order in the 2D melting transition using Brownian dynamics simulations for particles with a “soft” Yukawa potential. The authors reported a two-stage transition and the existence of a hexatic phase, consistent with the prediction of KTHNY theory. Based on their extensive simulations, the authors suggested that the breakdown of local order is qualitatively only to occur on a fractional part of the colloidal system for 2D melting, but that in 3D melting breakdown takes place over the whole system at the same time.

To study 2D systems Han et al.^750^ used *N*-isopropylacrylamide (NIPA) spheres, with the hydrodynamic diameter varying linearly from 950 nm at 20 °C to 740 nm at 30 °C and with a size dispersity of less than 3% as determined by dynamic light scattering measurements. Confining these particles between two glass plates, a dense monolayer of 800 nm spheres formed with crystal domains of about 40 μm^2^, corresponding to about 3000 particles, for which measurements were done on a central area of about 20 μm^2^ away from the grain boundaries as a function temperature to tune the particle volume fraction. A two-step melting mechanism from the crystal to a hexatic phase and from the hexatic to the liquid phase as a function of the volume fraction was observed. The authors considered a variety of sample properties during melting, such as the correlation function *g*(*r*), the structure factor *S*(*q*), topological defect densities, the dynamic Lindemann parameter, translational and orientational order parameters, and order parameter correlation functions in space and time. From a fairly detailed analysis it appeared that the order parameter susceptibility, i.e., the order parameter fluctuations, is superior for finding phase transition points compared to other analyses which typically suffer finite-size and/or finite-time ambiguities, although the order of these two phase transitions could not be unambiguously resolved due to limited temperature resolution.

Brodin et al.^751^ used glycerol droplets at the free surface of a nematic liquid crystalline layer of a 2D colloidal system. They also conclude that melting occurs through an intermediate hexatic phase, as predicted by KTHNY theory. However, the temperature range of the intermediate phase was rather narrow, less than about 1 °C, and the characteristic critical power law decays of the correlation functions were not fully developed. The authors concluded that the melting of these 2D systems qualitatively occurs according to KTHNY theory, but that quantitative details of the transition may partly depend on the details of interparticle interaction. The melting of quasi-2D colloidal hard spheres in relation to the hexatic phase was also studied by Thorneywork et al.^752^ by considering a tilted monolayer of 2.79 μm diameter melamine formaldehyde spheres in sedimentation–diffusion equilibrium. The authors measured the equation of state from the density profiles and used time-dependent and height-resolved correlation functions to identify the liquid, hexatic, and crystal phases. While the liquid–hexatic transition appeared to be discontinuous, the hexatic–crystal transition was shown to be continuous. They measured the width of the liquid–hexatic coexistence gap from the fluctuations of the corresponding interface and thereby experimentally established the full phase behavior of hard disks.

Various models based on dynamical and structural properties to identify the crystal–hexatic and hexatic–isotropic liquid phase transitions for 2D melting of colloidal systems have been compared by Dillman et al.^753^ using superparamagnetic spherical particles arranged in a 2D monolayer at a water–air interface. These particles consisted of spherical polystyrene spheres, with diameter *d* = 4.5 μm, in which Fe_2_O_3_ nanoparticles were embedded, which were suspended in water and sterically stabilized with sodium dodecyl sulfate. As these particles have a density of ≅1.5 g cm^–3^, a droplet containing the particles was fixed in a 6 mm hole of a top-sealed glass plate by surface tension, thereby creating a particle loaded water–air interface. The (repulsive) dipolar interaction between the particles was controlled by a magnetic field perpendicular to the water–air interface. The authors used a monochrome CCD camera to observe the particles by video microscopy with the field of view (1158 × 865 μm^2^) showing ≈9 × 10^3^ particles for a system containing ≈2.5 × 10^5^ particles in total. During data acquisition the coordinates of the particles in the field of view were determined in situ every ≅2 s over a period of 25 min by digital image processing with an accuracy of about 50 nm.

To identify the solid–hexatic–liquid transitions properly, the authors used various models, as discussed below. Local measures, like the local bond order (Larson–Grier) criterion measuring how the neighbors of a particle fit locally on a hexagonal lattice, and the shape factor of Voronoi cells defined by 4*C*^2^/4π*S*, where *C* is the circumference and *S* us the area of the Voronoi cell, appeared to change not significantly on crossing the transition temperatures since the local order in 2D systems is 6-fold in both the fluid and the solid phases, and these criteria are rather insensitive to global symmetry changes. For the Hansen–Verlet rule modified for 2D systems, measuring the height of the first peak of the structure factor *S*(*q*) at melting, values between *S*(*q*_0_) ≅ 4.4 and *S*(*q*_0_) ≅ 5.75 have been reported in simulations, while in this dipolar system *S*(*q*_0_) ≅ 10 at *T*_mel_, so this criterion with a quasi-universal critical value should be used with care. The Löwen–Palberg–Simon criterion, the ratio of the long-time and short-time diffusion coefficients, states that crystallization in 3D systems takes place at a critical ratio of 0.1. In 2D values between 0.072 and 0.099 were obtained by simulations, whereas in this system a value of 0.03 resulted, the discrepancies possibly being due the presence of grain boundaries in the simulations, and therefore this criterion is also to be used with care. Minkowski functionals (see, e.g., ref (754)) as topological measures to identify the transitions were also used. In brief, in *n*D one has *n* + 1 Minkowski measures for a structure. In 3D they are the total volume, the total interfacial area, the mean curvature, and the total curvature of the system. In 2D the Minkowski measures are related to the surface area *A*, the circumference *U*, and the Euler characteristic χ = *N*_con_ – *N*_hole_, the difference between the number of connected surfaces *N*_con_ and number of holes *N*_hole_. While these measures appeared to be sensitive to locally heterogeneous distributions of particles in a binary mixture, they appeared to be also rather insensitive to global symmetry changes and phase transitions.

Overall, it seems that, for this system with long-range repulsion, the bond order correlation function *g*_6_(*r*) ≡ *g*_6_(|**r**_*l*_ – ***r***_*k*_|) = ⟨ψ_6_*(***r***_*l*_) ψ_6_(***r***_*k*_)⟩ between particles located at ***r***_*l*_ and ***r***_*k*_ and where ψ_6_(***r***_*k*_) = *n*_nn_^–1^∑_1_^*n*_nn_^ exp(i6θ_*kl*_) with *θ*_*kl*_ the angle between the *n*_nn_ nearest-neighbor particles *l* of particle *k* and its associated bond order susceptibility, worked best to identify the hexatic–isotropic liquid transition. A 2D dynamic Lindemann parameter, given by γ = (Δ***u***_*j*_ – Δ*u*_*j*+1_)^2^/2*a*^2^, where Δ***u***_*j*_ = ***u***_*j*_(*t*) – ***u***_*j*_(0) with ***u***_*j*_(*t*) the displacement of particle *j* at time *t* with respect to its nearest neighbors *j* + 1 and normalized to the average interparticle distance *a*, appeared to identify unambiguously the hexatic–crystalline transition.

In his review on theoretical methods and experimental issues for various 2D systems, melting in relation to an hexatic phase was discussed by Murray,^755^ while Gasser et al.^756^ provide a more general review on 2D melting of colloidal systems.

To conclude this section, it is fair to say that colloidal and atomic systems show similarities as well dissimilarities. While the former can help to elucidate issues for the atomic scale, as time and size scales are more easily accessible, the latter renders colloidal systems a topic in its own right, being able to vary interactions, size, and size dispersity.

## 11 Melting in Perspective

Now changing to a perspective view, from all we have said in this review, a few specific and generic observations can be made. We start with the specific ones.

• Despite its age, the Lindemann rule is still an important player in the explanation of melting. However, the various existing formulations neither always agree nor are always clear about which Lindemann parameter is used. As noted, various authors disagree on whether good, reasonable, or poor predictions are made.

• The dislocation, vacancy, and interstitialcy models are limited to metals. The first two have been used for many metals, while the interstitialcy model has been mainly substantiated by data on Cu. The first two types of models have been applied both to mechanical (or bulk) melting and to thermodynamic (or surface-mediated) melting.

• Many MD simulations studies discuss primarily energy and entropy without paying too much attention to the mechanisms. Although some attempts have been made to define a proper characteristic for a particular mechanism, the lack of attention to discern mechanisms is partially due to the difficulty of defining such a characteristic and, once chosen, such a characteristic generally does not catch other mechanisms.

• For both normal and high-pressure conditions, rather similar models can produce rather different results. Differences between the various Debye temperatures and Grüneisen parameters and their volume or pressure dependence that can be defined^8^ are often not considered. Moreover, experimental differences between shock wave and diamond anvil cell results contribute further to the confusion.

• Atomic and colloidal systems can show substantial similarities as well as significant dissimilarities.

More generic observations are the following:

• Despite the fact that melting is a familiar and well-defined phenomenon, formulating a general theory of melting appeared to be rather intractable and none of the proposed models or explanations is generally accepted. This stands in contrast to continuous transitions where renormalization group theory has provided a general framework. Although Bruno and Sak^757^ presented a modification of the renormalization group for discontinuous transitions, this seems not to have been used for melting, possibly, as pointed out by Yukalov,^684^ because such methods do not take into account the fluctuations in both phases. However, at least for clusters and nanoparticles, there are several molecular dynamics studies explicitly dealing with melting mechanisms, which are reliably singled out if the starting structure is correct.

• It will be clear that a straightforward calculation of enthalpy and entropy for both phases provides a scheme for a general theory. However, as a generally accepted liquid state model has not materialized (yet), the focus is directed on one-phase models, but a generally accepted one-phase theory has not materialized either. The lack of such a more general one-phase theory for various types of materials may also be due, at least partially, to the fact that many melting studies focused on a limited group of related compounds, thereby not paying attention to aspects relevant to other material groups.

•The difference between mechanical (or bulk) melting and thermodynamic (or surface-mediated) melting has become abundantly clear. This has evident bearing on various phenomena. In the presence of a relevant surface, thermodynamic melting is normally observed, and proper understanding of its size effect is required for such different applications as the control of solubility for pharmaceuticals and the stability of nanocomposites. However, bulk melting does occur in the absence of relevant surfaces and is most important for some other conditions, e.g., for shock wave experiments.

• While most models as discussed in sections 5 and 6 aim at estimating *T*_mel_, some models, as noted in section 10, are providing only qualitative mechanistic arguments.

To become even more generic, we note that the challenges associated with formulating a general mechanistic theory of melting are linked to the following aspects:

• Melting is conventionally considered as a physical process. Although many solids melt by redistributing relatively weak vdW forces, many other solids melt by redistributing relatively strong interactions, akin to chemical bonding.

• Although melting involves generally clear characteristics, such as a precise melting point, loss, or long-range order and reversibility, there are a large variety of interactions between the constituents.

• As we have seen on several occasions before, the (structural) information available for liquids is much less than that for solids. This is reinforced by difficulties to obtain quantitative information for the interface between a melting solid and a liquid. Interfaces may or may not roughen, as described by the continuum Kosterlitz–Thouless scenario or the discrete solid on solid model.

• In many cases, small amounts of impurities may obscure the nature of the melting process by exhibiting disproportional effects, in particular for the onset of fusion.

Such a wide range of aspects might be rather difficult to catch in one model.

Therefore, in conclusion, despite the ubiquitous presence of melting, it is fair to say that although the thermodynamics are well understood the mechanisms involved are much less so. Nearly all types of defects have been proposed. Although in 3D melting dislocations provide only one of the routes, in 2D melting dislocations definitely do play an important role and an extensive review has been provided by Joós.^237^ Apart from the general materials science problem that various types of materials may be controlled by different mechanisms, even for one type of material, several options exist to explain many of the phenomena involved. Indeed, several theories can describe the overall behavior of melting reasonably well, and possibly because of that, some authors claim universal applicability of their theory but usually illustrate it by using data for one type of material only. Nevertheless, it is clear that for normal melting surfaces are an important factor for all types of materials. This is most clearly illustrated by the decrease in melting temperature for nanosized particles. Alternative, attractive simple views still surface regularly, so a final verdict is hardly possible, although it may well be that a generally applicable mechanistic theory, in view of the many aspects that require attention, does not exist.

## Abbreviations

BCC: body centered cubic
CP: critical point
DFT: density functional theory
DTM: dislocation theory of melting
EAM: embedded atom potential
EoS: equation of state
FCC: face centered cubic
G: gas
GGA: generalized gradient approximation
HCP: hexagonal close packed
HRT: hierarchical reference theory
L: liquid
LDA: local density approximation
LJ: Lennard-Jones
LJD: Lennard-Jones–Devonshire
MD: molecular dynamics
MC: Monte Carlo
NN: nearest neighbor
NNN: next nearest neighbor
OZ: Ornstein–Zernike
PoCS: principle of corresponding states
QCE: quantum cluster equilibrium
S: solid
SC: simple cubic
SCPT: self-consistent phonon theory
TP: triple point
V: vapor
XRD: X-ray diffraction
**Symbols**
*C*_*X*_: heat capacity (at constant *X* = *V* or *P*)
*E*: energy
*F*: Helmholtz energy
*G*: Gibbs energy
*H*: enthalpy
*K*_*X*_: bulk modulus (at constant *X* = *T* or *S*)
*M*: molar mass
*N*: number of molecules
*N*_A_: Avogadro number
*P*: pressure
*Q*: configurational partition function
*R*: gas constant
*S*: entropy
*T*: temperature
*U*: internal energy
*V*: volume
*Z*: partition function
ℏ: Planck constant *h*/2π
*k*: Boltzmann constant
*m*: mass
*n*: number of moles, refractive index
*u*: displacement
*z*: coordination number, single particle partition function
*Λ*: thermal length
*Ω*: atomic volume
α: thermal expansivity
β: 1/*kT*
γ: Grüneisen parameter, surface energy, parameter
ε: characteristic energy
ϕ: potential
θ_D_: Debye temperature
θ_E_: Einstein temperature
μ: shear modulus, chemical potential
ν: frequency, Poisson ratio
ω: circular frequency
ξ: Lindemann–Gilvarry parameter
ρ: density
σ: characteristic size
ω: circular frequency
ω_D_: Debye frequency
ω_E_: Einstein frequency
